# Mapping of quorum sensing interaction network of commensal and pathogenic staphylococci

**DOI:** 10.1128/mbio.00967-25

**Published:** 2025-07-16

**Authors:** Bengt H. Gless, Benjamin S. Sereika-Bejder, Iben Jensen, Martin S. Bojer, Katerina Tsiko, Sabrina H. Schmied, Ludovica Vitolo, Bruno Toledo-Silva, Sarne De Vliegher, Hanne Ingmer, Christian A. Olsen

**Affiliations:** 1 Center for Biopharmaceuticals and Department of Drug Design and Pharmacology, Faculty of Health and Medical Sciences, University of Copenhagen 53139 , Copenhagen, Denmark; 2 Department of Veterinary and Animal Sciences, Faculty of Health and Medical Sciences, University of Copenhagen 555025, Frederiksberg C, Denmark; 3 M-team & Mastitis and Milk Quality Research Unit, Department of Internal Medicine, Reproduction and Population Medicine, Faculty of Veterinary Medicine, Ghent University, Salisburylaan, Merelbeke, Belgium; University of Pretoria, Pretoria, Gauteng, South Africa

**Keywords:** autoinducing peptides, cyclic peptides, RiPPs, skin microbiome, anti-virulence, bacterial interactions, MRSA

## Abstract

Staphylococci utilize secreted autoinducing peptides (AIPs) to regulate group behavior through a process called quorum sensing (QS). For pathogenic staphylococci, such as *Staphylococcus aureus*, QS regulates the expression of major virulence factors, and QS inhibition has been proposed as an alternative to antibiotics for the treatment of infections with methicillin-resistant *S. aureus* (MRSA). Here, we surveyed the interaction map between QS systems of the pathogens *S. aureus, Staphylococcus epidermidis,* and *Staphylococcus lugdunensis* and all the currently known staphylococcal AIPs, covering 21 different species. We identified six of these ribosomally synthesized and post-translationally modified peptides (RiPPs) in this study and compiled the full collection of AIPs by chemical synthesis. The resulting mapping provided 280 QS interactions that were divided into human- and animal-associated staphylococci, showing substantial differences in inhibitory potencies between the groups. AIPs of the bovine-associated species *Staphylococcus simulans* displayed potential as QS inhibitors toward the investigated strains and were therefore chosen as a starting point for a structure-activity relationship study. This study provides insights into the requirements for QS interference, yielding the most potent inhibitors reported to date for *S. epidermidis* and *S. lugdunensis*. Furthermore, we tested an *S. simulans* AIP as an anti-virulence agent in an assay to assess the risk of acquired suppression of the inhibitory effect, and we established an assay set-up to successfully monitor *agr* deactivation of virulent MRSA by the QS inhibitor. Finally, a peptide was shown to attenuate skin infection caused by MRSA in a mouse model. Our results reveal a complex network of staphylococcal interactions and provide further impetus for the investigation of QS modulation in the targeting of antibiotic-resistant pathogens.

**IMPORTANCE:**

Bacteria from the *Staphylococcus* genus produce macrocyclic peptides, called autoinducing peptides (AIPs), used in inter-cell communication with their kin. Differences in AIP sequence and length produced by different staphylococcal species can result in communication interference, altering the physiology of co-inhabiting staphylococci in complex microbiotas. Opportunistic pathogens like *Staphylococcus aureus* regulate the expression of toxins using this peptide-mediated communication, and its inhibition has, therefore, been proposed as a strategy to target infections caused by methicillin-resistant *S. aureus* (MRSA). The systematic mapping of AIP activities, structure-activity relationship studies, and evaluation of resistance development provided in this paper, therefore, serve as a resource for the future discovery of inhibitory peptides for the investigation of bacterial communication.

## INTRODUCTION

**Fig 1:**
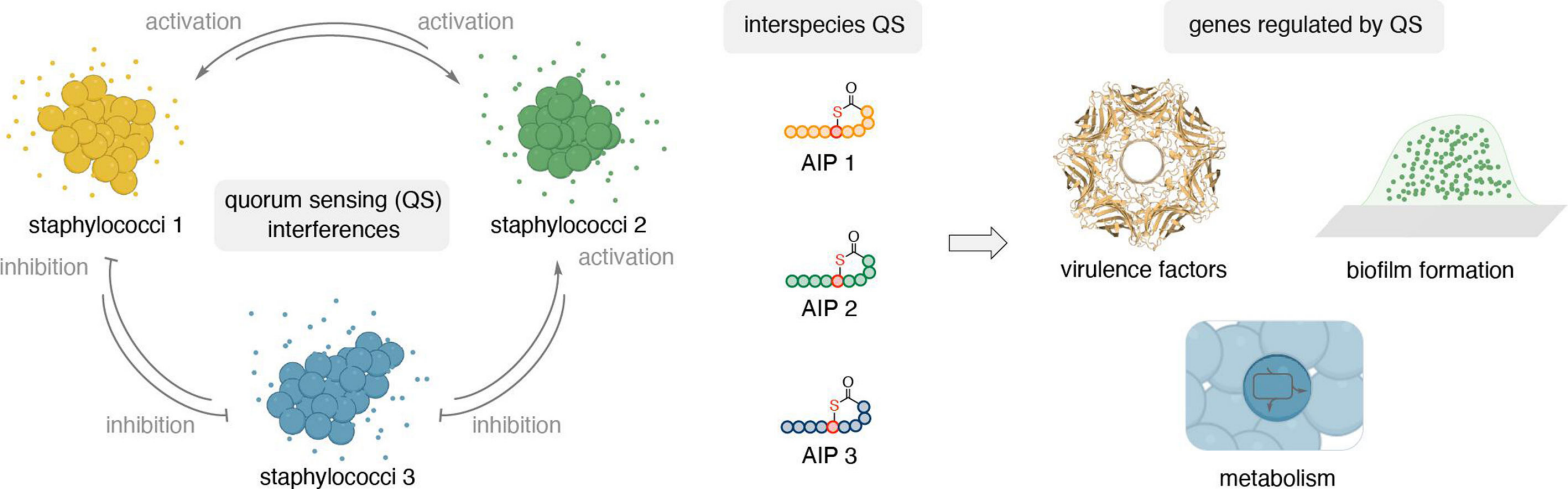
Quorum sensing interference of co-inhabiting staphylococci. The QS interference within habitats of multiple staphylococcal species is complex as each staphylococcal species and *agr* specificity group secretes a unique AIP. Co-inhabiting staphylococci are exposed to non-cognate AIPs, which can interfere with their QS systems depending on their inhibitory potency and thereby alter gene expression and change group behavior.

Staphylococci are Gram-positive bacteria that frequently colonize humans and animals, representing some of the most abundant microbes found in the human microbiota (1). Among the numerous staphylococcal species, there are harmless commensal species, whereas others, especially *S. aureus*, are pathogenic. All staphylococci have genes encoding a quorum sensing (QS) system that enables changes in group behavior and gene expression in response to cell density (2, 3). This cell-to-cell communication plays an important role in the transition from harmless skin colonizer to invasive pathogen and is regulated through the secretion and detection of autoinducing peptides (AIPs), which are 7–12 residue peptides, containing a characteristic thiolactone peptide cycle at the C-terminus (lactone for *Staphylococcus intermedius* group) (4–6). The AIP-regulated QS machinery is encoded by a chromosomal locus termed *accessory gene regulator* (*agr*), which controls the expression of genes involved in biofilm formation, surface adhesion, and toxin production as well as the Agr proteins involved in the QS process (Fig. S1) (5, 6). The *agr* system has been studied in detail for *S. aureus,* but *agr* loci are found in all staphylococci, with each species utilizing a unique AIP as a QS signaling molecule. Another key feature of AIP secretion is QS interference with *agr* systems of other staphylococcal species and *agr* specificity groups within the same species (2, 7). This phenomenon has been thoroughly studied for *S. aureus,* and many non-cognate AIPs act as potent inhibitors of its QS system (8–15). QS interference has been less studied in other staphylococci; however, it might be a common occurrence in shared habitats of staphylococci, resulting in altered gene expression levels of co-inhabiting species susceptible to *agr* inhibition by non-cognate AIPs (Fig. 1).

Determining the influence of QS interference on bacterial multi-species human and animal microbiotas is difficult due to their complex nature and the multitude of non-QS interactions (3). Nevertheless, recent advances focused on QS interference with *S. aureus* by commensal staphylococci in the context of atopic dermatitis and therapy (12, 16, 17). Early phase clinical studies have shown a beneficial effect of the secreted inhibitory AIPs of *Staphylococcus hominis* on the outcome of atopic dermatitis caused by *S. aureus* as a result of QS inhibition (18). These are promising results from the perspective of investigating anti-virulence strategies based on QS inhibition as alternatives to traditional treatment with antibiotics or as synergistic options together with antibiotics (19, 20).

QS inhibitor development had and still has a focus on the AIPs of *S. aureus* and structure-activity relationship studies thereof, which afforded potent inhibitors of *S. aureus* itself (21–24). However, recent developments in technologies for the identification of AIP have led to a significant increase in known non-aureus staphylococcal and mammaliicoccal AIPs (10–15, 25). Several of these AIPs are potent QS inhibitors of *S. aureus* displaying effective *in vivo* attenuation of infections caused by methicillin-resistant strains of *S. aureus* (MRSA) (10, 13–15). The effects of non-cognate AIPs on *agr* systems different from *S. aureus* have been scarcely investigated, including that of the common skin colonizer *S. epidermidis*, despite its abundance on the human skin (26–29). The roles of *S. epidermidis* as a symbiont are manifold (30) with recent studies even showing a beneficial role for the host (31, 32) while at the same time being able to cause medical device infections (33). A few studies have investigated modifications to the cognate AIPs to create activators and inhibitors of its *agr* system (28, 34, 35). Similarly, the human skin commensal *S. lugdunensis* has been reported to cause severe endocarditis (36), and QS interference with its *agr* system has been rarely investigated (37).

Thus, the substantial increase in recently identified AIPs, combined with the lack of exploration of their interactions, encouraged us to systematically map the QS interactions of all known AIPs (**1**–**35**) , with eight *agr* reporter strains of three therapeutically relevant species *S. aureus*, *S. epidermidis*, and *S. lugdunensis*. Our motivation was 2-fold: first, to create a defining data set of QS interactions as a resource to explore trends between bacterial species in the same habitat, and second, to discover potent inhibitory interactions, especially against the less studied *agr* systems of *S. epidermidis* and *S. lugdunensis*.

## RESULTS

### Identification of new autoinducing peptides and quorum sensing interaction map

**Fig 2:**
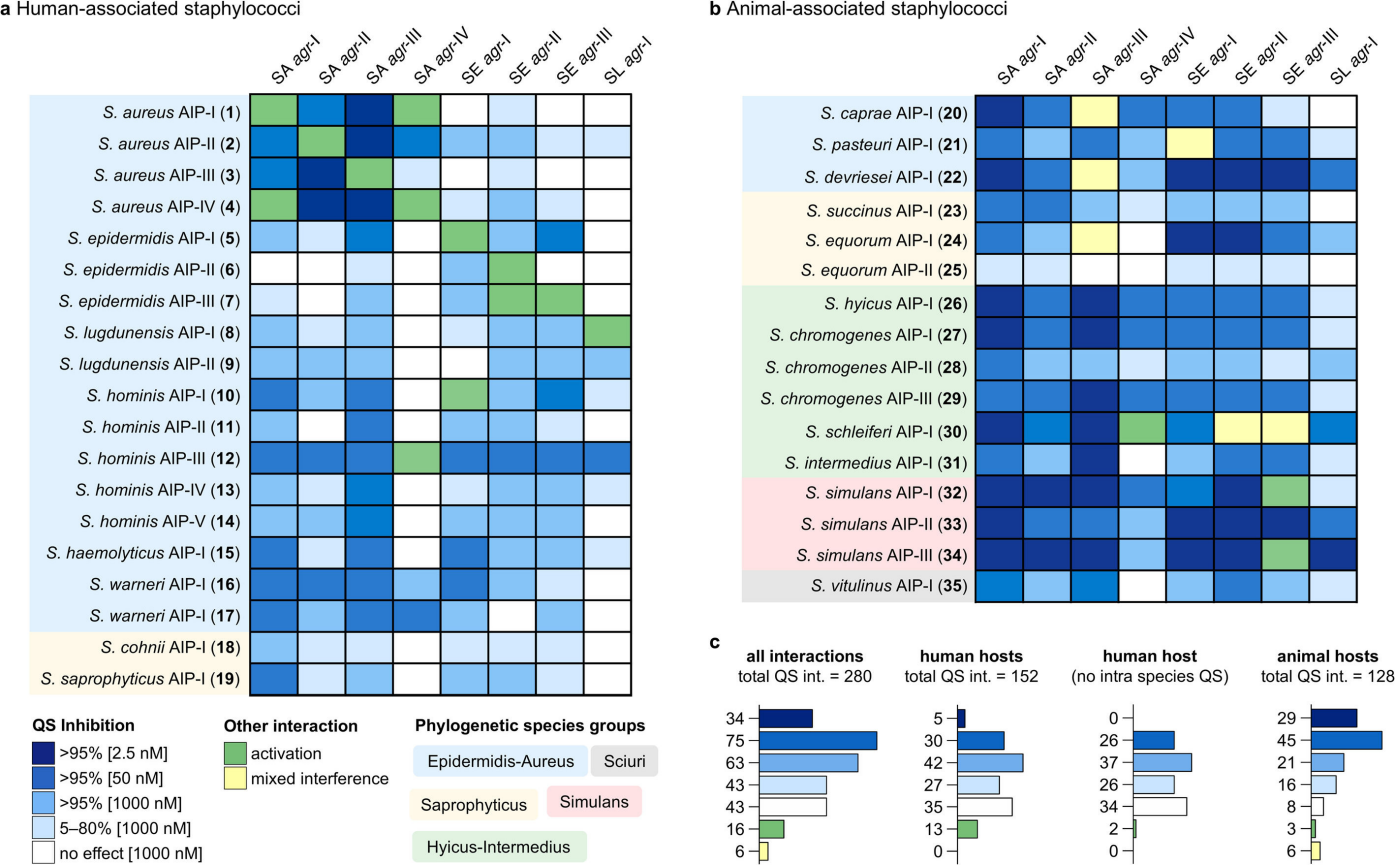
Quorum sensing interaction map. (**a**) Map of human-associated staphylococci. (**b**) Map of animal-associated staphylococci. (**c**) Summary of QS interactions with human and animal hosts. Synthetic AIPs were tested at several concentrations (1,000 nM, 50 nM, 2.5 nM) against fluorescent reporter strains of *S. aureus* (SA) *agr*-I–IV, *S. epidermidis* (SE) *agr*-I–III, and *S. lugdunensis* (SL) *agr*-I to assess their QS modulation abilities. Blue shading of boxes represents different potencies of QS inhibition (>95% at 2.5 nM, 50 nM, and 1,000 nM or 5%–80% at 1,000 nM), white boxes represent no interaction at 1000 nM, green boxes represent QS activation, and yellow boxes represent “mixed interference” (activation at 1,000 nM and inhibition at 50 nM or partial inhibition over a range of concentrations).

We previously developed a native chemical ligation-based trapping method for the rapid identification of AIPs from bacterial supernatants, by exploiting the chemo-selective reaction between thioesters and N-terminal cysteine residues (38), which led to a substantial increase in the number of known AIPs (Fig. S2) (11). Here, we report the additional identification of six previously unidentified AIPs from a collection of human and animal isolates, namely *Staphylococcus cohnii* AIP-I (**18**), *Staphylococcus pasteuri* AIP-I (**21**), *Staphylococcus devriesei* AIP-I (**22**), *Staphylococcus succinus* AIP-I (**23**), as well as *Staphylococcus equorum* AIPs I (**24**) and II (**25**) (Fig. S2 to S8). This elevates the number of currently known staphylococcal AIPs to 37 (35 unique structures [**1**–**35**], originating from 21 staphylococcal species from across five of the six phylogenetic species groups as classified through multi-locus sequence typing (Table S1) (39). In order to create a comparative and reproducible data set of QS interactions of all known AIPs against *S. aureus*, *S. epidermidis*, and *S. lugdunensis*, we utilized widely used fluorescent reporter strains, which have a naturally functioning *agr* system, modified to produce green fluorescent protein (GFP)/yellow fluorescent protein (YFP) once the *agr*-dependent promoter P3 is activated (40). As a first step, we compiled our library of 35 unique AIPs by chemical synthesis (Schemes S1 to S3) and established an assay setup in which the peptides were initially screened at 1 μM and at 50 nM concentration. In cases where we observed >75% inhibition at 50 nM AIP concentration, lower concentrations of 2.5 nM and 0.125 nM were tested (Fig. 2; Fig. S9 to S15).

To make sure that our systematic survey, using fluorescent reporter strains, correlated with previously reported QS inhibition values, we determined half maximal inhibitory concentration (IC_50_) values for selected AIPs in both fluorescent reporter strain assays (Fig. S16 to S18) and β-lactamase reporter strain assays for QS inhibition of *S. aureus agr*-I–IV (Fig. S19 to S22) (11), which showed excellent correlation between single concentration data points and full dose-response experiments as well as with previously reported QS inhibition values collected from the literature (Tables S2 to S4).

All staphylococcal species were divided into human- and animal-associated species based on their most commonly reported hosts (1, 41–44), where *S. aureus*, a known colonizer of both humans and animals, was included in the human group. The resulting maps contain 280 QS interactions of native AIPs, representing the largest resource of its kind (Fig. 2). The likelihood of interactions between certain human and animal-associated species might be low; however, these interactions still represent a promising source for the discovery of potent QS inhibitors. Most measured QS interactions were inhibitory (221 of 280, 79%), where a clear difference between AIPs from human- and animal-associated species could be observed (Fig. 2). The QS interactions of human-associated AIPs with *S. aureus*, *S. epidermidis,* and *S. lugdunensis* only exhibited >95% inhibition at 2.5 nM AIP concentration for intra-species interferences between *S. aureus* specificity groups. Furthermore, most of the combinations that produced no effect at an AIP concentration of 1 μM were from the human-associated group of AIPs (35 of 43, 81%) (Fig. 2a and c). In contrast, most QS interactions of animal-associated AIPs (74 of 128, 58%) reached >95% inhibition at 2.5 nM or 50 nM AIP concentration (Fig. 2b and c). The seven AIPs from species primarily associated with bovine colonization (*Staphylococcus hyicus, Staphylococcus chromogenes,* and *S. simulans*) displayed potent interactions, responsible for more than half of the examples of AIPs exhibiting >95% inhibition at 2.5 nM concentration (Fig. 2c).

All AIPs that increased the endpoint fluorescence readout compared with untreated controls were monitored continuously overnight for growth and fluorescence, along with all previously known activators (Fig. 2a and b, shown in green, and Fig. S23 and S24). We concluded that if the addition of synthetic AIP gave rise to either a higher endpoint fluorescence or early induction of activation of the reporter, compared with the untreated control, this could be interpreted as the ability to activate the non-native *agr* system (Fig. S23 and S24). Often, an early increase in fluorescence output was accompanied by a delay in growth, possibly due to the metabolic burden of producing fluorescent protein and QS regulated gene products. Interestingly, we found *S. epidermidis* AIP-III (**7**) to be an activator of *S. epidermidis agr*-II, in contrast to previous experiments with bacterial supernatant where the AIP had no reported effect (45). Furthermore, several cross-species activators were discovered: *Staphylococcus hominis* AIP-I (**10**) activated *S. epidermidis agr*-I, *S. hominis* AIP-III (**12**) activated *S. aureus agr*-IV, and *S. simulans* AIP-I (**32**) and AIP-III (**34**) activated *S. epidermidis agr*-III. We observed inconsistent inhibition behavior of several AIPs (**20-22**, **24,** and **30**) against some reporter strains (Fig. 2b, shown in yellow and termed “mixed interference”). This behavior manifested itself either by causing activation at 1 μM and inhibition at lower concentrations or by showing 70% inhibition at multiple concentrations from 2.5 to 1,000 nM, which was also recently observed for analogs of *S. epidermidis* AIPs (35).

The QS interaction map identified peptides that were inhibitory across all staphylococcal reporter strains, namely *S. hyicus* AIP-I (**26**) and *S. chromogenes* AIP-I (**27**), which potently inhibited all *S. aureus* and *S. epidermidis agr* variants, but only weakly inhibited *S. lugdunensis agr*-I as well as *S. simulans* AIP-II (**33**), which inhibited all eight *agr* systems (Fig. 2b). Interestingly, many AIPs that acted as strong inhibitors across *S. aureus*, *S. epidermidis,* and *S. lugdunensis* (**26**, **27**, **29**–**34**) were 9-mer peptides with positively charged residues at the N-terminus (Table S1). We found the AIPs of *S. simulans* interesting because they displayed strong QS interaction profiles, including the most potent inhibition of *S. lugdunensis* and the activation of *S. epidermidis agr*-III by *S. simulans* AIPs I (**32**) and III (**34**) but not by *S. simulans* AIP-II (**33**). We therefore performed a structure-activity relationship study to glean further insights about the function of these molecules.

### Structure-activity relationship study of autoinducing peptides of *S. simulans*

**Fig 3:**
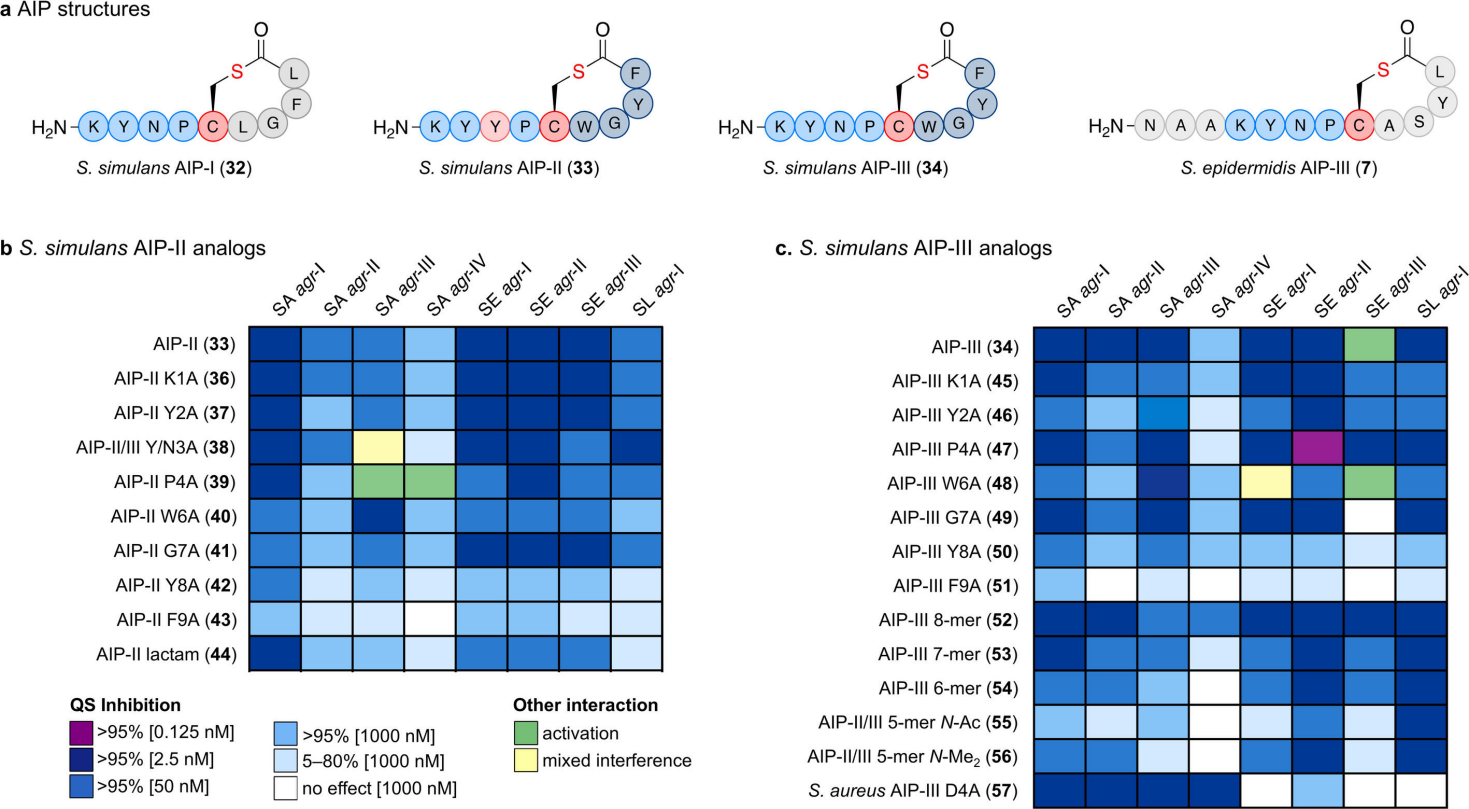
Structure-activity relationship study of *S. simulans* AIP-II (**33**) and AIP-III (**34**). (**a**) Structures of *S. simulans* AIP-I–III (**32**–**34**) compared with *S. epidermidis* AIP-III (**7**). (**b**) Alanine scan of *S. simulans* AIP-II (**33**). (**c**) Alanine and truncation scan of *S. simulans* AIP-III (**34**). Synthetic peptides were tested at several concentrations (1,000 nM, 50 nM, 2.5 nM, 0.125 nM) against fluorescent reporter strains of *S. aureus* (SA) *agr*-I–IV, *S. epidermidis* (SE) *agr*-I–III, and *S. lugdunensis* (SL) *agr*-I. Shading of boxes represents different potencies of QS inhibition (>95% at 0.125 nM, 2.5 nM, 50 nM, and 1,000 nM or 5%–80% at 1000 nM), white boxes represent no interaction at 1,000 nM, green boxes represent QS activation, and yellow boxes represent “mixed interference” (activation at 1,000 nM and inhibition at 50 nM or partial inhibition over a range of concentrations).

*S. simulans* is primarily an animal-associated staphylococcal species, commonly found in bovine livestock (46), although human infections have also been documented, particularly involving *agr*-I type strains (13). The species has three confirmed AIPs (**32**–**34**), which share structural features (11, 13). *S. simulans* AIPs I (**32**) and III (**34**) share an identical exo-tail sequence, KYNP, which is also part of the exo-tail of *S. epidermidis* AIP-III (**7**) and could therefore explain the activating properties toward *S. epidermidis agr*-III (Fig. 3a). Furthermore, *S. simulans* AIPs II (**33**) and III (**34**) share an identical macrocycle and are both highly potent inhibitors of *S. lugdunensis* QS, compared with the *S. simulans* AIP-I (**32**) (Fig. 3a). As a starting point for our structure-activity relationship study, we determined the IC_50_ values of *S. simulans* AIP-I–III (**32**–**34**) giving sub- or low nanomolar potencies against *S. aureus agr*-I–III groups as well as *S. epidermidis agr*-I–II (Fig. S25 to S27). For *S. aureus agr*-IV, the IC_50_ value was slightly lower for **32** (10 nM) compared with **33** (32 nM) and **34** (34 nM) and against *S. lugdunensis agr*-I, **33** and **34** displayed sub- or low-nanomolar potencies with the IC_50_ value of **34** (0.48 nM) being 400-fold lower compared with the most potent, previously reported inhibitors of *S. lugdunensis* (37). *S. simulans* AIP-II (**33**) displayed sub-nanomolar inhibition against *S. epidermidis agr*-III, whereas **32** and **34** acted as activators (Fig. S24).

Based on these results, we conducted alanine scans on both *S. simulans* AIPs-II (**33**) and III (**34**) and tested the peptides to identify important residues for the activation of *S. epidermidis agr*-III and the inhibition of *S. lugdunensis* (Fig. 3b and c; Fig. S28 to S32). The alanine mutations of *S. simulans* AIP-II (**33**) starting from the N-terminus, K1A-II (**36**) and Y2A-II (**37**), showed minor effects on the QS interaction profile (Fig. 3b). The Y3A-II mutant (**38**) also represents the N3A-III mutation of *S. simulans* AIP-III (**34**) as these peptides differ only in this position, and this common mutant acted as an inhibitor of *S. epidermidis agr*-III. Interestingly, the mutant P4A-II (**39**) became an activator of *S. aureus agr*-III and *agr*-IV, which we confirmed in a continuous assay (Fig. S23). Changes to the macrocycle in W6A-II (**40**) resulted in decreased inhibitory potency, except against *S. aureus agr*-III, while reducing structural flexibility by substituting glycine in G7A-II (**41**) furnished a decrease against all *S. aureus* strains. In agreement with the previous consensus (47), mutations to the C-terminal hydrophobic residues F8A-II (**42**) and L9A-II (**43**) led to a significant loss in potency against all reporter strains. A thioester-to-amide analog of **33** (peptide **44**) (Supplementary Scheme S4) resulted in a decrease in potency against all species apart from *S. aureus agr*-I (Fig. 3b). For the alanine scan of *S. simulans* AIP-III (**34**), the two N-terminal mutants K1A-III (**45**) and Y2A-III (**46**) both lost the ability to activate *S. epidermidis agr*-III and showed reduced overall inhibitory potencies (Fig. 3c). The proline mutation P4A-III (**47**) led to an increase in inhibition of *S. epidermidis* and *S. lugdunensis* and was the only tested peptide resulting in >95% inhibition at 0.125 nM AIP concentration. Interestingly, the mutation W6A-III (**48**) in the macrocycle had no effect on *S. epidermidis agr*-III activation while otherwise leading to a weaker inhibition profile. Like G7A-II (**41**), G7A-III (**49**) also had less effect on inhibition but led to the loss of activation of *S. epidermidis agr*-III. Finally, substitution of the two hydrophobic C-terminal residues in F8A-III (**50**) and L9A-III (**51**) led to diminished potency of the peptides, as also observed for *S. simulans* AIP-II above. Next, we performed a truncation scan of *S. simulans* AIP-III (**34**), revealing that the inhibition of *S. aureus* was generally reduced by each truncation going from octamer to pentamer length (**52**–**56**), with the N-terminus of the pentamer being either acetylated or bis-N-methylated (48), to circumvent spontaneous rearrangement to the corresponding homodetic pentamer (48, 49) (Fig. 3c). The only exception was an increased inhibition of *S. aureus agr*-IV by **52,** and the truncations had minor effects on *S. epidermidis* until the 6-mer (**54**), except for the loss of *S. epidermidis agr*-III activation. As anticipated, the macrocycle represented the key feature for potent inhibition of *S. lugdunensis agr*-I as all truncations including a 5-mer with di-methylated N-terminus (**56**) remained highly potent. Finally, we included a known inhibitor of all *S. aureus agr* groups, *S. aureus* AIP-III D4A (**57**) (22) , and observed potent inhibition of *S. aureus* with the only observed >95% inhibition at 2.5 nM against *S. aureus agr*-IV but weak interference with *S. epidermidis* and *S. lugdunensis* (Fig. 3c).

Having established a foundation to design future QS inhibitors based on *S. simulans* AIPs, we were interested in examining the potential of such compounds as anti-virulence agents.

### Autoinducing peptide of *S. simulans* as anti-virulence agent

**Fig 4:**
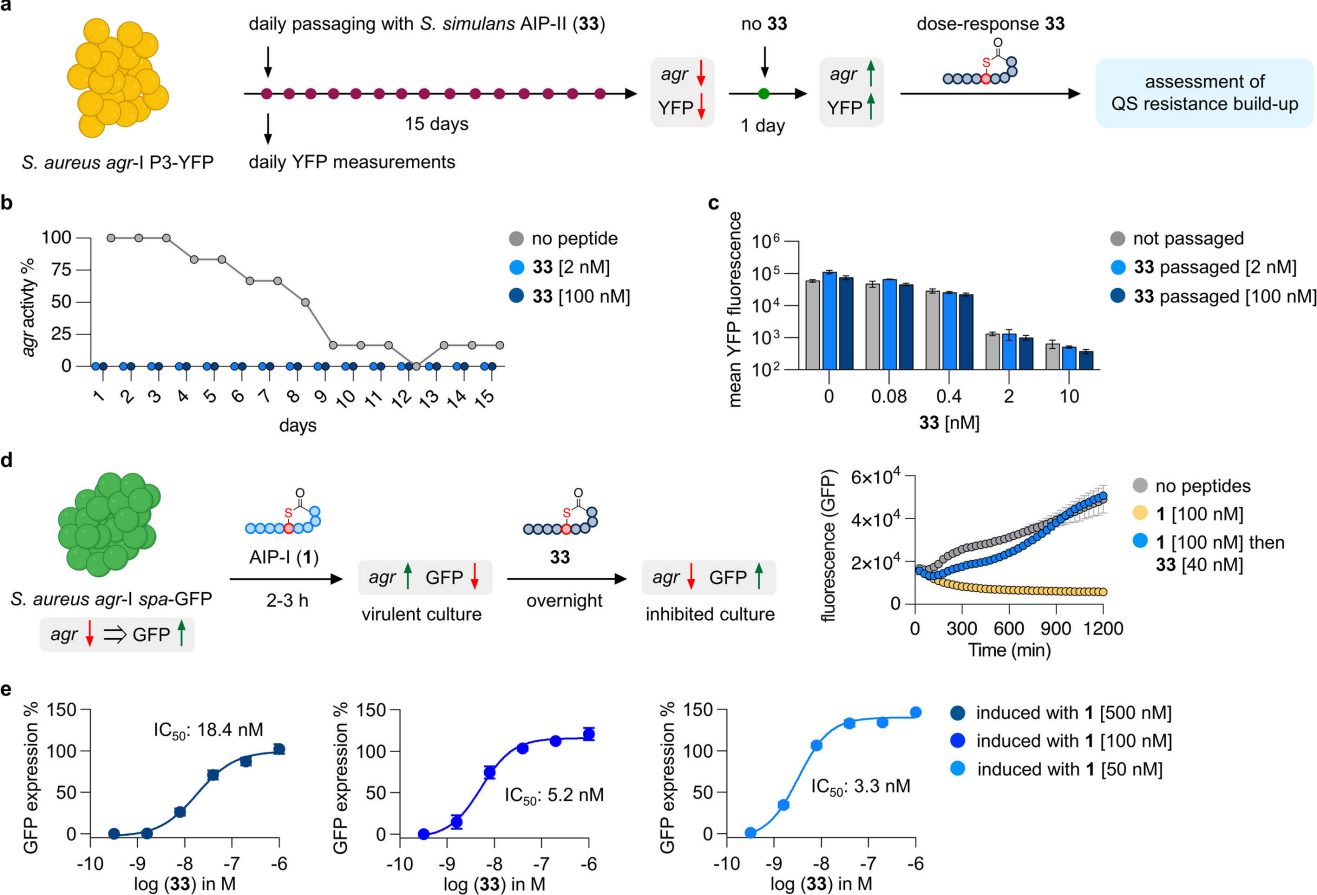
Quorum sensing resistance development and *agr* deactivation assays. (**a**) QS resistance development in a *S. aureus agr*-I P3-YFP reporter strain was examined by passaging cultures daily in the presence of *S. simulans* AIP-II (**33**) for 15 days. On day 16, the cultures were passaged without compound **33**, and dose-response curves against **33** were measured to assess changes in inhibitory potency. Fluorescence measurements were performed by flow cytometry. (**b**) Activity of *agr* over 15 days measured by flow cytometry. The *agr* activity of untreated cultures decreases during treatment while daily treatments with **33** repressed *agr* activity at 2 nM and 100 nM. (**c**) Passaged cultures were treated with **33** (10–0.08 nM) and remained equally susceptible to QS inhibition by **33** as not passaged cultures. (**d**) The *S. aureus agr*-I *spa*-GFP reporter strain (JE2, MRSA) was treated with *S. aureus* AIP-I (**1**)during early growth to activate *agr* and repress GFP expression. At the start of exponential growth, **33** was added, and GFP expression was monitored continuously. Treatment with **33** resulted in an immediate increase of fluorescence while induced cultures without **33** remained non-fluorescent. (**e**) IC_50_ values were determined for **33** against *S. aureus agr*-I *spa*-GFP induced with different concentrations of AIP **1**. GFP expression was plotted relative to non-induced cultures.

Anti-virulence treatments based on QS inhibition for *S. aureus*, in particular MRSA, have been postulated since the discovery of *agr* cross-inhibition and could become an important addition in fighting resistant infections, as it may attenuate their severity (50). However, despite a single recent clinical study with a commensal *S. hominis* strain (18), QS-based anti-virulence strategies require further development (51), and certain questions need to be answered: first, can *S. aureus* develop resistance toward QS inhibition, and second, how effective are QS inhibitors against already virulent bacteria. We attempted to address whether bacteria would develop resistance toward QS inhibitors, by assessing the effects of prolonged treatment of a fluorescent *S. aureus agr*-I P3-YFP reporter strain with *S. simulans* AIP-II (**33**) (Fig. 4a). With an estimated IC_50_ value of 0.45 nM against *S. aureus agr*-I (Fig. S26), we passaged the reporter strain daily for 15 days with and without the addition of compound **33** (either 2 nM or 100 nM), and the *agr* activity was measured daily by flow cytometry, showing full repression of *agr* activity at 2 nM dosing of compound **33** over the full period (Fig. 4b). After the 15 passages, all cultures were passaged once without the addition of compound **33**, followed by assessment of the sensitivity of the strains toward the inhibition of QS by compound **33**. Thus, both treatment groups were exposed to a dilution series of the inhibitory AIP, and no change in potency was observed compared to the non-passaged reporter strain (Fig. 4c). Despite the simplicity of this experiment, the results represent a first indicator that repeated treatments with AIP-based anti-virulence agents do not cause rapid resistance development *in vitro*.

Most QS inhibition data in the literature is performed by treating cultures with inhibitors before the *agr* system was activated, thereby measuring the prevention of *agr* activation. However, treatments would more often require *agr* deactivation of virulent bacteria. To better mimic a potential treatment scenario, we therefore transformed JE2, a highly virulent MRSA USA300 isolate of the *agr*-I type, with a *spa*-GFP reporter plasmid (52), creating a reporter strain that enables the measurement of *agr* deactivation. The *spa* gene is downregulated when *agr* is activated (53) and the reporter strain will therefore not become fluorescent when the QS system is active (Fig. 4d). We induced the *agr* system of reporter cultures with cognate AIP **1** at 100 nM upon inoculation in fresh medium, and once early exponential phase was reached, the inhibitor **33** (40 nM) was added, and GFP expression was monitored. Cultures induced with **1** remained non-fluorescent over the time of the assay, in contrast to cultures treated with **33**, which rapidly started to express GFP and reached fluorescence levels like uninduced cultures because of *agr* deactivation (Fig. 4d; Fig. S33). Next, we induced the *spa*-GFP reporter strain with different concentrations of **1,** followed by serial dilutions of **33**, affording IC_50_ values for deactivation of *agr* in the low nanomolar range, which increased when challenged by induction with higher concentrations of **1** (Fig. 4e; Fig. S33). In comparison, the IC_50_ value for prevention of *agr* activation of *S. aureus agr*-I by compound **33**, measured in the P3-YFP reporter assay (0.45 nM), is 40-fold lower than the highest measured IC_50_ value for *agr* deactivation (18.4 nM at induction with 0.5 μM of **1**). The combined observations of these two *in vitro* experiments highlight that QS-based anti-virulence agents are promising with respect to avoiding resistance development and can turn off a fully activated *agr* system in MRSA.

**Fig 5:**
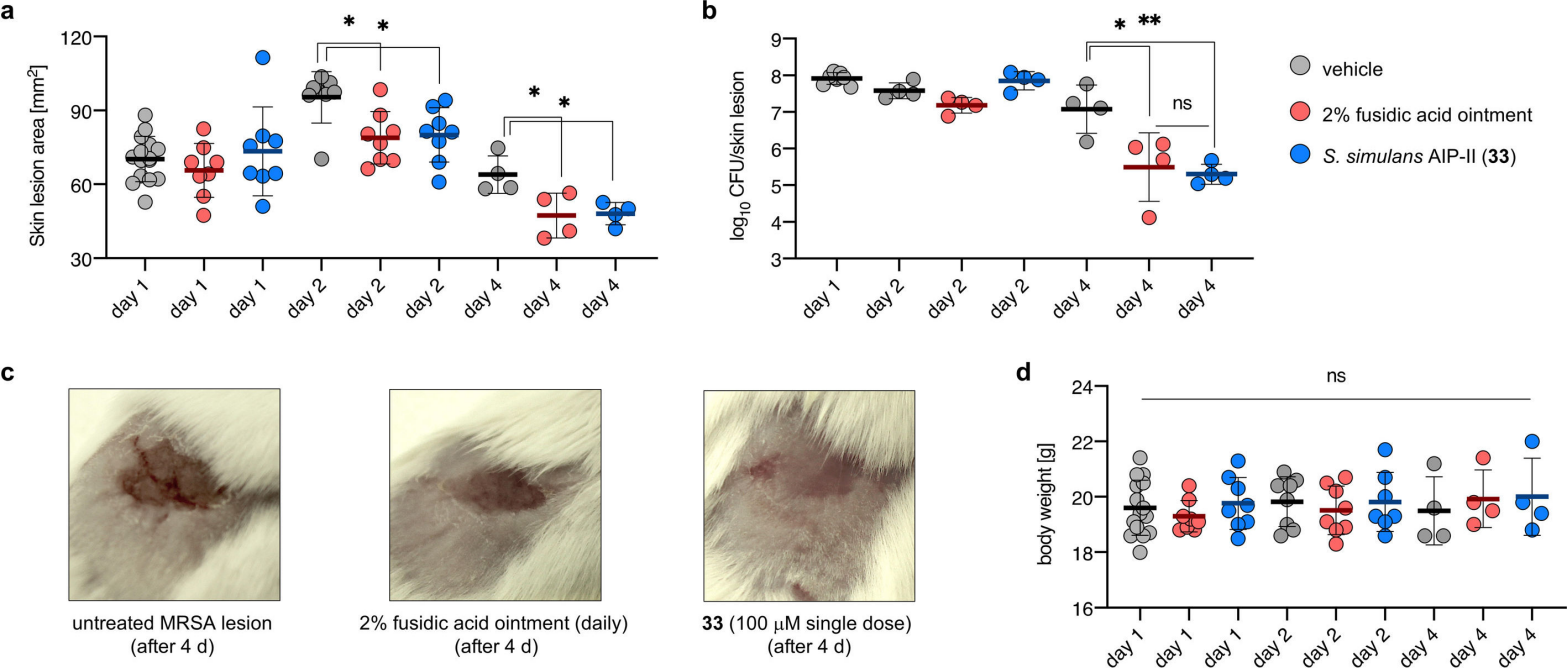
*S. simulans* AIP-II (**33**) attenuates MRSA infection in a murine skin model. (**a**) Murine skin infection model performed with vehicle control group [day 1 (*n* = 16), day 2 (*n* = 8), and day 4 (*n* = 4)], fusidic acid (daily application of a 38.7 mM ointment (Fucidin) [days 2 (*n* = 8) and 4 (*n* = 4)] and **33** (single treatment at day 0 at 100 μM [days 2 (*n* = 8) and 4 (*n* = 4)]. MRSA inoculum: 10^7^ CFU. Skin lesions measured on days 1, 2, and 4 showed a significant reduction in lesion size for mice treated with fusidic acid and **33**. (**b**) CFU count determined after days 1, 2, and 4 showing significant reduction in bacterial load per skin lesion. (**c**) Representative pictures taken of MRSA lesions at day 4 for untreated and treated mice. (**d**) Body weight measured on days 1, 2, and 4 showing no statistically significant differences. Data are presented as mean, and error bars represent the standard deviation (SD) of the mean. Statistical significance between treatment and vehicle groups was assessed by one-way analysis of variance (ANOVA) with multiple comparisons (Dunnett’s test), *P* > 0.05 (ns), *P* < 0.05 (*), *P* < 0.01 (**).

Finally, we assessed the potential of *S. simulans* AIP-II (**33**) as an anti-virulence agent in an *in vivo* MRSA (*agr*-I) mouse skin infection model (Fig. 5a through d; Table S5). The importance of a functioning *agr* system for *S. aureus* during infection to evade the immune response has been established, and it was shown that inhibition of *agr* during early stages of infection can lead to improved disease outcome 48–72 h after its initiation (50). Thus, *S. simulans* AIP-II (**33**) (100 μM) was added to the MRSA inoculum (10^7^ CFU) that was applied to the skin and compared with vehicle and daily treatment with the commercial antibacterial product Fucidin (2% fusidic acid). A significant reduction in the skin lesion size was observed after 48 h and 96 h for mice treated with **33** (*P* = 0.0174 and *P* = 0.0249) as well as fusidic acid (*P* = 0.0108 and *P* = 0.0202) compared with the vehicle control (Fig. 5a and c). Furthermore, a significant decrease in bacterial load (~60-fold, *P* = 0.009) was observed for mice treated with **33** compared with vehicle control after 4 days, which was comparable with daily treatment with fusidic acid (~38-fold, *P* = 0.0168) (Fig. 5b).

No statistical difference between treatment or vehicle was found for body weight over the course of infection, which was expected as no systemic infections were observed (Fig. 5d). These results are encouraging for the prospects of anti-virulence treatments of staphylococcal infections with non-antibiotic peptides, such as **33**. The high extent of bacterial clearance is most likely a result of a more efficient immune response toward non-virulent MRSA bacteria.

## DISCUSSION

Altering gene expression of co-inhabiting staphylococci through secreted AIPs represents an intriguing ability of the *agr* system. The *agr* loci can be found in the genomes of all staphylococcal species, and 37 AIPs from 21 species have now been identified from bacterial supernatants, pointing toward a broad utilization of the *agr* system, including potential QS interference. The colonization of humans and animals by a wide range of staphylococcal species emphasizes that many species share the same environments, providing an arena for biologically relevant inter-staphylococcal interactions, through AIP-mediated communication. Here, we report the most comprehensive mapping of QS interference by staphylococcal AIPs performed to date, including six newly identified AIPs together with all previously reported AIPs. Testing this collection of AIPs against fluorescent reporter strains of *S. aureus agr*-I–IV, *S. epidermidis agr*-I–III, and *S. lugdunensis agr*-I provided a map of 280 QS interactions, which revealed a largely cross-inhibitory network and led to the discovery of several potent inhibitors against all the eight tested *agr* systems as well as previously unknown cross-species activators. This comparative data set will help uncover the ecological importance of potential interactions between staphylococci in their natural niches, such as the skin microbiome. Metagenomic analyses have indicated that the human skin microbiome is relatively stable despite exposure to the external environment (54), but the severity of skin disorders like atopic dermatitis has been correlated with a lower abundance of commensal *S. hominis* (55). Although *S. hominis* is known to produce bacteriocins that kill *S. aureus* (56), it also plays a protective role in the skin microbiome of atopic dermatitis patients by interfering with *S. aureus* QS (12). A strength of the current study is the delineation of cause-and-effect by testing synthetic AIPs. This requires knowledge of the specific AIP produced by the bacterium, which cannot be inferred from the AgrD sequence (11). Previous works have indirectly studied the effect of AIPs in reporter strain assays using cell-free supernatants (3, 9, 10, 13, 15). This fast screening approach does not require AIP identification and chemical synthesis. However, other factors than AIPs can result in changes to QS, and promising inhibitors can be overlooked due to sub-inhibitory concentrations, as exemplified in a recent study where the supernatant from an *S. simulans agr*-II type isolate was unable to inhibit *agr* of *S. aureus* (13). Furthermore, real ecology is influenced by environmental conditions, host factors, and other microbes and their metabolites—complicating our understanding of QS interference beyond what can be learned from reductionist systems (1, 57).

Nevertheless, during our screening, several cross-activators were revealed. Among them was *S. hominis* AIP-I (**10**), which activates *S. epidermidis agr*-I, albeit only observed at the highest concentration tested (1 μM). This interaction could be relevant to human skin as it would occur between the two most abundant staphylococci (15, 58). However, it is not clear whether this interaction is beneficial to the host. On one hand, the *agr* system of *S. epidermidis agr*-I positively regulates a cysteine protease, Ecp, that was important for skin colonization in a porcine model (45). On the other hand, a different study found a correlation between increased severity of atopic dermatitis and elevated levels of *S. epidermidis*-derived *ecp* transcripts (59). Thus, further investigation of the potential importance of this cross-activation on human skin would be highly interesting.

Although cross-activation was generally sparsely represented in the QS interaction map, it is noteworthy that AIP activities that were characterized as “mixed interference” could also be considered partial activators. A recent report possibly explains our finding that certain AIPs were able to fully inhibit an *agr* system at low doses, whereas only partial inhibition was observed at higher concentrations. The proposed mechanism relies on the capacity of the AgrC dimer to bind two ligands. Thus, at the lower concentrations, only one binding site is occupied by the non-native AIP, whereas the native AIP is bound to the other site, resulting in full inhibition. In contrast, at higher concentrations, both binding sites are occupied by non-native AIP, resulting in partial agonism (35).

To further scrutinize the requirements for the potent inhibition and activation profiles of the *S. simulans* AIPs, a structure-activity relationship study was performed based on *S. simulans* AIP-II (**33**) and *S. simulans* AIP-III (**34**), including the evaluation of alanine mutants and truncated peptides. The results of this exercise highlighted the importance of different structural features of the peptides. Truncations of the exo-tail affected the ability to inhibit *S. aureus* the most, whereas the macrocycle alone was enough to effectively inhibit *S. lugdunensis*. The *S. simulans* AIPs represent the first potent inhibitors of QS in *S. lugdunensis* with a 400-fold increase in potency compared with previously reported inhibitors (37). Through substitution to alanine in each position of the best multi-group inhibitor, *S. simulans* AIP-II (**33**), we found that aside from the K1A substitution (**36**), which had minor effects on the overall inhibition profile, alanine substitution at all other positions had marked effects on the original profile. We encourage building on this SAR study of *S. simulans* AIP-II and AIP-III, possibly through peptide structure determination by NMR, coupled with structure predictions of the AgrC receptors to further understand the antagonistic interactions.

Furthermore, *S. simulans* AIP-II (**33**) was investigated for its potential as an anti-virulence agent, by assessing whether MRSA would develop resistance toward the inhibitor upon dosing over 2 weeks and assessing whether *agr* could be deactivated in a pathogenic MRSA strain. The fluorescent *agr* reporter strain used for this *in vitro* experiment still produced fluorescence, that is, had a functional *agr* system, after 15 days of passaging in the presence of **33**. No difference in susceptibility to dose-dependent QS inhibition was observed between the non-passaged reporter and the passaged strain. To the best of our knowledge, this represents the first assessment of resistance development in *S. aureus* in response to QS inhibition via AgrC. Although we observed no indication of resistance development, AIP treatment in a more complex infection environment could lead to different outcomes. However, no resistance development was observed in response to *in vitro* or *in vivo* treatment of *S. aureus* with the AgrA inhibitor, savirin (60).

Additionally, through the construction of an inverse fluorescent *agr* reporter strain, we demonstrated that **33** could shut down the fully active *agr* system of an MRSA isolate. Finally, a significant effect of this inhibitory AIP on the colonization and pathogenesis of MRSA *in viv*o was demonstrated in a mouse model, highlighting the power of gene repression through QS inhibition (10, 12, 13, 18). Similar experiments have shown how inhibitory AIPs can reduce disease measures to levels comparable with *agr* mutants (15). Remarkably, we achieved significant improvement in outcome with an approximately 10-fold lower dose of **33** compared with the lowest dosages of other inhibitory AIPs previously tested in similar murine epi-cutaneous infection models (12, 13, 15). A limitation of these experiments is that inhibitory AIPs were always administered together with the bacterial inoculum. Based on our *in vitro* data, we could inactivate an already active *agr* system using **33**. Thus, further *in vivo* testing should focus on delayed administration to provide stronger evidence for the use of AIP-based anti-virulence agents and assess the severity of potential tissue damage caused by already secreted toxins and proteases prior to treatment.

Our results highlight the potential importance of the *agr* system and cross-species interference on the colonization of commensal staphylococci and the pathogenesis of, for example, *S. aureus*. However, the impact of the substantial QS interference among commensal staphylococci on the human microbiota remains to be explored further.

It is our hope that the mapping of cross-species QS interactions initiated in the present work will help provide insights into the roles of *agr* systems in future investigations. Furthermore, our findings highlight the potential utility of natural scaffolds as a promising platform for the development of inhibitors for anti-virulence treatment of *Staphylococcus* infections.

## MATERIALS AND METHODS

### Preparation of resins for native chemical ligation trapping

Amino PEGA resin (1.00 g, loading: 0.42 mmol/g, 0.42 mmol) was placed in a polypropylene syringe equipped with a fritted disk, swelled in DMF for 15 min and washed with DMF (5 × 1 min). Fmoc-Rink-amide linker (1.13 g, 2.10 mmol, 5.0 equiv), HATU (782 mg, 2.06 mmol, 4.9 equiv), and *i-*Pr_2_NEt (736 μL, 4.20 mmol, 10.0 equiv) were pre-incubated in DMF (10.0 mL) for 2 min and then added to the resin. After 2 h, the resin was washed with DMF (3 × 1 min), MeOH (3 × 1 min), and CH_2_Cl_2_ (3 × 1 min) and treated with a capping solution (Ac_2_O–*i-*Pr_2_NEt–CH_2_Cl_2_, 2:2:6, vol/vol/vol, 10.0 mL). After 2 h, the resin was washed with DMF (3 × 1 min), MeOH (3 × 1 min), and CH_2_Cl_2_ (3 × 1 min). The resin was then treated with piperidine in DMF (1:4, vol/vol, 10.0 mL) (1 × 2 min, 1 × 20 min) and washed with DMF (3 × 1 min), MeOH (3 × 1 min), and CH_2_Cl_2_ (3 × 1 min). Fmoc-Cys(S*t*-Bu)-OH (272 mg, 0.63 mmol, 1.5 equiv) or Fmoc-Cys(SIT)-OH (61) (335 mg, 0.63 mmol, 1.5 equiv), HATU (240 mg, 0.63 mmol, 1.5 equiv), and *i-*Pr_2_NEt (220 μL, 1.26 mmol, 3.0 equiv) were pre-incubated in DMF (10.0 mL) for 2 min and then added to the resin. After 2 h, the resin was washed with DMF (3 × 1 min), MeOH (3 × 1 min), and CH_2_Cl_2_ (3 × 1 min) and dried under a high vacuum for 16 h.

### Native chemical ligation trapping of AIPs from bacterial supernatants

Bacterial isolates were streaked on agar plates and grown overnight at 37°C. Single colonies were then inoculated in 50 mL TSB media overnight at 37°C in an incubator at 200 rpm shaking. Overnight cultures were centrifuged at 8,000 rpm at 4°C, and supernatants were filtered through a sterile filter (0.22 μm) and stored at 4°C for direct use or frozen and stored at −20°C until use. For resin preparation, Fmoc-Cys(S*t*-Bu)-Rink-PEGA resin (50 mg) or Fmoc-Cys(SIT)-Rink-PEGA resin (50 mg) was placed in a 2.0 mL polypropylene syringe equipped with a fritted disk, swelled in DMF for 15 min and washed with DMF (5 × 1 min). The resin was treated with piperidine in DMF (1:4, vol/vol, 2.0 mL) (1 × 2 min, 1 × 20 min) and washed with DMF (5 × 1 min). The resin was then treated with a solution of β-mercaptoethanol in DMF (1:4, vol/vol, 2.0 mL) containing *N*-methyl morpholine (NMM) (0.1 M) or DL-dithiothreitol (DTT) in DMF (0.05:0.95, wt/vol, 2.0 mL) containing NMM (0.1 M) (3 × 10 min) and subsequently washed with DMF (3 × 1 min), MeOH (3 × 1 min), and H_2_O (3 × 1 min). For NCL trapping, the sterile and filtered bacterial supernatant (~50 mL) was added to a 50 mL centrifugal tube, and the pH was adjusted to pH = ~7.0 using aqueous NaOH (1.0 M). An aqueous tris(2-carboxyethyl)phosphine hydrochloride (TCEP) solution (1.0 mL, 0.5 M, pH = 7.0; final conc. = 10.0 mM) was added to the supernatant followed by the deprotected Cys-Rink-PEGA-resin, and the centrifugal tube containing the trapping mixture was agitated at 37°C overnight. The next day, the resin was separated from the supernatant through filtration using a 10 mL polypropylene syringe equipped with a fritted disk under suction and washed with DMF (3 × 1 min), H_2_O (3 × 1 min), and DMF (3 × 1 min). A solution of DTT in DMF (0.05:0.95, wt/vol, 2.0 mL) containing NMM (0.1 M) was added to the resin, and the resin was agitated at 37°C. After 30 min, the resin was washed with DMF (3 × 1 min), MeOH (3 × 1 min), and CH_2_Cl_2_ (3 × 1 min) and dried under suction for 15 min. The dried resin was treated with a cleavage cocktail (2.0 mL, TFA–MilliQ water, 97:3, vol/vol) for 2 h at room temperature. The peptide-containing cleavage solution was removed from the resin and collected, and the resin was rinsed with neat TFA (1.0 mL). The combined TFA fractions were evaporated under the N_2_ stream to near dryness, redissolved in a solution of MeCN in H_2_O (100 μL, 1:1, vol/vol) and filtered (0.22 μm). For LC-MS analysis, the filtered TFA cleavage solution was analyzed using a Waters Acquity system equipped with a Phenomenex Kinetex column (1.7 μm, 100 Å, 50 × 2.10 mm) applying a gradient with eluent C (0.1% HCOOH in water) and eluent D (0.1% HCOOH in MeCN) rising linearly from 0 to 50% of D over 10.0 min at a flow rate of 0.6 mL min^−1^ and an injection volume of 40 μL. The total ion chromatograms (TIC) were analyzed by displaying extracted ion chromatograms (EIC) of *m/z* [M + H]^+^ values of the possible linear peptides with an additional *C*-terminal cysteine and amide functionality based on the AgrD sequence.

### Fluorescence reporter assay for screening *agr*-interference of peptides

Peptides were evaluated for the ability to interfere with *agr-*mediated quorum sensing in *S. aureus* (AH1677, AH430, AH1747, and AH1872 for *agr*-I–IV, respectively) (40) using reporter strains expressing yellow fluorescent protein (YFP) upon *agr* activation. Interference with *agr-*mediated quorum sensing in *S. epidermidis* (AH3408, AH3623, and AH3409 for *agr*-I–III, respectively) (45), and *S. lugdunensis* (AH4031 for *agr*-I) (37) was evaluated using reporter strains expressing superfolder green fluorescent protein (sGFP) upon *agr* activation. Overnight cultures of the reporter strains were grown in TSB medium containing chloramphenicol (CAM, 10 μg/mL for *S. aureus*) or erythromycin (ERM, 10 μg/mL of *S. epidermidis* and *S. lugdunensis*) and diluted 1:100 in fresh TSB medium containing the same antibiotic. Assays were performed in sterile black 96-well plates with a clear bottom. All peptides were screened at concentrations of 1.0 μM and 50 nM and peptides showing at least 75% inhibition at 50 nM were screened further at 2.5 nM against the respective reporter strain, and similarly at 0.125 nM in the case of at least 75% inhibition at 2.5 nM. DMSO stock solutions of peptides (1 mM) were diluted in TSB media and added (15 μL) in technical triplicate to the 96-well plate, followed by diluted bacterial overnight cultures (135 μL). Control wells for 100% *agr* activity were wells replacing the peptide solution with TSB medium (15 μL). Control wells did not contain DMSO, but the potential effect of DMSO on *agr* activity was indirectly tested, since examples against each of the eight reporter strains demonstrated that certain peptides had no effect at the highest concentration (0.1% DMSO, vol/vol). Wells containing 150 μL TSB medium were used to measure background fluorescence. The 96-well plates were incubated in a humidified incubator at 37°C shaking at 1,000 rpm for 22–24 h and fluorescence (for GFP: excitation 479 nm, emission 520 nm; for YFP: excitation 500 nm, emission 541 nm; automatic gain), and OD_600_ values were subsequently measured using a plate reader. Background fluorescence was subtracted from all wells and further normalized to the corresponding OD_600_ value of the respective wells. The average fluorescence of control wells was used as a relative measure for 100% activation of the *agr*-circuit, and bar graphs were generated using GraphPad Prism 10.4 software. All assays were performed in at least a biological duplicate.

### Fluorescence reporter assay for IC_50_ determination

Overnight cultures of the reporter strains were grown in TSB medium containing chloramphenicol (CAM, 10 μg/mL for *S. aureus*) or erythromycin (ERM, 10 μg/mL of *S. epidermidis* and *S. lugdunensis*) and diluted 1:100 in fresh TSB medium containing the same antibiotic. Assays were performed in sterile black 96-well plates with clear bottoms. Peptide solutions (15 μL) in 1:5 serial dilutions from DMSO stock solutions (1 mM) in TSB medium were added to the 96-well plate in technical duplicate followed by diluted bacterial overnight cultures (135 μL). Control wells for 100% *agr* activity were wells replacing the peptide solution with TSB medium (15 μL). Wells containing 150 μL TSB medium were used to measure background fluorescence. The 96-well plates were incubated in a humidified incubator at 37°C shaking at 1,000 rpm for 22–24 h and fluorescence (for GFP: excitation 479 nm, emission 520 nm; for YFP: excitation 500 nm, emission 541 nm; automatic gain), and OD_600_ values were subsequently measured using a plate reader. Background fluorescence was subtracted from all wells and further normalized to the corresponding OD_600_ value of the respective wells. The average fluorescence of control wells was used as a relative measure for 100% activation of the *agr*-circuit. Relative *agr* activity was plotted to obtain IC_50_ values by non-linear regression with variable slope using GraphPad Prism 10.4 software. All assays were performed in biological triplicate.

### β-Lactamase assay for IC_50_ determination against *S. aureus agr*-I–IV

The β-lactamase reporter strain RN10829 (P2-*agrA*:P3-*blaZ*) (62), with p*agrC-*I (63), p*agrC-*II, p*agrC-*III, or p*agrC-*IV (11), substituting the native *agr* locus with a chromosomal integration of P2-*agrA* and P3-*blaZ* and a plasmid from which a wild-type variant of the corresponding AgrC is expressed, was used to assess inhibition and activation of the AgrC receptor via β-lactamase activity in response to varying concentrations of the QS modulating peptides. Overnight cultures of the reporter strains in TSB medium were diluted 1:250 in fresh TSB medium and grown to OD_600_ = 0.35–0.40 (early exponential phase) at 37°C. Peptide solutions (10 μL) in 1:10 serial dilutions from DMSO stock solutions (1 mM) in TSB medium (final concentrations = 10 μM–10 pM) were added to each well of a clear 96-well plate as well as solutions (10 μL) of cognate AIP (final concentration = 100 nM) in TSB medium followed by 80 μL of bacterial cells. Control wells for 100% β-lactamase activity were wells replacing peptide solution with TSB media (10 μL), and control wells for 0% β-lactamase activity were wells replacing both peptide and cognate AIP solutions with TSB medium (20 μL). The 96-well plates were incubated at 37°C shaking at 200 rpm for 1 h and immediately frozen down at −80°C to minimize growth during nitrocefin treatment. Next, the 96-well plates were thawed, and OD_600_ values were determined using a plate reader followed by the addition of 50 μL of nitrocefin solution to the wells (final concentration = 33.3 μg/mL). β-Lactamase activity was monitored at OD_486_ every 20 s for 10 min at 37°C using a plate-reader. Linear nitrocefin conversion rates were plotted to obtain IC_50_ values by non-linear regression with variable slope using GraphPad Prism 10.4 software. Assays were performed at least as duplicate determinations in biological triplicate.

### Fluorescence reporter assay for *agr* deactivation of virulent *S. aureus*

A fluorescent reporter strain of *S. aureus* JE2 containing a plasmid with a *spa::GFP* promoter was constructed by transforming the strain with pALC1741 (52). Cultures of the reporter strain were grown from single colonies in TSB medium containing chloramphenicol (CAM, 10 μg/mL) and *S. aureus* AIP-I (**1**) (concentrations = 500 nM, 100 nM, or 50 nM) to OD_600_ = 0.2–0.3 (early exponential phase) at 37°C. Assays were performed in sterile black 96-well plates with clear bottoms. Peptide solutions (15 μL) in 1:5 serial dilutions from DMSO stock solutions (1 mM) in TSB medium were added to the 96-well plate in technical triplicate followed by the induced bacterial cultures (135 μL). Control wells were uninduced cultures containing no *S. aureus* AIP-I (**1**). The 96-well plates were incubated in a plate reader at 37°C shaking at 283 rpm and fluorescence (GFP: excitation 479 nm, emission 520 nm, automatic gain), and OD_600_ values were continuously measured. For IC_50_ values, the final fluorescence measurements were normalized to the corresponding OD_600_ value of the respective wells, and the average fluorescence of control wells was used as a relative measure for 100% GFP expression. Relative GFP expression was plotted to obtain IC_50_ values by non-linear regression with variable slope using GraphPad Prism 10.4 software. All assays were performed in biological triplicate.

### Quorum sensing inhibition resistance development experiment

Bacterial cultures (*n* = 3 per treatment) of a fluorescent reporter strain of *S. aureus* (AH1677) with a *P3::YFP* promoter plasmid were cultured in 15 mL centrifugal tubes in 2.0 mL of TSB medium containing chloramphenicol (CAM, 10 μg/mL) and *S. simulans* AIP-II (**33**) (concentrations = 100 nM, or 2.0 nM) or no peptide overnight at 37°C in a shaking incubator. The next day, YFP expression levels were assessed by flow cytometry (YFP: excitation 500 nm, emission 541 nm), and the overnight cultures were diluted 1:250 in fresh TSB medium containing CAM and **33** or no peptide (final volume 2.0 mL) and incubated overnight at 37°C in a shaking incubator. The procedure was repeated in total for 15 days, and the passaged cultures were subsequently frozen at −80°C. The passaged cultures were inoculated from frozen stocks in 2.0 mL TSB medium containing CAM in 15 mL centrifugal tubes and incubated overnight at 37°C in a shaking incubator. Overnight cultures were diluted 1:250 in fresh TSB medium containing CAM, and 1:5 serial dilutions of *S. simulans* AIP-II (**33**) (concentrations = 10 nM–0.08 nM) were added; the cultures were incubated overnight at 37°C in a shaking incubator. The next day, YFP expression levels were assessed by flow cytometry (YFP: excitation 500 nm, emission 541 nm) to determine the susceptibility toward *agr* inhibition by *S. simulans* AIP-II (**33**).

### *In vivo* MRSA skin infection model

The mouse model was performed under contract at Statens Serum Institut (DK) essentially as previously described (64). In brief, eight to 10-week-old Balb/c female mice (Taconic Denmark) were used for all experiments [*n* = 16 for vehicle, *n* = 8 for Fucidin (2% fusidic acid ointment) treatment, and *n* = 8 for *S. simulans* AIP-II (**33**) treatment]. All animal experiments were approved by the National Committee of Animal Ethics, Denmark. Mice were anesthetized, and the hair was removed on a 2 cm^2^ skin area on the back, and thereafter, the outermost layer of the skin was scraped off with a dermal curette to obtain a 1 cm^2^ superficial skin lesion. For vehicle control and Fucidin treatment, 10 μL inoculum containing approximately 10^7^ CFU of methicillin resistant *S. aureus* (MRSA43484) were spread on the skin lesions. For *S. simulans* AIP-II (**33**) treatment, 10 μL of the same inoculum containing **33** (100 μM prepared from a 10 mM DMSO stock solution of **33·**HCl immediately before application) were spread on the skin lesions. This leads to the absolute application of 1 nmol, corresponding to ~10-fold less inhibitory AIP than previously tested in other murine epicutaneous infection models (12, 13, 15). After the inoculum had dried, the mice were placed in a cage and kept in a warming cabinet until fully awake. The topical skin treatment with Fucidin was initiated one day after inoculation (days 2, 3, and 4) by spreading 50 μL of Fucidin on the inoculated skin area once a day. Skin lesion size and body weight were measured on days 1, 2, and 4 of all mice. Mice were sacrificed on days 1 (*n* = 8 vehicle), 2 (*n* = 4 vehicle, Fucidin, **33**), and 4 (*n* = 4 vehicle, Fucidin, **33**), and the infected skin area was cut out and homogenized to determine the CFU count in the skin lesions. Statistical testing was conducted using one-way analysis of variance (ANOVA) with multiple comparisons (Dunnett’s test) and an alpha level of 0.05.

### Associated content

The supplemental material contains supplemental figures illustrating AIP trapping experiments, dose-response curves, and bar graphs for tested compounds, supplemental schemes showing the compounds syntheses, and supplemental tables containing assay data and library compound sequences. Peptide synthesis procedures and compound characterization data are provided as well as copies of HPLC chromatograms and copies of ^1^H and ^13^C NMR spectra.

## SUPPLEMENTAL MATERIAL

Supplemental Characterization mbio.00967-25-s0001.pdf NMR spectra and chromatograms.

Supplemental Material mbio.00967-25-s0002.pdf Additional figures, schemes, and tables as well as compound characterization data.

ASM does not own the copyrights to Supplemental Material that may be linked to, or accessed through, an article. The authors have granted ASM a non-exclusive, world-wide license to publish the Supplemental Material files. Please contact the corresponding author directly for reuse.
