# Supplementary material for: Mapping of quorum sensing interaction network of commensal and pathogenic staphylococci
Source: mBio. 2025 Jul 16;16(8):e00967-25. doi: 10.1128/mbio.00967-25 (PMC12345137; doi:10.1128/mbio.00967-25)

UPLC traces of synthetic peptides

S. epidermidis AIP-I (**5**)

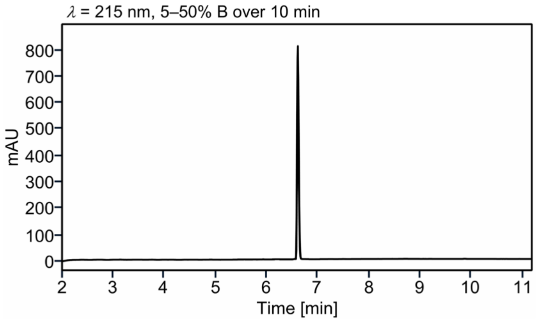

S. epidermidis AIP-II (**6**)

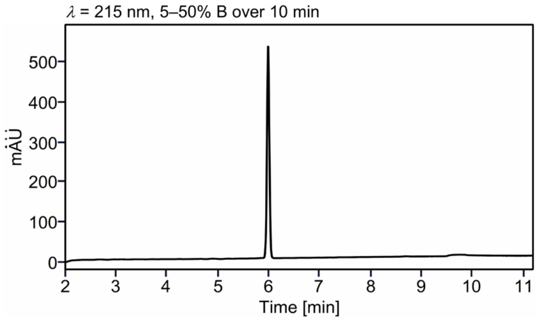

S. epidermidis AIP-III (**7**)

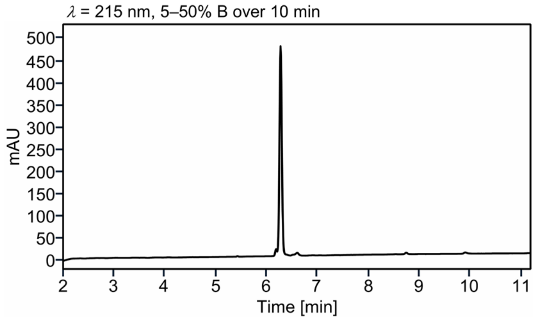

S. lugdunensis AIP-I (**8**)

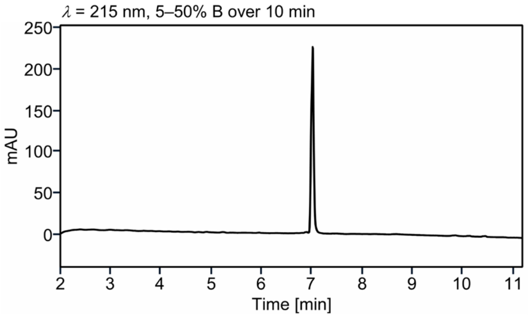

S. hominis AIP-I (**10**)

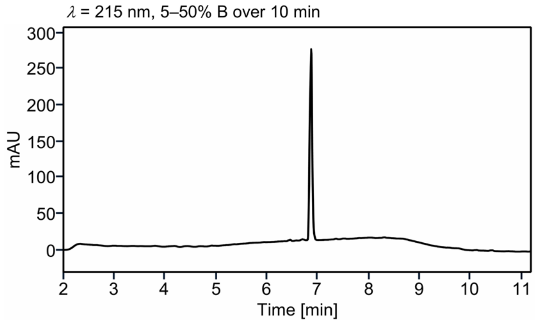

S. hominis AIP-II (**11**)

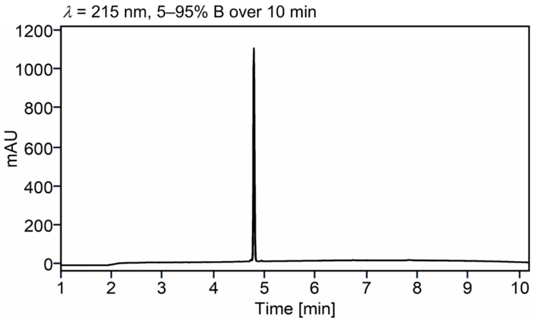

S. hominis AIP-IV (**13**)

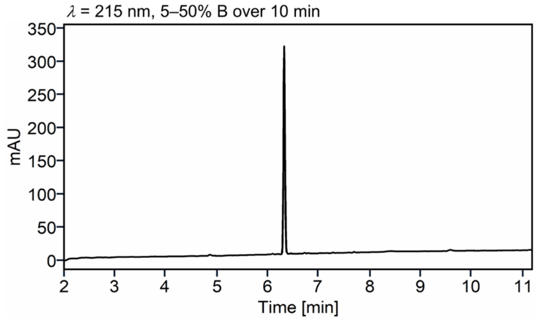

S. hominis AIP-V (**14**)

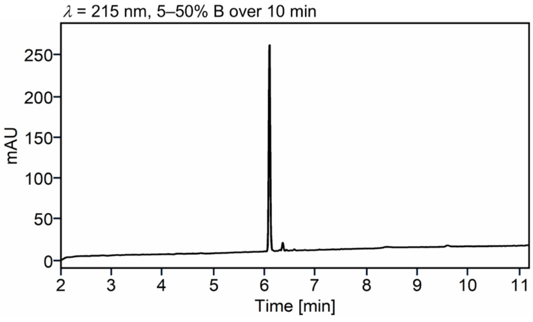

S. warneri AIP-II (**17**)

λ = 215 nm, 5–50% B over 10 min

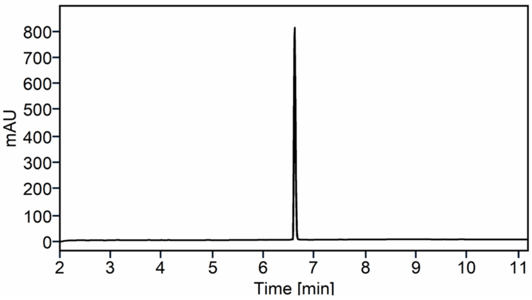

S. cohnii AIP-I (**18**)

λ = 215 nm, 5–50% B over 10 min

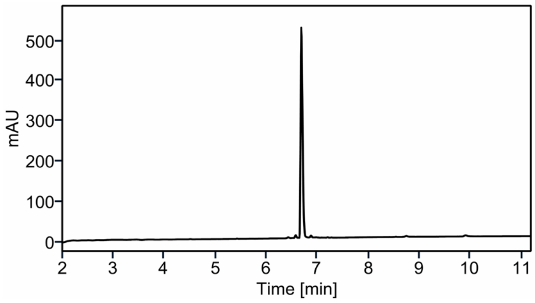

S. caprae AIP-I (**20**)

λ = 215 nm, 5–50% B over 10 min

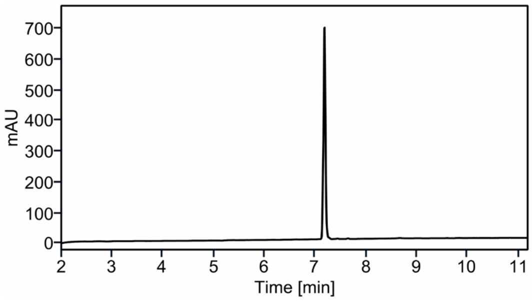

S. pasteuri AIP-I (**21**)

λ = 215 nm, 5–50% B over 10 min

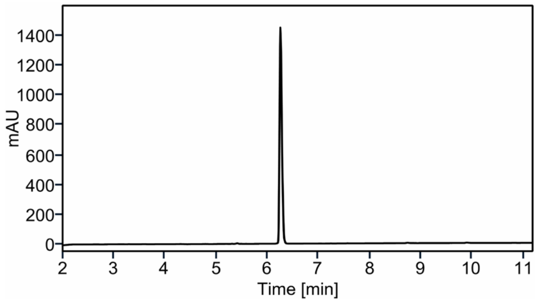

S. devriesei AIP-I (**22**)

λ = 215 nm, 5–50% B over 10 min

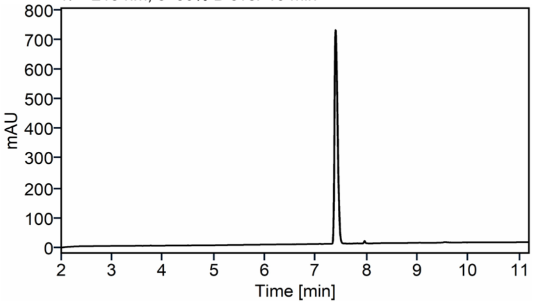

S. succinus AIP-I (**23**)

λ = 215 nm, 5–50% B over 10 min

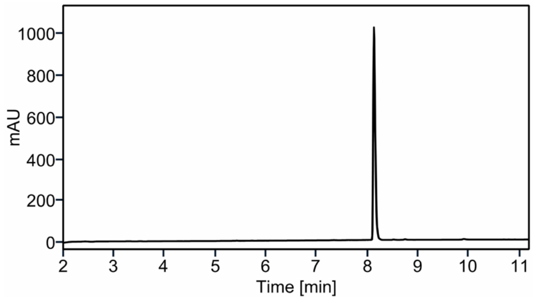

S. equorum AIP-I (**24**)

λ = 215 nm, 5–50% B over 10 min

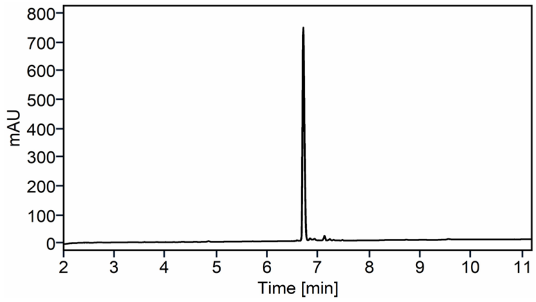

S. equorum AIP-II (**25**)

λ = 215 nm, 5–50% B over 10 min

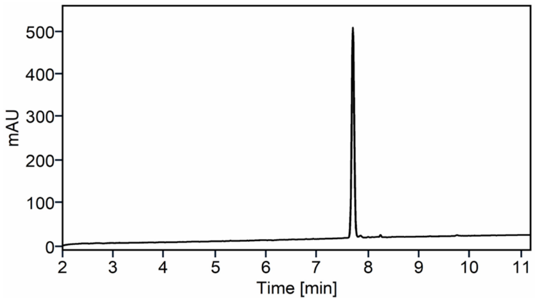

S. intermedius AIP-I (**31**)

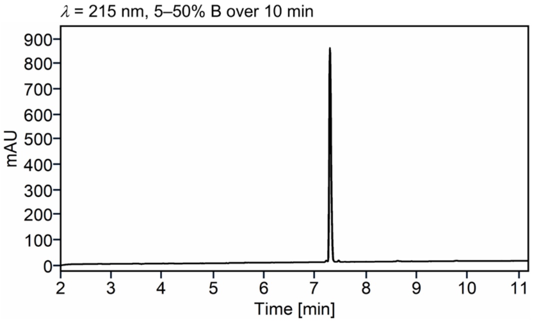

S. simulans AIP-II (**33**)

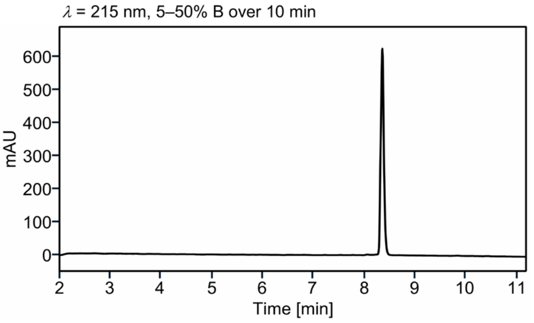

S. simulans AIP-III (**34**)

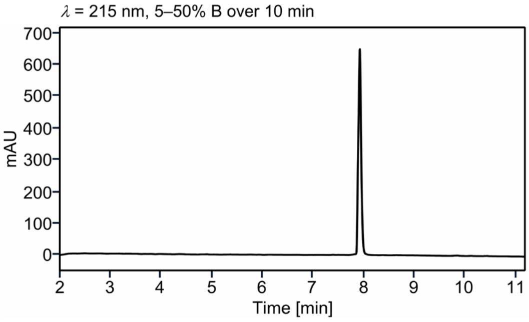

S. simulans AIP-II K1A (**36**)

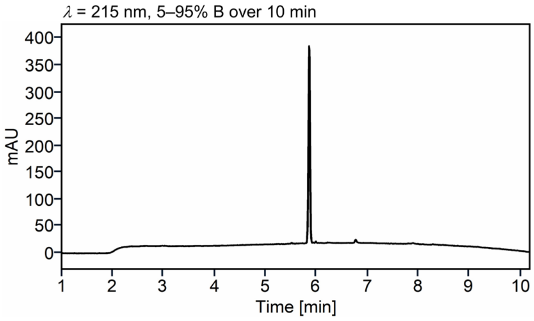

S. simulans AIP-II Y2A (**37**)

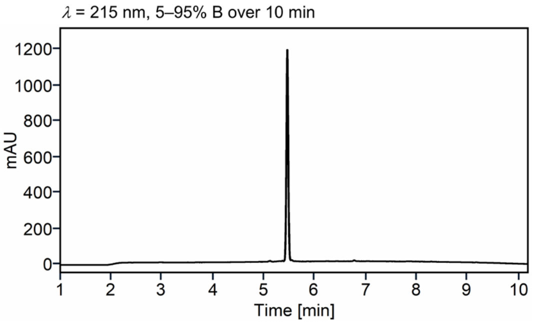

S. simulans AIP-II/III Y3A/N3A (**38**)

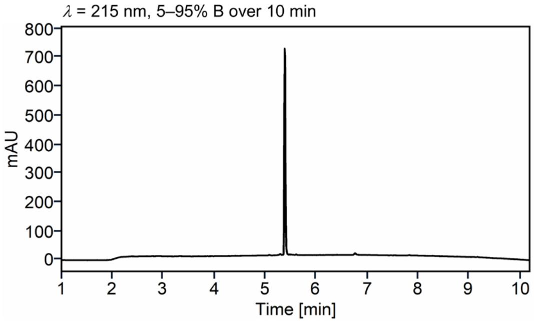

S. simulans AIP-II P4A (39)

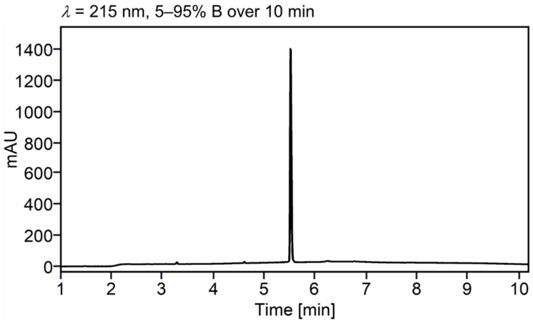

S. simulans AIP-II W6A (**40**)

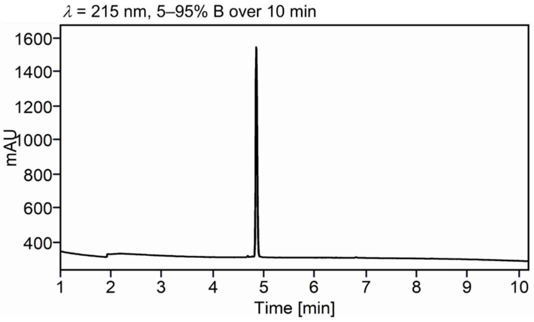

S. simulans AIP-II G7A (**41**)

λ = 215 nm, 5–95% B over 10 min

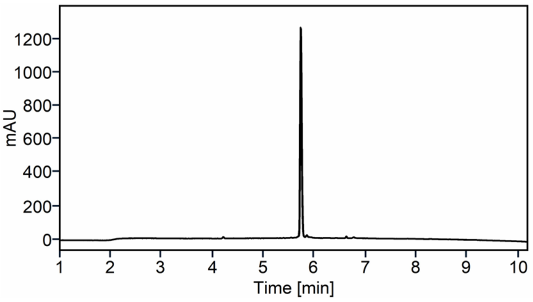

S. simulans AIP-II Y8A (**42**)

λ = 215 nm, 5–95% B over 10 min

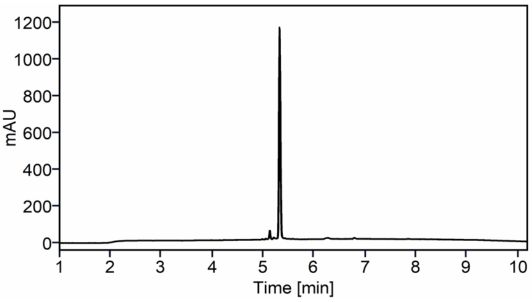

S. simulans AIP-II F9A (**43**)

λ = 215 nm, 5–95% B over 10 min

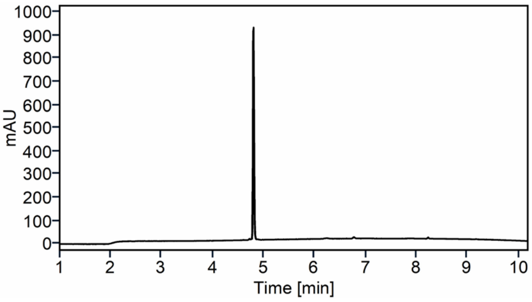

S. simulans AIP-II lactam (**44**)

λ = 215 nm, 5–95% B over 10 min

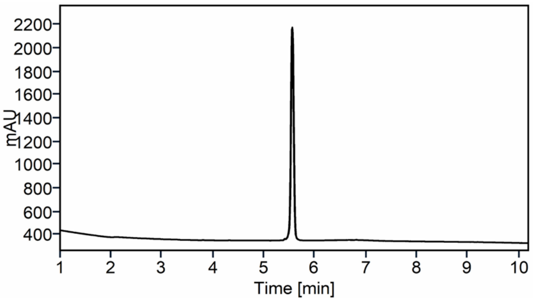

S. simulans AIP-III K1A (**45**)

λ = 215 nm, 5–95% B over 10 min

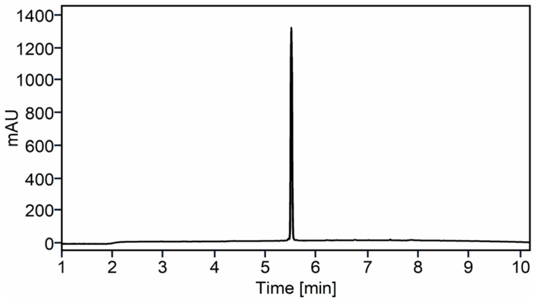

S. simulans AIP-III Y2A (**46**)

λ = 215 nm, 5–95% B over 10 min

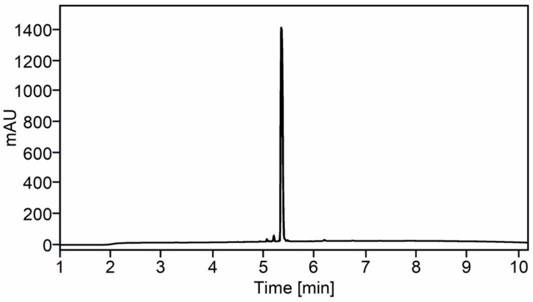

S. simulans AIP-III P4A (**47**)

λ = 215 nm, 5–95% B over 10 min

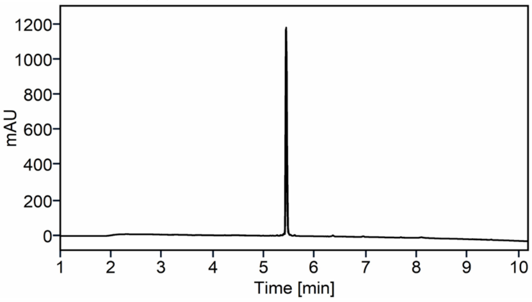

S. simulans AIP-III W6A (**48**)

λ = 215 nm, 5–95% B over 10 min

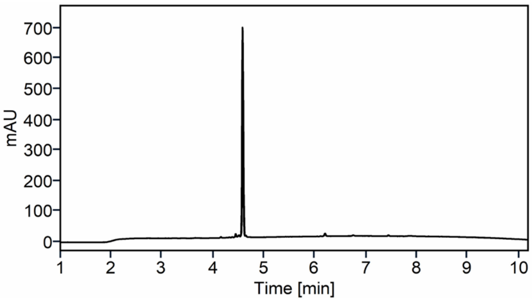

S. simulans AIP-III G7A (**49**)

λ = 215 nm, 5–95% B over 10 min

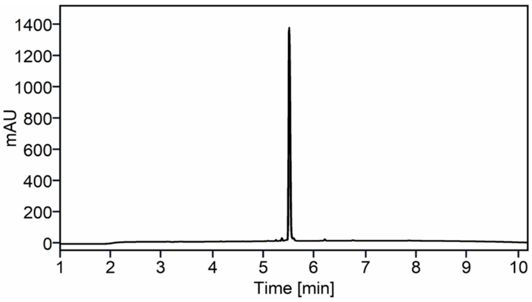

S. simulans AIP-III Y8A (**50**)

λ = 215 nm, 5–95% B over 10 min

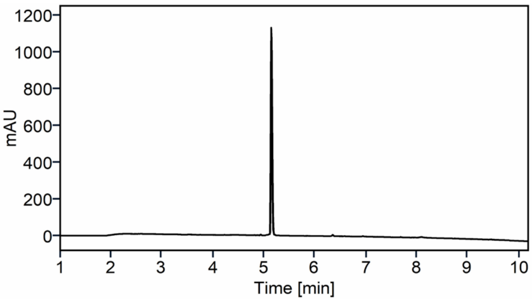

S. simulans AIP-III F9A (**51**)

λ = 215 nm, 5–95% B over 10 min

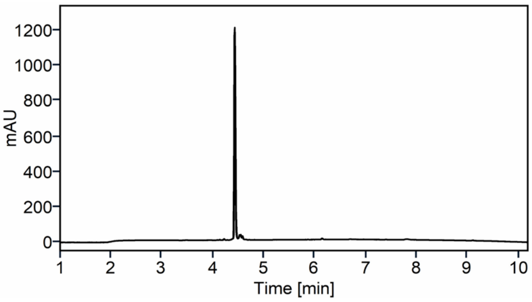

S. simulans AIP-III 8-mer (**52**)

λ = 215 nm, 5–95% B over 10 min

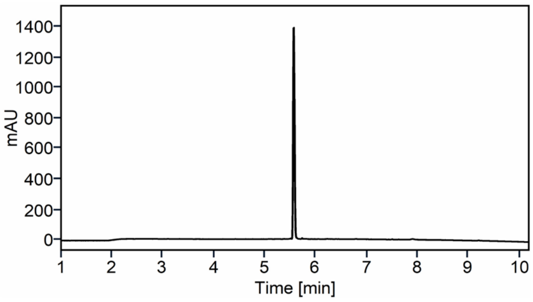

S. simulans AIP-III 7-mer (**53**)

λ = 215 nm, 5–95% B over 10 min

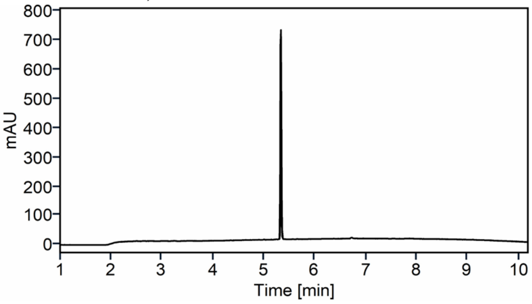

S. simulans AIP-II/III 6-mer (**54**)

λ = 215 nm, 5–95% B over 10 min

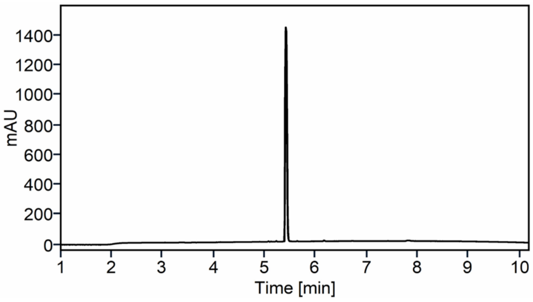

S. simulans AIP-II/III 5-mer *N*-Ac (**55**)

λ = 215 nm, 5–95% B over 10 min

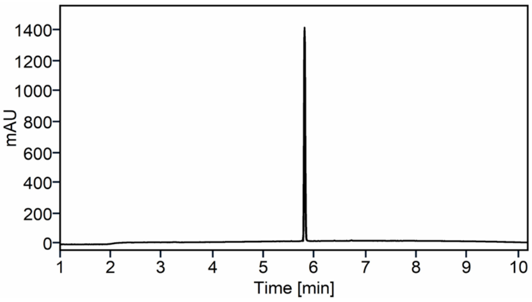

S. simulans AIP-II/III 5-mer *N*-Me_2 (**56**)

λ = 215 nm, 5–50% B over 10 min

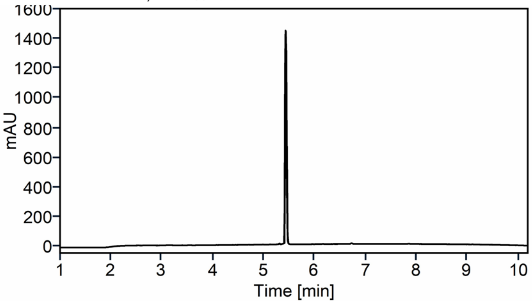

Copies of NMR spectra

***S. epidermidis* AIP-I (5)**

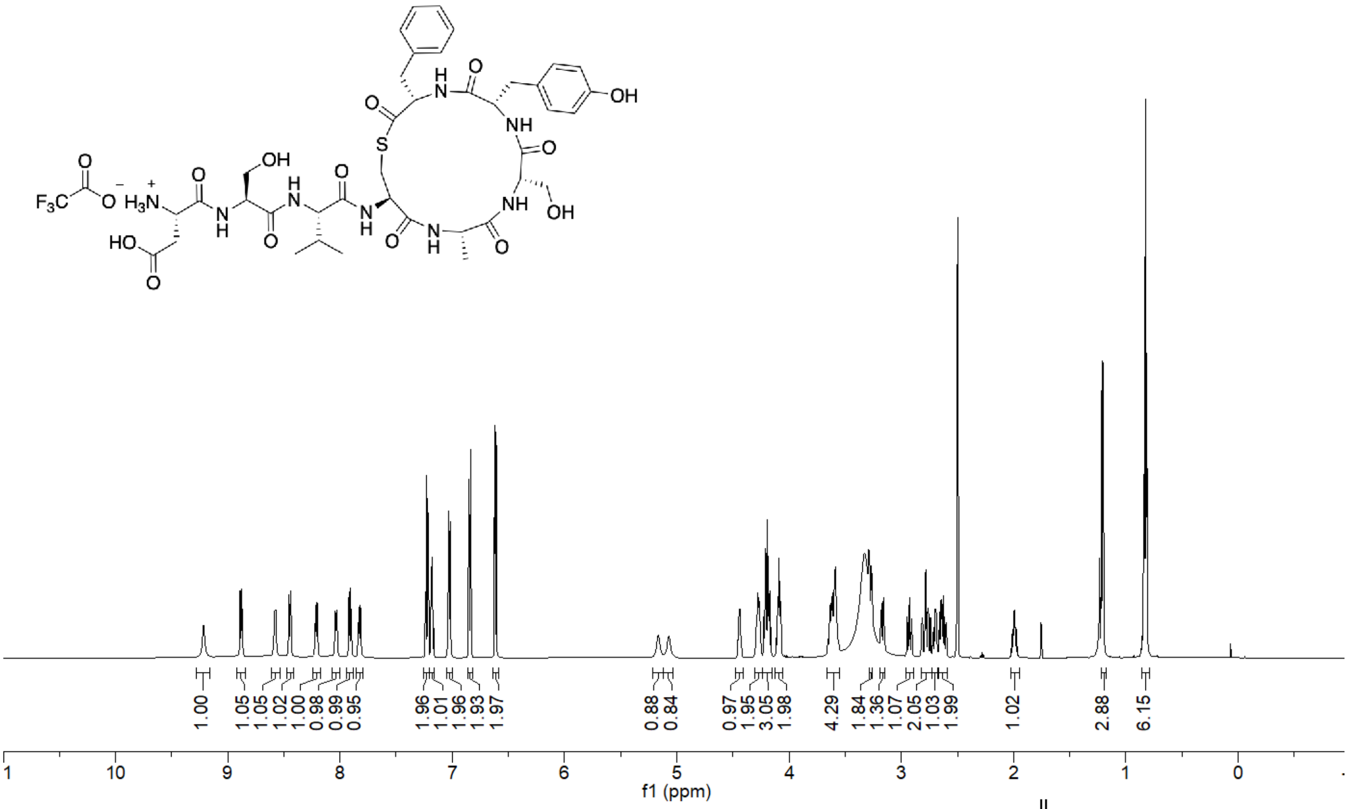

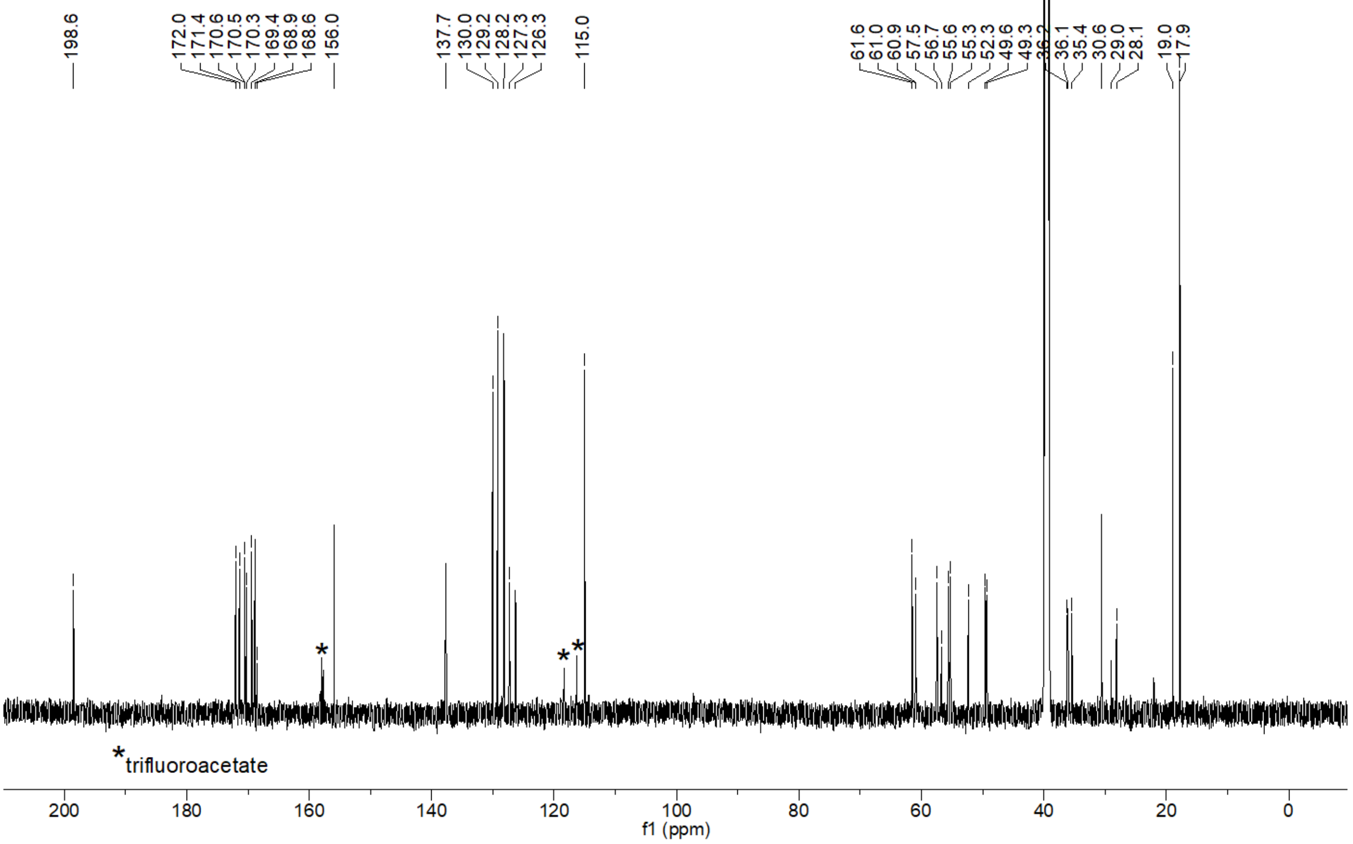

***S. epidermidis* AIP-II (6)**

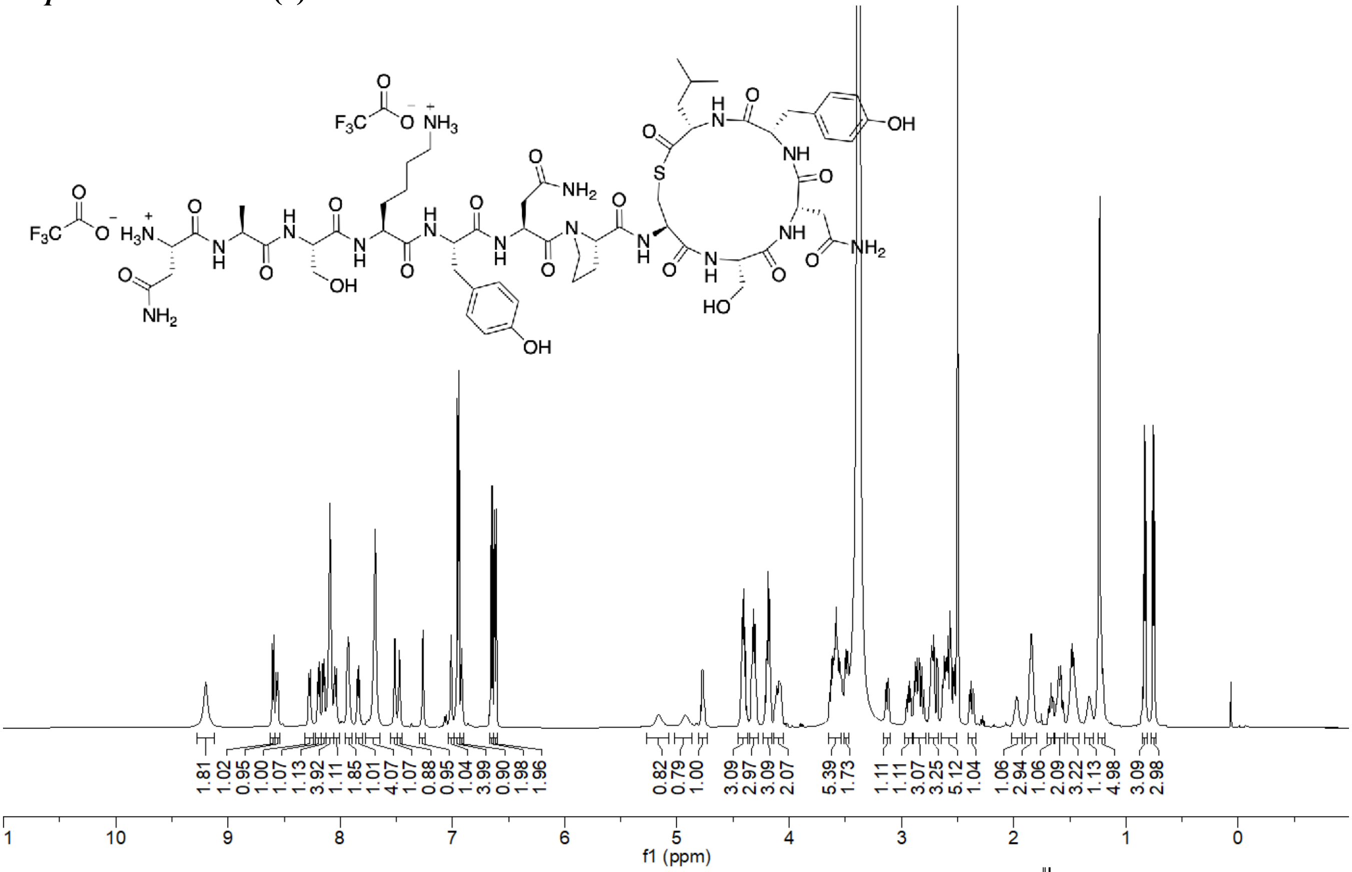

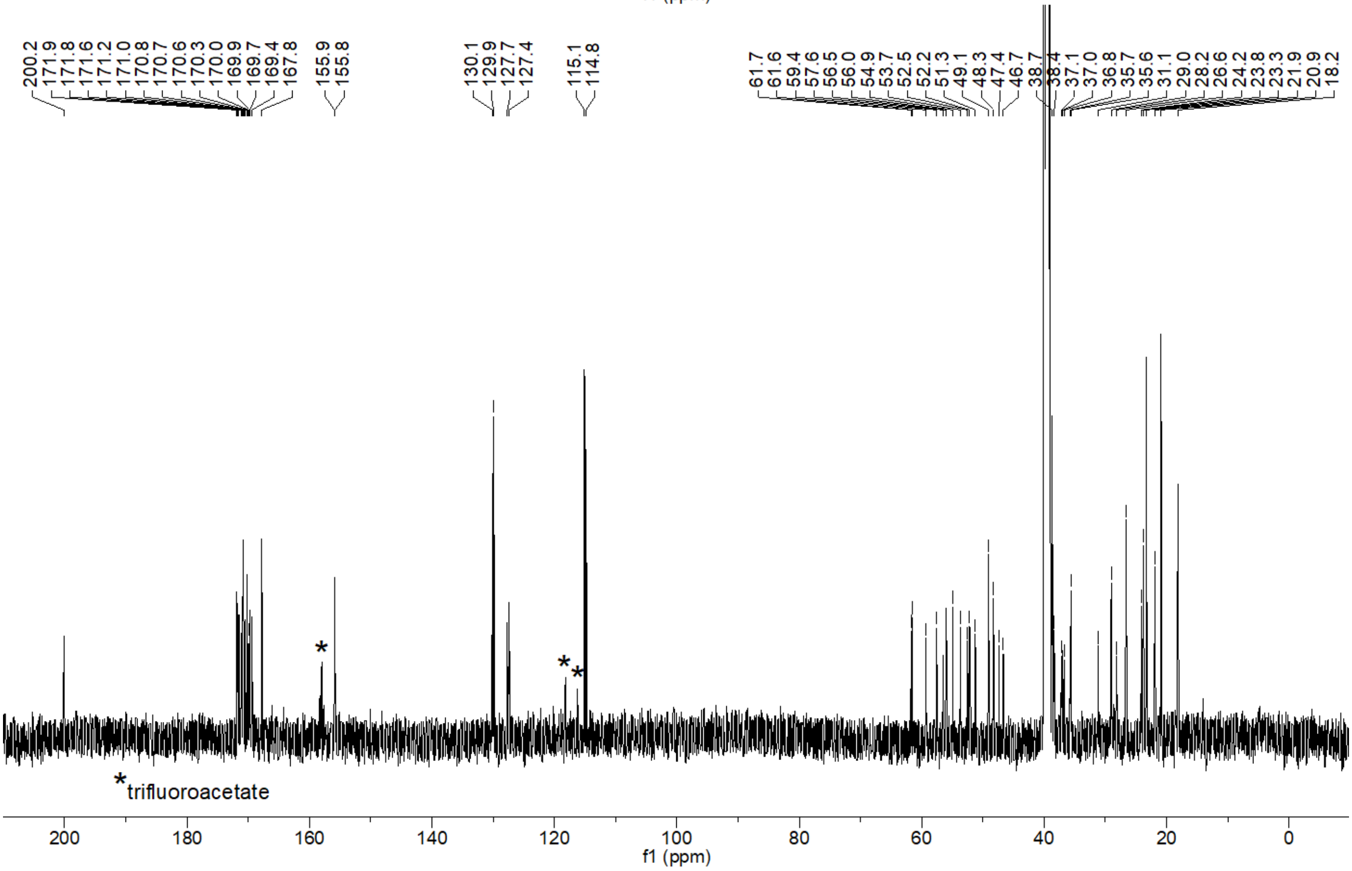

***S. epidermidis* AIP-III (7)**

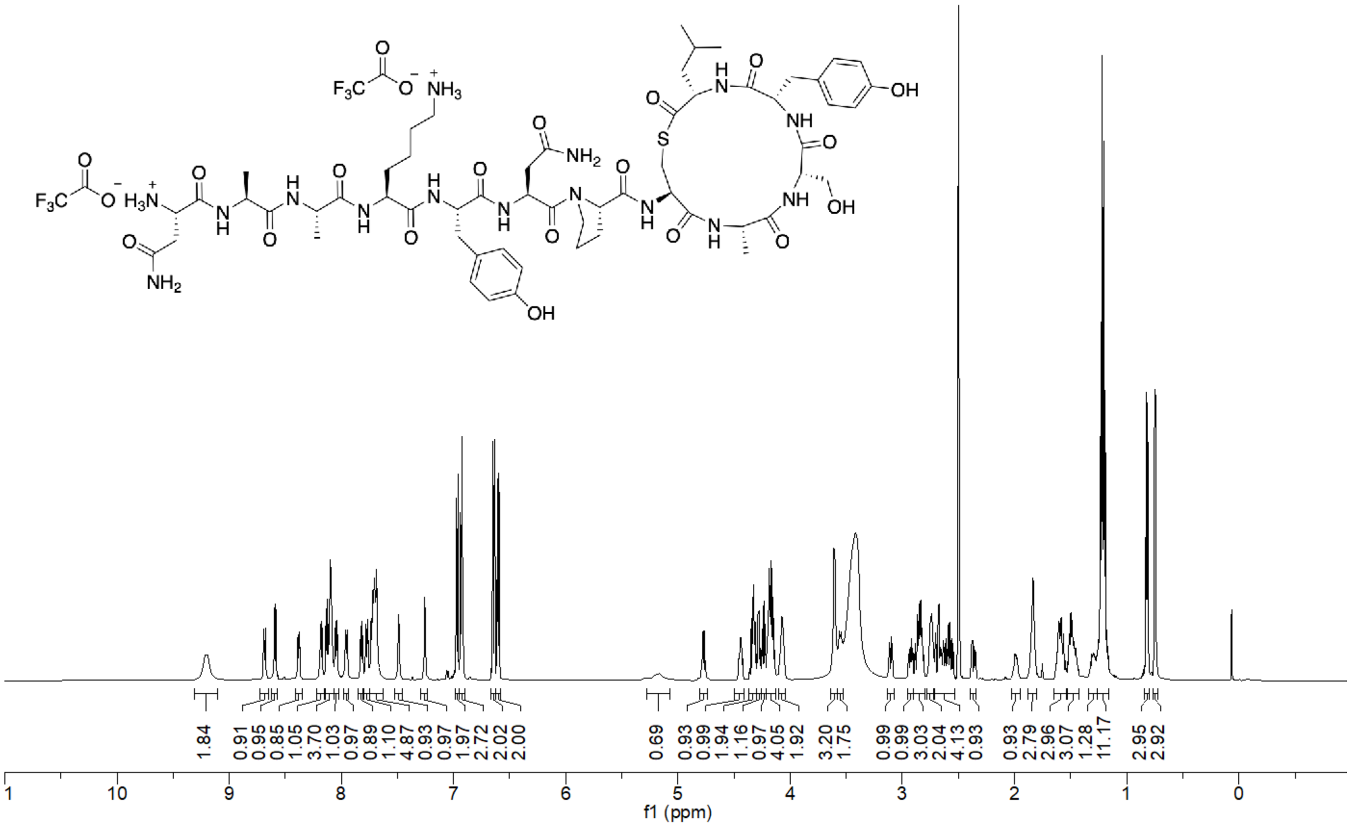

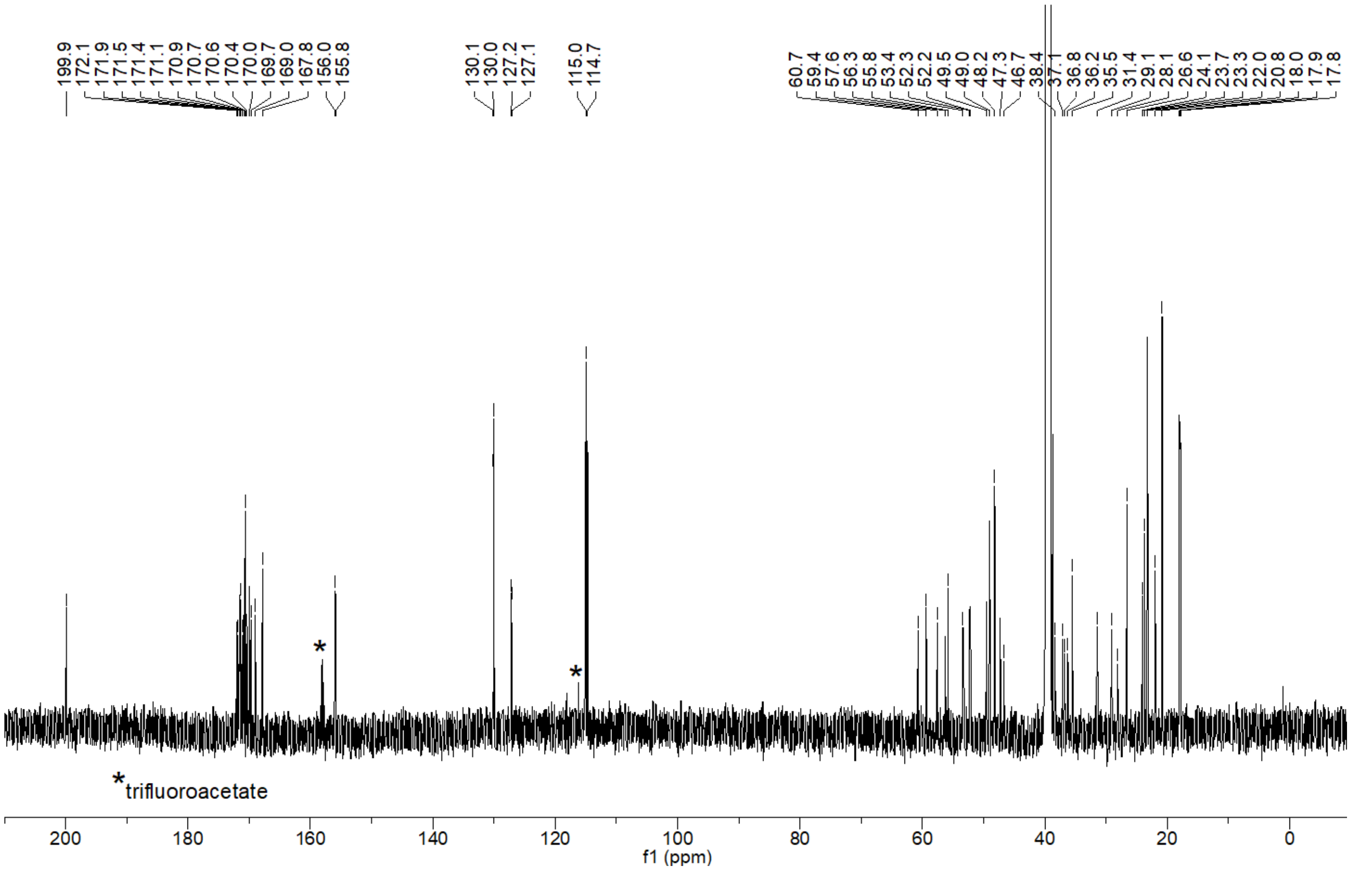

***S. lugdunensis* AIP-I (8)**

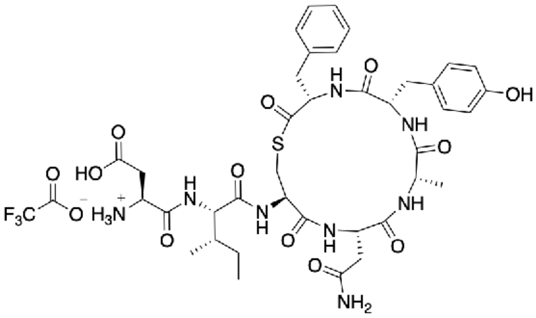

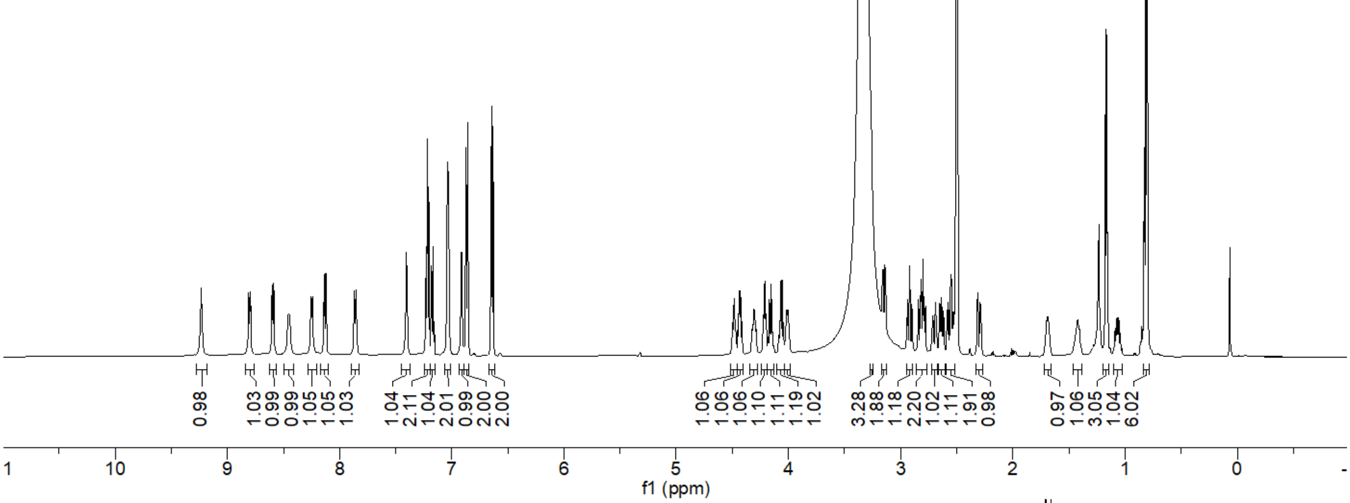

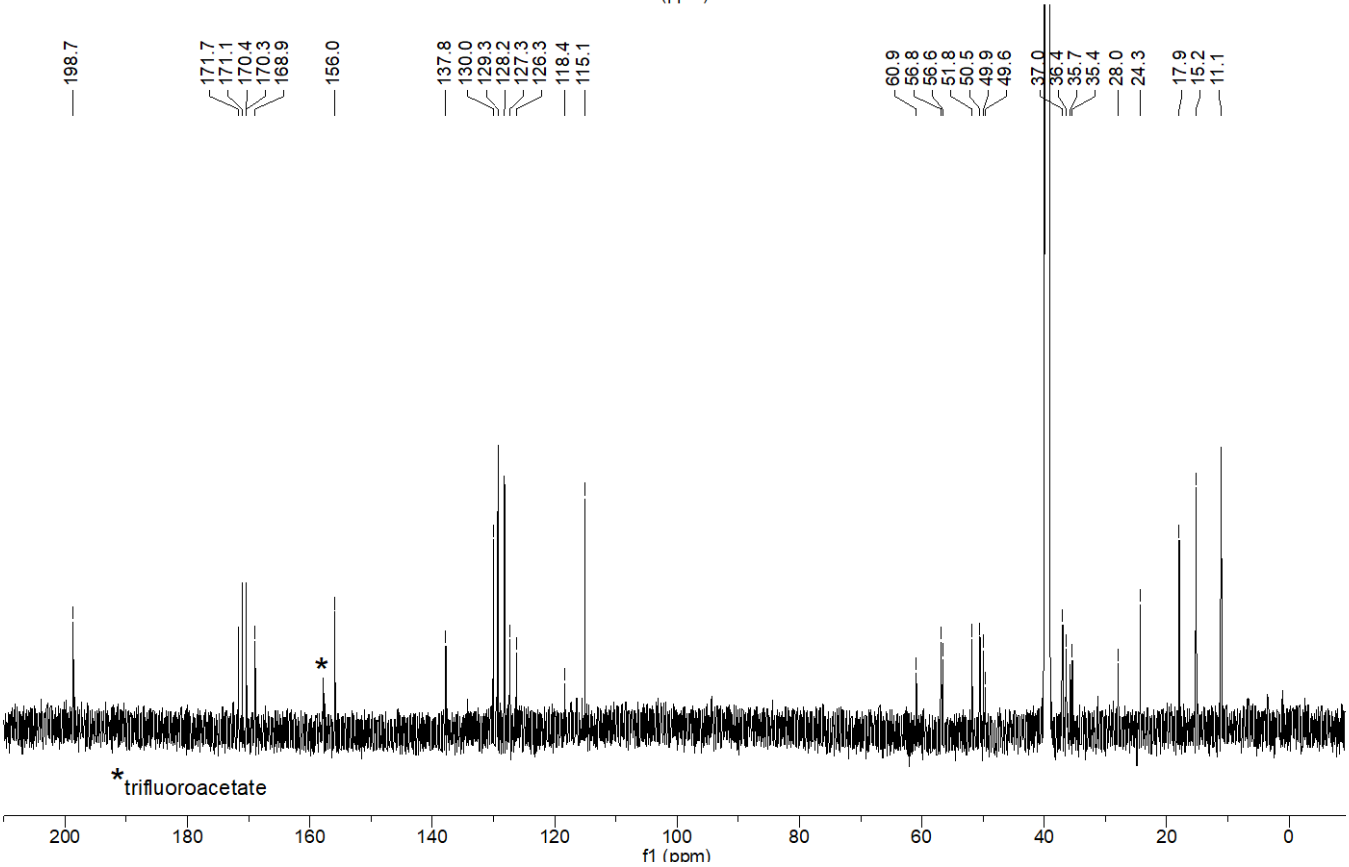

S. hominis **AIP-I (10)**

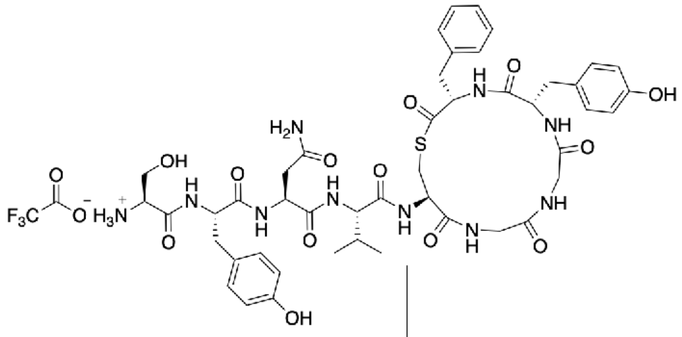

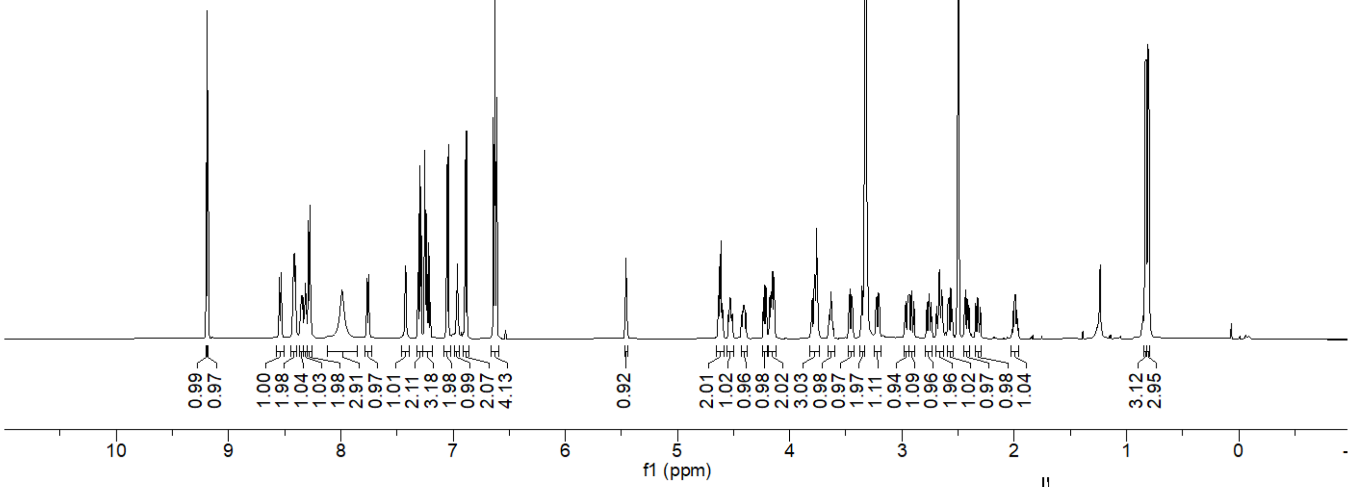

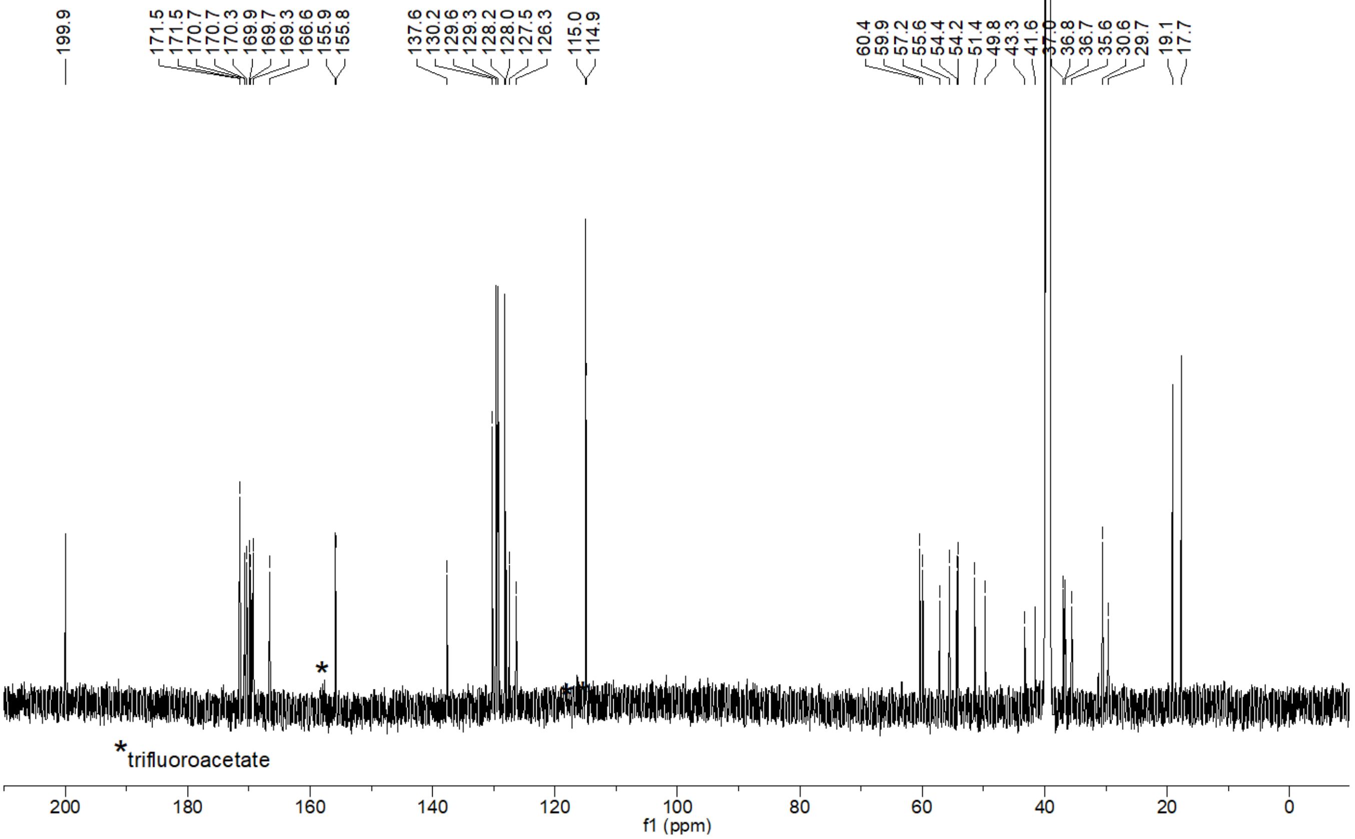

***S. hominis* AIP-II (11)**

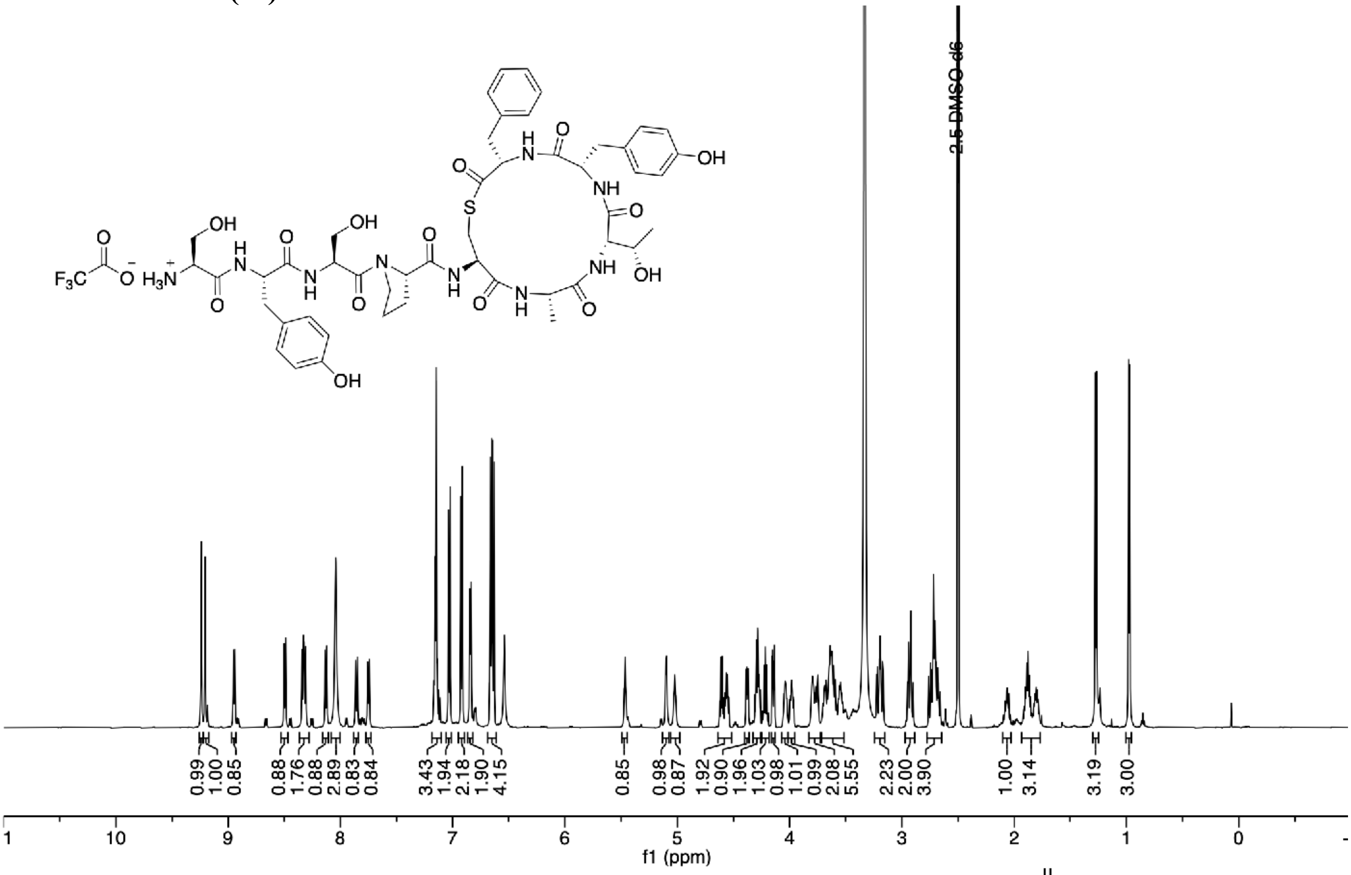

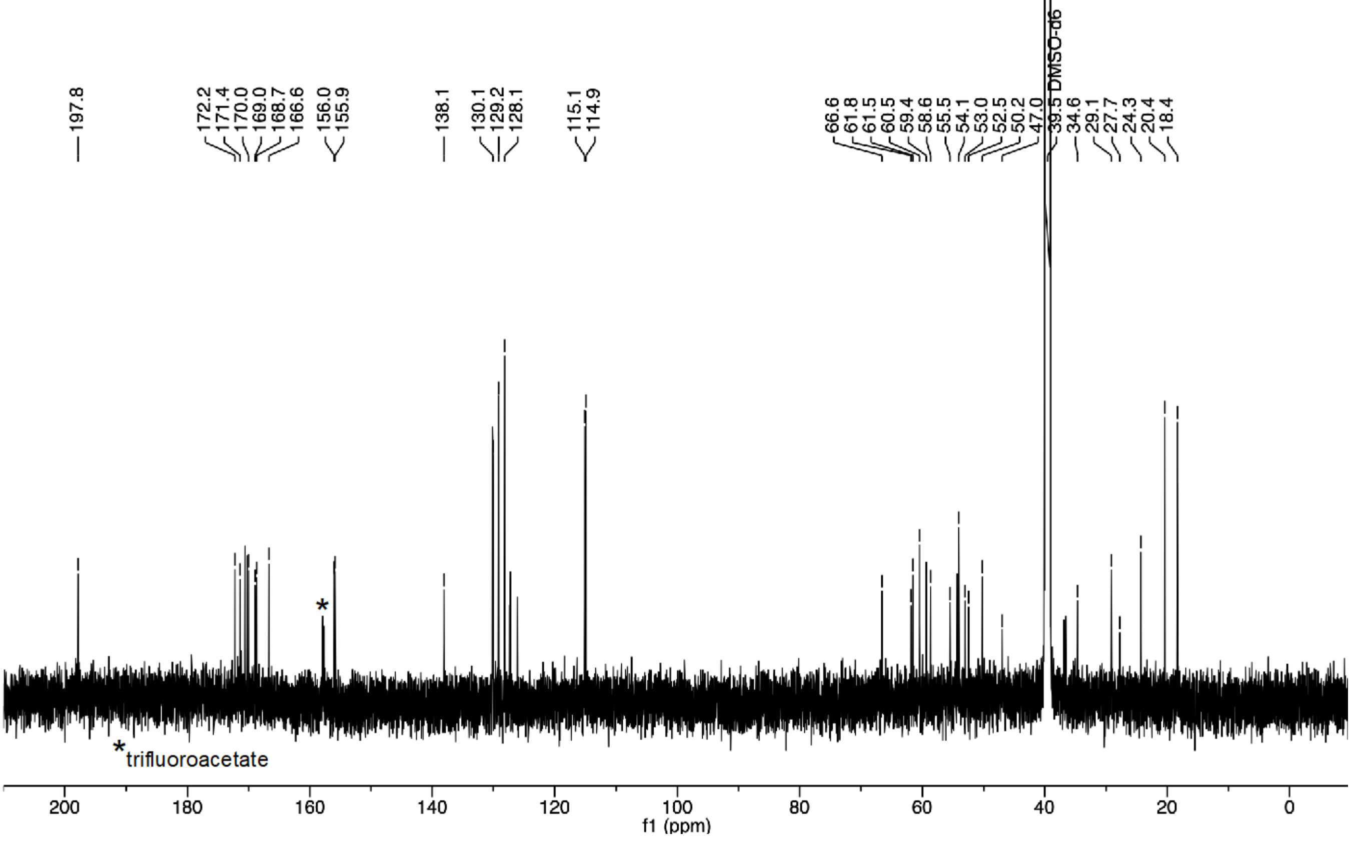

***S. hominis* AIP-IV (13)**

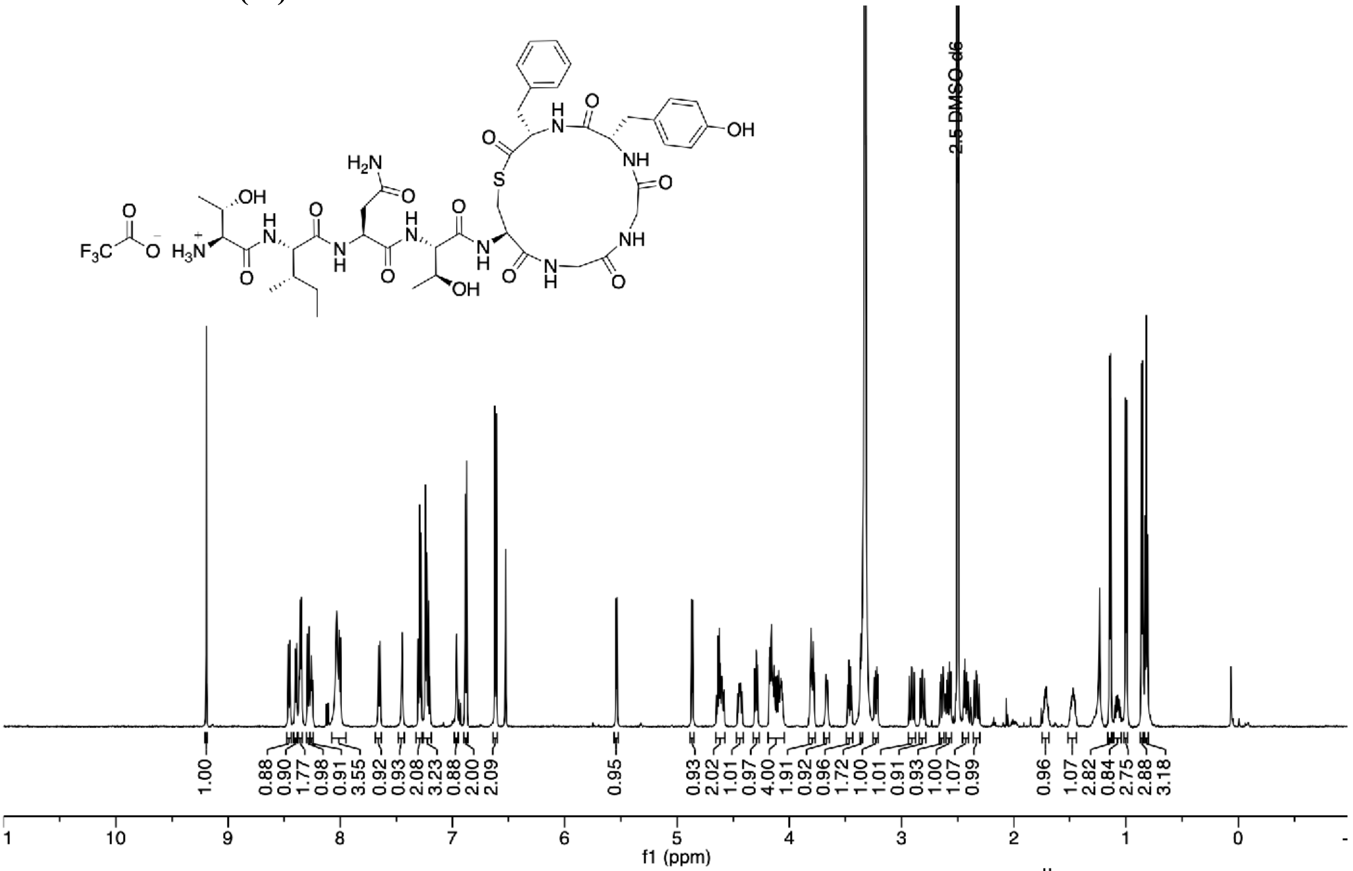

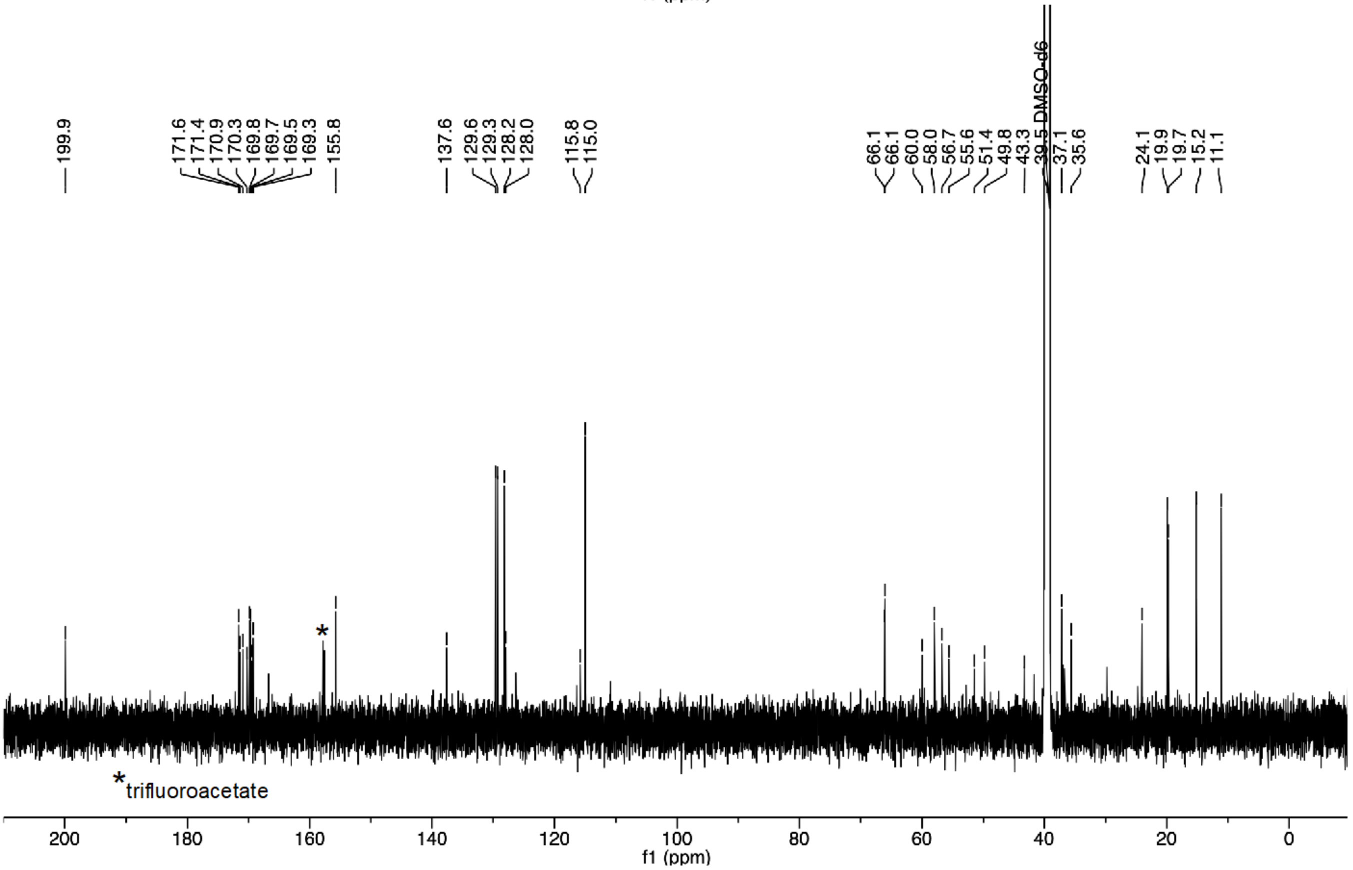

***S. hominis* AIP-V (14)**

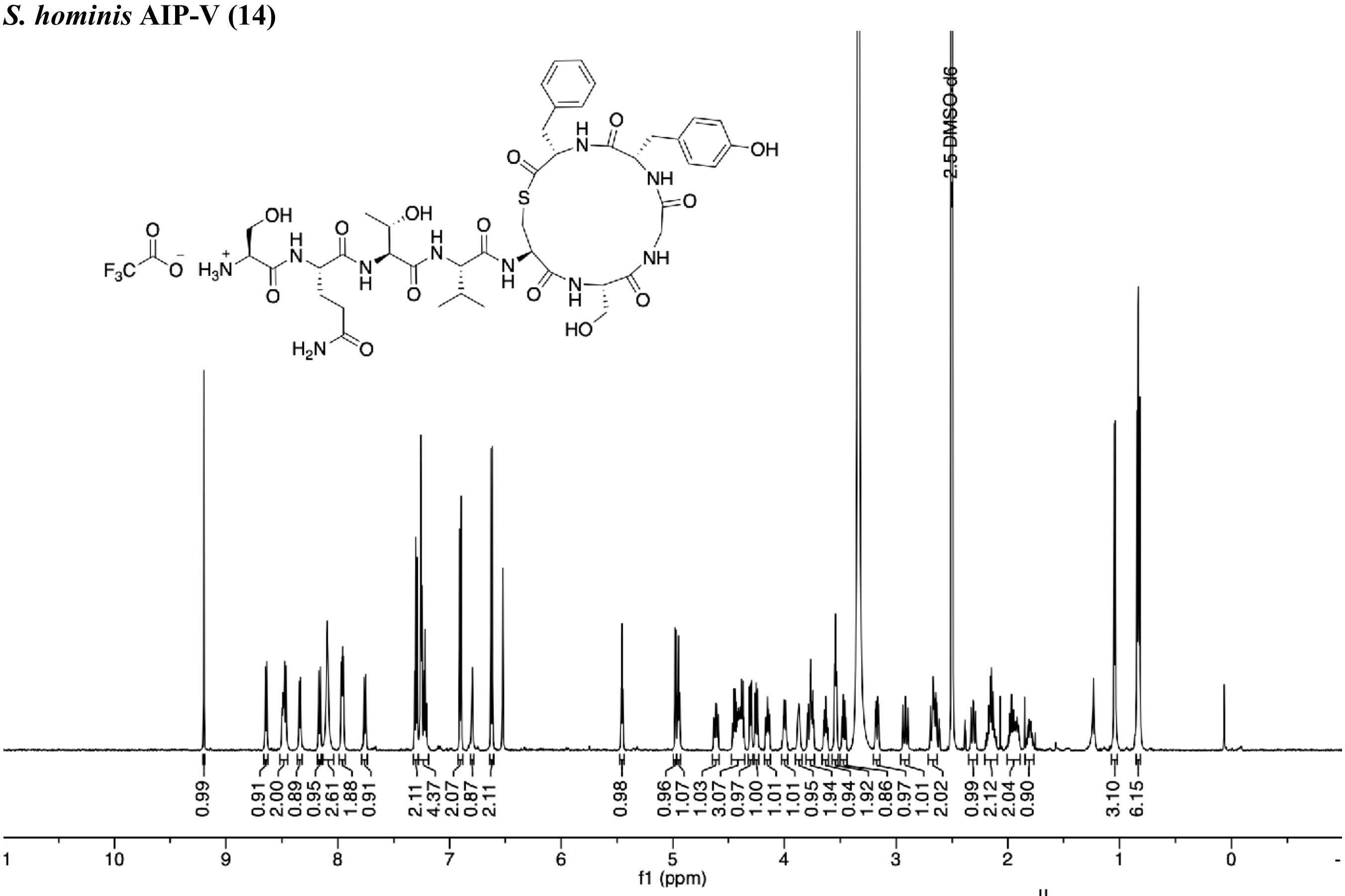

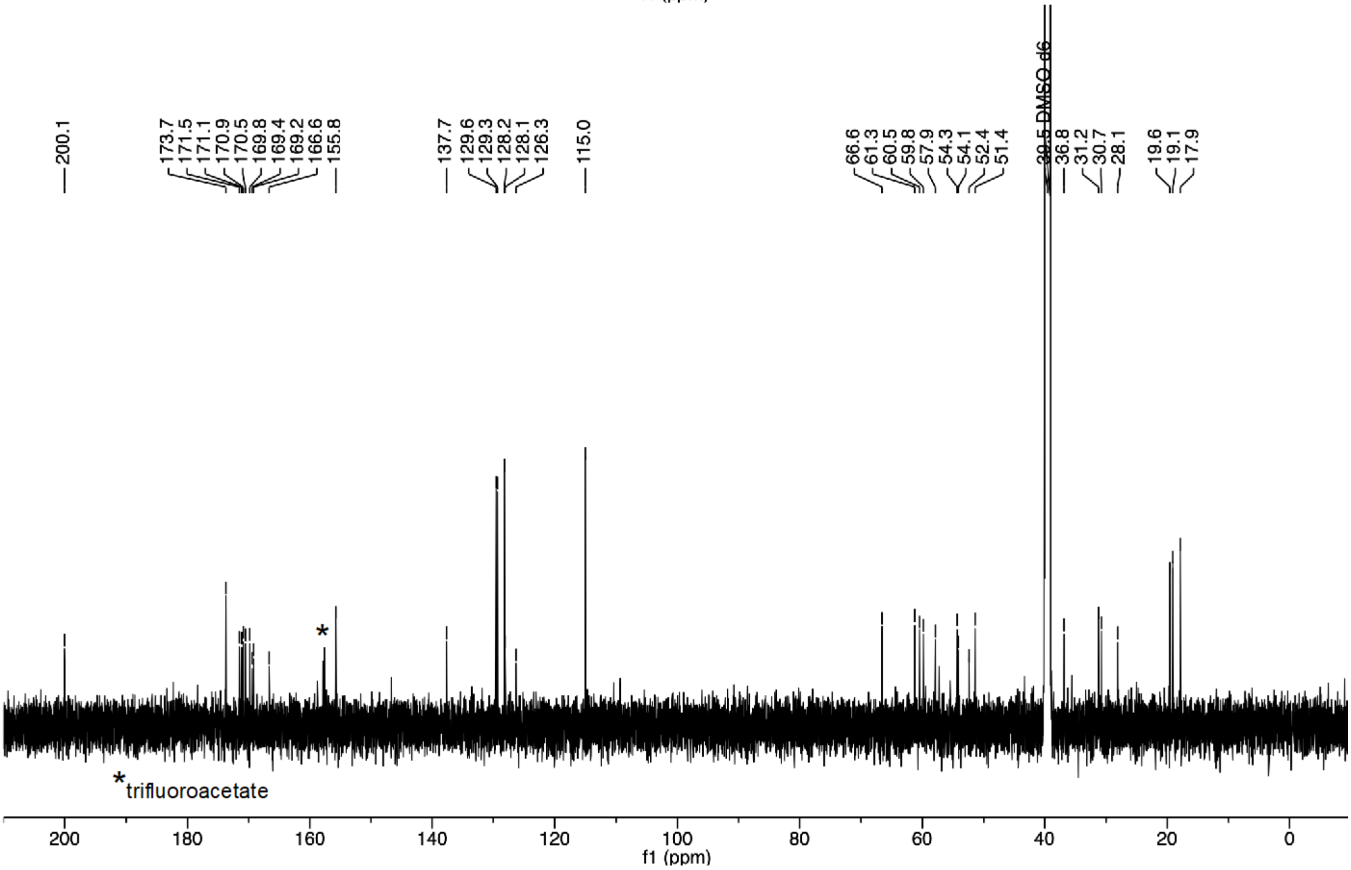

***S. warneri* AIP-II (17)**

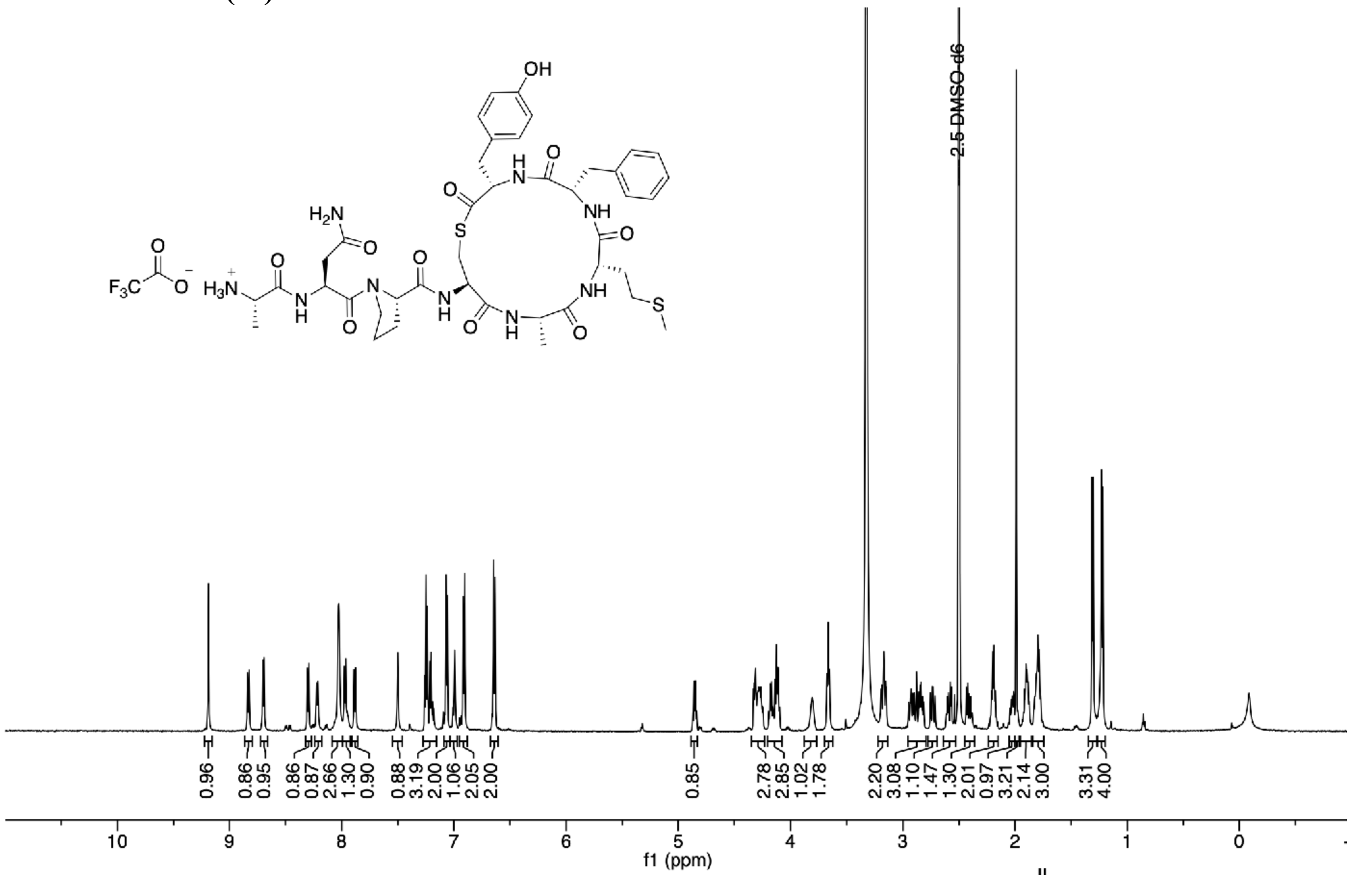

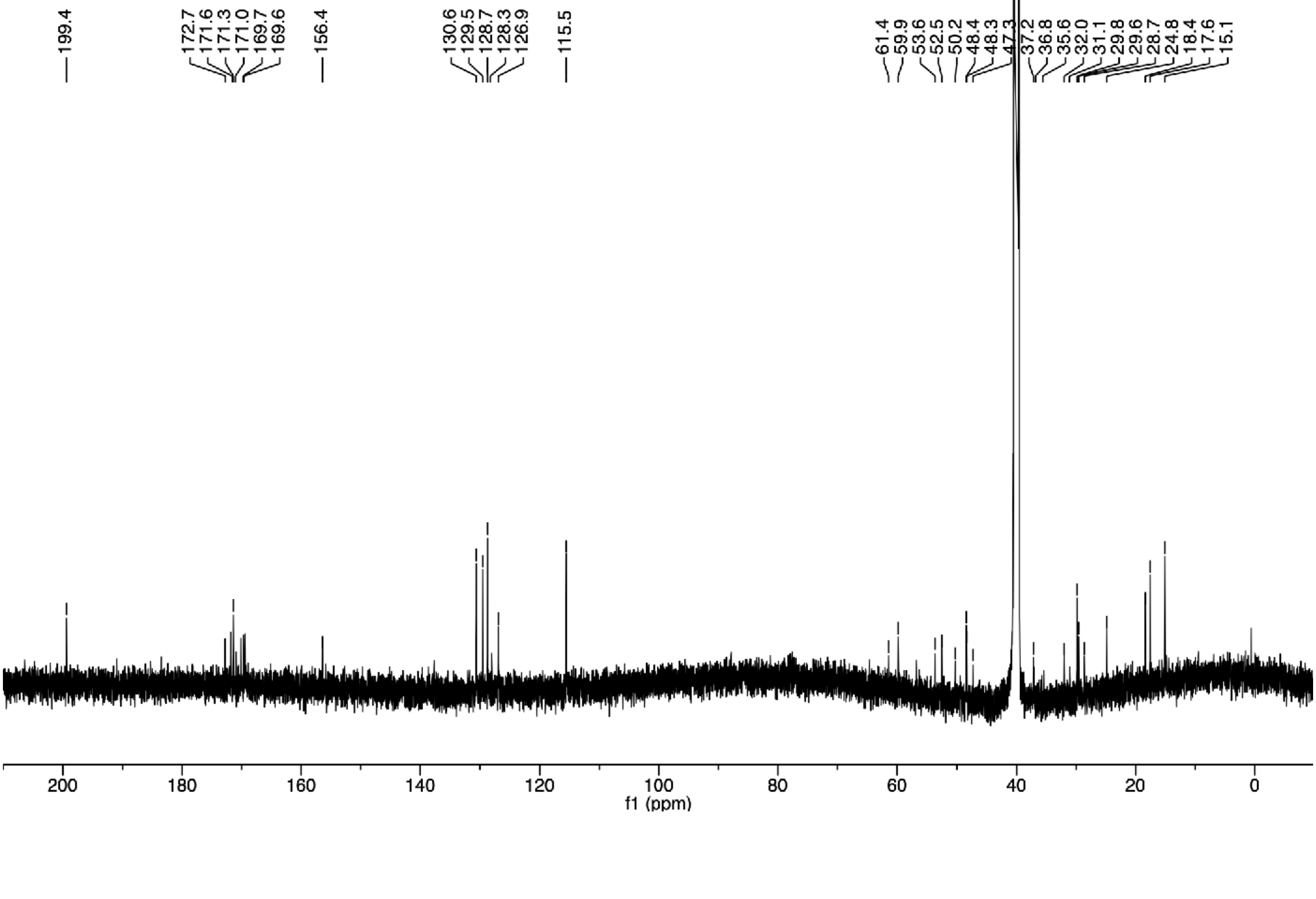

***S. cohnii* AIP-I (18)**

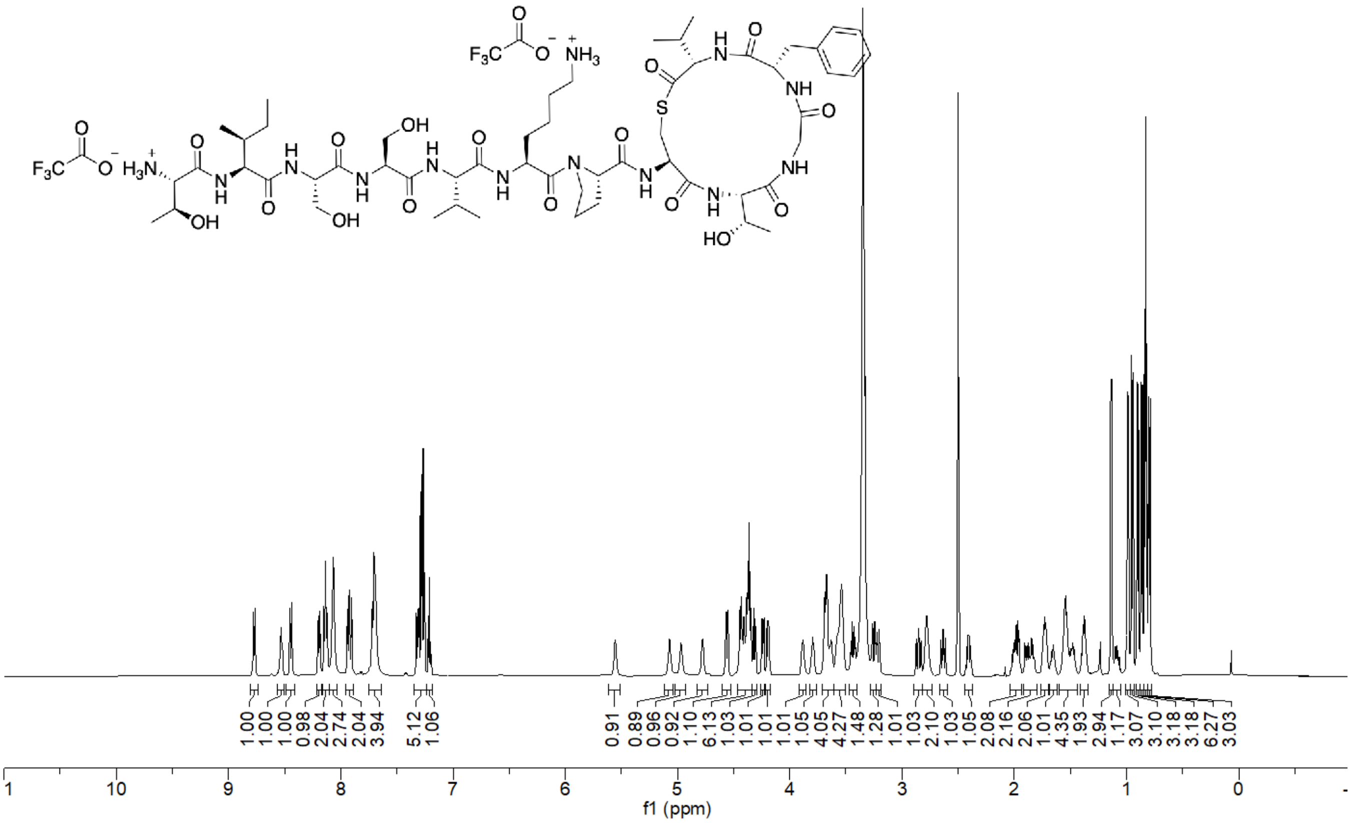

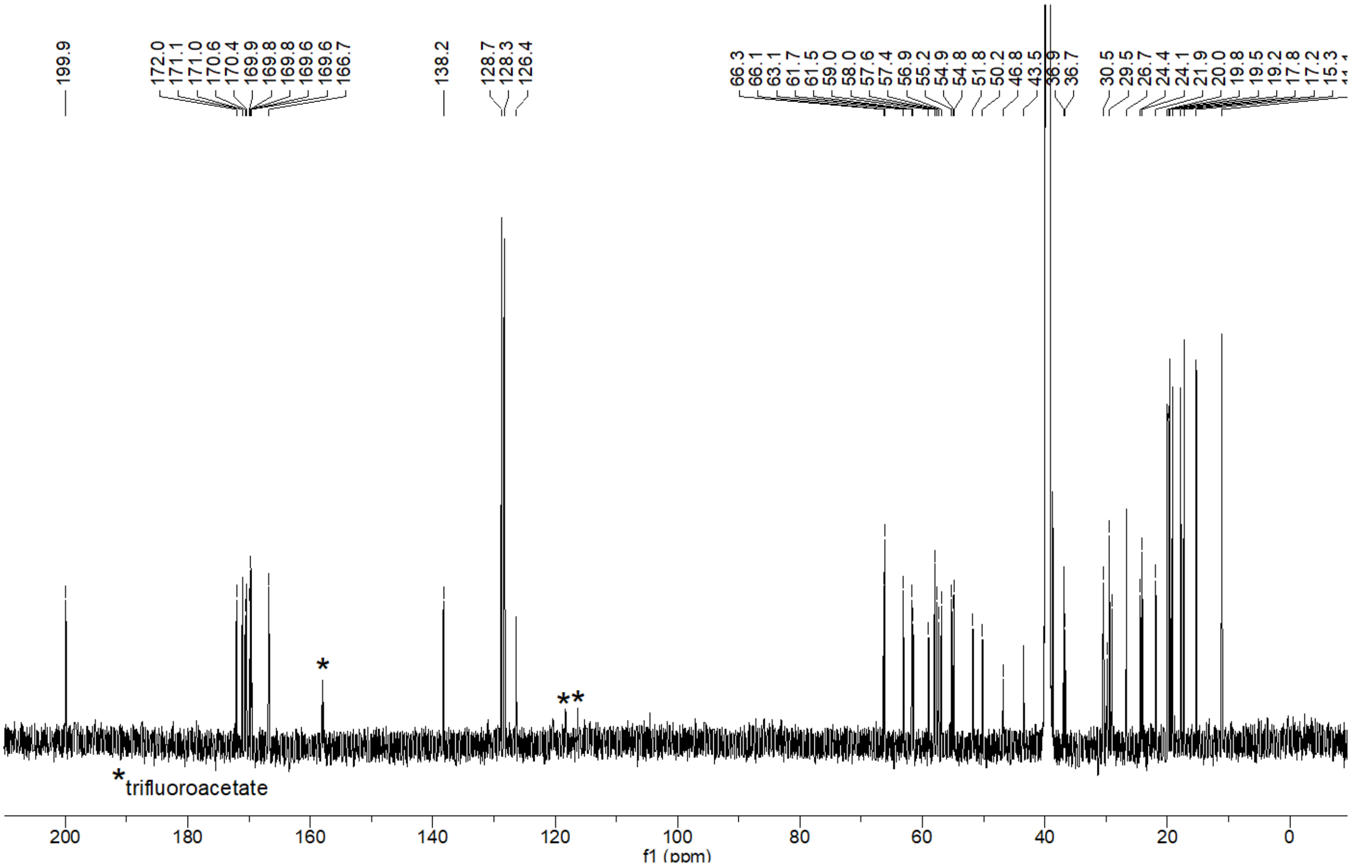

***S. caprae* AIP-I (20)**

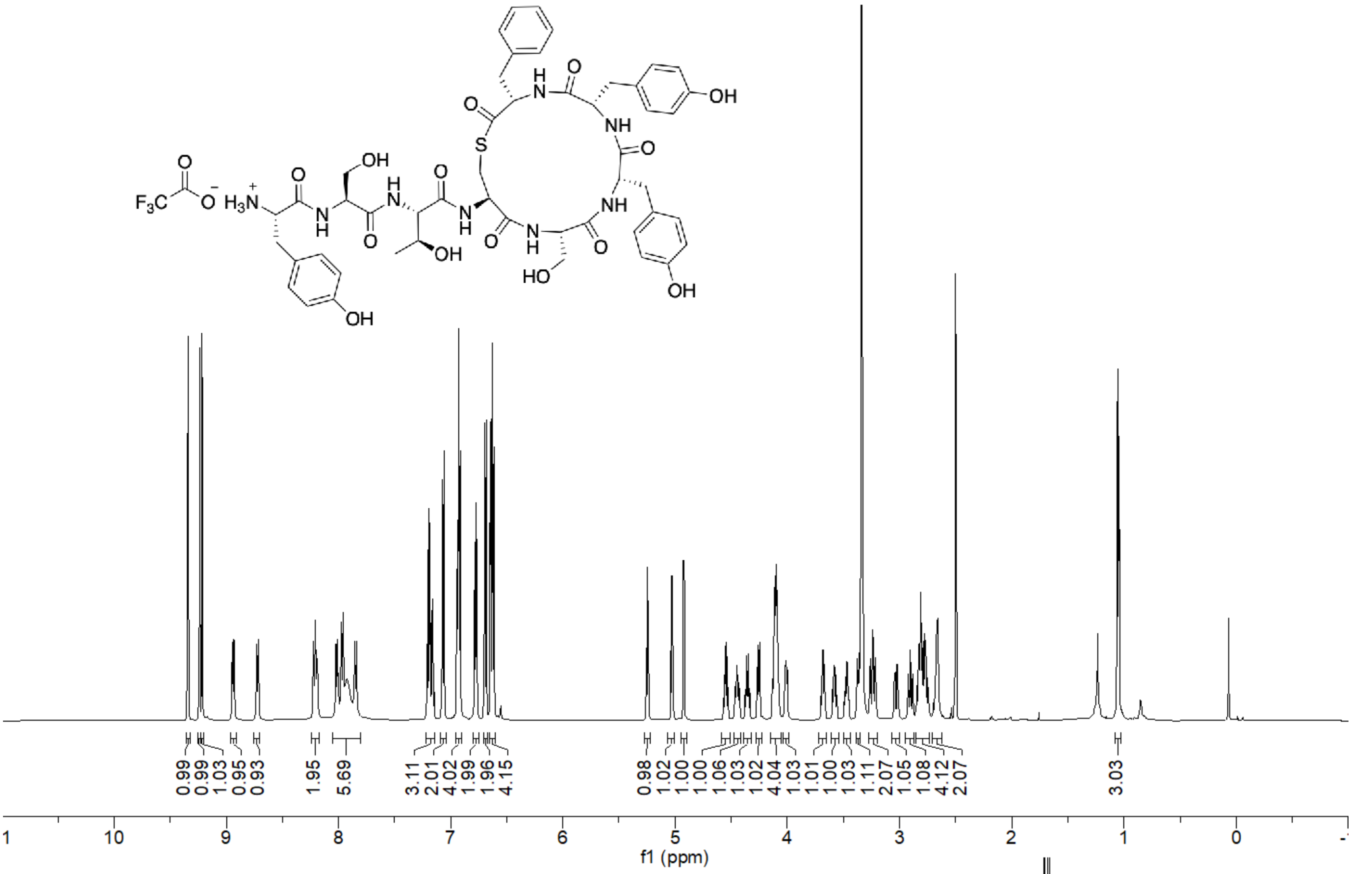

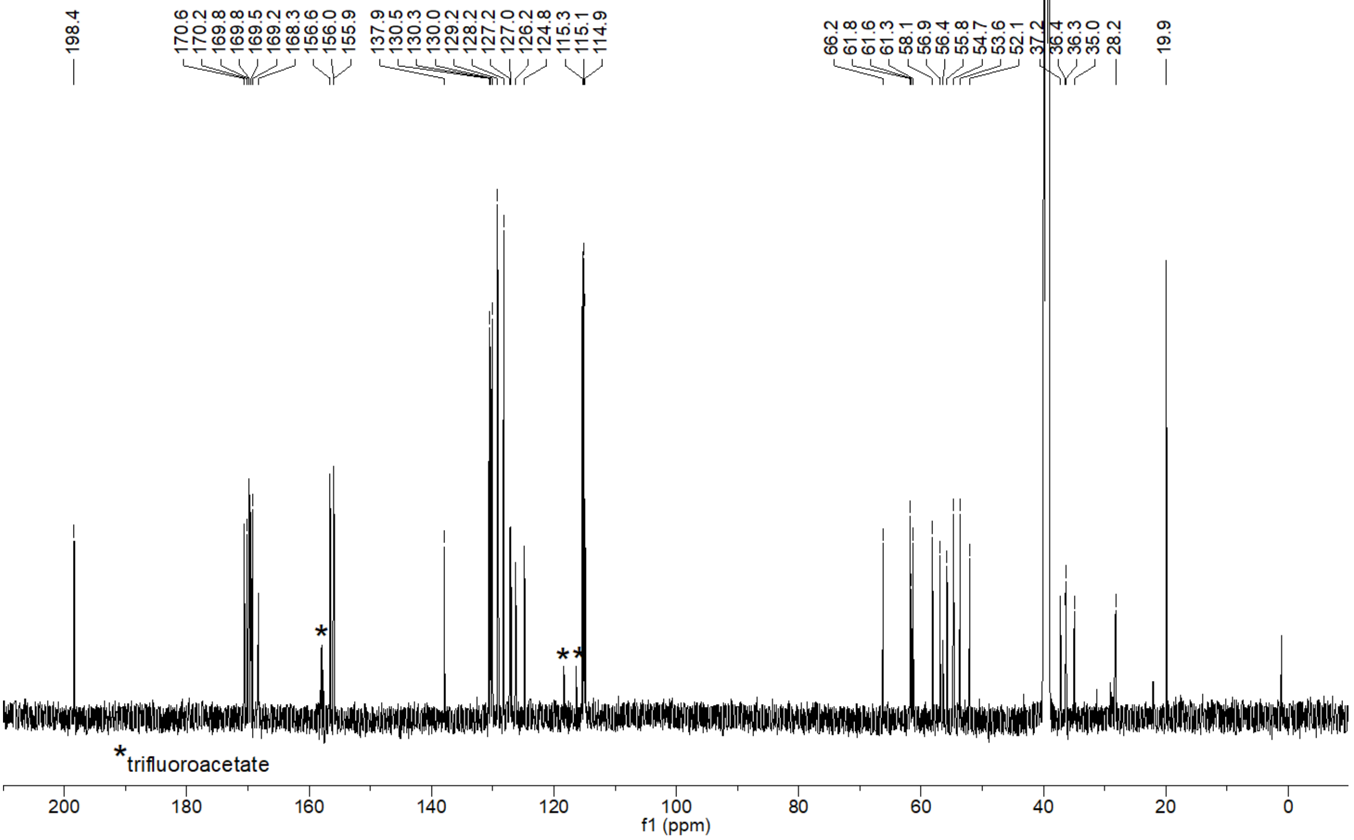

S. pasteuri **AIP-I (21)**

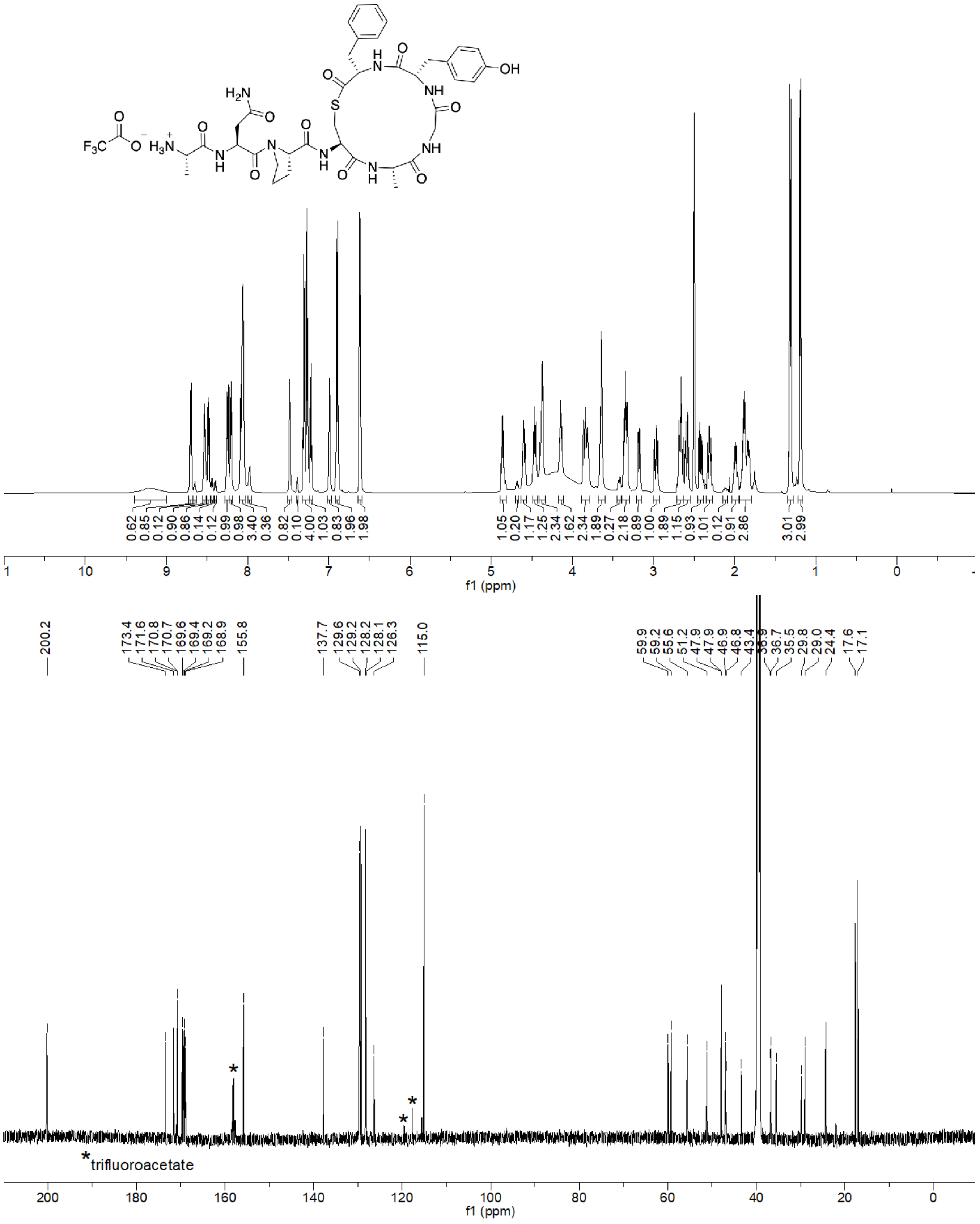

S. devriesei **AIP-I (22)**

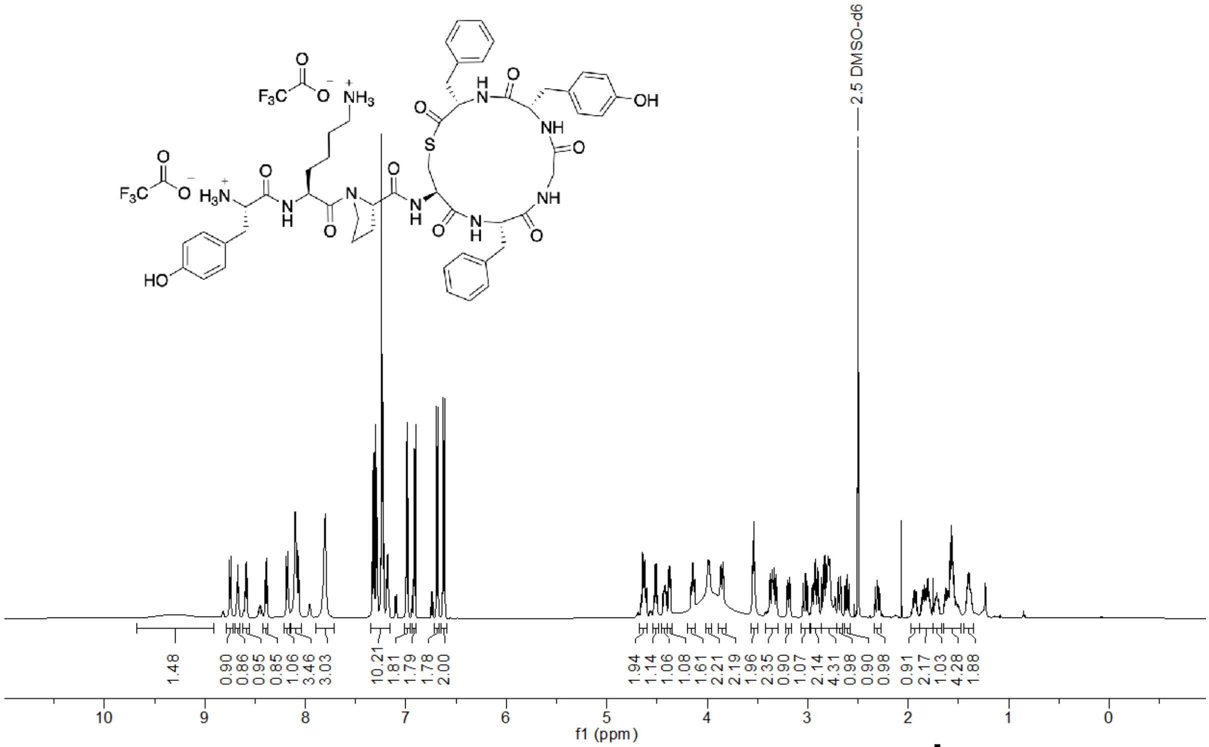

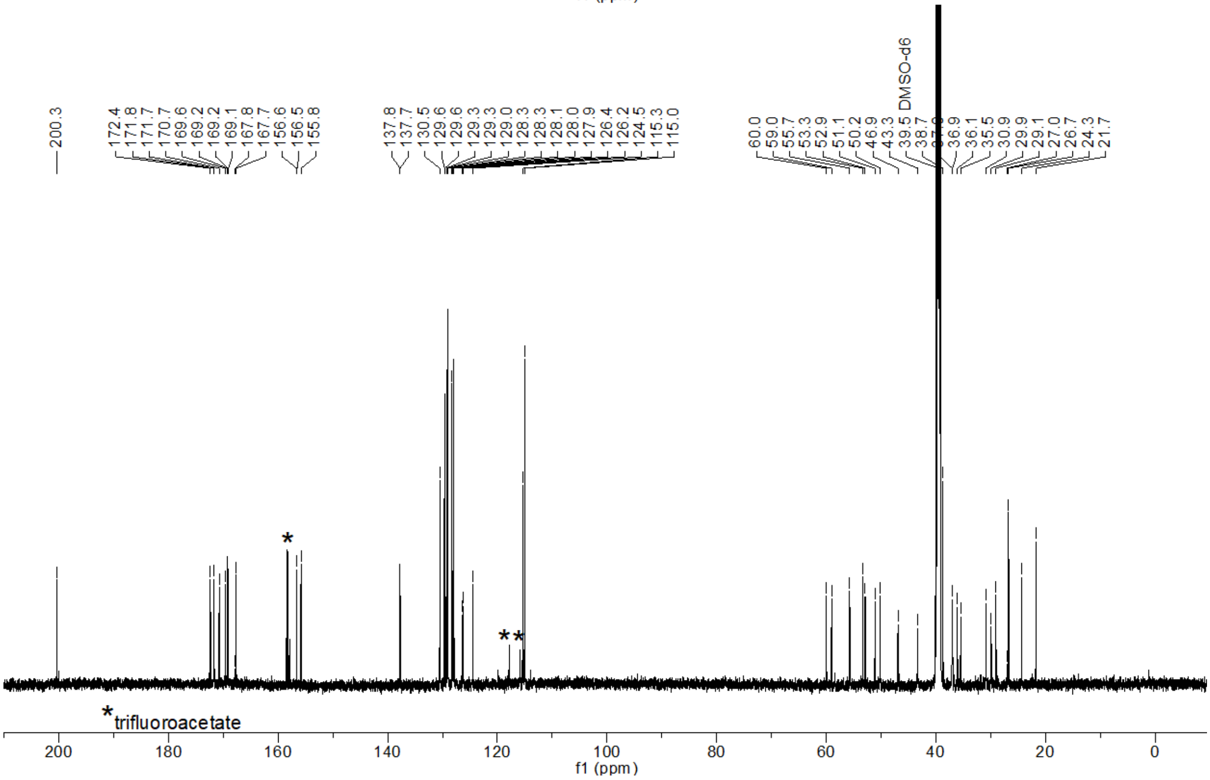

***S. succinus* AIP-I (23)**

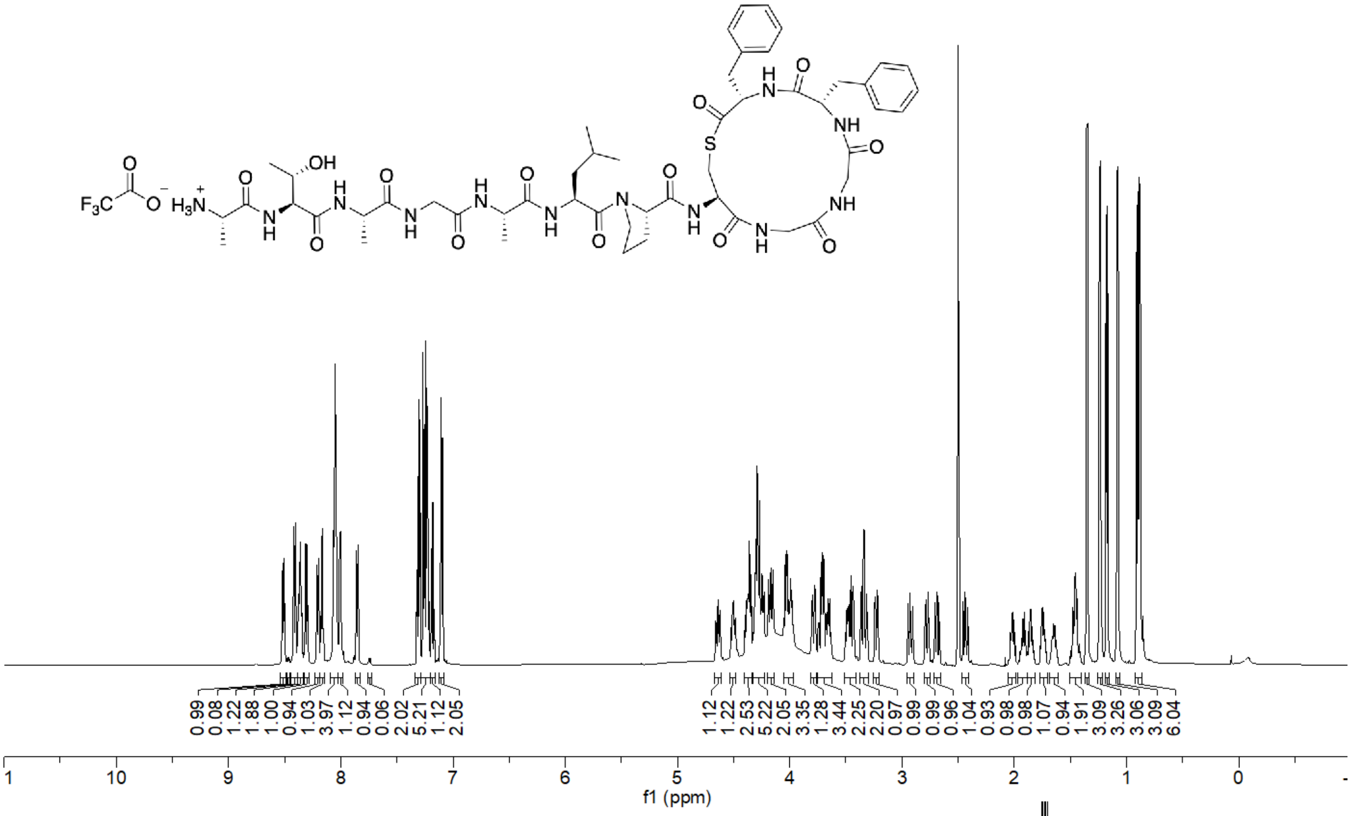

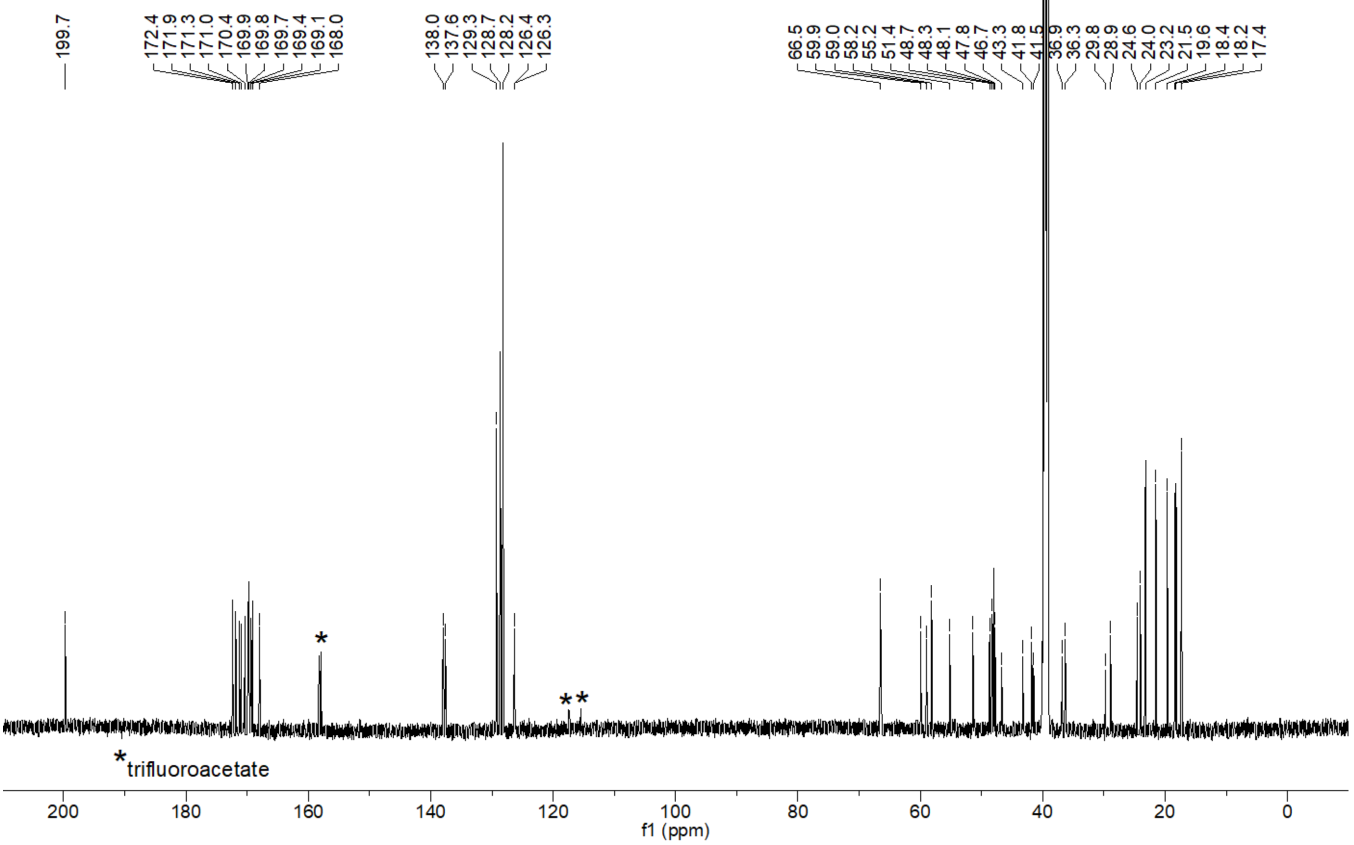

***S. equorum* AIP-I (24)**

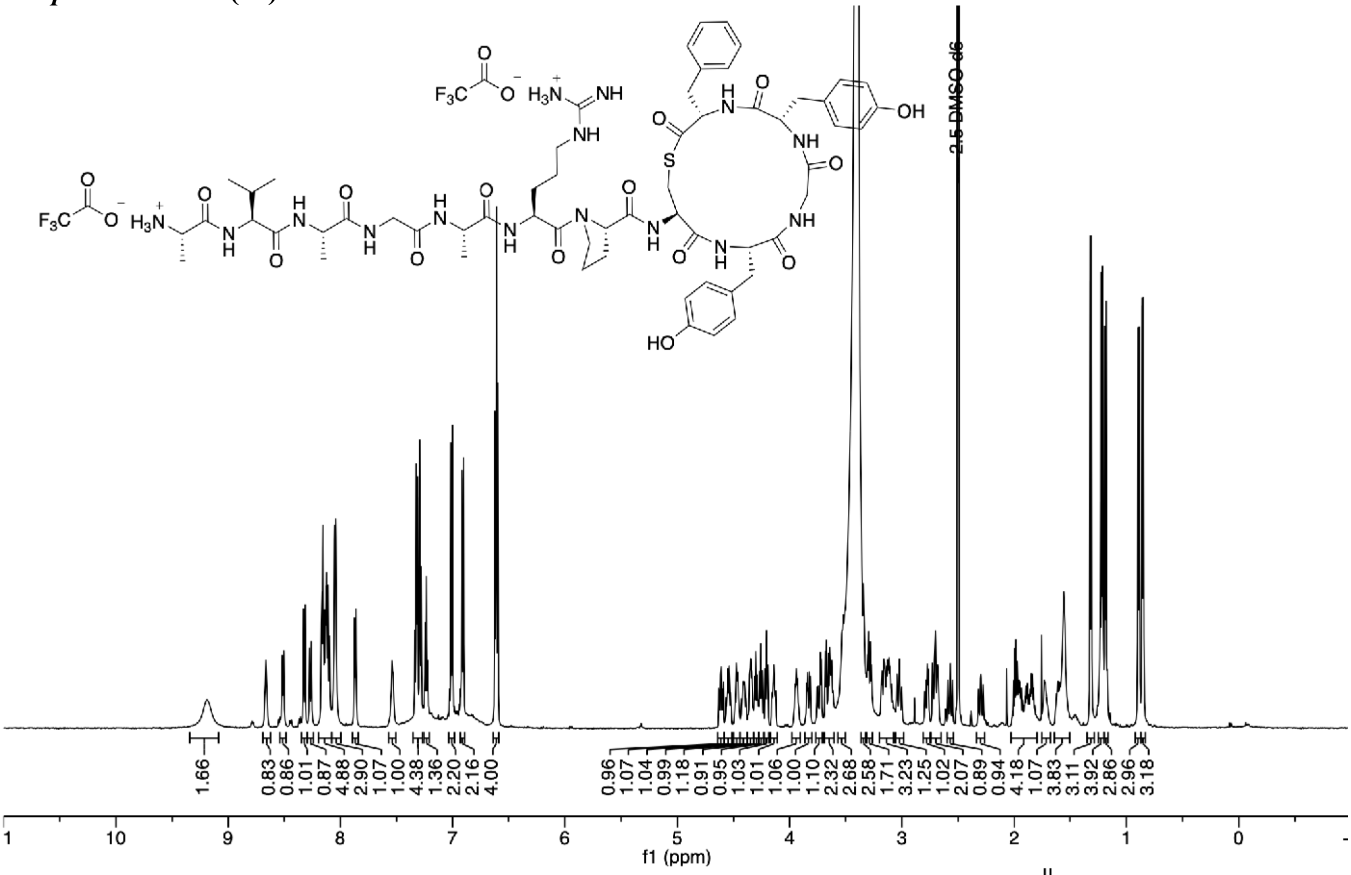

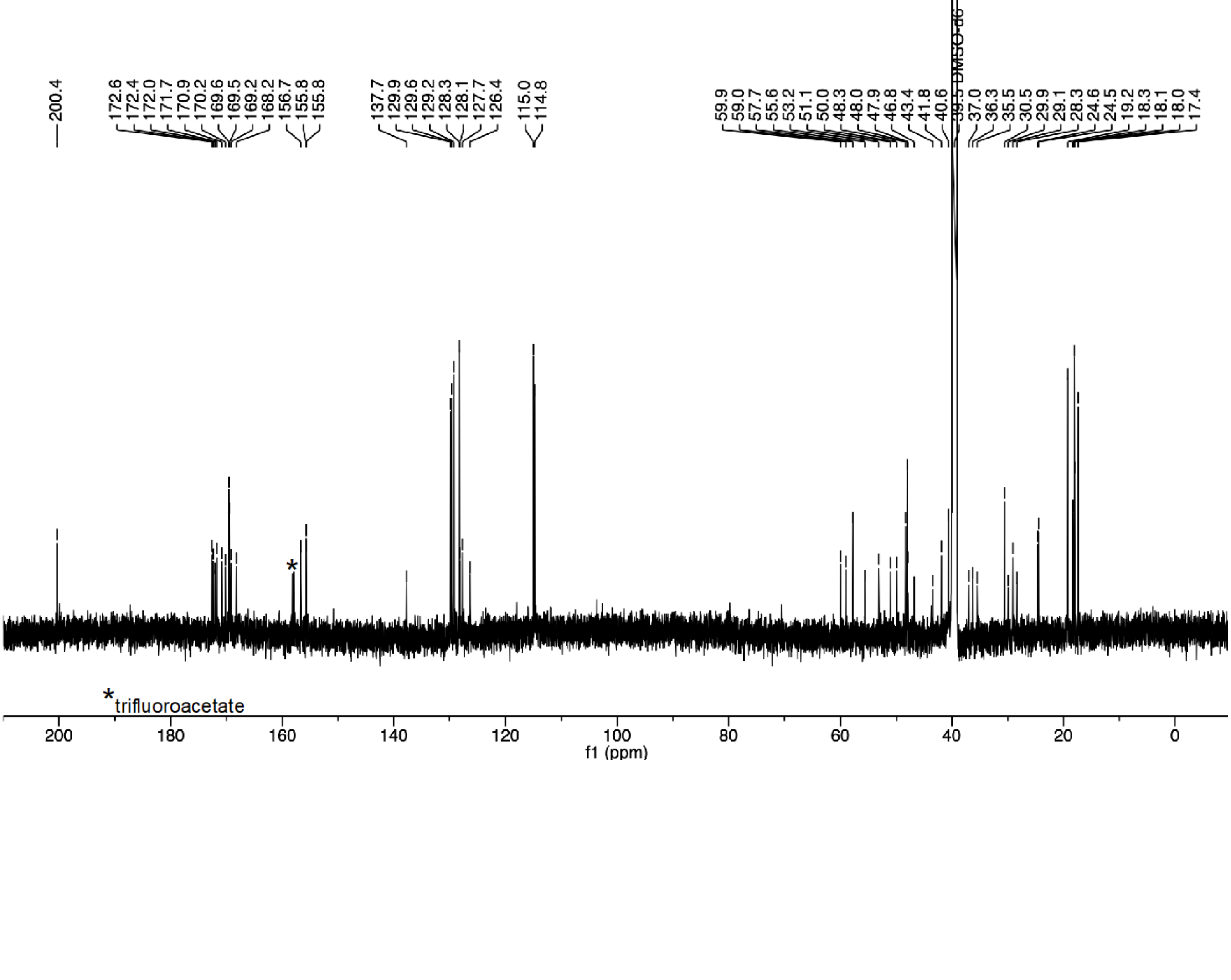

***S. equorum* AIP-II (25)**

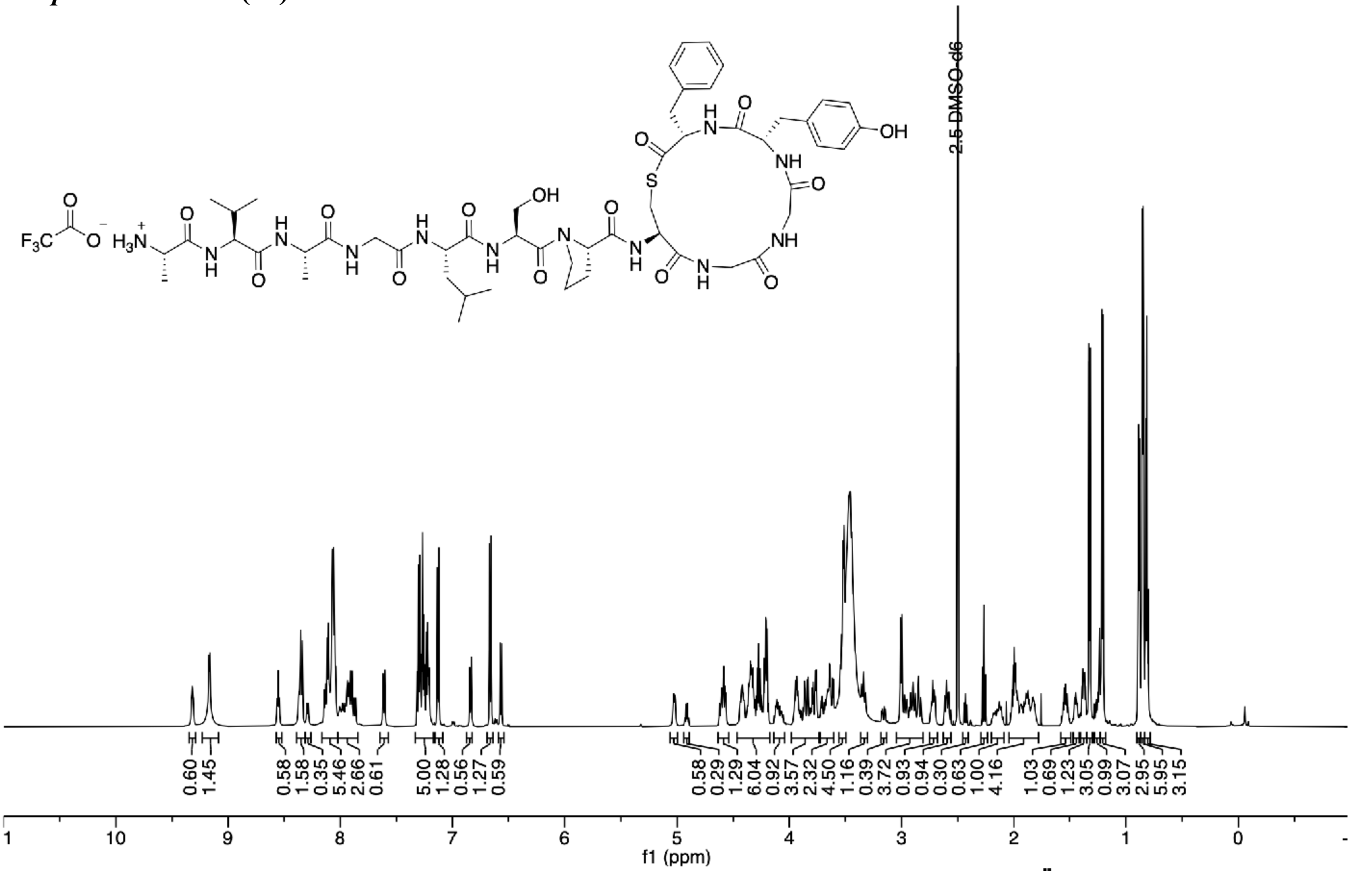

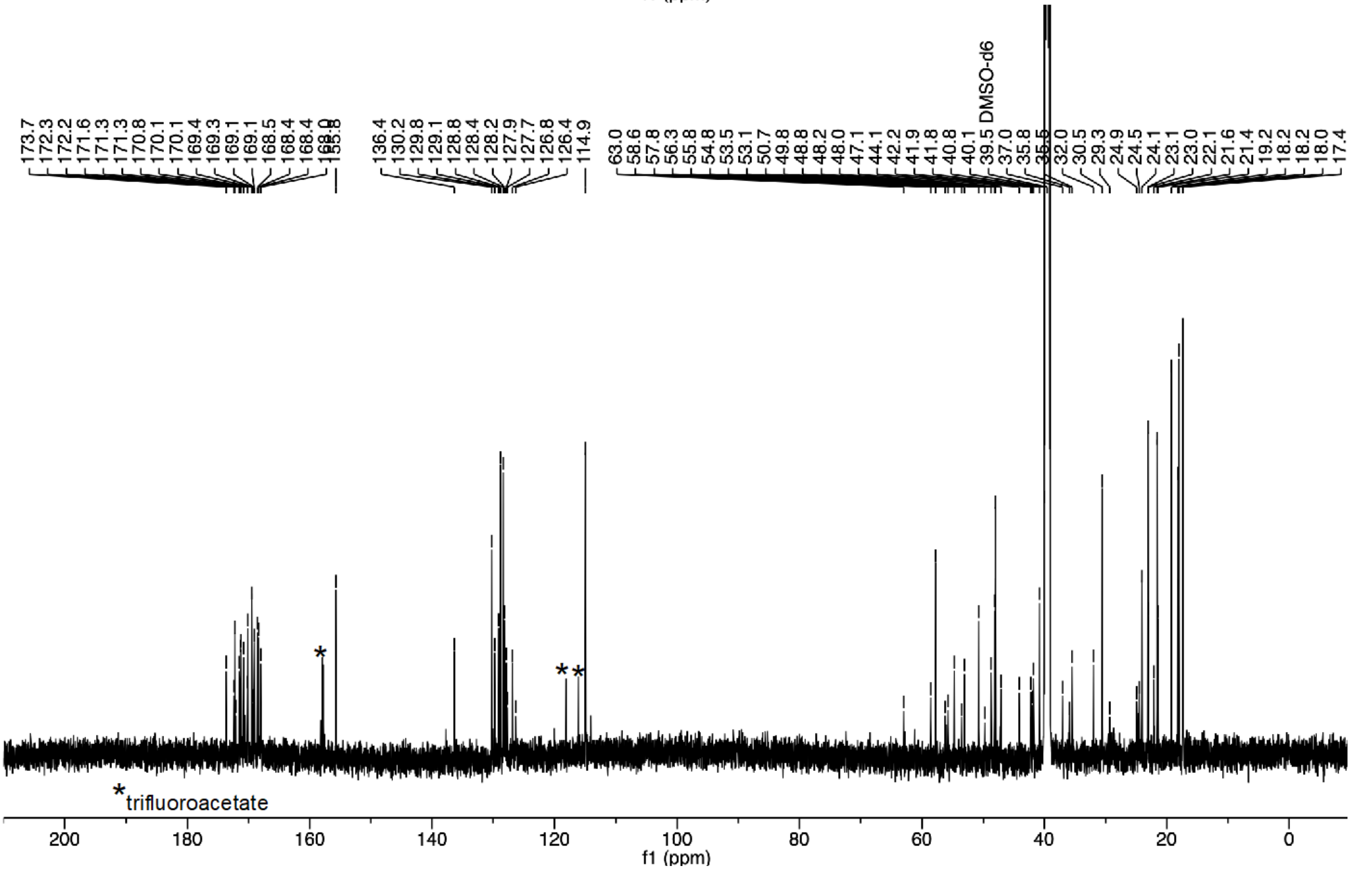

S. intermedius **AIP-I (31)**

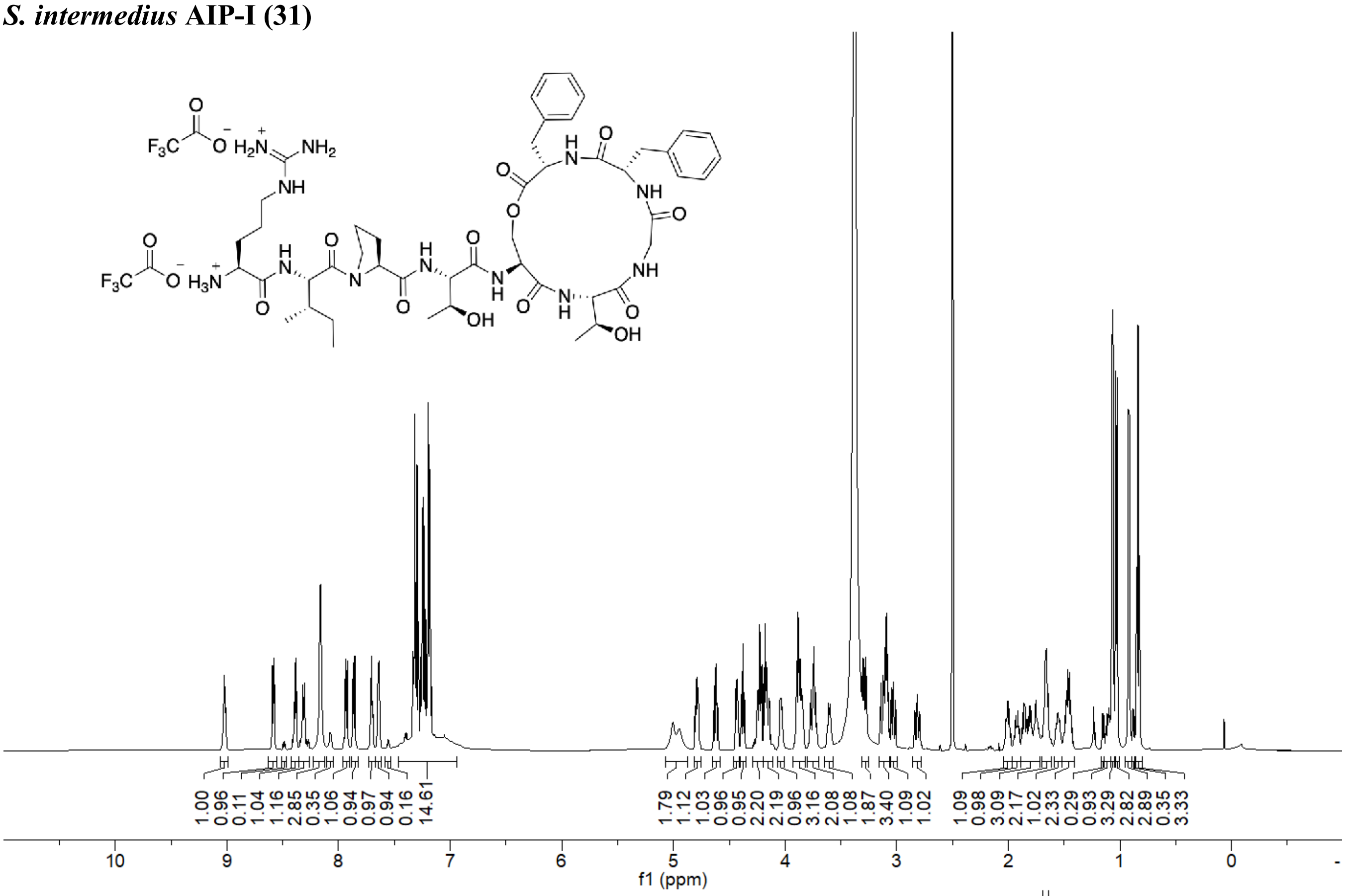

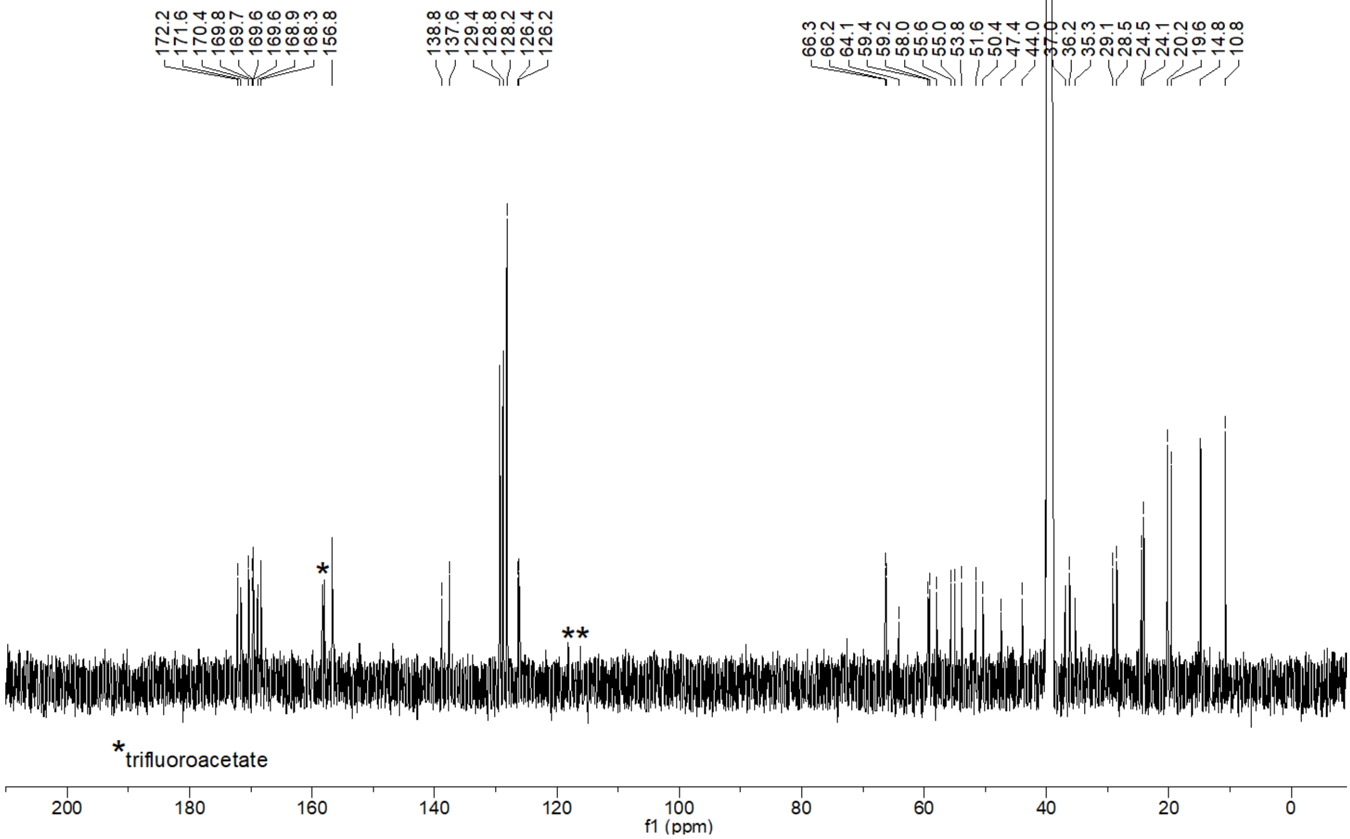

***S. simulans* AIP-II (33)**

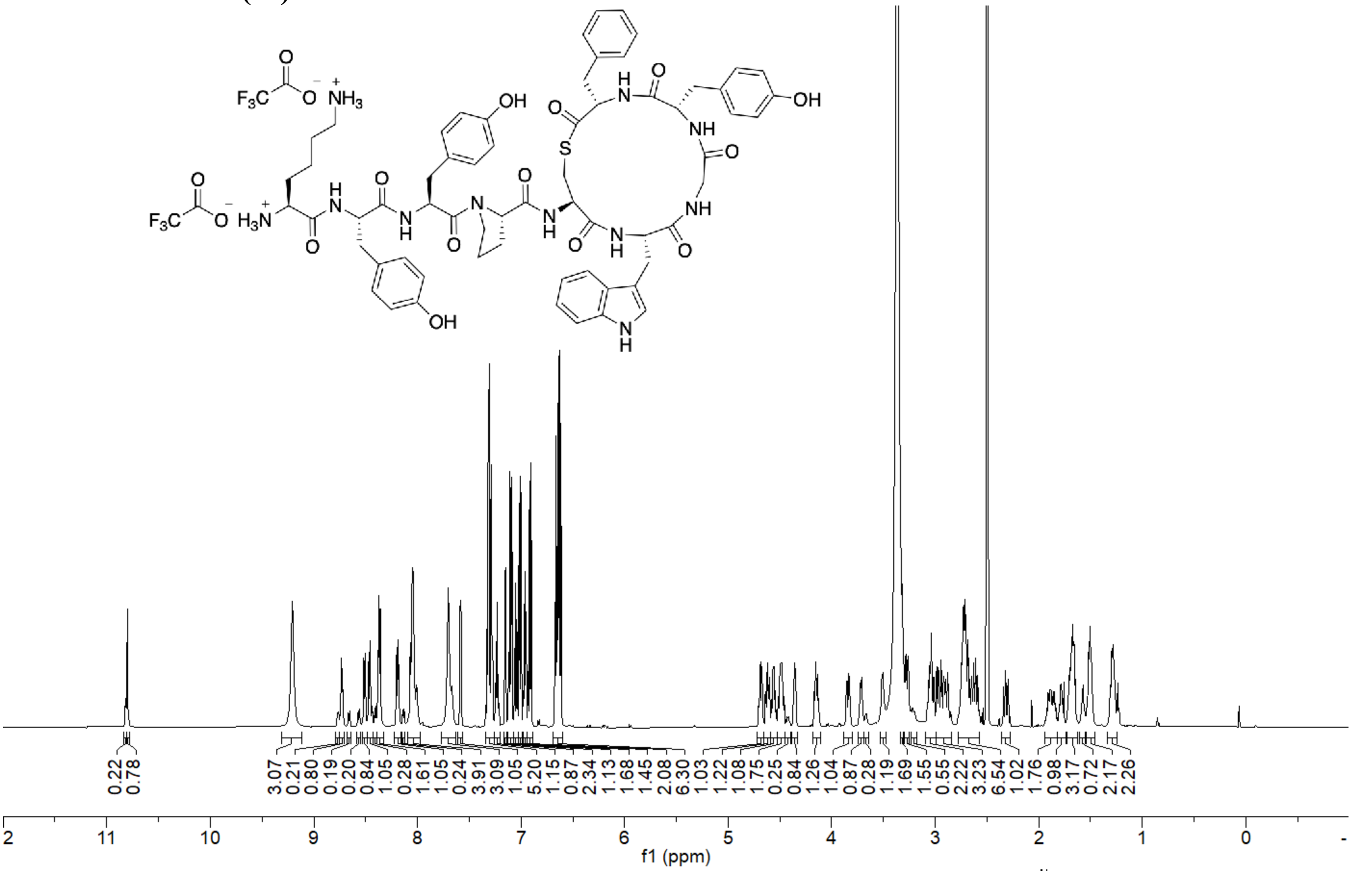

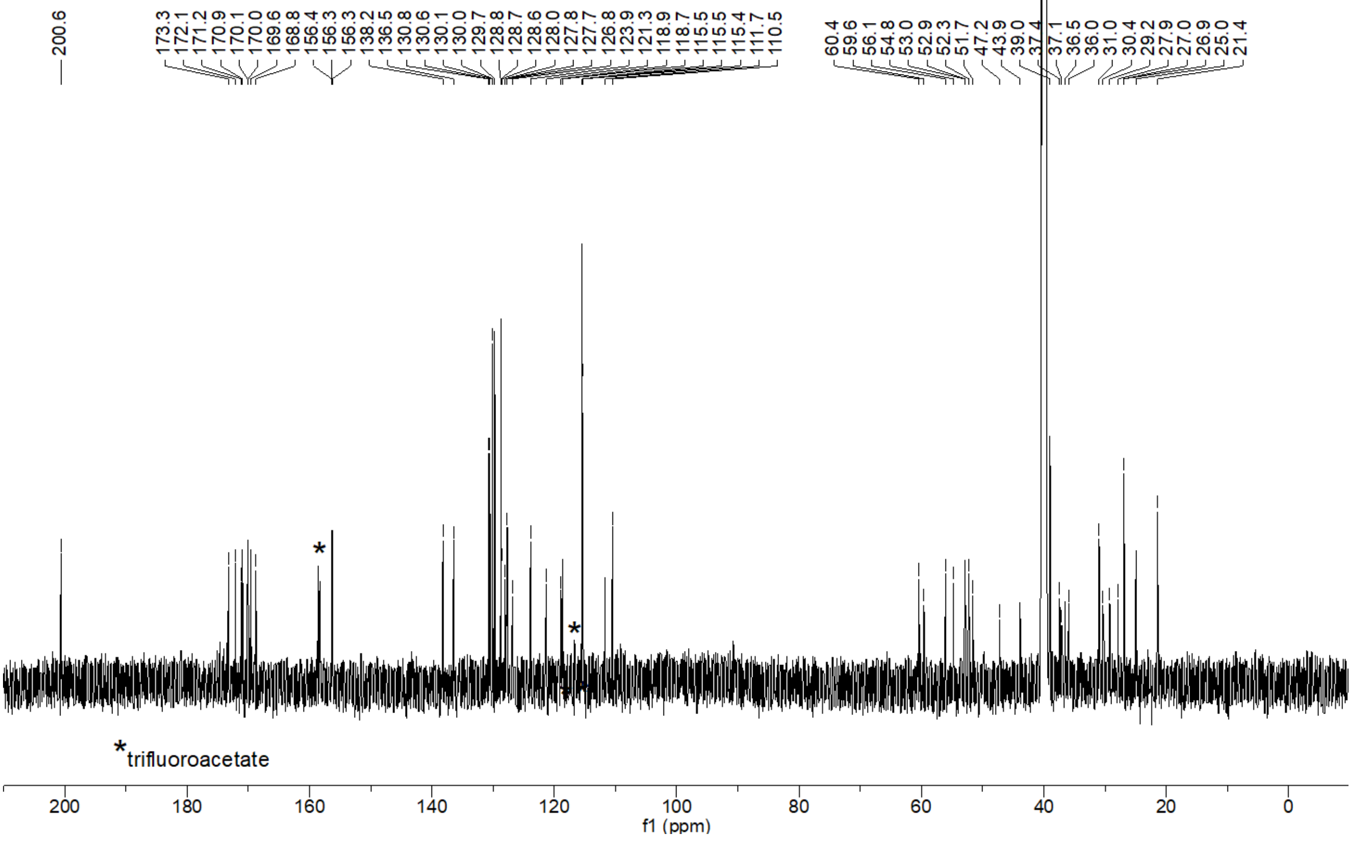

S. simulans **AIP-III (34)**

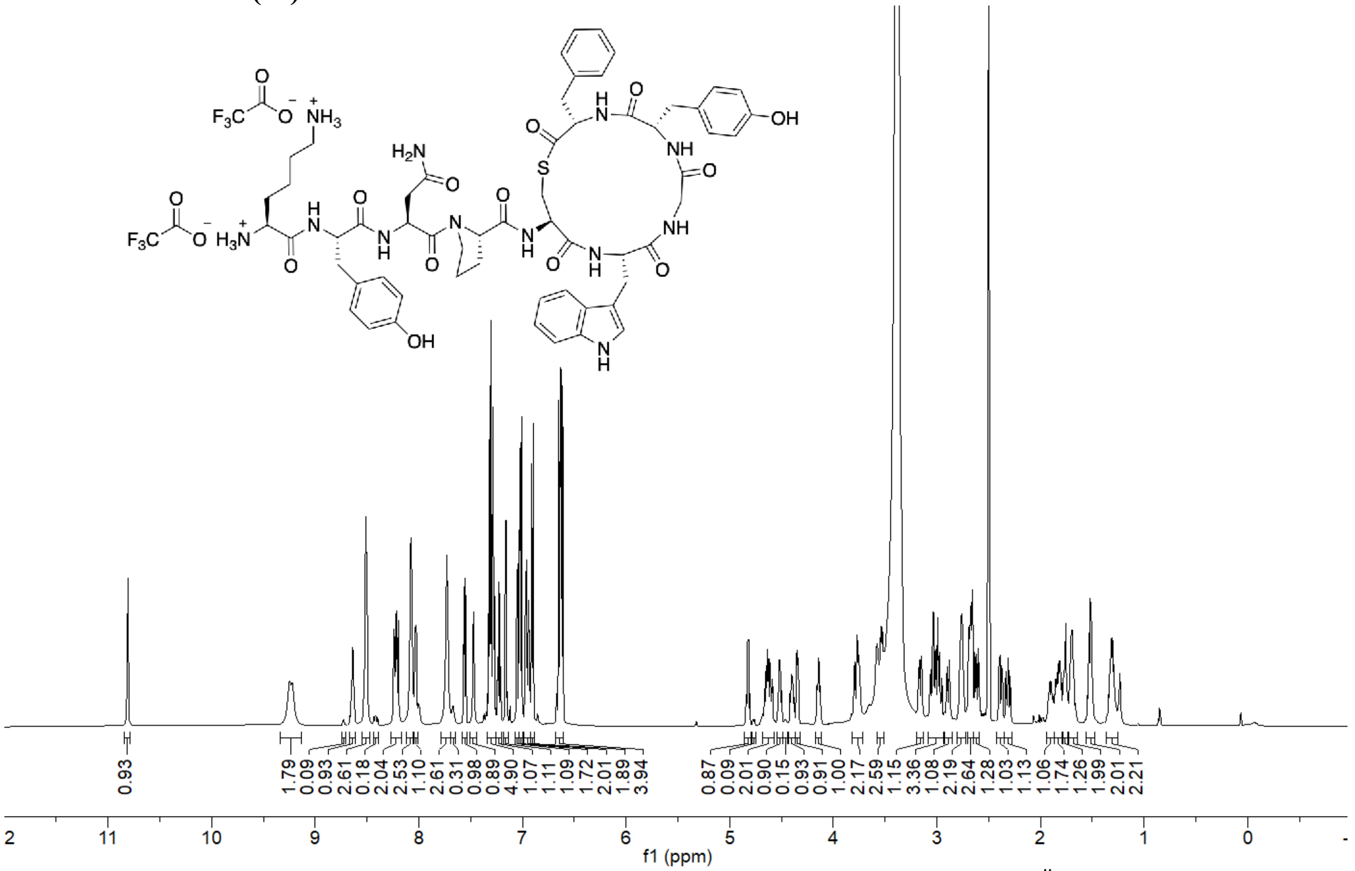

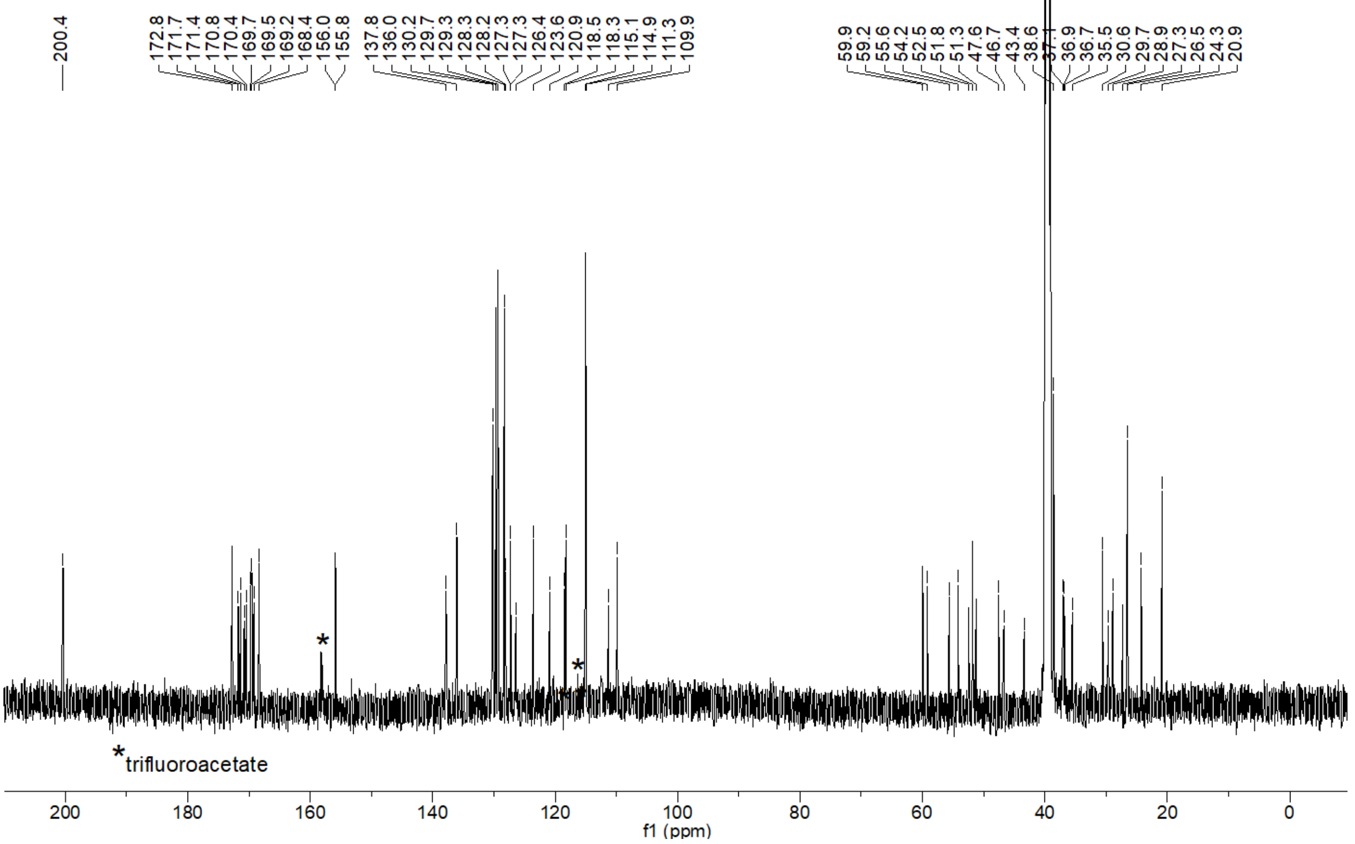

Supplement: Supplemental Characterization — NMR spectra and chromatograms. [file mbio.00967-25-s0001.pdf]
