# Supplementary material for: Mapping of quorum sensing interaction network of commensal and pathogenic staphylococci
Source: mBio. 2025 Jul 16;16(8):e00967-25. doi: 10.1128/mbio.00967-25 (PMC12345137; doi:10.1128/mbio.00967-25)
Supplement: Supplemental Material — Additional figures, schemes, and tables as well as compound characterization data. [file mbio.00967-25-s0002.pdf]

Supplemental Information

Mapping of quorum sensing interaction network of commensal and pathogenic staphylococci

Bengt H. Gless,[1,#,*] Benjamin S. Sereika-Bejder,[1,#], Iben Jensen,[1] Martin S. Bojer,[2] Katerina Tsiko,[1] Sabrina H. Schmied,[1] Ludovica Vitolo,[1] Bruno Toledo-Silva,[3] Sarne De Vliegher,[3] Hanne Ingmer,[2] and Christian A. Olsen[1,*]

[1]*Center for Biopharmaceuticals and Department of Drug Design and Pharmacology, Faculty of Health and Medical Sciences, University of Copenhagen, Jagtvej 160, DK-2100, Copenhagen, Denmark.*
[2]*Department of Veterinary and Animal Sciences, Faculty of Health and Medical Sciences, University of Copenhagen, Stigbøjlen 4, DK-1870 Frederiksberg C, Denmark.*
[3]*M-team & Mastitis and Milk Quality Research Unit, Department of Internal Medicine, Reproduction and Population Medicine, Faculty of Veterinary Medicine, Ghent University, Salisburylaan 133, B-9820 Merelbeke, Belgium*
[#]*These authors contributed equally to the work*

*Correspondence should be addressed to: bengt.gless@gmail.com or cao@sund.ku.dk

Table of Contents

1. Supplementary figures 3
The staphylococcal *accessory gene regulator* (*agr*) system 3
Native chemical ligation (NCL) trapping of autoinducing peptides (AIPs) 4
LC-MS traces of sequence-guided identifications of AIPs 5
Bar graphs for *agr* interference using fluorescence reporter assays of native AIPs 11
Dose-response curves for *agr* interference for heat map validation 18
Dose-response curves for AgrC interference using β-lactamase reporter assays 21
Overnight growth and fluorescence curves in presence of synthetic AIPs 25
Dose-response curves for *agr* interference of *S. simulans* AIPs 27
Bar graphs for *agr* interference of *S. simulans* AIPs SAR study 29
Overnight growth and fluorescence curves of *spa*-GFP agr deactivation assay 34
2. Supplementary schemes 35
Synthesis of thiolactone-containing AIPs 35
Synthesis of *S. intermedius* AIP 36
Synthesis of *S simulans* AIP-II lactam 36
3. Supplementary tables 37
Staphylococci species and sequences of known AIPs. 37
List of recorded and reported quorum sensing interactions of staphylococcal AIPs 38
MRSA mouse skin infection model data 42
Bacterial strains and *agrD* sequencing 43
4. Methods and protocols 44
4.1 General information 44
4.2 Synthetic procedures 45
5. Synthetic peptides 47
5.1 Native AIPs 47
5.2 *S. simulans* AIP SAR study 58
6. Supplementary references 66

1. Supplementary figures

The staphylococcal *accessory gene regulator* (*agr*) system

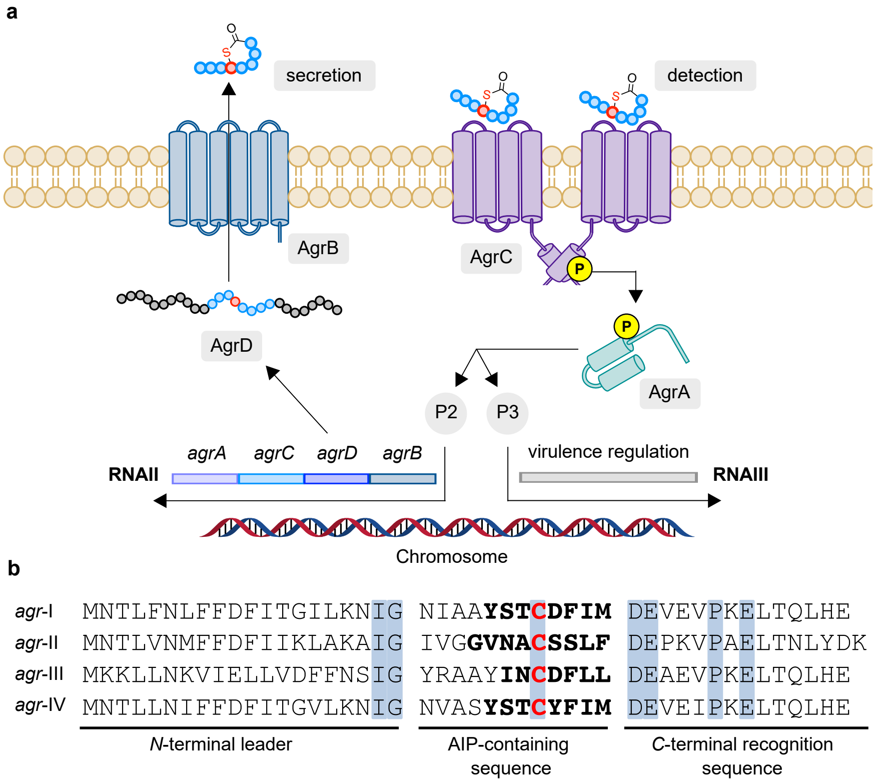

Supplementary Figure S1. The staphylococcal *agr* system. a, The chromosomal *agr* locus consists of the two promoter regions P2 and P3, which initiate the transcription of RNAII and RNAIII, respectively. RNAII encodes the four protein components (AgrA, AgrB, AgrC, and AgrD) of the *agr* system, while RNAIII is a regulatory RNA controlling the expression of virulence factors. In the first step of the *agr* circuit, the AIP-precursor peptide AgrD (44–46 amino acids for *S. aureus*) is processed by the membrane-embedded endopeptidase AgrB installing the thiolactone functionality. The peptide is further translocated to the extracellular space and finally cleaved to release the mature AIP. Once a concentration threshold of the AIP molecules is reached due to increased cell density, the AgrC receptor, a homodimeric membrane-bound histidine kinase, is activated. AIP-induced activation is followed by auto-phosphorylation of AgrC and subsequent phosphoryl transfer to AgrA, the response regulator of the *agr* system, making the AgrC–AgrA interaction a classical two-component regulatory system. Phosphorylated AgrA binds to the promotors P2 and P3, resulting in upregulated transcription of RNAII and RNAIII, which lead to a positive feedback loop for AgrBDCA expression as well as upregulated expression of virulence factors. **b**, AgrD peptides consist of three domains, the *C*-terminal recognition sequence, the *N*-terminal leader peptide and in between the 12 amino acid long AIP-containing sequence. Conserved residues are highlighted in blue boxes.

Native chemical ligation (NCL) trapping of autoinducing peptides (AIPs)

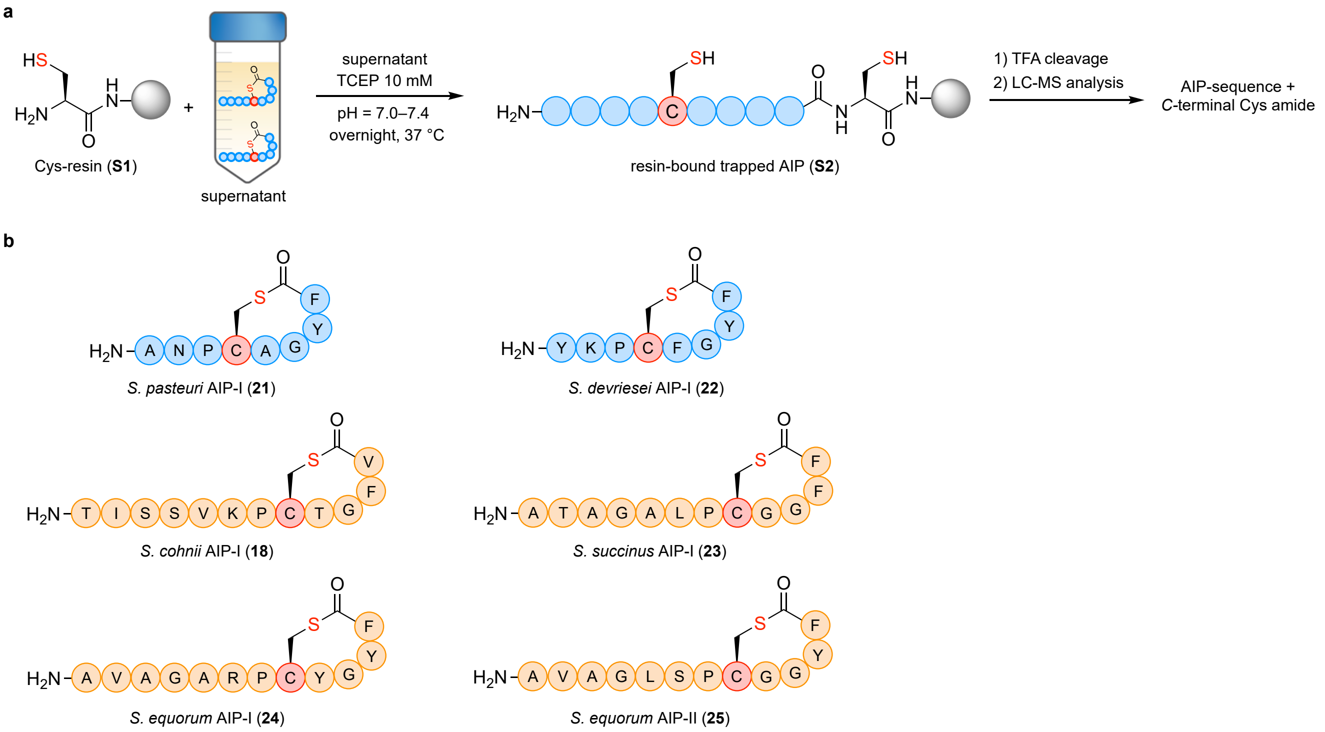

Supplementary Figure S2. Identification of AIPs through NCL trapping. a, Cys-resin (**S1**) is incubated overnight in pH-adjusted bacterial supernatant containing tris(2-carboxyethyl)phosphine (TCEP, 10 mM) enabling chemoselective trapping of AIPs. The resin with the trapped AIP (**S2**) is washed extensively and subsequently treated with trifluoroacetic acid (TFA) to release the trapped AIP. The concentrated cleavage solution is analyzed by liquid-chromatography mass-spectrometry (LC-MS) for the 7 possible AIPs sequences with an additional *C*-terminal Cys amide extracted from the AIP-precursor peptide AgrD. **b**, Newly identified AIPs in this study.

LC-MS traces of sequence-guided identifications of AIPs

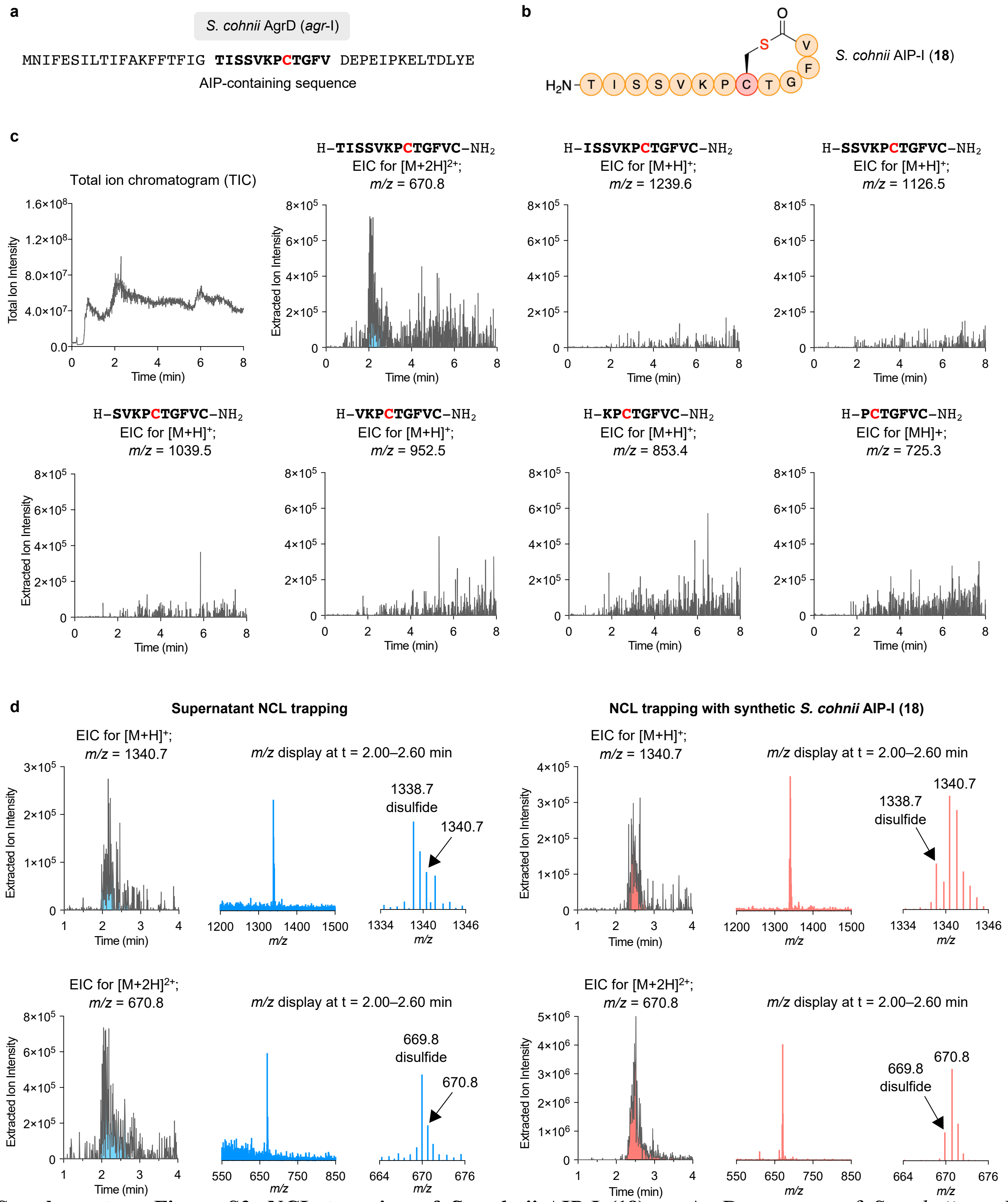

Supplementary Figure S3. NCL trapping of *S. cohnii* AIP-I (18). **a**, AgrD sequence of *S. cohnii agr*-I. **b**, Structure of *S. cohnii* AIP-I (**18**). **c**, LC-MS analysis of the TFA cleavage solution: TIC and EICs of *m/z* = $[M+H]^+$ of possible linear AIP sequences including a C-terminal cysteine amide. **d**, NCL trapping of synthetic *S. cohnii* AIP-I (**18**) confirms the identity of trapped AIP **18** from bacterial supernatant.

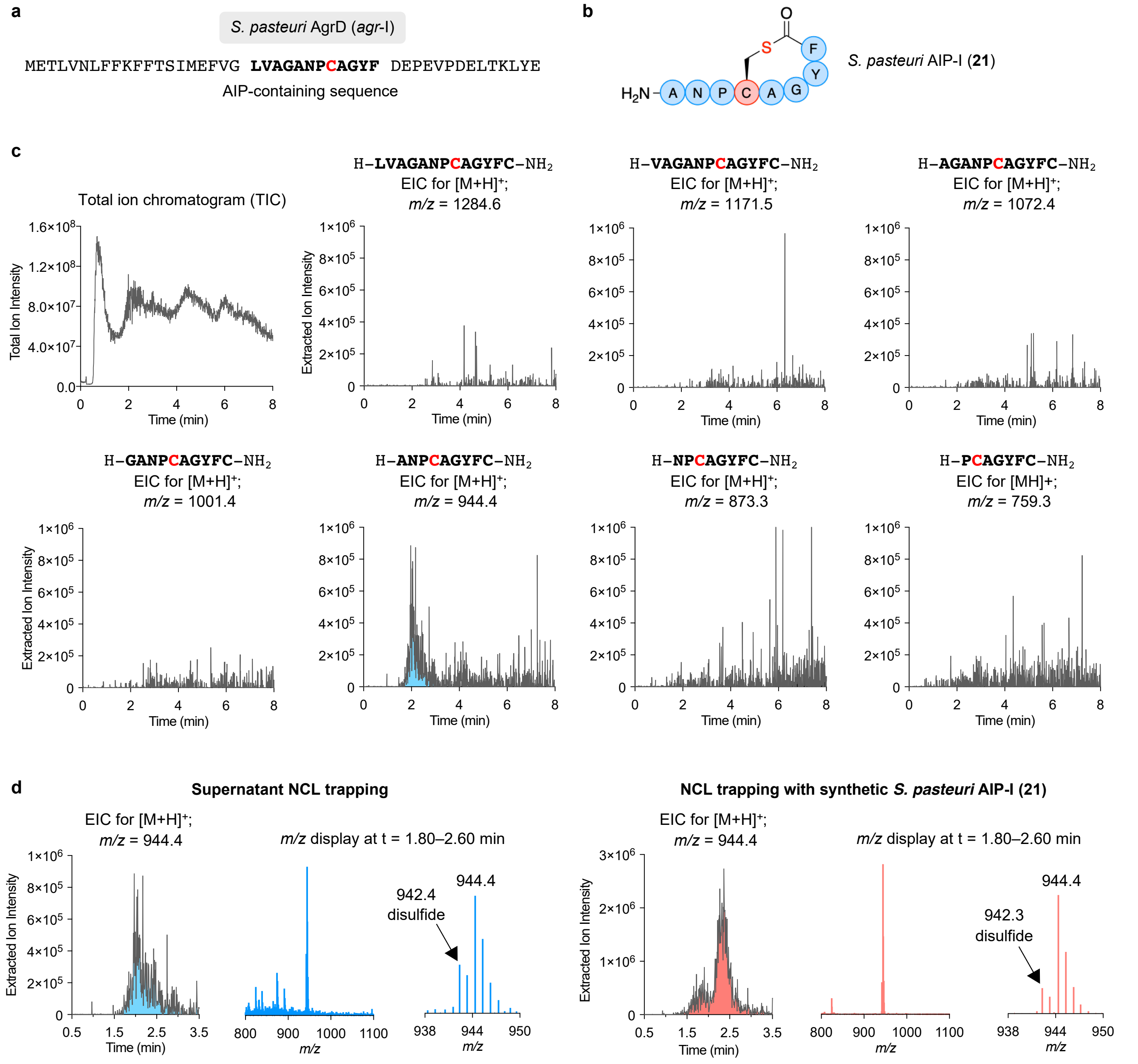

Supplementary Figure S4. NCL trapping of *S. pasteuri* AIP-I (21). **a**, AgrD sequence of *S. pasteuri agr*-I. **b**, Structure of *S. pasteuri* AIP-I (**21**). **c**, LC-MS analysis of the TFA cleavage solution: TIC and EICs of $m/z = [M+H]^+$ of possible linear AIP sequences including a C-terminal cysteine amide. **d**, NCL trapping of synthetic *S. pasteuri* AIP-I (**21**) confirms the identity of trapped AIP **21** from bacterial supernatant.

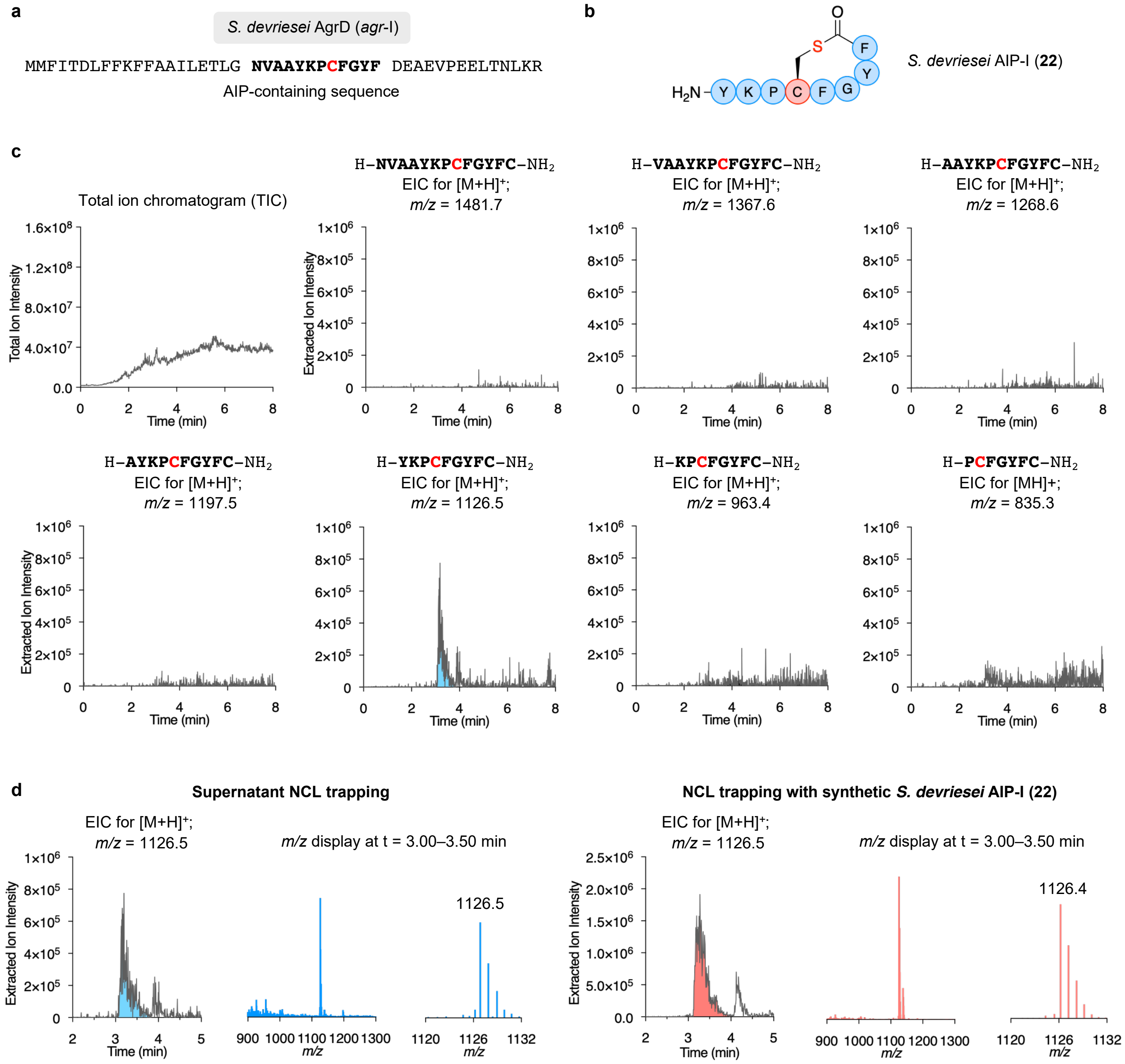

Supplementary Figure S5. NCL trapping of *S. devriesei* AIP-I (22). a, AgrD sequence of *S. devriesei agr*-I. **b**, Structure of *S. devriesei* AIP-I (**22**). **c**, LC-MS analysis of the TFA cleavage solution: TIC and EICs of m/z = $[M+H]^+$ of possible linear AIP sequences including a C-terminal cysteine amide. **d**, NCL trapping of synthetic *S. devriesei* AIP-I (**22**) confirms the identity of trapped AIP **22** from bacterial supernatant.

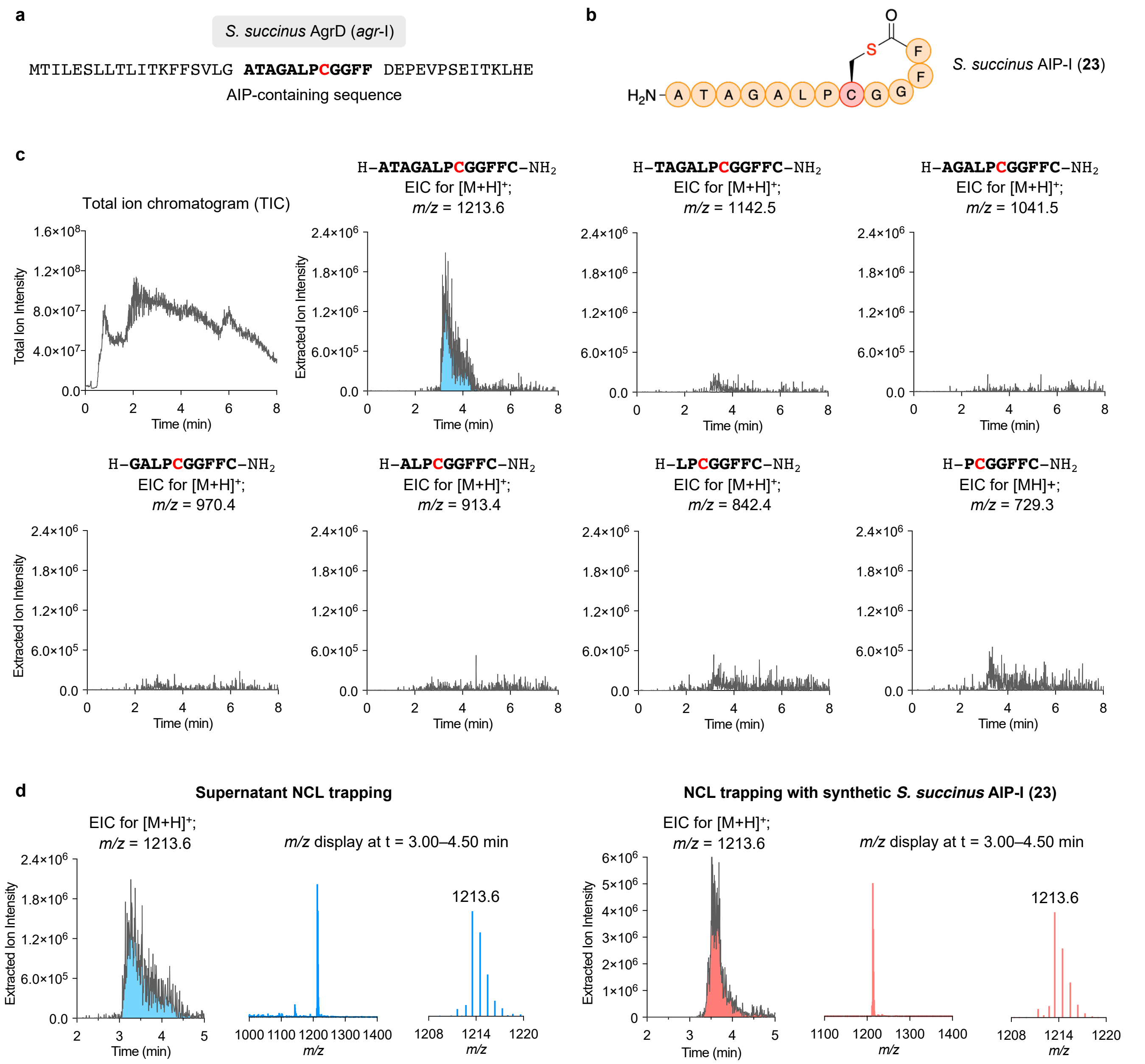

Supplementary Figure S6. NCL trapping of *S. succinus* AIP-I (23). **a**, AgrD sequence of *S. succinus agr*-I. **b**, Structure of *S. succinus* AIP-I (**23**). **c**, LC-MS analysis of the TFA cleavage solution: TIC and EICs of m/z = $[M+H]^+$ of possible linear AIP sequences including a C-terminal cysteine amide. **d**, NCL trapping of synthetic *S. succinus* AIP-I (**23**) confirms the identity of trapped AIP **23** from bacterial supernatant.

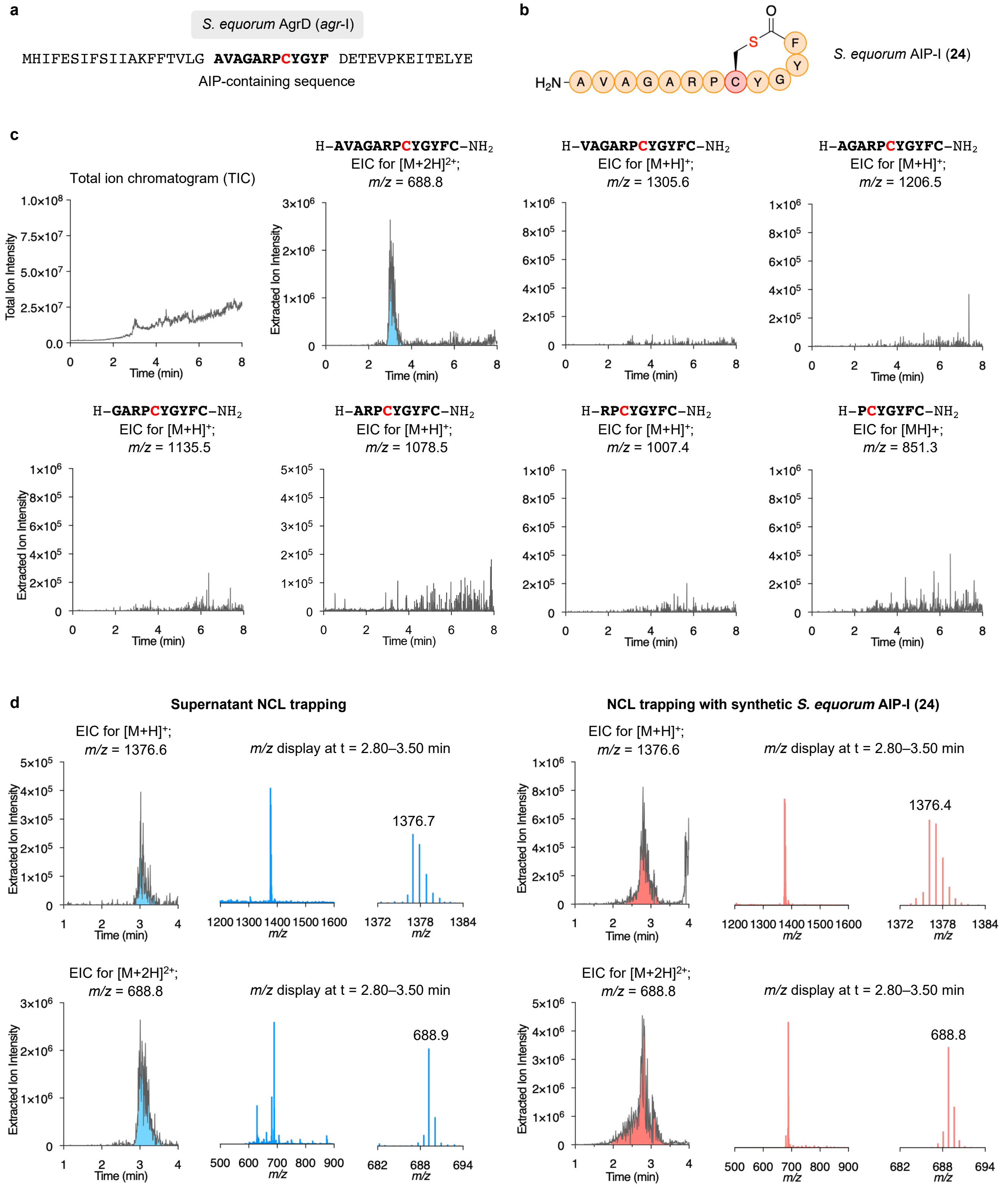

Supplementary Figure S7. NCL trapping of *S. equorum* AIP-I (24). **a**, AgrD sequence of *S. equorum agr*-I. **b**, Structure of *S. equorum* AIP-I (**24**). **c**, LC-MS analysis of the TFA cleavage solution: TIC and EICs of m/z = [M+H]$^+$ of possible linear AIP sequences including a C-terminal cysteine amide. **d**, NCL trapping of synthetic *S. equorum* AIP-I (**24**) confirms the identity of trapped AIP **24** from bacterial supernatant.

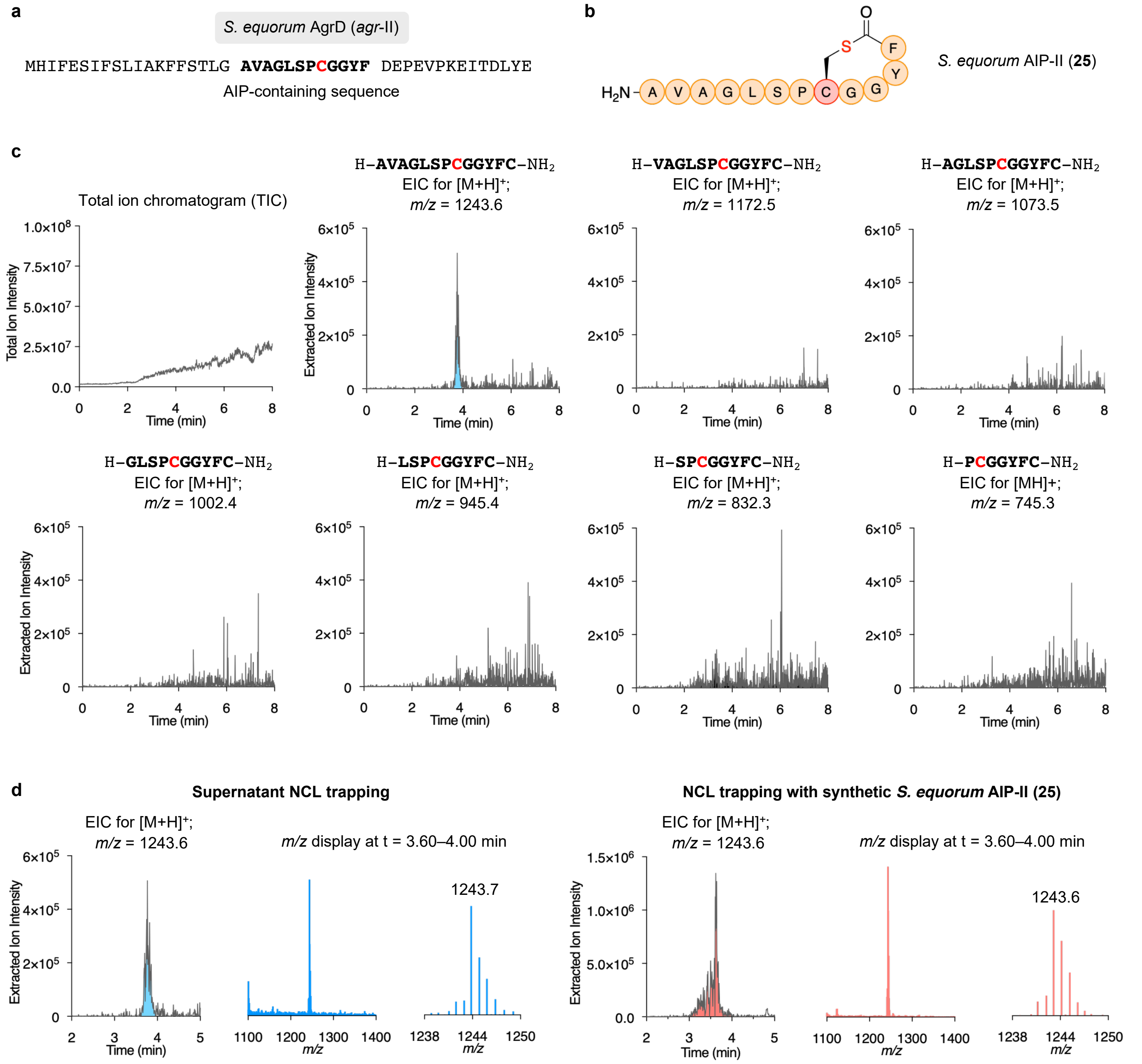

Supplementary Figure S8. NCL trapping of *S. equorum* AIP-II (25). **a**, AgrD sequence of *S. equorum agr*-II. **b**, Structure of *S. equorum* AIP-II (**25**). **c**, LC-MS analysis of the TFA cleavage solution: TIC and EICs of *m/z* = $[M+H]^+$ of possible linear AIP sequences including a C-terminal cysteine amide. **d**, NCL trapping of synthetic *S. equorum* AIP-II (**25**) confirms the identity of trapped AIP **25** from bacterial supernatant.

Bar graphs for *agr* interference using fluorescence reporter assays of native AIPs

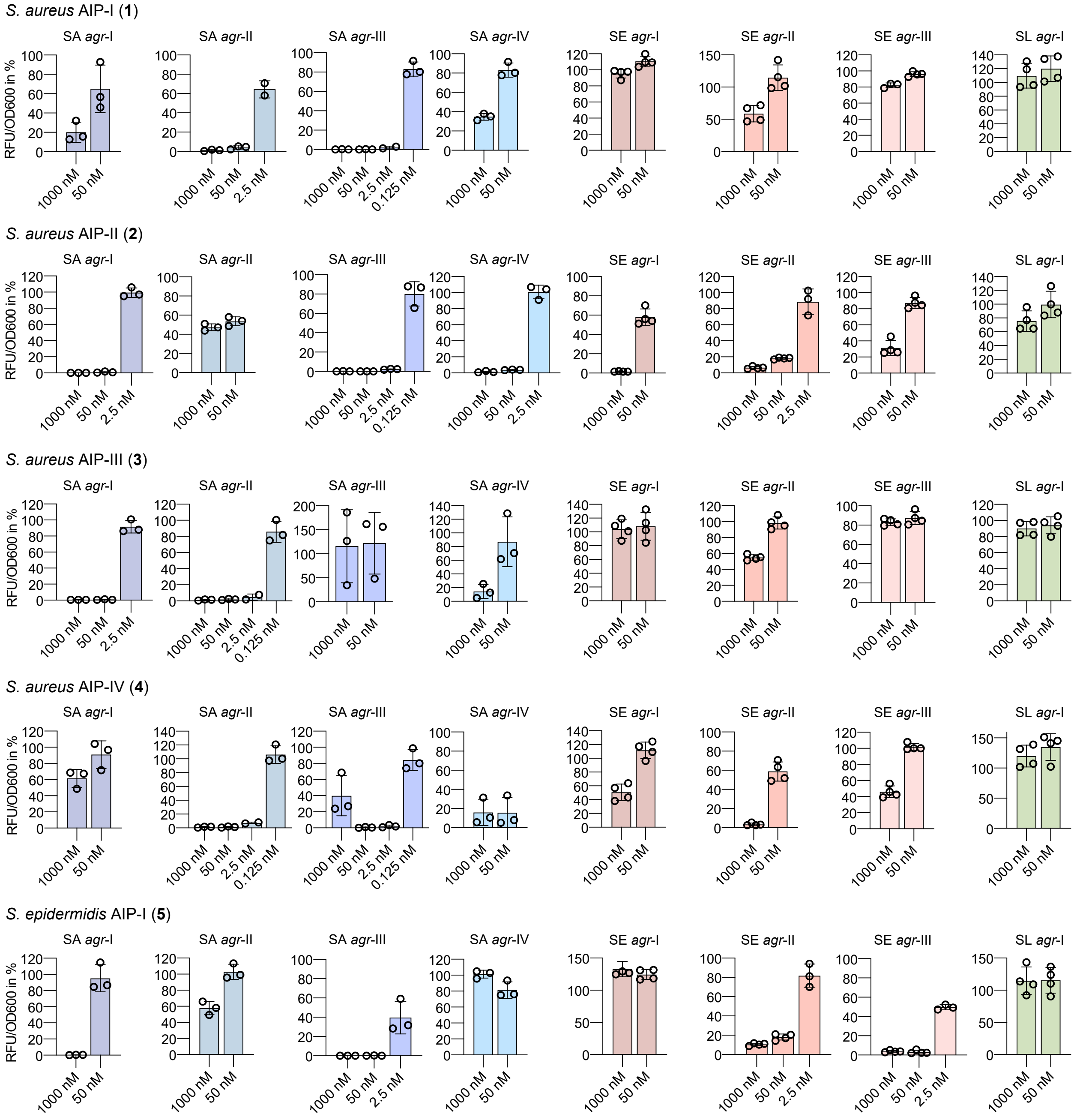

Supplementary Figure S9. Fluorescence reporter strain assay for *agr* interference with native AIPs. Fluorescent reporter strains of *S. aureus* (SA), *S. epidermidis* (SE), S. *lugdunensis* (SL) were treated with AIPs at 1000 nM and 50 nM. AIPs were further tested at 2.5 nM and 0.125 nM in case >75% inhibition was observed at higher concentrations. Error bars are the standard error of the mean (SEM) of at least two individual biological assays performed in technical triplicate.

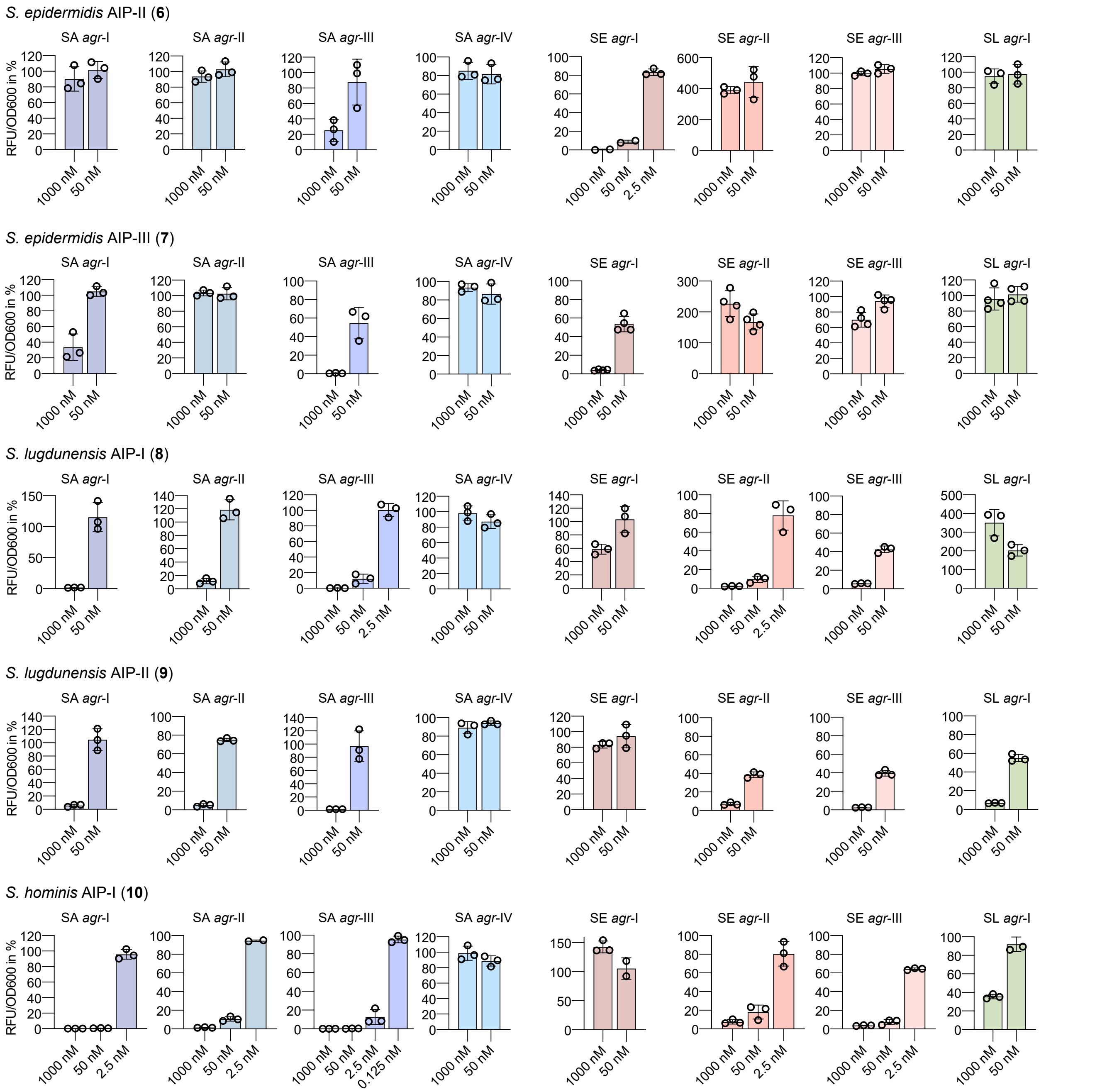

Supplementary Figure S10. Fluorescence reporter strain assay for *agr* interference with with native AIPs. Fluorescent reporter strains of of *S. aureus* (SA), *S. epidermidis* (SE), S. *lugdunensis* (SL) were treated with AIPs at 1000 nM and 50 nM. AIPs were further tested at 2.5 nM and 0.125 nM in case >75% inhibition was observed at higher concentrations. Error bars are the SEM of at least two individual biological assays performed in technical triplicate.

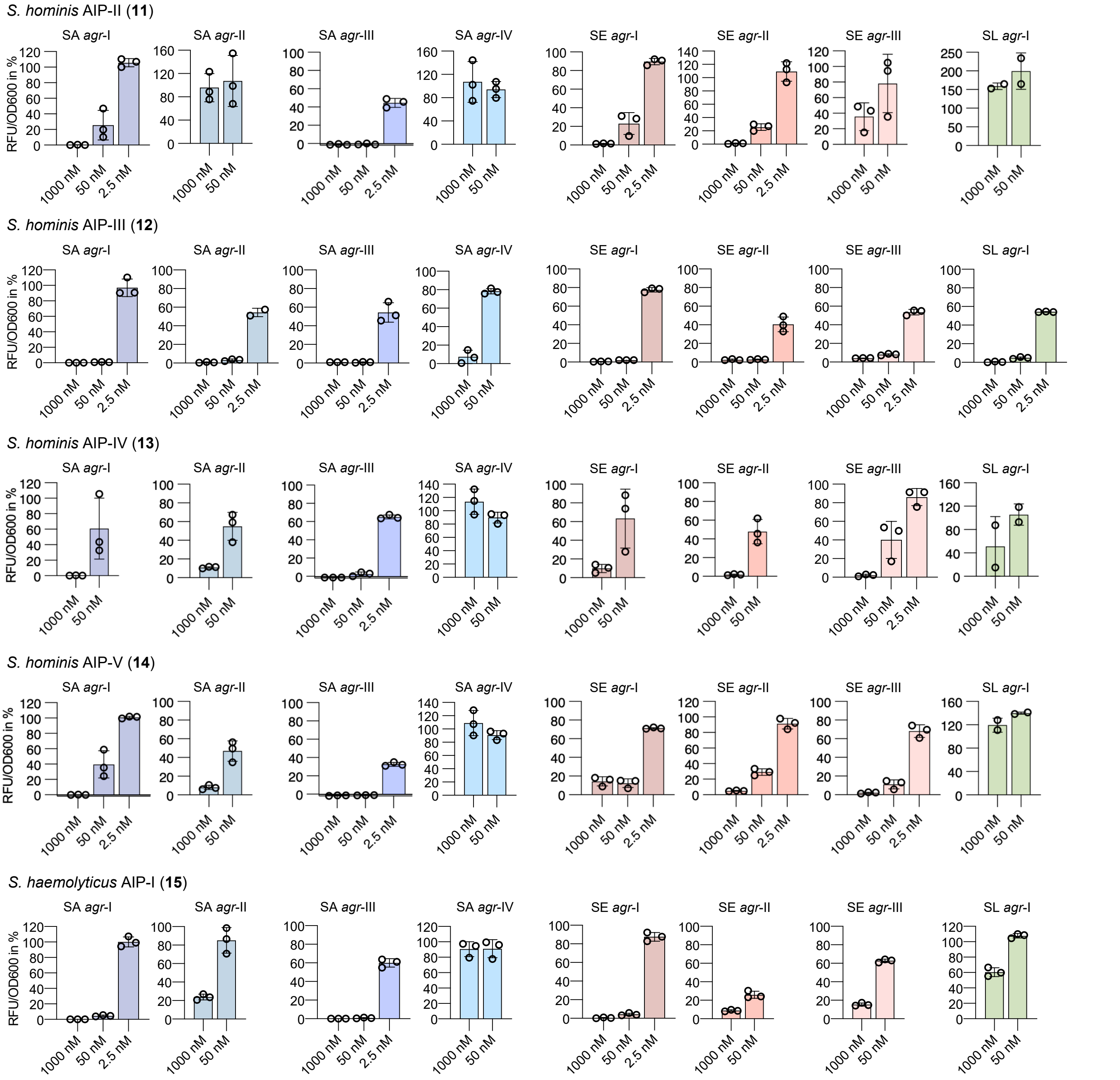

Supplementary Figure S11. Fluorescence reporter strain assay for *agr* interference with native AIPs. Fluorescent reporter strains of of *S. aureus* (SA), *S. epidermidis* (SE), S. *lugdunensis* (SL) were treated with AIPs at 1000 nM and 50 nM. AIPs were further tested at 2.5 nM and 0.125 nM in case >75% inhibition was observed at higher concentrations. Error bars are the SEM of at least two individual biological assays performed in technical triplicate.

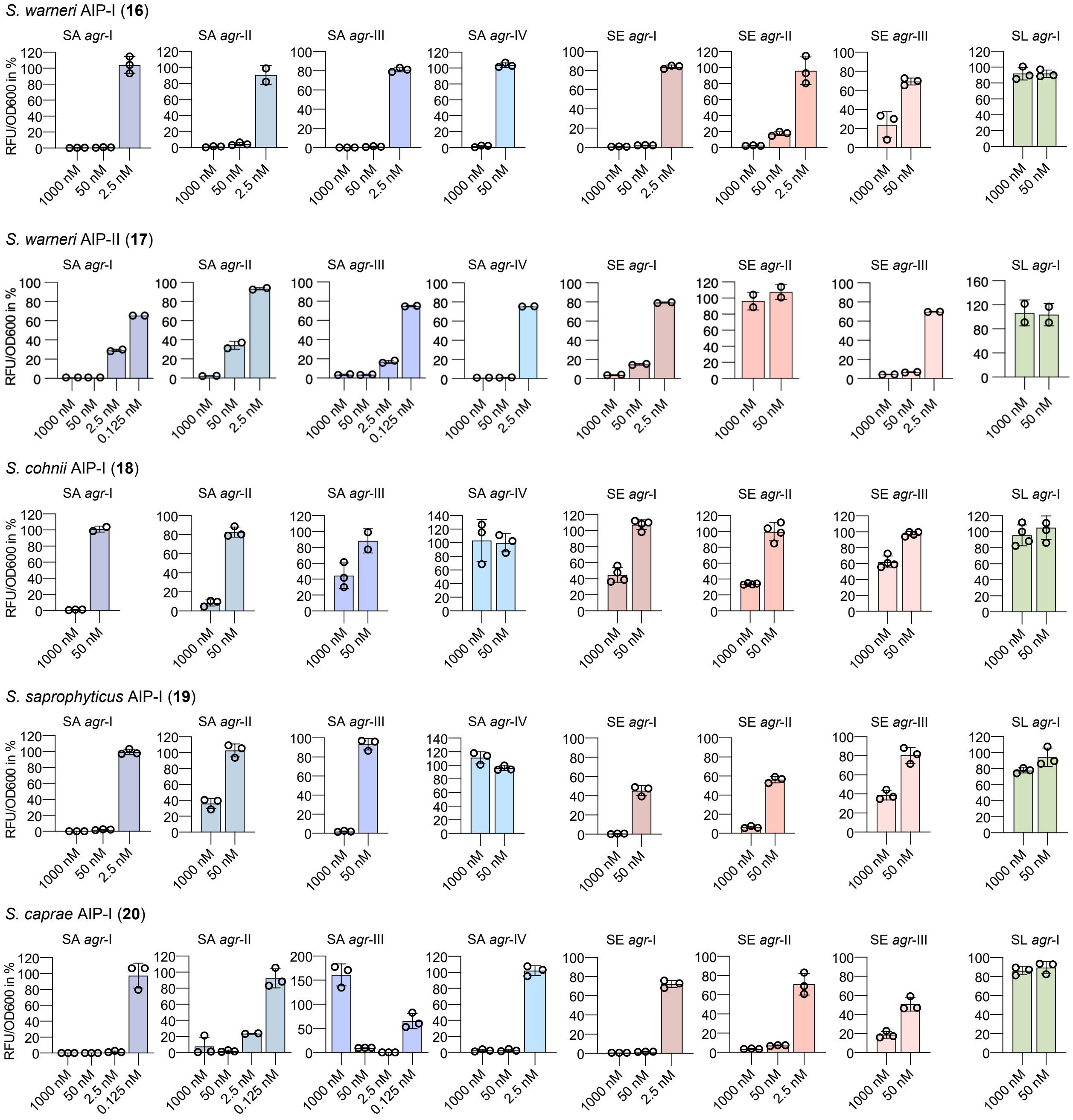

Supplementary Figure S12. Fluorescence reporter strain assay for *agr* interference with native AIPs. Fluorescent reporter strains of of *S. aureus* (SA), *S. epidermidis* (SE), S. *lugdunensis* (SL) were treated with AIPs at 1000 nM and 50 nM. AIPs were further tested at 2.5 nM and 0.125 nM in case >75% inhibition was observed at higher concentrations. Error bars are the SEM of at least two individual biological assays performed in technical triplicate.

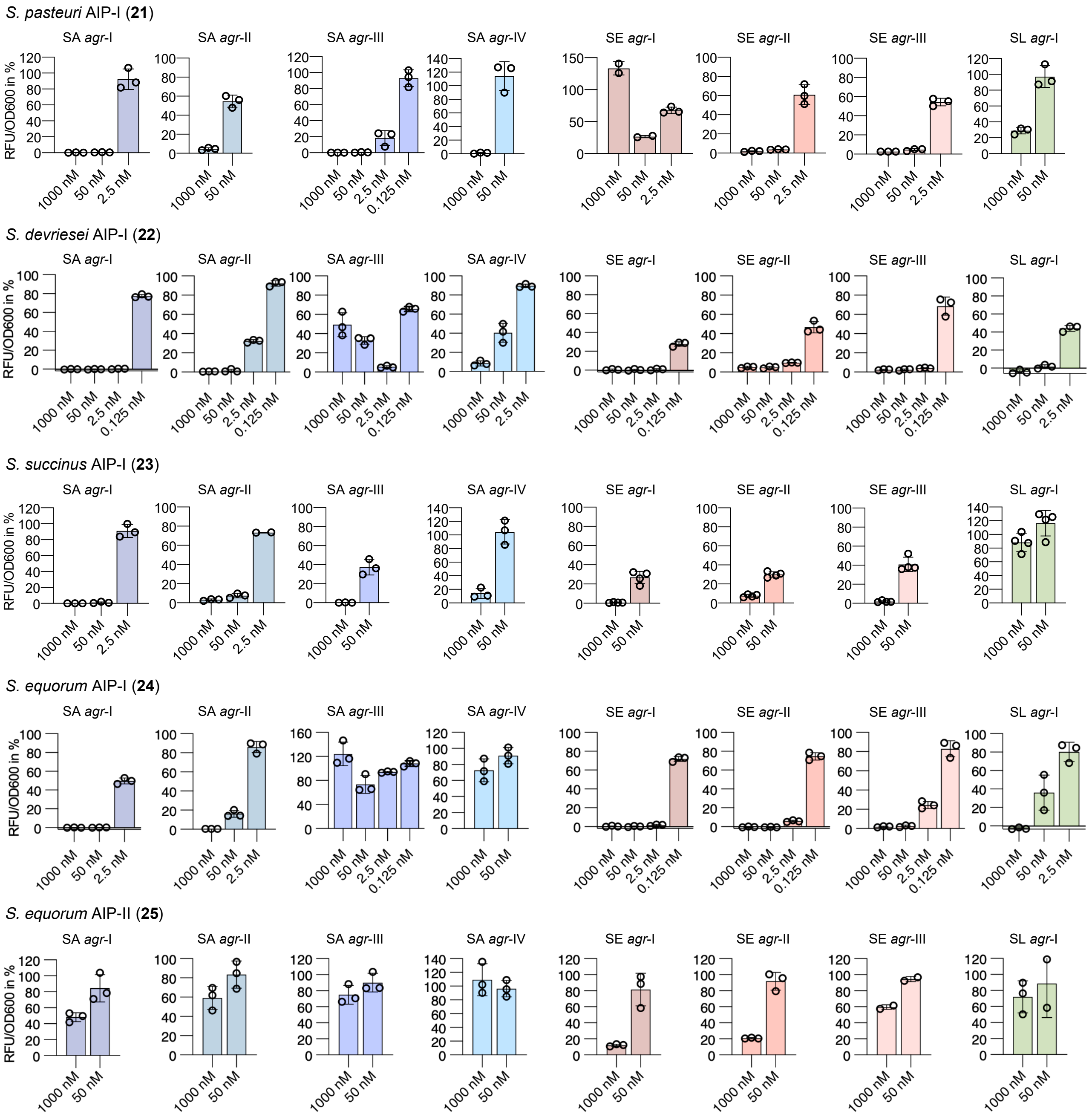

Supplementary Figure S13. Fluorescence reporter strain assay for *agr* interference with native AIPs. Fluorescent reporter strains of of *S. aureus* (SA), *S. epidermidis* (SE), S. *lugdunensis* (SL) were treated with AIPs at 1000 nM and 50 nM. AIPs were further tested at 2.5 nM and 0.125 nM in case >75% inhibition was observed at higher concentrations. Error bars are the SEM of at least two individual biological assays performed in technical triplicate.

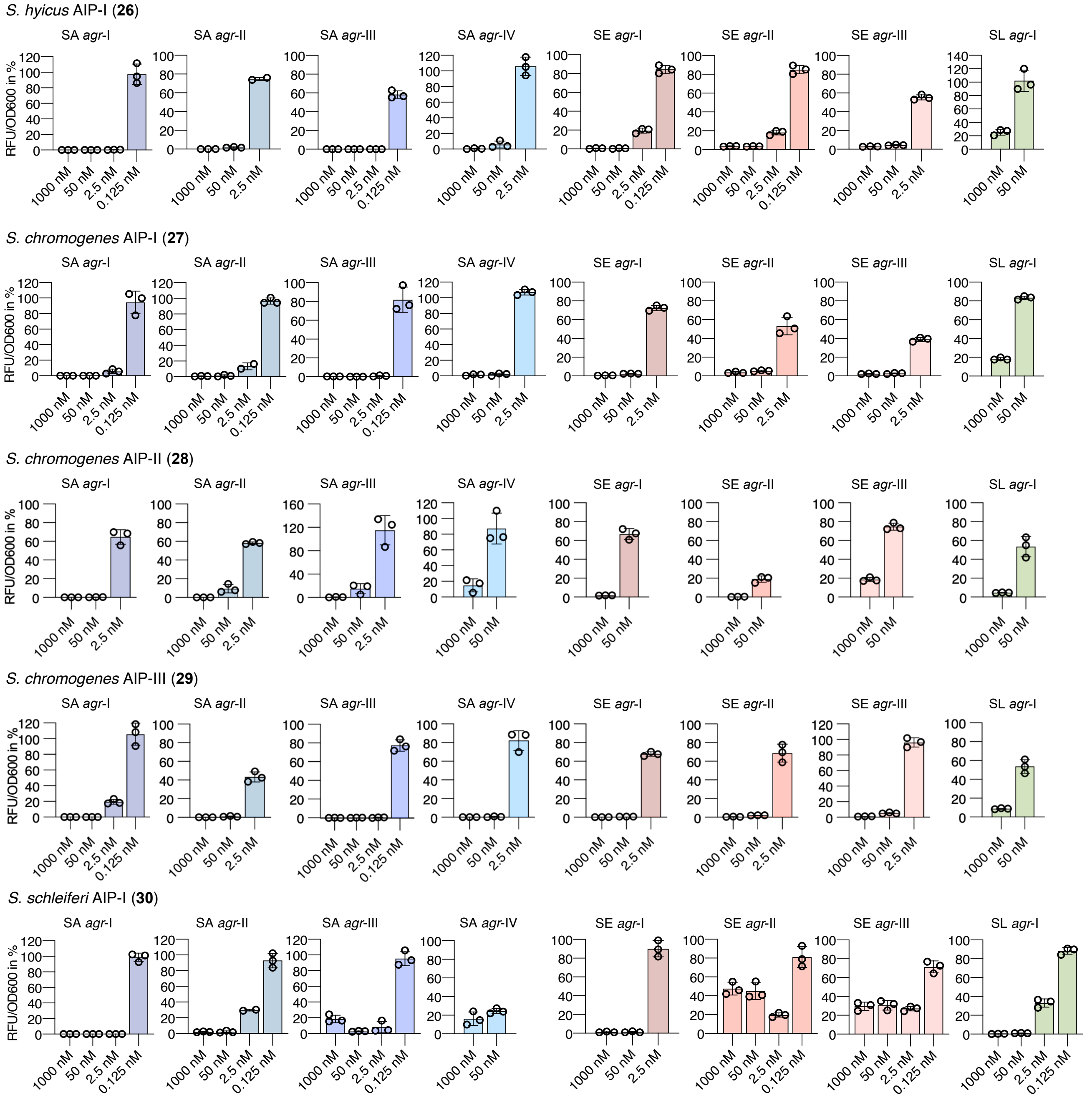

Supplementary Figure S14. Fluorescence reporter strain assay for *agr* interference with native AIPs. Fluorescent reporter strains of of *S. aureus* (SA), *S. epidermidis* (SE), S. *lugdunensis* (SL) were treated with AIPs at 1000 nM and 50 nM. AIPs were further tested at 2.5 nM and 0.125 nM in case >75% inhibition was observed at higher concentrations. Error bars are the SEM of at least two individual biological assays performed in technical triplicate.

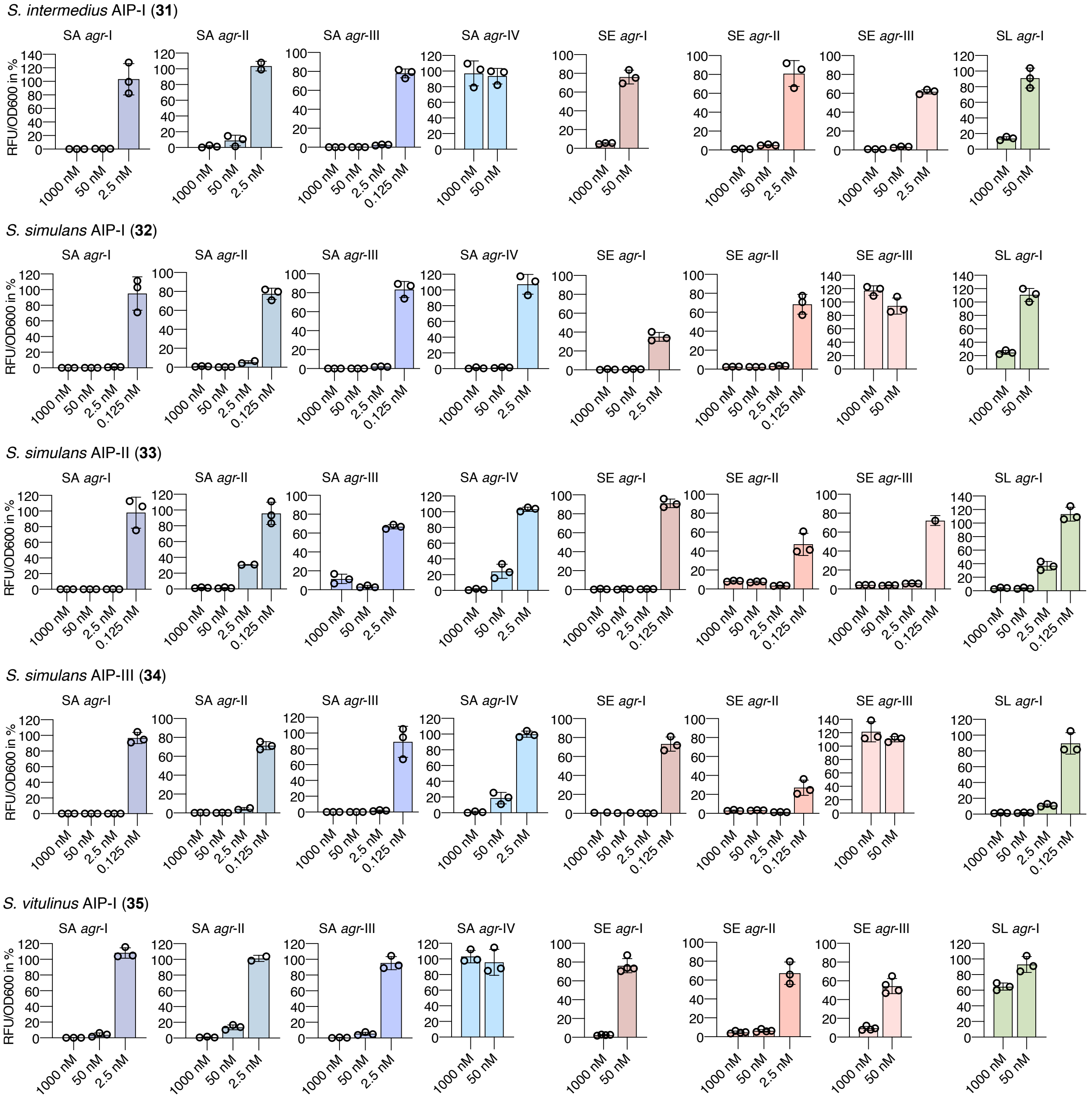

Supplementary Figure S15. Fluorescence reporter strain assay for *agr* interference with native AIPs. Fluorescent reporter strains of of *S. aureus* (SA), *S. epidermidis* (SE), S. *lugdunensis* (SL) were treated with AIPs at 1000 nM and 50 nM. AIPs were further tested at 2.5 nM and 0.125 nM in case >75% inhibition was observed at higher concentrations. Error bars are the SEM of at least two individual biological assays performed in technical triplicate.

Dose-response curves for *agr* interference for heat map validation

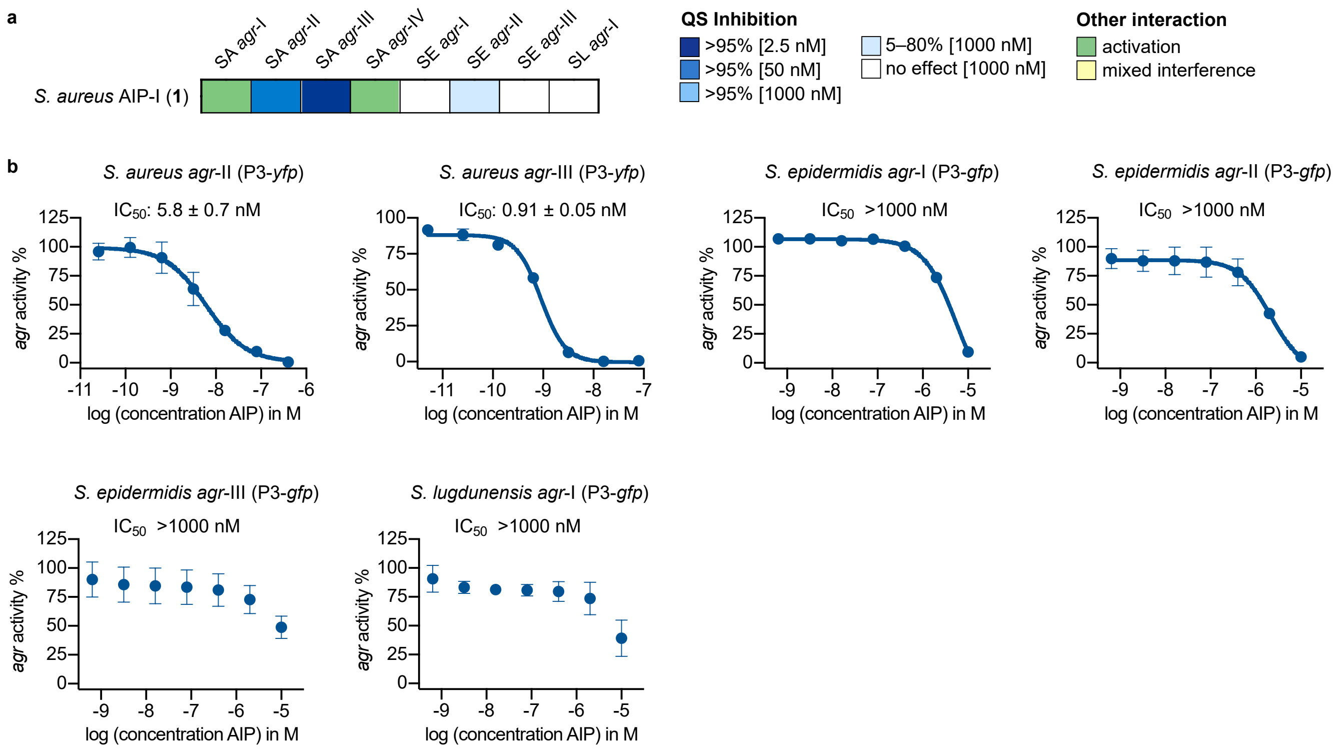

Supplementary Figure S16. Heat map assay validation for *S. aureus* AIP-I (1) through full dose-response curves. a, Heat map data of *S. aureus* AIP-I (**1**). **b**, IC50 values of *S. aureus* AIP-I (**1**) against reporter strains of *S. aureus* (SA), *S. epidermidis* (SE), *S. lugdunensis* (SL) were determined through fluorescence readout as a measure of agr activity. The curves were generated from three individual assays performed in technical duplicate and shown error bars are the standard deviation of the mean (SD).

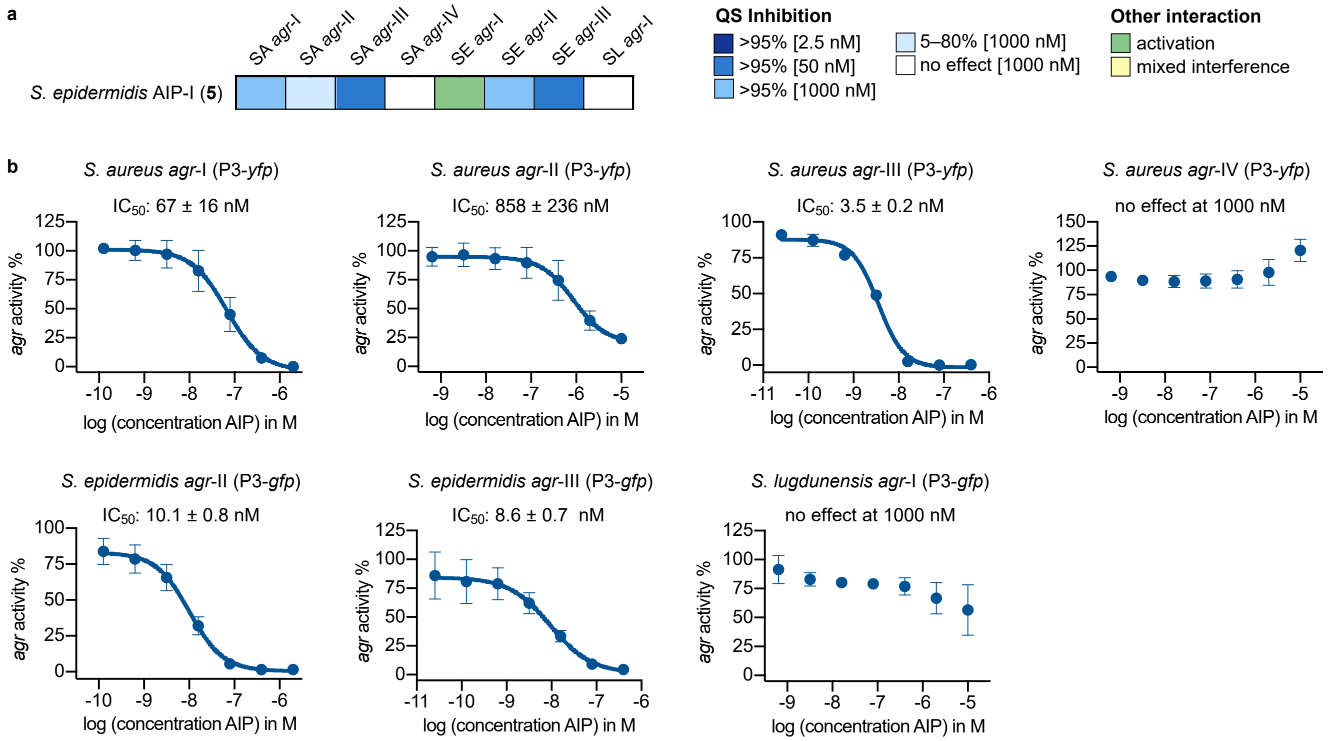

Supplementary Figure S17. Heat map assay validation for *S. epidermidis* AIP-I (5) through full dose-response curves. **a**, Heat map data of *S. epidermidis* AIP-I (**5**). **b**, IC50 values of *S. epidermidis* AIP-I (**5**) against reporter strains of *S. aureus* (SA), *S. epidermidis* (SE), *S. lugdunensis* (SL) were determined through fluorescence readout as a measure of agr activity. The curves were generated from three individual assays performed in technical duplicate and shown error bars are the standard deviation of the mean (SD).

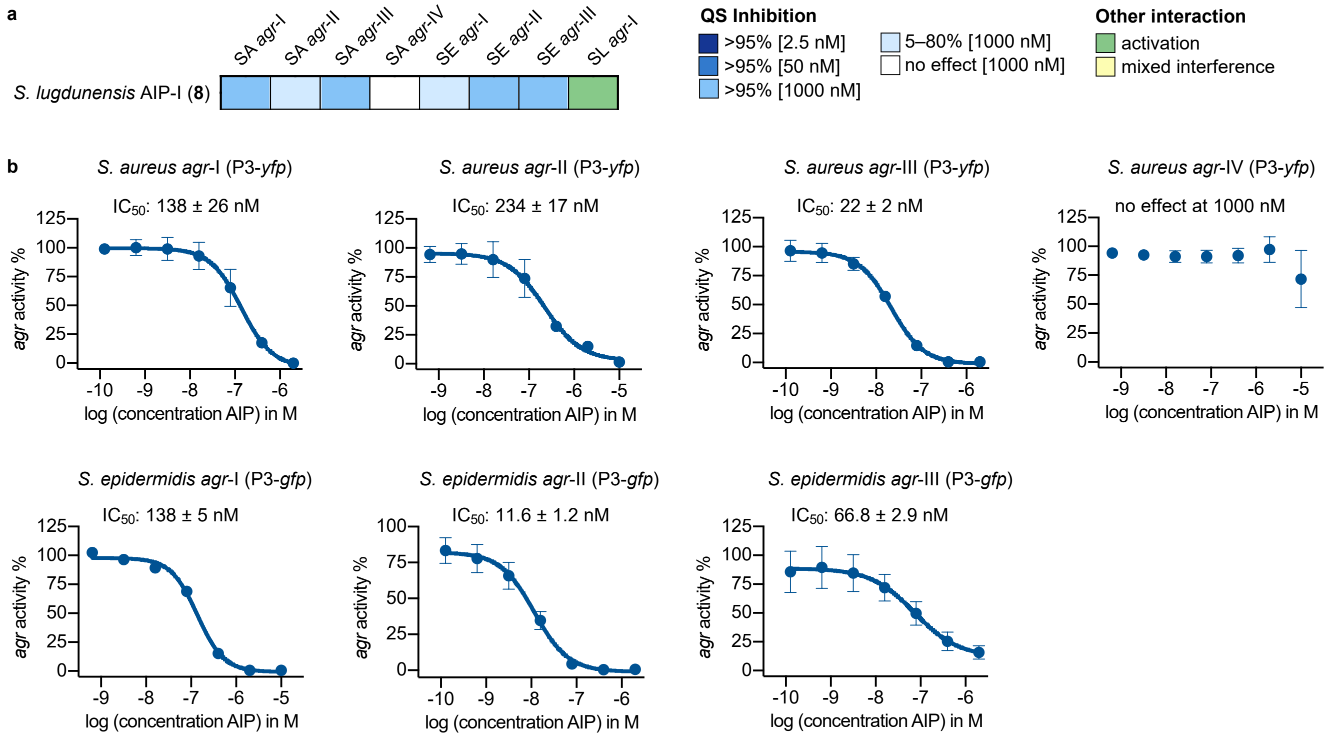

Supplementary Figure S18. Heat map assay validation for *S. lugdunensis* AIP-I (8) through full dose-response curves. a, Heat map data of *S. lugdunensis* AIP-I (**8**). **b**, IC50 values of *S. lugdunensis* AIP-I (**8**) against reporter strains of of *S. aureus* (SA), *S. epidermidis* (SE), *S. lugdunensis* (SL) were determined through fluorescence readout as a measure of agr activity. The curves were generated from three individual assays performed in technical duplicate and shown error bars are the standard deviation of the mean (SD).

Dose-response curves for AgrC interference using *β*-lactamase reporter assays

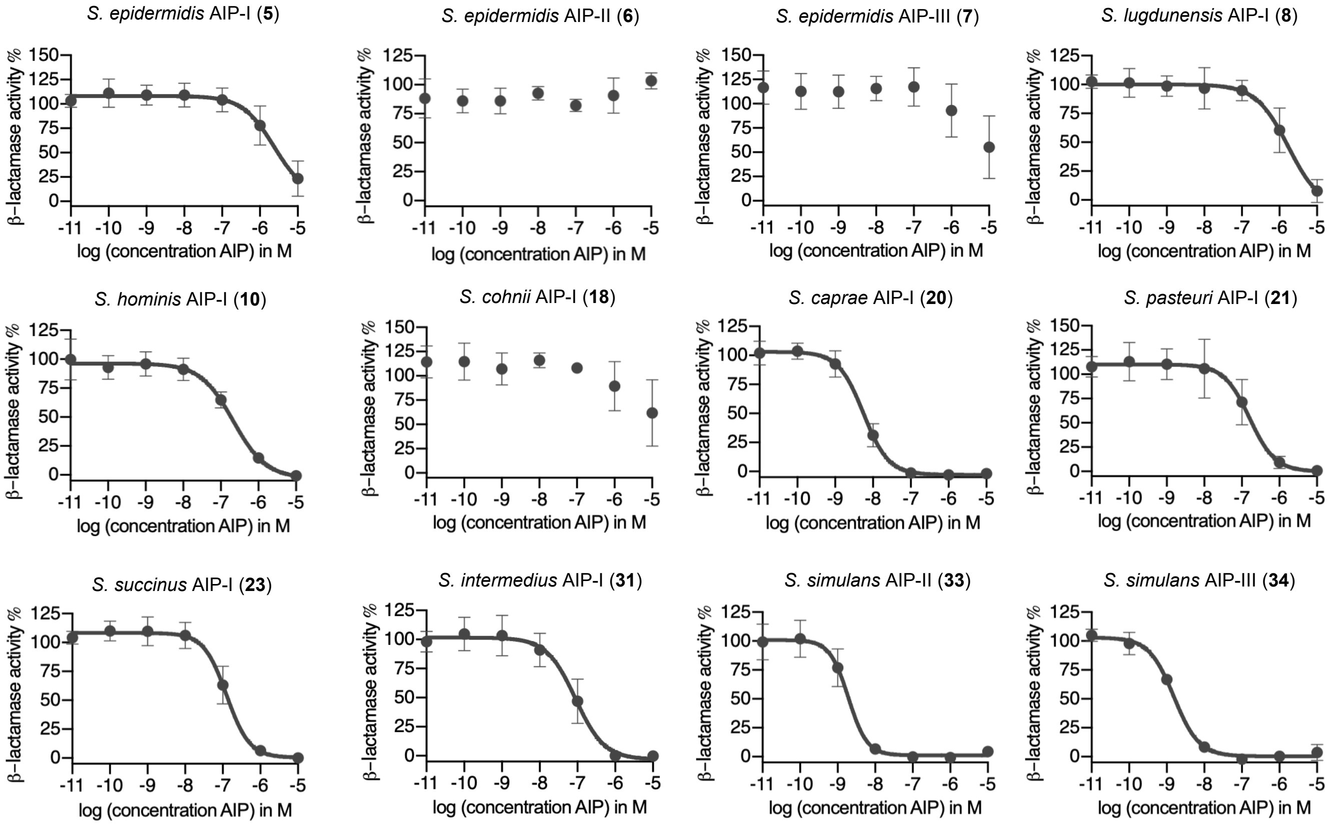

Supplementary Figure S19. Dose-response curves of AgrC inhibition of *S. aureus agr*-I. Inhibition properties of synthetic AIPs (10 μM to 10 pM) were determined through *β*-lactamase activity using the *S. aureus agr*-I reporter strain in the presence of 100 nM *S. aureus* AIP-I (**1**). The curves were generated from three individual assays performed in technical duplicate and shown error bars are the standard deviation of the mean (SD).

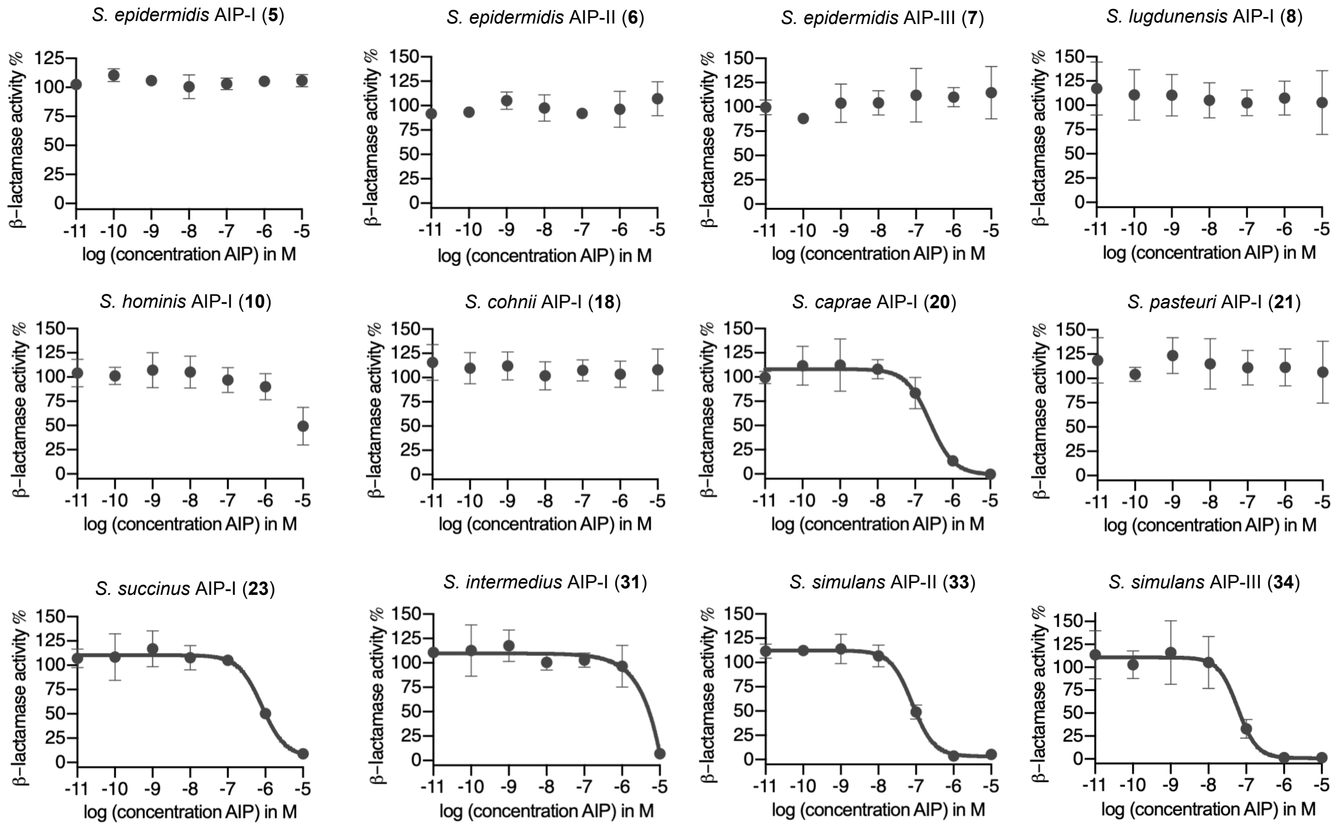

Supplementary Figure S20. Dose-response curves of AgrC inhibition of *S. aureus agr*-II. Inhibition properties of synthetic AIPs (10 µM to 10 pM) were determined through *β*-lactamase activity using the *S. aureus agr*-II reporter strain in the presence of 100 nM *S. aureus* AIP-II (**2**). The curves were generated from three individual assays performed in technical duplicate and shown error bars are the standard deviation of the mean (SD).

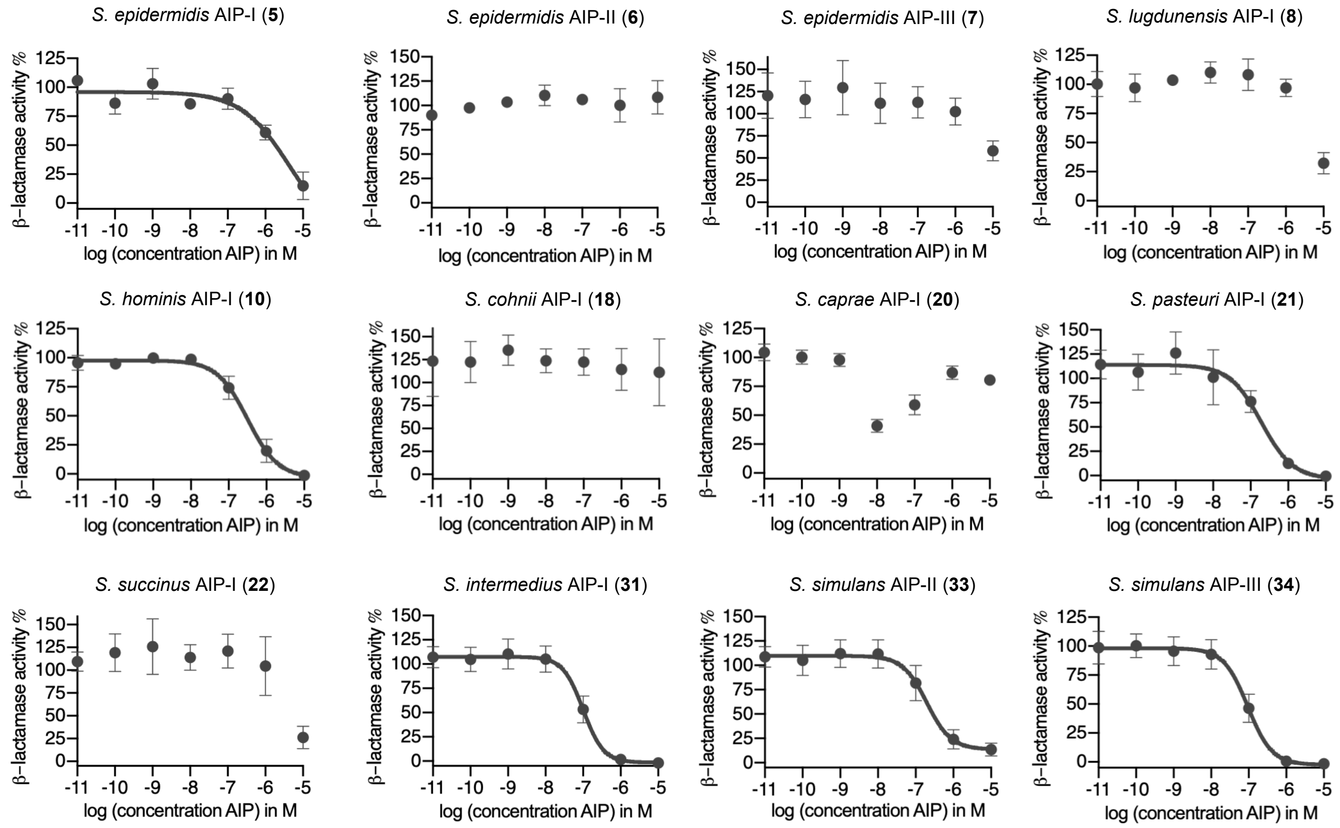

Supplementary Figure S21. Dose-response curves of AgrC inhibition of *S. aureus agr*-III. Inhibition properties of synthetic AIPs (10 μM to 10 pM) were determined through *β*-lactamase activity using the *S. aureus agr*-III reporter strain in the presence of 100 nM *S. aureus* AIP-III (**3**). The curves were generated from three individual assays performed in technical duplicate and shown error bars are the standard deviation of the mean (SD).

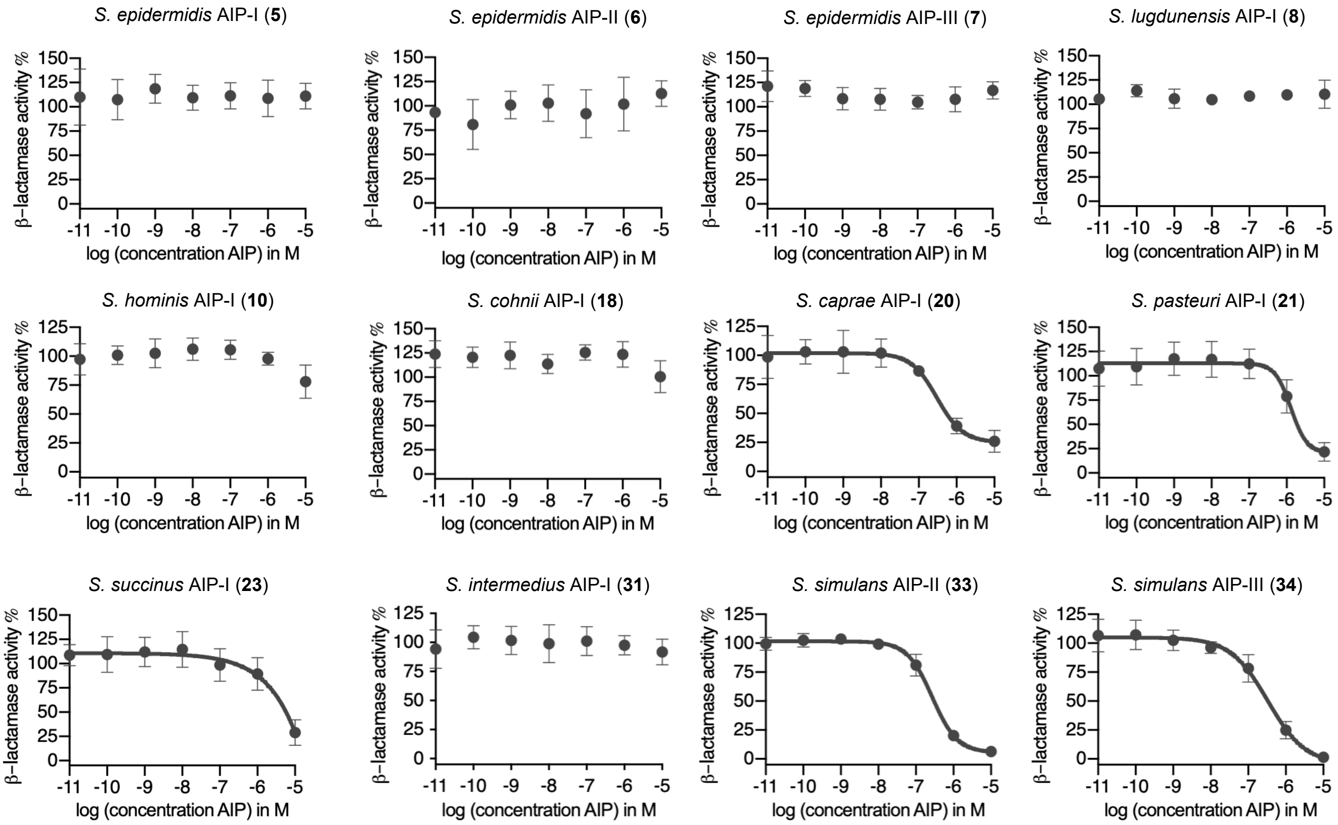

Supplementary Figure S22. Dose-response curves of AgrC inhibition of *S. aureus agr*-IV. Inhibition properties of synthetic AIPs (10 µM to 10 pM) were determined through *β*-lactamase activity using the *S. aureus agr*-IV reporter strain in the presence of 100 nM *S. aureus* AIP-IV (**4**). The curves were generated from three individual assays performed in technical duplicate and shown error bars are the standard deviation of the mean (SD).

Overnight growth and fluorescence curves in presence of synthetic AIPs

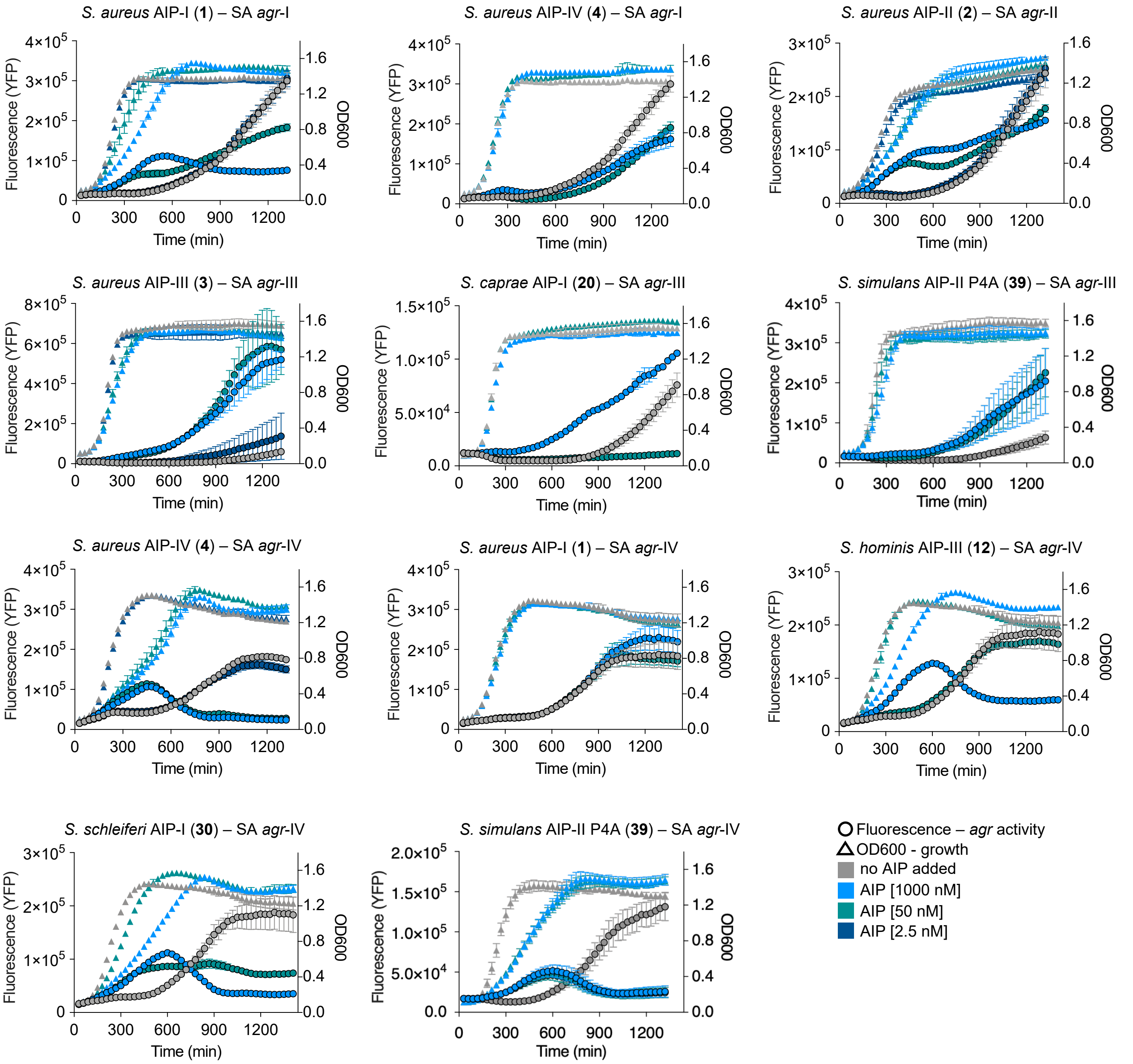

Supplementary Figure S23. Fluorescence and OD_{600} curves for peptides with *S. aureus* (SA) *agr*-I–IV. YFP-producing reporter strains of SA were grown overnight in the absence or presence of AIPs (1000 nM, 50 nM and 2.5 nM) with continuous measurements of fluorescence (YFP) and OD_{600}. The curves represent single experiments performed in technical triplicate. Data points are the mean and error bars are the standard deviation of the mean (SD).

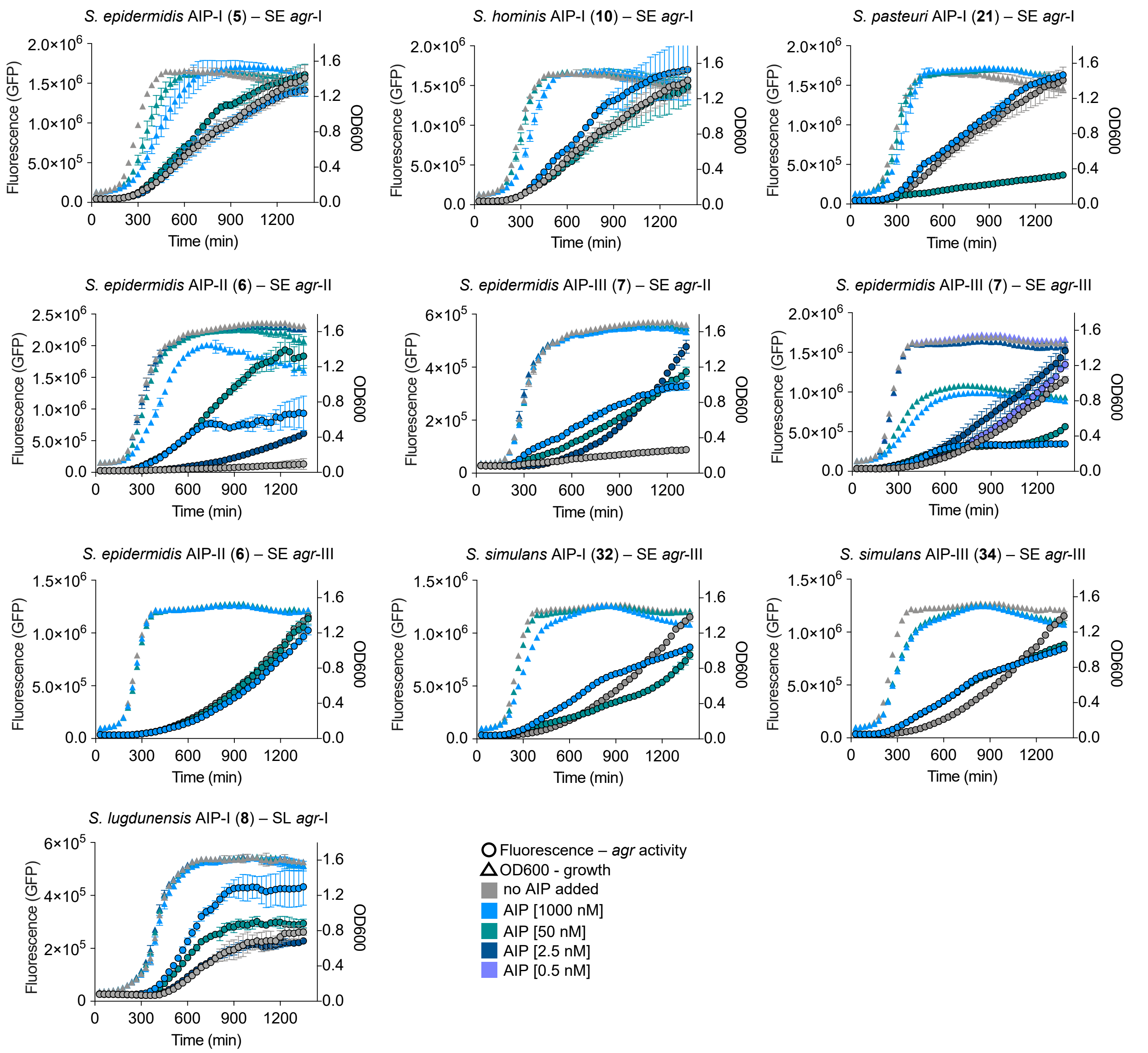

Supplementary Figure S24. Fluorescence and OD_{600} curves for peptides with *S. epidermidis* (SE) *agr*-I–III and *S. lugdunensis* (SL) *agr*-I. sGFP-producing reporter strains of SE and SL were grown overnight in the absence or presence of AIPs (1000 nM, 50 nM, 2.5 nM and 0.5 nM) with continuous measurements of fluorescence (GFP) and OD_{600}. The curves represent single experiments performed in technical triplicate. Data points are the mean and error bars are the SD.

Dose-response curves for *agr* interference of *S. simulans* AIPs

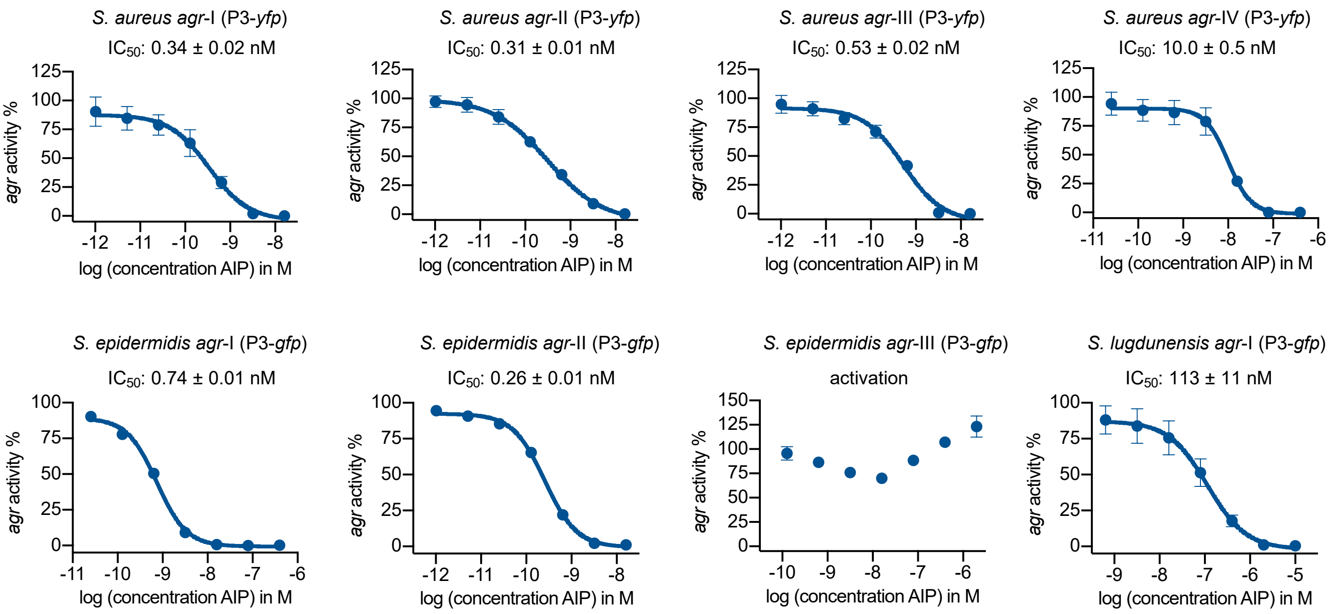

Supplementary Figure S25. Dose-response (IC_{50}) curves for *agr* inhibition by *S. simulans* AIP-I (32). Inhibition properties of *S. simulans* AIP-I (**32**) were determined through fluorescence readout as a measure of *agr* activity against reporter strains of of *S. aureus* (SA), *S. epidermidis* (SE), S. *lugdunensis* (SL). The curves were generated from three individual assays performed in technical duplicate and shown error bars are the standard deviation of the mean (SD).

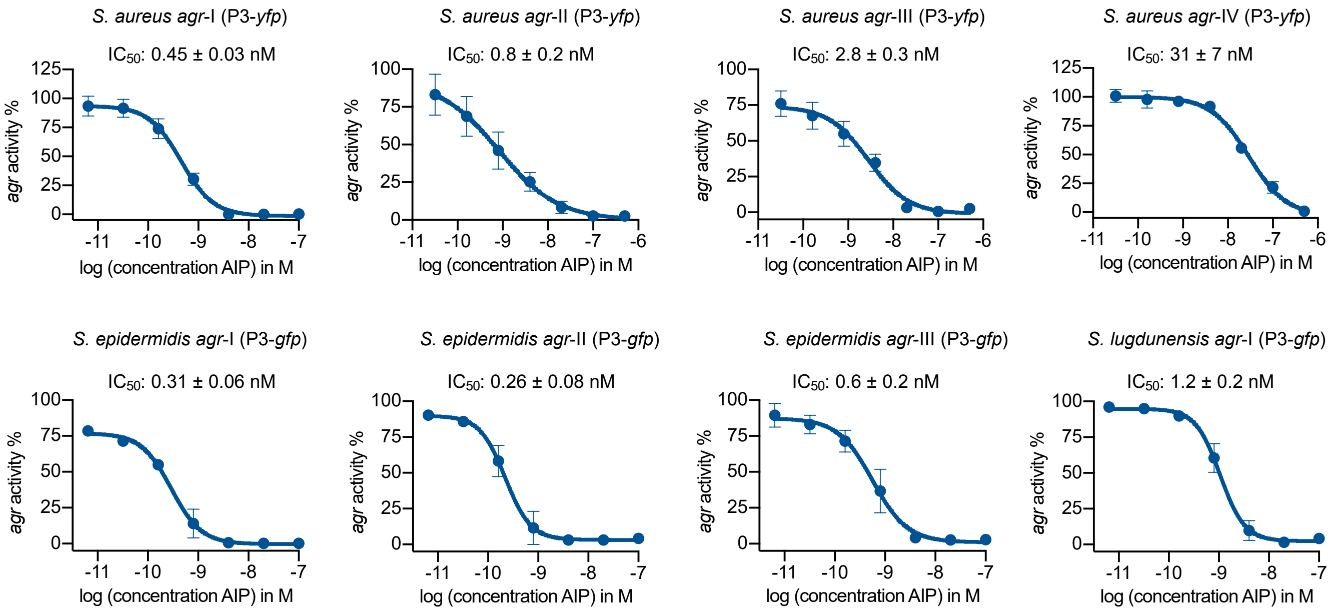

Supplementary Figure S26. Dose-response (IC_{50}) curves for *agr* inhibition by *S. simulans* AIP-II (33). Inhibition properties of *S. simulans* AIP-II (**33**) were determined through fluorescence readout as a measure of *agr* activity against reporter strains of of *S. aureus* (SA), *S. epidermidis* (SE), S. *lugdunensis* (SL). The curves were generated from three individual assays performed in technical duplicate and shown error bars are the standard deviation of the mean (SD).

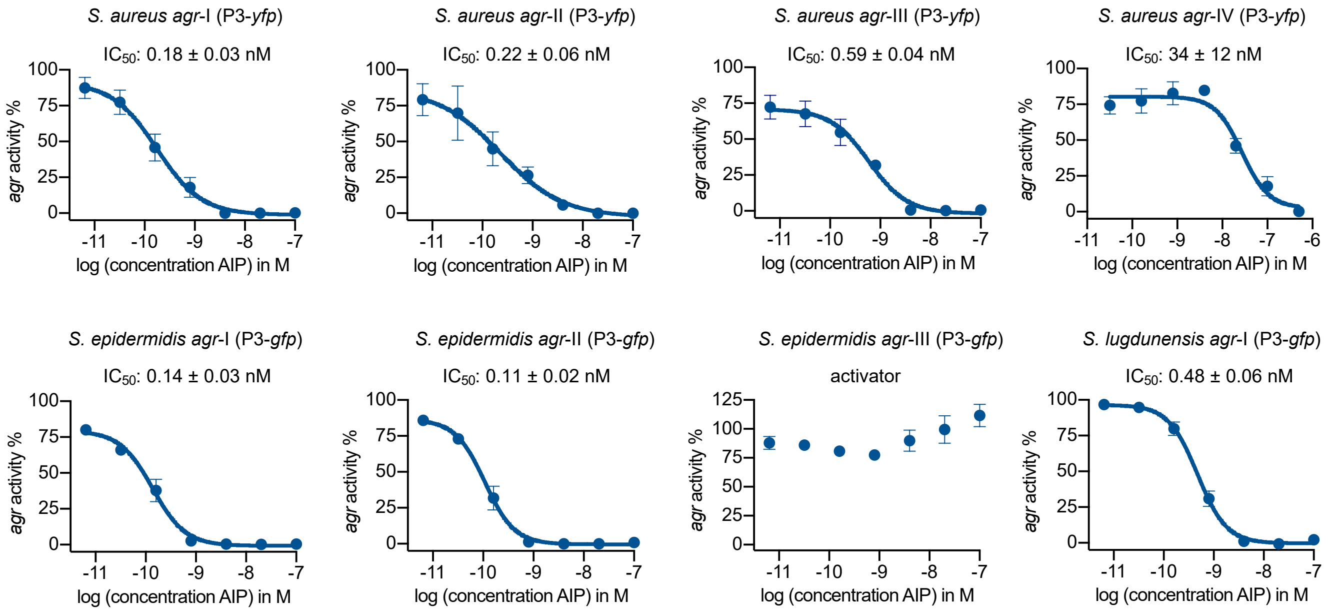

Supplementary Figure S27. Dose-response (IC_{50}) curves for *agr* inhibition by *S. simulans* AIP-III (34). Inhibition properties of *S. simulans* AIP-III (**34**) were determined through fluorescence readout as a measure of *agr* activity against reporter strains of of *S. aureus* (SA), *S. epidermidis* (SE), S. *lugdunensis* (SL). The curves were generated from three individual assays performed in technical duplicate and shown error bars are the standard deviation of the mean (SD).

Bar graphs for *agr* interference of *S. simulans* AIPs SAR study

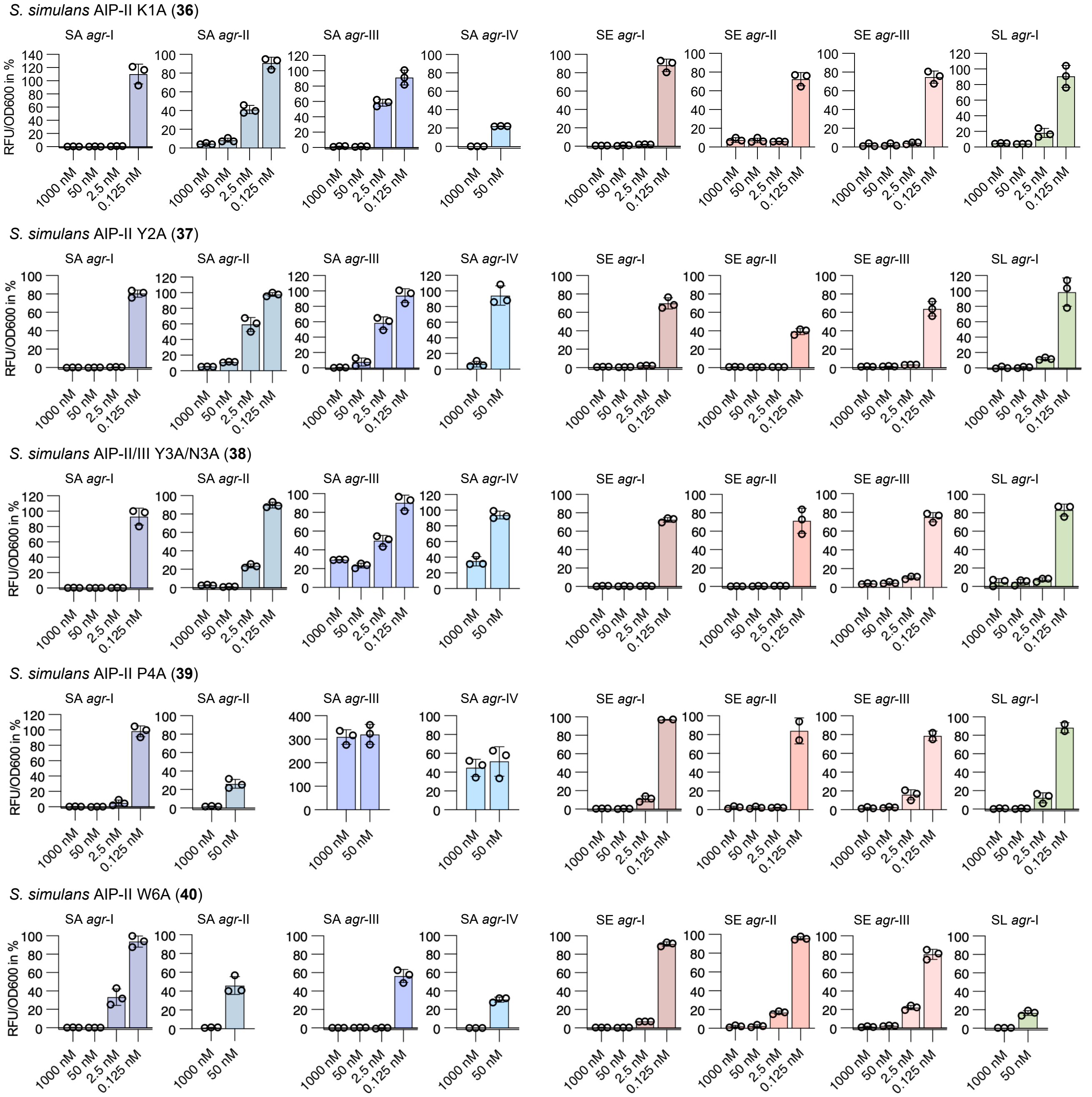

Supplementary Figure S28. Fluorescence reporter strain assay for *agr* interference with SAR peptides. Fluorescent reporter strains of of *S. aureus* (SA), *S. epidermidis* (SE), S. *lugdunensis* (SL) were treated with AIPs at 1000 nM and 50 nM. AIPs were further tested at 2.5 nM and 0.125 nM in case >75% inhibition was observed at higher concentrations. Error bars are the SEM of at least two individual biological assays performed in technical triplicate.

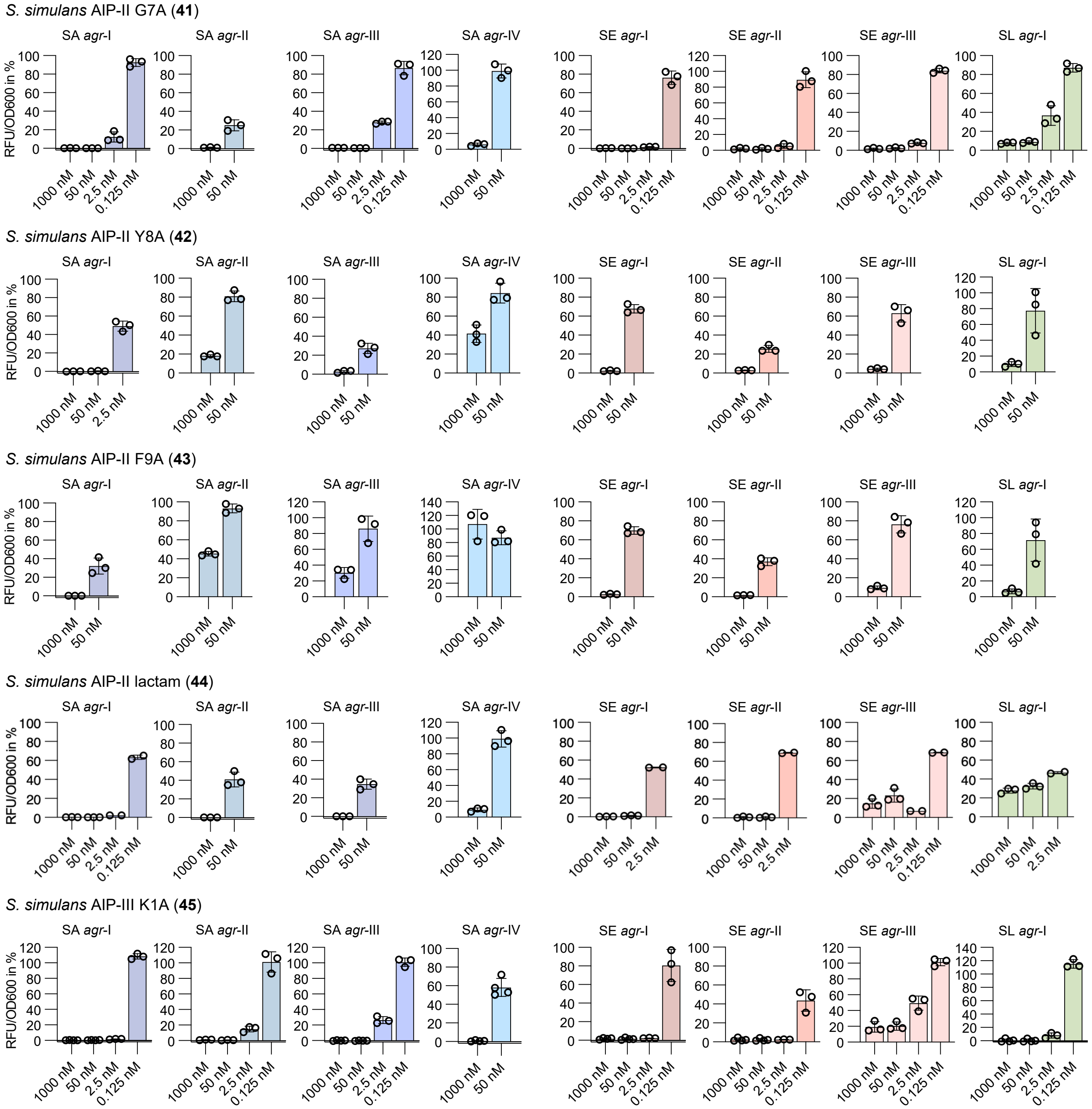

Supplementary Figure S29. Fluorescence reporter strain assay for *agr* interference with SAR peptides. Fluorescent reporter strains of of *S. aureus* (SA), *S. epidermidis* (SE), S. *lugdunensis* (SL) were treated with AIPs at 1000 nM and 50 nM. AIPs were further tested at 2.5 nM and 0.125 nM in case >75% inhibition was observed at higher concentrations. Error bars are the SEM of at least two individual biological assays performed in technical triplicate.

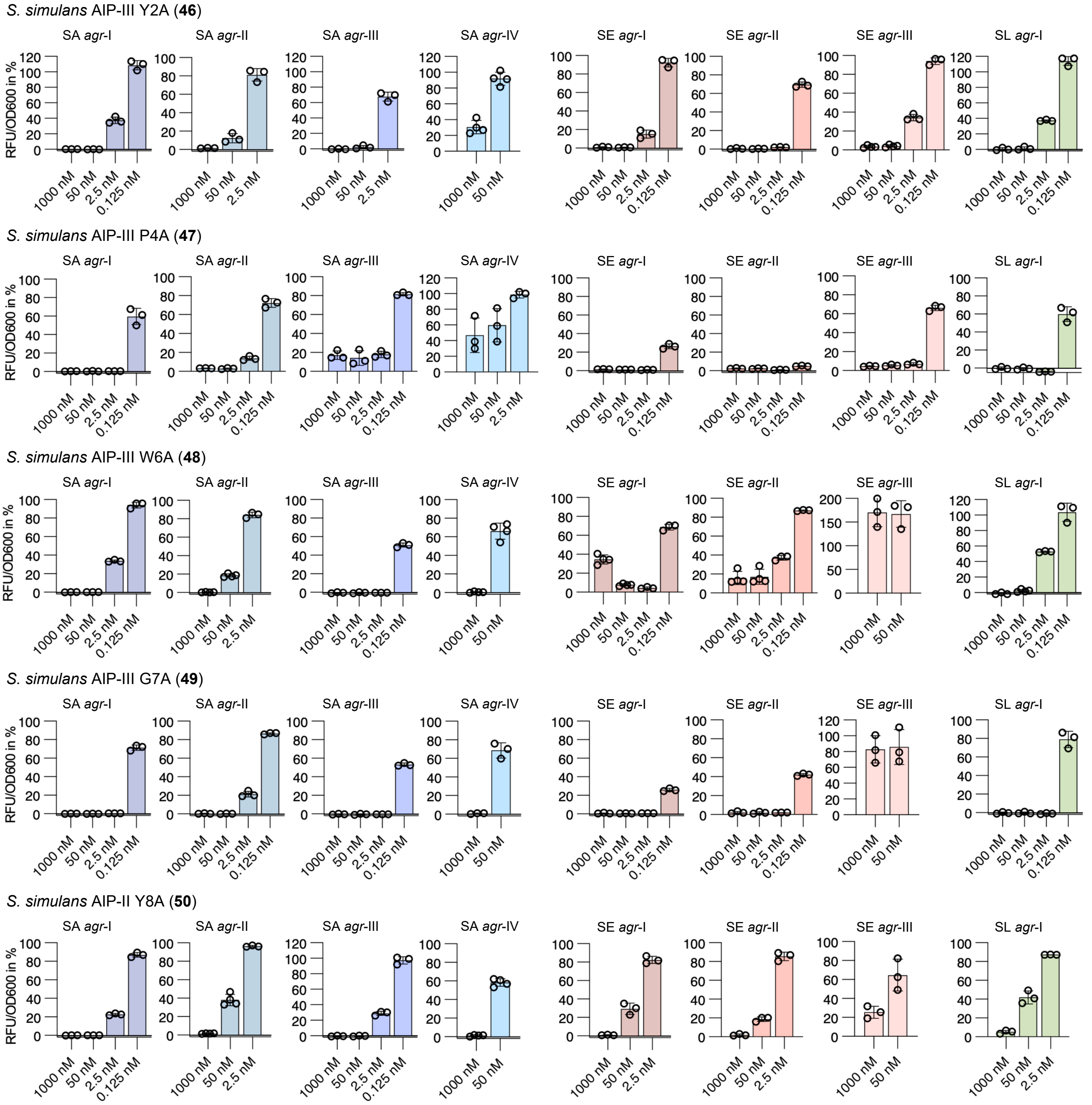

Supplementary Figure S30. Fluorescence reporter strain assay for *agr* interference with SAR peptides. Fluorescent reporter strains of of *S. aureus* (SA), *S. epidermidis* (SE), S. *lugdunensis* (SL) were treated with AIPs at 1000 nM and 50 nM. AIPs were further tested at 2.5 nM and 0.125 nM in case >75% inhibition was observed at higher concentrations. Error bars are the SEM of at least two individual biological assays performed in technical triplicate.

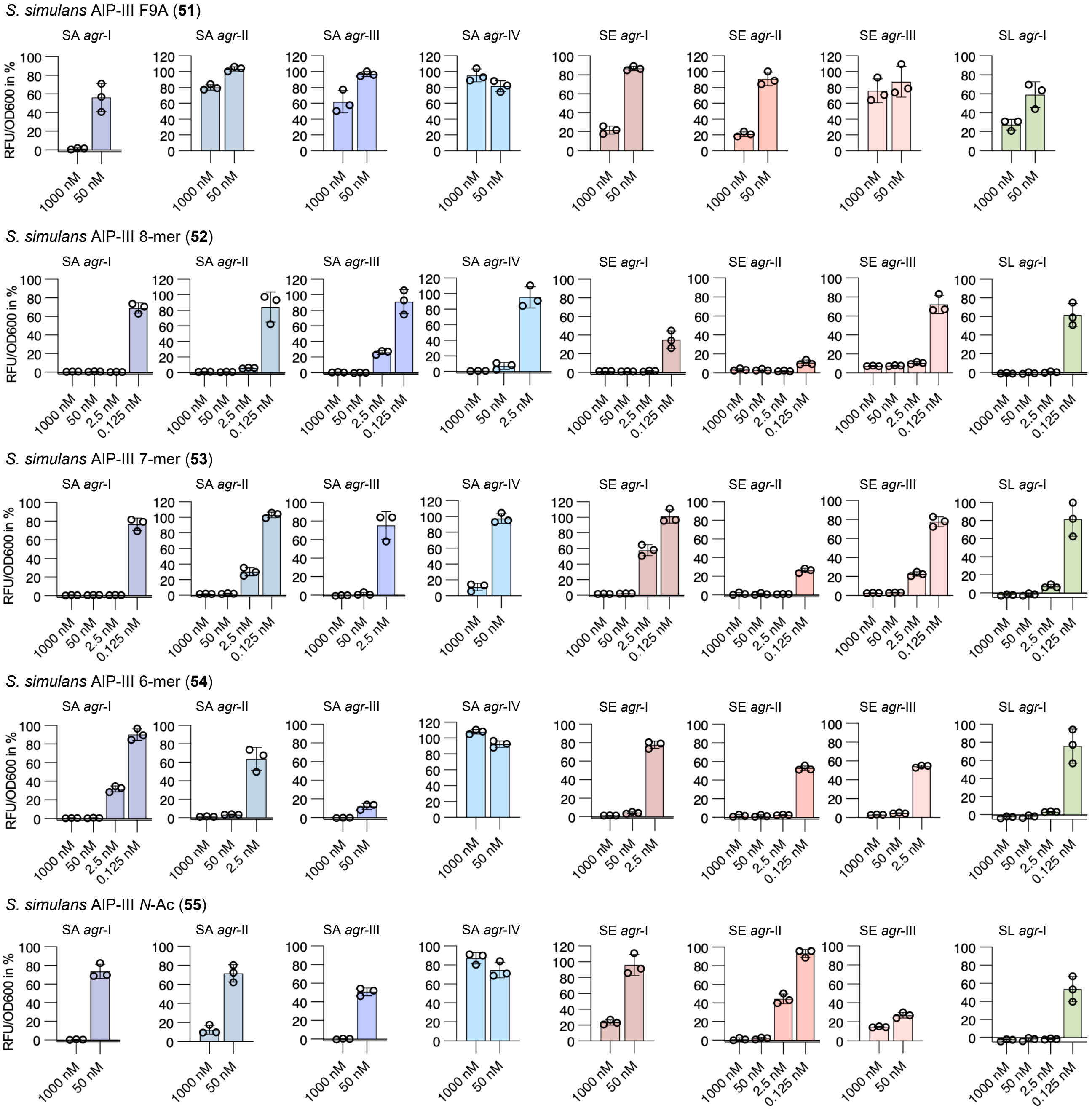

Supplementary Figure S31. Fluorescence reporter strain assay for *agr* interference with SAR peptides. Fluorescent reporter strains of of *S. aureus* (SA), *S. epidermidis* (SE), S. *lugdunensis* (SL) were treated with AIPs at 1000 nM and 50 nM. AIPs were further tested at 2.5 nM and 0.125 nM in case >75% inhibition was observed at higher concentrations. Error bars are the SEM of at least two individual biological assays performed in technical triplicate.

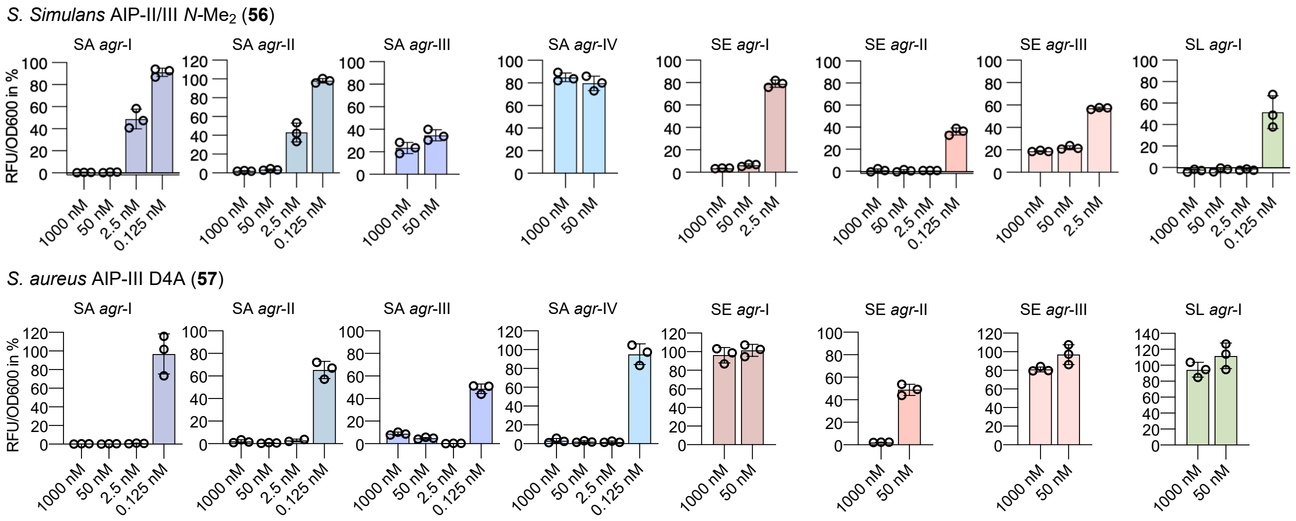

Supplementary Figure S32. Fluorescence reporter strain assay for *agr* interference with SAR peptides. Fluorescent reporter strains of of *S. aureus* (SA), *S. epidermidis* (SE), S. *lugdunensis* (SL) were treated with AIPs at 1000 nM and 50 nM. AIPs were further tested at 2.5 nM and 0.125 nM in case >75% inhibition was observed at higher concentrations. Error bars are the SEM of at least two individual biological assays performed in technical triplicate.

Overnight growth and fluorescence curves of *spa*-GFP agr deactivation assay

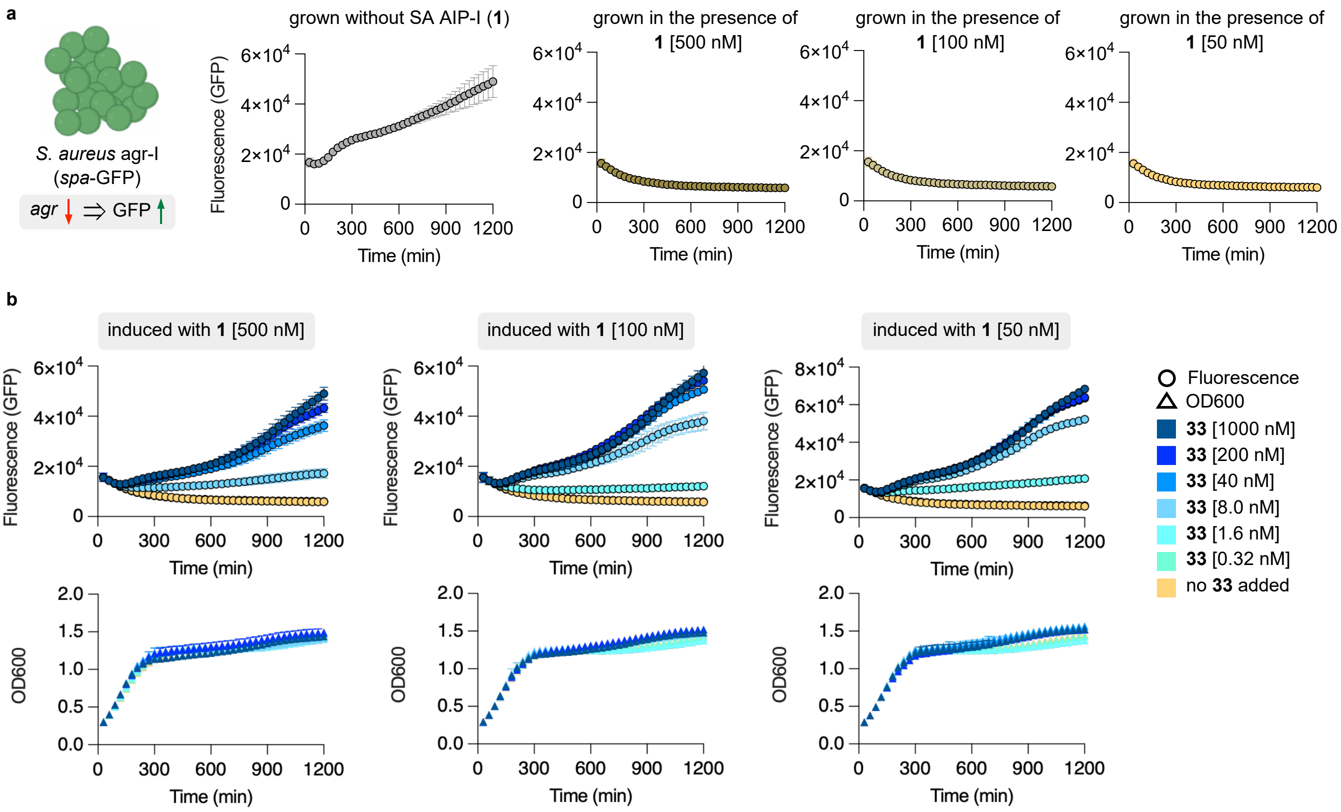

Supplementary Figure S33. Fluorescence and OD_{600} curves for *spa*-GFP *S. aureus agr*-I reporter strain assays. a, Fluorescence monitored of *S. aureus agr*-I *spa*-GFP reporter strain grown in the presence and absence of SA AIP-I (**1**). **b**, Fluorescence and growth curves of *S. aureus agr*-I *spa*-GFP induced with different concentrations of **1** (50, 100 and 500 nM) for 2-3 h followed by the addition of *S. simulans* AIP-II (**33**) at different concentrations (1000–0.32 nM). The curves were generated from three individual assays performed in technical duplicate and shown error bars are the standard deviation of the mean (SD).

2. Supplementary schemes

Synthesis of thiolactone-containing AIPs

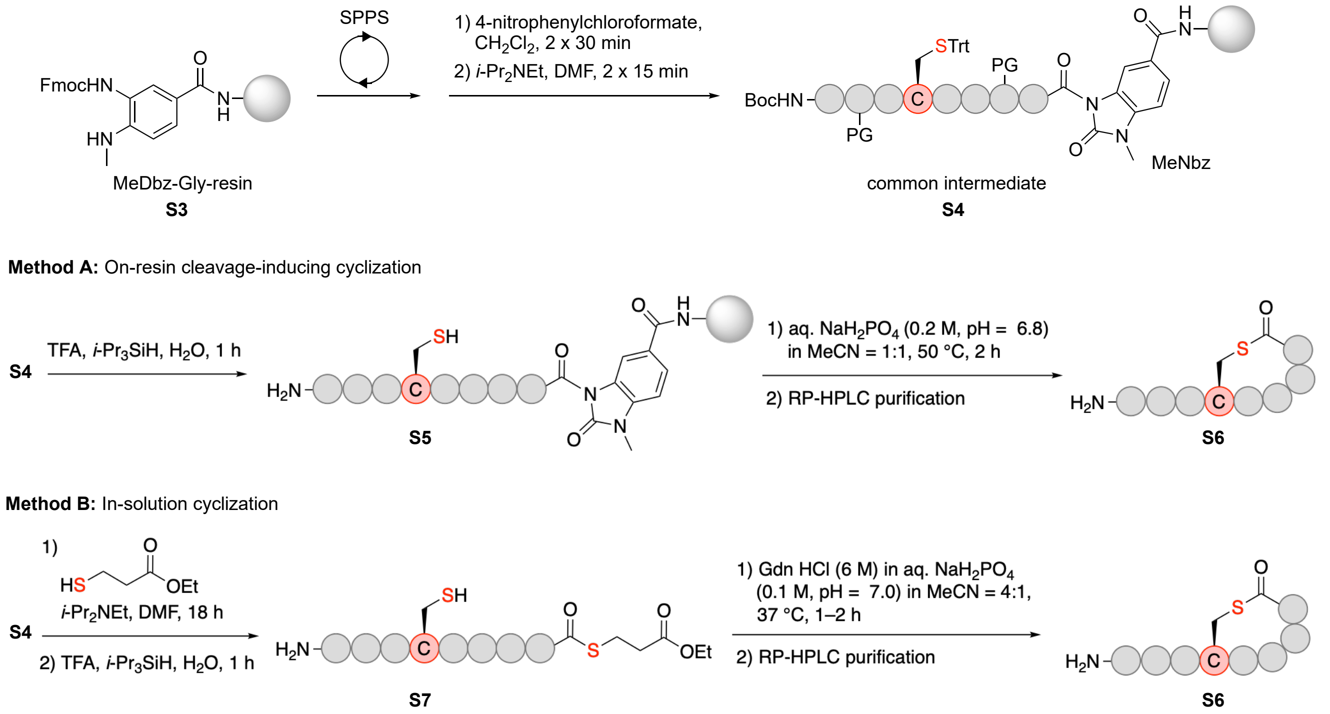

Supplementary Scheme S1. Synthesis of thiolactone-containing AIPs using the MeDbz linker. *N*-acyl-benzimidazolinone (Nbz) intermediates **S4** were synthesized on MeDbz-Gly resin and used for method A and B. In method A, **S4** was used for an on-resin cleavage-inducing cyclization protocol and in method B **S4** was treated with a thiol to induce a cleavage-inducing thioesterification followed by a chemoselective cyclization in solution to afford AIPs **S6**.

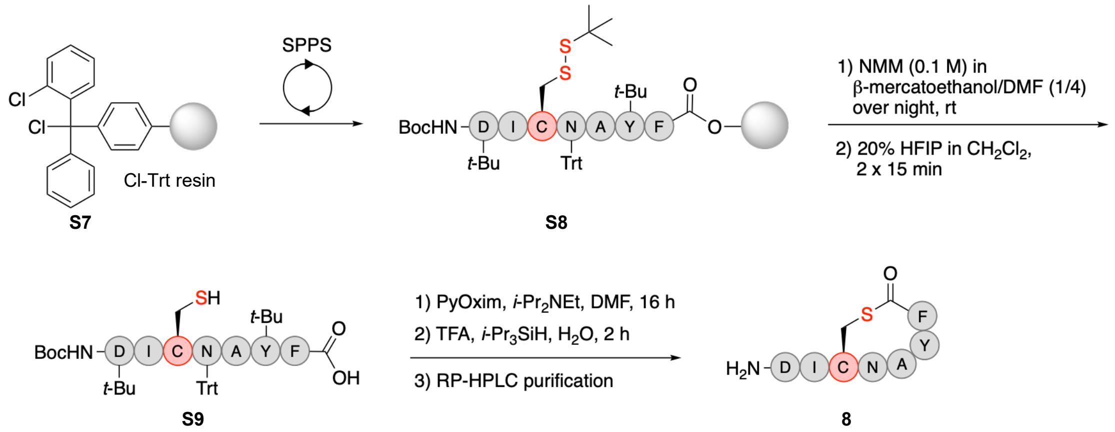

Supplementary Scheme S2. Synthesis of *S. lugdunensis* AIP-I (8) via PyOxim-mediated thiolactonization. The linear peptide **S8** was synthesized on Cl-Trt resin **S7** and the cysteine side chain protecting group removed on-resin. The partially-protected peptide **S9** was released from the resin and the peptide cyclized via PyOxim-mediated thiolactonization to give peptide **8**.

Synthesis of *S. intermedius* AIP

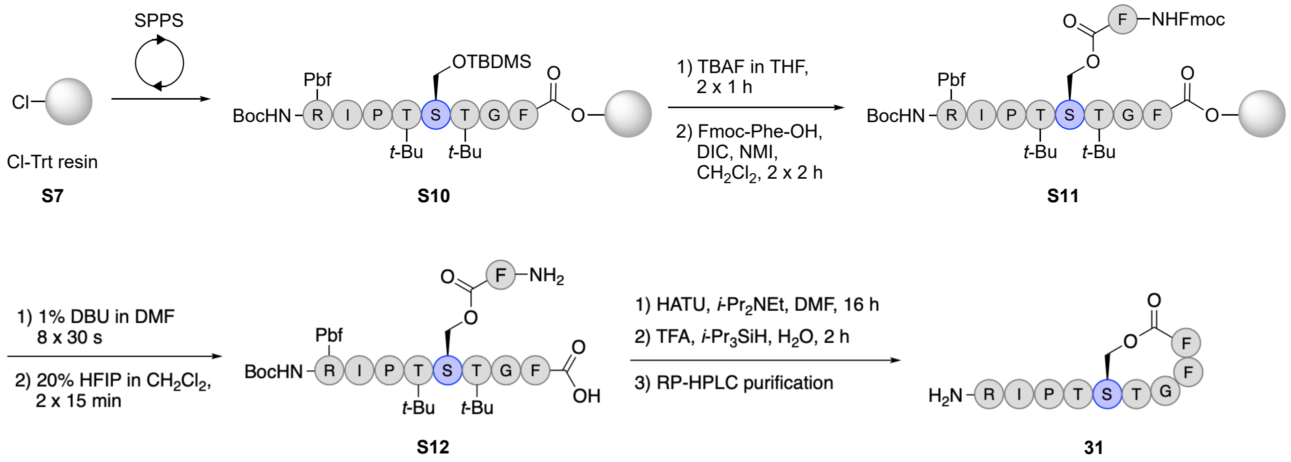

Supplementary Scheme S3. Synthesis of *S. intermedius* AIP-I (31) using an on-resin esterification strategy. The linear peptide **S10** was synthesized on Cl-Trt resin **S7** and the serine side chain protecting group was removed on-resin followed by esterification to give **S11**. The partially-protected peptide **S12** was released from the resin and the peptide cyclized via HATU-mediated amide coupling to give peptide **31**.

Synthesis of *S simulans* AIP-II lactam

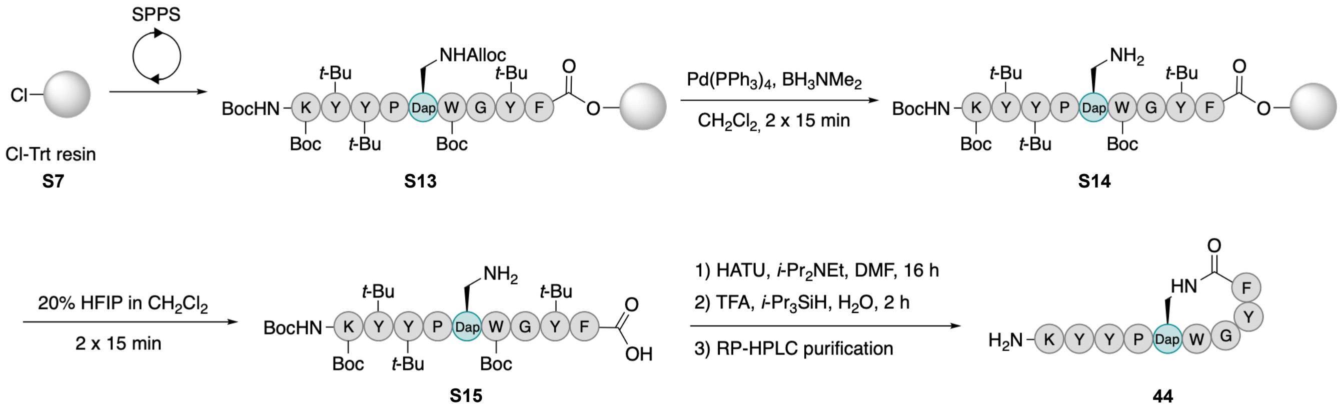

Supplementary Scheme S4. Synthesis of *S. simulans* AIP-II lactam (44). The linear peptide **S13** was synthesized on Cl-Trt resin **S7** and the 2,3-diaminopropionic acid (Dap) side chain protecting group was removed on-resin. The partially-protected peptide **S15** was released from the resin and the peptide cyclized via HATU-mediated amide coupling to give peptide **44**.

3. Supplementary tables

Staphylococci species and sequences of known AIPs.

Supplementary Table S1. Information about staphylococci species and sequences of known AIPs.

Species AIP	Sequence	Associated Host	Phylogenetic species group
S. aureus AIP-I (**1**) [*S. argenteus* AIP-I]	YST-[CDFIM]	human/animal	Epidermidis-Aureus
S. aureus AIP-II (**2**)	GVNA-[CSSLF]	human/animal	Epidermidis-Aureus
S. aureus AIP-III (**3**)	IN-[CDFLL]	human/animal	Epidermidis-Aureus
S. aureus AIP-IV (**4**) [*S. schweitzeri* AIP-I]	YST-[CYFIM]	human/animal	Epidermidis-Aureus
S. epidermidis AIP-I (**5**)	DSV-[CASYF]	human/animal	Epidermidis-Aureus
S. epidermidis AIP-II (**6**)	NASKYNP-[CSNYL]	human/animal	Epidermidis-Aureus
S. epidermidis AIP-III (**7**)	NAAKYNP-[CASYL]	human/animal	Epidermidis-Aureus
S. lugdunensis AIP-I (**8**)	DI-[CNAYF]	human	Epidermidis-Aureus
S. lugdunensis AIP-II (**9**)	DM-[CNGYF]	human	Epidermidis-Aureus
S. hominis AIP-I (**10**)	SYNV-[CGGYF]	human	Epidermidis-Aureus
S. hominis AIP-II (**11**)	SYSP-[CATYF]	human	Epidermidis-Aureus
S. hominis AIP-III (**12**)	TYST-[CYGYF]	human	Epidermidis-Aureus
S. hominis AIP-IV (**13**)	TINT-[CGGYF]	human	Epidermidis-Aureus
S. hominis AIP-V (**14**)	SQTV-[CSGYF]	human	Epidermidis-Aureus
S. haemolyticus AIP-I (**15**)	SFTP-[CTTYF]	human	Epidermidis-Aureus
S. warneri AIP-I (**16**)	YSP-[CTNFF]	human	Epidermidis-Aureus
S. warneri AIP-II (**17**)	ANP-[CAMFY]	human	Epidermidis-Aureus
S. cohnii AIP-I (**18**)	TISSVKP-[CTGFV]	human	Saprophyticus
S. saprophyticus AIP-I (**19**)	INP-[CFGYT]	human	Saprophyticus
S. caprae AIP-I (**20**)	YST-[CSYYF]	animal	Epidermidis-Aureus
S. pasteuri AIP-I (**21**)	ANP-[CAGYF]	animal	Epidermidis-Aureus
S. devriesei AIP-I (**22**)	YKP-[CFGYF]	animal	Epidermidis-Aureus
S. succinus AIP-I (**23**)	ATAGALP-[CGGFF]	animal	Saprophyticus
S. equorum AIP-I (**24**)	AVAGARP-[CYGYF]	animal	Saprophyticus
S. equorum AIP-II (**25**)	AVAGLSP-[CGGYF]	animal	Saprophyticus
S. hyicus AIP-I (**26**)	KINP-[CTVFF]	animal	Hyicus-Intermedius
S. chromogenes AIP-I (**27**)	SINP-[CTGFF]	animal	Hyicus-Intermedius
S. chromogenes AIP-II (**28**)	AMDP-[CTGFF]	animal	Hyicus-Intermedius
S. chromogenes AIP-III (**29**)	SINP-[CTAFF]	animal	Hyicus-Intermedius
S. schleiferi AIP-I (**30**)	KYPF-[CIGYF]	animal	Hyicus-Intermedius
S. intermedius AIP-I (**31**)	RIPT-[STGFF]	animal	Hyicus-Intermedius
S. simulans AIP-I (**32**)	KYNP-[CLGFL]	animal	Hyicus-Intermedius
S. simulans AIP-II (**33**)	KYYP-[CWGYF]	animal	Hyicus-Intermedius
S. simulans AIP-III (**34**)	KYNP-[CWGYF]	animal	Hyicus-Intermedius
S. vitulinus AIP-I (**35**)	VIRG-[CTAFL]	animal	Sciuri

List of recorded and reported quorum sensing interactions of staphylococcal AIPs

Supplementary Table S2. IC_{50} and EC_{50} values (nM) measured in β-lactamase assay using *S. aureus* (SA) AgrC-I–IV reporter strains.

Species AIP	SA AgrC-I	SA AgrC-II	SA AgrC-III	SA AgrC-IV
S. aureus AIP-I (**1**)	**EC50:** 5.4 ± 0.5	700 ± 165	80 ± 11	**EC50:** 790 ± 66
S. aureus AIP-II (**2**)	110 ± 12	**EC50:** 3.7 ± 0.5	40 ± 15	230 ± 19
S. aureus AIP-III (**3**)	120 ± 15	150 ± 63	**EC50:** 11 ± 1	>1000
S. aureus AIP-IV (**4**)	**EC50:** 59 ± 2	47 ± 9 nM	9.8 ± 0.2[a]	**EC50:** 2.6 ± 0.2
S. epidermidis AIP-I (**5**)	>1000	-[b]	>1000	-[b]
S. epidermidis AIP-II (**6**)	-	-[b]	-[b]	-[b]
S. epidermidis AIP-III (**7**)	>10000	-[b]	>10000	-[b]
S. lugdunensis AIP-I (**8**)	>1000	-[b]	>1000	-[b]
S. lugdunensis AIP-II (**9**)	>1000	-[b]	-[b]	-[b]
S. hominis AIP-I (**10**)	224 ± 37	>1000	338 ± 83	-[b]
S. hominis AIP-III (**12**)	134 ± 8	580 ± 61	220 ± 46[a]	**EC50:** 54 ± 5
S. haemolyticus AIP-I (**15**)	340 ± 25	-[b]	340 ± 91	-
S. warneri AIP-I (**16**)	100 ± 10	1000 ± 105	460 ± 59	>1000
S. cohnii AIP-I (**18**)	>10000	-[b]	-[b]	-[b]
S. saprophyticus AIP-I (**19**)	360 ± 30	-	-[b]	-[b]
S. caprae AIP-I (**20**)	5.7 ± 0.8	252 ± 44	-[a]	199 ± 19
S. pasteuri AIP-I (**21**)	155 ± 34	-[b]	191 ± 18	>1000
S. succinus AIP-I (**23**)	117 ± 26	854 ± 65	>1000	>1000
S. hyicus AIP-I (**26**)	3.3 ± 0.5	350 ± 90	4.0 ± 0.8	180 ± 33
S. chromogenes AIP-I (**27**)	15 ± 1	200 ± 13	60 ± 13	350 ± 85
S. schleiferi AIP-I (**30**)	2.8 ± 0.8	86 ± 6	80 ± 16[a]	**EC50:** 31 ± 6
S. intermedius AIP-I (**31**)	87 ± 20	>1000	104 ± 20	-[b]
S. simulans AIP-I (**32**)	8.6 ± 0.5	23 ± 2	50 ± 11	280 ± 66
S. simulans AIP-II (**33**)	1.9 ± 0.3	80 ± 7	198 ± 33	273 ± 26
S. simulans AIP-III (**34**)	1.6 ± 0.1	60 ± 7	103 ± 5	351 ± 60
S. vitulinus AIP-I (**35**)	190 ± 15	800 ± 116	690 ± 46	-[b]
S. aureus AIP-III D4A (**57**)	3.3 ± 0.7	34 ± 1	1.6 ± 0.2[a]	10 ± 0.2

All inhibition assays were performed in the presence of 100 nM cognate AIP and the results represent means ± standard error of the mean (SEM) of at least duplicate determinations performed in biological triplicate. [a]Only partial inhibition observed. [b]No inhibition recorded at the highest AIP concentration tested. Blue shaded boxes represent IC_{50} values determined in this study. White represent IC_{50}/EC_{50} values determined previously.(1)

Supplementary Table S3. Reported QS interactions of staphylococcal AIPs against *S. aureus* (SA) *agr*-I–IV.

Species	SA *agr*-I	SA *agr*-II	SA *agr*-III	SA *agr*-IV	Assay type	Ref.
S. aureus AIP-I (**1**)	n. t.	**IC_{50}:** 3.4 ± 1.2 nM	**IC_{50}:** 3.1± 1.3 nM	n. t.	*β*-lactamase	(2)
	**EC_{50}:** 40 ± 9 nM	**IC_{50}:** 26 ± 7 nM	n. t.	n. t.	*β*-lactamase	(3)
	**EC_{50}:** 23 ± 7 nM	**IC_{50}:** 135 ± 52 nM	n. t.	n. t.	*β*-lactamase	(3)
	**EC_{50}:** 28 nM (18–46 CI)	**IC_{50}:** 25 nM (14–45 CI)	**IC_{50}:** 3 nM (2–5 CI)	**EC_{50}:** 26 µM (23–29 CI)	*β*-lactamase	(4)
	n. v.	**IC_{50}:** 8.00 nM (3.7–17.5 CI)	**IC_{50}:** 0.53 nM (0.31–0.88 CI)	n. v.	fluorescence	(5)
	n. t.	**IC_{50}:** 3.34 nM (0.67–16.5 CI)	**IC_{50}:** 6.12 nM (5.20–7.21 CI)	n. t.	hemolysis	(5)
	**EC_{50}:** 11 nM (9–12 CI)	n. t.	n. t.	n. t.	*β*-lactamase	(6)
	**EC_{50}:** 3.21 nM (1.21–8.57 CI)	n. t.	n. t.	n. t.	*β*-lactamase	(7)
S. aureus AIP-II (**2**)	**IC_{50}:** 2.9 ± 1.2 nM	n. t.	**IC_{50}:** 3.2 ± 1.3 nM	n. t.	*β*-lactamase	(2)
	**IC_{50}:** 90 ± 30 nM	**EC_{50}:** 34 ± 6 nM	n. t.	n. t.	*β*-lactamase	(3)
	**IC_{50}:** 78 ± 12 nM	**EC_{50}:** 28 ± 14 nM	n. t.	n. t.	*β*-lactamase	(3)
	**IC_{50}:** 40 nM (12–140 CI)	**EC_{50}:** 30 nM (10–90 CI)	**IC_{50}:** 1 nM (0.7–2.6 CI)	**IC_{50}:** 86 nM (65–111 CI)	*β*-lactamase	(4)
	**IC_{50}:** 1.62 nM (0.93–2.82 CI)	n. v.	**IC_{50}:** 0.53 nM (0.24–1.19 CI)	**IC_{50}:** 0.40 nM (0.21–0.76 CI)	fluorescence	(5)
	**IC_{50}:** 0.89 nM (0.37–2.15 CI)	n. t.	**IC_{50}:** 3.59 nM (1.22–10.6 CI)	**IC_{50}:** 1.19 nM (0.55–2.60 CI)	hemolysis	(5)
	n. t.	**EC_{50}:** 40.9 nM (30.3–55.3 CI)	n. t.	n. t.	*β*-lactamase	(7)
	**IC_{50}:** 12 ± 2.9 nM	n. t.	n. t.	n. t.	*β*-lactamase	(8)
S. aureus AIP-III (**3**)	**IC_{50}:** 70 nM (30–150 CI)	**IC_{50}:** 6 nM (5–6.5 CI)	**EC_{50}:** 26 nM (22–31 CI)	**IC_{50}:** 150 nM (104–207 CI)	*β*-lactamase	(4)
	**IC_{50}:** 5.05 nM (2.46–10.4 CI)	**IC_{50}:** 5.63 nM (1.89–16.7 CI)	n. v.	**IC_{50}:** 8.53 nM (4.15–17.5 CI)	fluorescence	(5)
	**IC_{50}:** 8.07 nM (4.34–15.0 CI)	**IC_{50}:** 0.46 nM (0.27–0.77 CI)	n. t.	**IC_{50}:** 23.8 nM (13.7–41.3 CI)	hemolysis	(5)
	n. t.	n. t.	**EC_{50}:** 406 nM (281–586 CI)	n. t.	*β*-lactamase	(7)
	**IC_{50}:** 8 ± 1.1 nM	n. t.	n. t.	n. t.	*β*-lactamase	(8)
S. aureus AIP-IV (**4**)	**EC_{50}:** 62 nM (52–75 CI)	**IC_{50}:** 4 nM (3–5 CI)	**IC50:** 1 nM (0.5–3 CI)	**EC_{50}:** 13 nM (7–40 CI)	*β*-lactamase	(4)
	n. v.	**IC_{50}:** 0.37 nM (0.22–0.64 CI)	**IC50:** 0.46 nM (0.21–0.99 CI)	n. v.	fluorescence	(5)

	n. t.	**IC$_{50}$:** 0.090 nM (0.078–0.103 CI)	**IC50:** 1.49 nM (0.70–3.14 CI)	n. t.	hemolysis	(5)
	n. t.	n. t.	n. t.	**EC$_{50}$:** 7.90 nM (4.93–12.7 CI)	*β*-lactamase	(7)
S. epidermidis AIP-I (**5**)	**IC$_{50}$:** ~250 nM	**IC$_{50}$:** ~30-40 nM	**IC$_{50}$:** ~10 nM	no inhibition at 1000 nM	HPLC δ-toxin	(9)
	**IC$_{50}$:** 166 nM (68.7–402 CI)	**IC$_{50}$:** >1000 nM	**IC$_{50}$:** 13.0 nM (6.41–26.5 CI)	**IC$_{50}$:** >1000 nM	fluorescence	(10)
S. epidermidis AIP-II (**6**)	no inhibition at 100 nM	no inhibition at 100 nM	no inhibition at 100 nM	no inhibition at 100 nM	fluorescence	(11)
S. lugdunensis AIP-I (**8**)	**IC$_{50}$:** 384 nM (353–418 CI)	**IC$_{50}$:** 419 nM (353–418 CI)	**IC$_{50}$:** 36.6 nM (353–418 CI)	**IC$_{50}$:** >1000 nM	fluorescence	(10)
S. hominis AIP-I (**10**)	**IC$_{50}$:** 0.6243 nM	~70% inhibition by supernatant	~80% inhibition by supernatant	no inhibition by supernatant	fluorescence	(12)
	**IC$_{50}$:** 13 nM (11.0–15.2 CI)	**IC$_{50}$:** 31 nM (25.4–39.0 CI)	**IC$_{50}$:** 5 nM (4.2–6.0 CI)	**IC$_{50}$:** 2910 nM (2155–4543 CI)	fluorescence	(13)
S. hominis AIP-II (**11**)	**IC$_{50}$:** 15 nM (13.5–16.8 CI)	**IC$_{50}$:** 2109 nM (1504–3473 CI)	**IC$_{50}$:** 3 nM (3.0–3.8 CI)	**IC$_{50}$:** 1130 nM (882.2–1474 CI)	fluorescence	(13)
S. hominis AIP-III (**12**)	**IC$_{50}$:** 11 nM (8.8–13.0 CI)	**IC$_{50}$:** 4 nM (2.7–6.8 CI)	**IC$_{50}$:** 6 nM (5.3–7.7 CI)	NA	fluorescence	(13)
S. hominis AIP-IV (**13**)	**IC$_{50}$:** 128 nM (103.5–157.4 CI)	**IC$_{50}$:** 140 nM (109.8–178.0 CI)	**IC$_{50}$:** 37 nM (30.0–46.4 CI)	NA	fluorescence	(13)
S. hominis AIP-V (**14**)	**IC$_{50}$:** 43 nM (36.8–51.3 CI)	**IC$_{50}$:** 59 nM (50.2–69.3 CI)	**IC$_{50}$:** 4 nM (3.3–5.0 CI)	**IC$_{50}$:** 3809 nM (3199–7372 CI)	fluorescence	(13)
S. warneri AIP-I (**16**)	**IC$_{50}$:** 10 nM (9.1–11.7 CI)	**IC$_{50}$:** 4 nM (3.9–4.4 CI)	**IC$_{50}$:** 13 nM (11.4–14.0 CI)	**IC$_{50}$:** 146 nM (122.0–188.0 CI)	fluorescence	(14)
S. warneri AIP-II (**17**)	**IC$_{50}$:** 2 nM (1.6–2.5 CI)	**IC$_{50}$:** 30 nM (22.4–39.1 CI)	**IC$_{50}$:** 2 nM (1.5–1.9 CI)	**IC$_{50}$:** 2 nM (1.4–2.5 CI)	fluorescence	(14)
S. caprae AIP-I (**20**)	**IC$_{50}$0:** 0.6 ± 0.02 nM	**IC$_{50}$:** 0.26 ± 0.08 nM	**IC$_{50}$:** 0.2 nM	**IC$_{50}$:** 8.98 ± 0.32 nM	fluorescence	(15)
S. intermedius AIP-I (**31**)	80–100% inhibition from supernatant	40–90% inhibition from supernatant	60–100% inhibition from supernatant	10–60% inhibition from supernatant	*β*-lactamase	(16)
S. simulans AIP-I (**32**)	**IC$_{50}$:** 2.2 nM	**IC$_{50}$:** 1.1 nM	**IC$_{50}$:** 3.5 nM	**IC$_{50}$:** 23 nM	fluorescence	(17)
S. simulans AIP-II (**33**)	**IC$_{50}$:** 1.6 nM	**IC$_{50}$:** 15 nM	**IC$_{50}$:** 11.5 nM	**IC$_{50}$:** 40 nM	fluorescence	(17)
S. simulans AIP-III (**34**)	**IC$_{50}$:** 1.7 nM	**IC$_{50}$:** 6.0 nM	**IC$_{50}$:** 3.2 nM	**IC$_{50}$:** 48 nM	fluorescence	(17)
S. aureus AIP-III D4A (**57**)	**IC$_{50}$:** 0.49 nM (0.29–0.81 CI)	**IC$_{50}$:** 0.43 nM (0.21–0.89 CI)	**IC$_{50}$:** 0.051 nM (0.023–0.113CI)	**IC$_{50}$:** 0.035 nM (0.012–0.099 CI)	fluorescence	(5)
	**IC$_{50}$:** 0.082 nM (0.047–0.142 CI)	**IC$_{50}$:** 0.060 nM (0.038–0.094 CI)	**IC$_{50}$:** 0.16 nM (0.069–0.39 CI)	**IC$_{50}$:** 0.11 nM (0.066–0.17 CI)	hemolysis	(5)
	**IC$_{50}$:** 0.16 ± 0.01 nM	n. t.	n. t.	n. t.	*β*-lactamase	(8)

Supplementary Table S4. Reported QS interactions of staphylococcal AIPs against *S. epidermidis* (SE) *agr*-I–III.

Species	SE *agr*-I	SE *agr*-II	SE *agr*-III	Assay type	Ref.
S. aureus AIP-I (**1**)	no inhibition at 1000 nM	n. t.	n. t.	HPLC δ-toxin	(9)
	no inhibition at 1000 nM	n. t.	n. t.	fluorescence	(11)
S. aureus AIP-II (**2**)	no inhibition at 1000 nM	n. t.	n. t.	HPLC δ-toxin	(9)
	**IC_{50}:** 62.9 nM (26.1–151 CI)	n. t.	n. t.	fluorescence	(11)
S. aureus AIP-III (**3**)	no inhibition at 1000 nM	n. t.	n. t.	HPLC δ-toxin	(9)
	no inhibition at 1000 nM	n. t.	n. t.	fluorescence	(11)
S. aureus AIP-IV (**4**)	40% inhibition at 1000 nM	n. t.	n. t.	HPLC δ-toxin	(9)
	**IC_{50}:** >1000 nM	n. t.	n. t.	fluorescence	(11)
S. epidermidis AIP-I (**5**)	activation from supernatant	~90% inhibition from supernatant	~90% inhibition from supernatant	fluorescence	(18)
	**EC_{50}:** 196 nM (162–238 CI)	n. t.	n. t.	fluorescence	(11)
S. epidermidis AIP-II (**6**)	~90% inhibition from supernatant	activation from supernatant	no effect from supernatant	fluorescence	(18)
	**IC_{50}:** 9.64 nM (7.99–11.6 CI)	activation at 10 μM	no effect at 10 μM	fluorescence	(11)
S. epidermidis AIP-III (**7**)	~50% inhibition from supernatant	no effect from supernatant	activation from supernatant	fluorescence	(18)
	**IC_{50}:** 34.3 nM (31.4–37.4 CI)	n. t.	n. t.	fluorescence	(11)
S. hominis AIP-I (**10**)	NA	**IC_{50}:** 34 nM (27.3–41.6 CI)	**IC_{50}:** 16 nM (14.5–18.5 CI)	fluorescence	(13)
S. hominis AIP-II (**11**)	**IC_{50}:** 20 nM (18.6–21.5 CI)	**IC_{50}:** 19 nM (16.9–21.2 CI)	**IC_{50}:** 62 nM (51.3–74.4 CI)	fluorescence	(13)
S. hominis AIP-III (**12**)	**IC_{50}:** 4 nM (2.5–7.3 CI)	**IC_{50}:** 3 nM (2.6–4.5 CI)	**IC_{50}:** 3 nM (2.1–5.6 CI)	fluorescence	(13)
S. hominis AIP-IV (**13**)	**IC_{50}:** 237 nM (199.3–281.1 CI)	**IC_{50}:** 93 nM (84.8–102.3 CI)	**IC_{50}:** 28 nM (26.1–29.6 CI)	fluorescence	(13)
S. hominis AIP-V (**14**)	**IC_{50}:** 10 nM (8.8–10.7 CI)	**IC_{50}:** 22 nM (17.9–27.8 CI)	**IC_{50}:** 2 nM (1.5–2.2 CI)	fluorescence	(13)
S. warneri AIP-I (**16**)	**IC_{50}:** 3 nM (2.8–3.4 CI)	**IC_{50}:** 19 nM (16.8–21.2 CI)	n. t.	fluorescence	(14)
S. warneri AIP-II (**17**)	**IC_{50}:** 12 nM (8.6–17.3 CI)	**IC_{50}:** 4 nM (3.2–4.1 CI)	n. t.	fluorescence	(14)
S. aureus AIP-III D4A (**57**)	~25% inhibition at 10 μM	~70% inhibition at 100 nM	~25% inhibition at 10 μM	fluorescence	(11)

MRSA mouse skin infection model data

Supplementary Table S5. Skin lesion size, colony-forming unit (CFU) values and body weight for MRSA mouse skin infection model.

Treatment	Mouse	Skin lesion in mm^2			log_{10} CFU			Body weight in g		
		Day 1	Day 2	Day 4	Day 1	Day 2	Day 4	Day 1	Day 2	Day 4
vehicle	1	71.0	-	-	7.68	-	-	18.6	-	-
	2	76.2	-	-	7.89	-	-	21.4	-	-
	3	62.3	-	-	7.98	-	-	20.8	-	-
	4	69.9	-	-	8.11	-	-	19.6	-	-
	5	88.1	-	-	8.06	-	-	19.1	-	-
	6	69.7	-	-	7.95	-	-	18.9	-	-
	7	74.3	-	-	7.74	-	-	18.0	-	-
	8	79.4	-	-	7.68	-	-	18.6	-	-
	9	63.4	98.3	-	-	7.38	-	18.6	20.6	-
	10	70.5	99.3	-	-	7.89	-	20.8	20.4	-
	11	61.8	70.3	-	-	7.49	-	18.7	20.4	-
	12	52.8	101.3	-	-	7.56	-	20.4	19.0	-
	13	82.2	103.7	64.4	-	-	7.11	19.3	19.9	19.6
	14	73.4	96.5	58.8	-	-	7.76	19.9	18.8	18.6
	15	60.4	96.3	74.7	-	-	7.24	20.5	18.6	18.6
	16	71.0	97.5	58.2	-	-	6.19	19.4	20.9	21.2
2% fusidic acid ointment	1	64.3	76.7	-	-	7.27	-	18.8	18.3	-
	2	82.5	98.4	-	-	6.89	-	18.8	20.5	-
	3	69.1	69.7	-	-	7.18	-	18.9	20.7	-
	4	47.4	66.4	-	-	7.38	-	20.4	19.1	-
	5	55.2	80.5	38.2	-	-	4.12	19.2	18.9	19.5
	6	74.9	81.4	53.9	-	-	5.70	19.1	20.2	21.4
	7	69	88.1	41	-	-	6.12	19.9	18.8	19.0
	8	63.3	70.1	56.5	-	-	6.04	19.3	19.6	19.8
S. simulans AIP-II (**33**)	1	75.5	84.7	-	-	7.88	-	19.1	20.0	-
	2	77.6	91.5	-	-	7.95	-	20.7	19.3	-
	3	111.4	81.3	-	-	8.08	-	19.0	20.2	-
	4	79.6	81.5	-	-	7.51	-	18.5	20.7	-
	5	63.3	94.1	47.7	-	-	5.68	19.5	21.7	22.0
	6	64.6	77.4	41.9	-	-	5.19	20.3	19.3	19.8
	7	51	61	50	-	-	5.30	21.3	19.1	19.4
	8	64.5	69	52.6	-	-	5.04	19.7	18.6	18.8

Bacterial strains and *agrD* sequencing

Supplementary Table S6. Bacterial strains used in this study.

Name	Characteristics	Reference
P3-*blaZ*/*pagrC*-I	*S. aureus* RN10829 reporter expressing AgrC-I	(1)
P3-*blaZ*/*pagrC*-II	*S. aureus* RN10829 reporter expressing AgrC-II	(1)
P3-*blaZ*/*pagrC*-III	*S. aureus* RN10829 reporter expressing AgrC-III	(1)
P3-*blaZ*/*pagrC*-IV	*S. aureus* RN10829 reporter expressing AgrC-IV	(1)
AH3408	*S. epidermidis* ATCC1228(AH1740) + pCM40 (ermR, arP3_sGFP, type-I)	(18)
AH3623	*S. epidermidis* 1457(AH1738) ica::DHFR + pCM40 (ermR, arP3_sGFP, type-II)	(18)
AH3409	*S. epidermidis* Fey 8247+ pCM40 (ermR, arP3_sGFP, type-III)	(18)
AH4031	*S. lugdunensis* N920143+ pCM40 (ermR, arP3_sGFP, type-I)	(19)
AH1677	*S. aureus* LAC/pDB59 (*agr*-I P3::YFP-reporter strain)	(20)
AH430	*S. aureus* 502A/pDB59 (*agr*-II P3::YFP-reporter strain)	(20)
AH1747	*S. aureus* MW2/pDB59 (*agr*-III P3::YFP-reporter strain)	(20)
AH1872	*S. aureus* MNEV/pDB59 (*agr*-IV P3::YFP-reporter strain)	(20)
JE2/pALC1741	*S. aureus* JE2/pALC1741 (*agr*-I spa::GFP-reporter strain)	This study
5927201	*S. pasteuri* human nose isolate	This study
102 L88	*S. succinus* horse isolate	This study
2934 c-30869-41	*S. cohnii* isolate from skim milk	This study
AW 0837	*S. devriesei* isolate from bovine milk	This study
KS 155	*S. equorum* isolate from bovine milk	This study
DV 026	*S. equorum* isolate from bovine milk	This study

Supplementary Table S7. Primer used for *agrD* sequencing.

Species	Forward primer	Reverse primer
S. pasteuri	ccattgttagataaaaacttacagcc	gaaaaggaaacgaatactctccattg
S. cohnii	ccgtctgaaataatgtataccttgtc	ttatgtctacaattagactacttaaccac
S. succinus	gtcaaagtagcaagccatgtag	cattagcaatcattggctttgttg
S. equorum (*agr-I*)	gcagcctataccacaacg	gatacgacacccatgaagc
S. equorum (*agr-II*)	cctggttaatcgggtatatgg	cccaataatggcaatataatccc
S. devriesei	ggttgatgcttatagtaatcttgg	ccactaaaatggctaaataatccc

Supplementary Table S8. AgrD sequences used for AIP identification.

Species	AgrD sequence	
S. pasteuri	METLVNLFFKFFTSIMEFVGLVAGANPCAGYFDEPEVPDELTKLYE	sequenced in this study
S. cohnii	MNIFESILTIFAKFFTFIGTISSVKPCTGFVDEPEIPKELTDLYE	sequenced in this study
S. succinus	MTILESLLTLITKFFSVLGATAGALPCGGFFDEPEVPSEITKLHE	sequenced in this study
S. equorum (*agr-I*)	MHIFESIFSIIAKFFTVLGAVAGARPCYGYFDETEVPKEITELYE	sequenced in this study
S. equorum (*agr-II*)	MHIFESIFSLIAKFFSTLGAVAGLSPCGGYFDEPEVPKEITDLYE	sequenced in this study
S. devriesei	MFITDLFFKFFAAILETLGNVAAYKPCFGYFDEAEVPEELTNLKR	sequenced in this study

4. Methods and protocols

4.1 General information

Abbreviations of chemicals

2-(1*H*-Benzotriazol-1-yl)-1,1,3,3-tetramethyluronoium hexafluorophosphate (HBTU), 2-(1*H*-7-azabenzotriazol-1-yl)-1,1,3,3-tetramethyluronoium hexafluorphosphate (HATU), [ethyl cyano(hydroxyimino)acetato-O^2]tri-1-pyrrolidinylphosphonium hexafluorophosphate (PyOxim), trifluoroacetic acid (TFA), *N*,*N*-dimethylformamide (DMF), tetrahydrofuran (THF), *N*-methylmorpholine (NMM), hexafluoroisopropanol (HFIP), 1,4-dithiothreitol (DTT), tris(2-carboxyethyl)phosphine hydrochloride (TCEP·HCl), *N*-methyl-2- pyrrolidone (NMP), guanidine hydrochloride (Gdn·HCl), dimethyl sulfoxide (DMSO), 9-fluorenylmethyloxycarbonyl (Fmoc), 2,3-diaminopropionic acid (Dap), *N*,*N*'-diisopropylcarbodiimide (DIC), *N*-methylimidazole (NMI), *sec*-isoamyl mercaptan (SIT), *tert*-butyldimethylsilyl (TBDMS), 4-(methylamino)benzoic acid (MeDbz), *N*-acyl-benzimidazolinone (Nbz), 1,8-biazabicyclo[5.4.0]undec-7-ene (DBU), tetrabutylammonium fluoride (TBAF), chloramphenicol (CAM), erythromycin (ERM).

Reagents and materials

Bacteria were cultured in tryptic soy broth (TSB) medium and on tryptic soy agar (TSA) plates (both Oxoid) supplemented with 10 µg/ml CAM or ERM (Sigma-Aldrich) when appropriate. *N*α-Fmoc protected and α-*N*-Boc protected amino acids, HBTU and HATU were obtained from ChemImpex and PepChem. Aminomethyl ChemMatrix resin was obtained from PCAS BioMatrix. Aminomethyl PEGA resin, *N*,*N*-diisopropylethylamine, 4-nitrophenylchloroformate, maleic acid (qNMR grade) and nitrocefin were obtained from Sigma-Aldrich. All other chemicals used were obtained in the highest available purity from CombiBlocks or IrisBiotech. All solvents used were of analytical grade and purchased from Fisher Scientific. Manual solid-phase peptide synthesis was performed in polypropylene syringes equipped with fritted disks, purchased from Torviq.

Compound analysis and purification

Analytical ultra-performance liquid chromatography (UPLC) analyses were performed on a C18 Agilent InfinityLab Poroshell 120 column (2.7 µm, 100 × 3.0 mm) using an Agilent 1260 Infinity II series system equipped with a diode array UV detector. A gradient with eluent A (water–MeCN–TFA, 95:5:0.1, v/v/v) and eluent B (0.1% TFA in MeCN) rising linearly from 0 to 50% of B over 10.0 min at a flow rate of 1.2 mL min^{-1} was applied to determine the purity of peptides ($\lambda = 215$ nm). UPLC-mass spectrometry (MS) analyses were performed on a Phenomenex Kinetex column (1.7 µm, 100 Å, 50 × 2.10 mm) using a Waters Acquity system. A gradient with eluent C (0.1% HCOOH in water) and eluent D (0.1% HCOOH in MeCN) rising linearly from 0 to 95% of D over 5.20 min at a flow rate of 0.6 mL min^{-1} was applied to analyse reaction mixtures. Preparative high-performance liquid chromatography (HPLC) purification was performed on a C18 Phenomenex Luna column (5 µm, 100 Å, 250 × 20 mm) using an Agilent 1260 LC system equipped with a diode array ultraviolet detector. Various gradients with eluent A and eluent B at a flow rate of 20 mL min^{-1} were applied for the purification. Fractions containing the purified target peptide were identified using UPLC-MS or matrix assisted laser desorption ionization–time of flight

mass spectrometry (MALDI-TOF MS). MALDI-TOF mass spectra were recorded with a Bruker microflex bench-top MALDI using a matrix of alpha-cyano-4-hydroxycinnamic acid or 2,5-dihydroxybenzoic acid in water/MeCN (1:1, v/v) containing 0.1% TFA. The observed *m/z* corresponded to the monoisotopic ions, unless otherwise stated. Nuclear magnetic resonance (NMR) spectra were recorded at 298 K using a Bruker Avance III HD (^{1}H NMR and ^{13}C NMR recorded at 600 MHz and 150 MHz, respectively). Chemical shifts are reported in parts per million (ppm) relative to the deuterated solvent peak of DMSO-d_6 (δ_H = 2.50 ppm; δ_C = 39.52 ppm) or $CDCl_3$ (δ_H = 7.26 ppm; δ_C = 77.16 ppm) as internal standard.

Concentration determination of DMSO stock solutions

Concentrations of DMSO stock solutions for compounds were determined by quantitative NMR relative to the signal (δ_H = 6.24 ppm, 2H) of the internal standard maleic acid (qNMR grade).

Plate reader for reporter strain assays

For fluorescence-based reporter strain assays, fluorescence (for YFP: excitation 500 nm and emission 541 nm; for sGFP: excitation 479 nm and emission 520 nm; automatic gain adjustment) was measured along with optical density at λ = 600 nm (OD600) using a BioTek Synergy H1 microplate reader with Gen5TM software.

Statistical analysis

All statistical analyses were performed using GraphPad Prism 10.4 software. *P* values were determined using one-way analysis of variance (ANOVA) and Dunnet's test. *P* values < 0.05 considered significant.

4.2 Synthetic procedures

General protocol for automated peptide synthesis

Automated peptide synthesis was carried out on a Biotage SyroWave™ synthesizer using standard Fmoc SPPS chemistry. The following Fmoc-protected amino acids with side chain protecting groups were used: Fmoc-Ala-OH, Fmoc-Asn(Trt)-OH, Fmoc-Cys(Trt)-OH, Fmoc-Cys(*S*t-Bu)-OH, Fmoc-Dap(Alloc)-OH, Fmoc-Gly-OH, Fmoc-Ile-OH, Fmoc-Leu-OH, Fmoc-Phe-OH, Fmoc-Pro-OH, Fmoc-Ser(*t*-Bu)-OH, Fmoc-Ser(TBDMS)-OH, Fmoc-Thr(*t*-Bu)-OH, Fmoc-Trp(Boc)-OH, Fmoc-Tyr(*t*-Bu)-OH, and Fmoc-Val-OH. The following amino acids with side chain protecting groups were used as *N*-terminal amino acids: Ac-OH, Boc-Ala-OH, Boc-Asn(Trt)-OH, Boc-Asp(O*t*-Bu)-OH, Boc-Arg(Pbf)-OH, Me_2N-Cys(Trt)-OH,(21) Boc-Lys(Boc)-OH, Boc-Ser(*t*-Bu)-OH, Boc-Thr(*t*-Bu)-OH and Boc-Tyr(*t*-Bu)-OH.

SPPS was performed on 0.02 mmol (method A) or 0.04 mmol (method B) scale using MeDbz-Gly-ChemMatrix resin(8) or on 0.04 mmol scale using preloaded chlorotrityl (Cl-Trt) resin. Fmoc deprotection was performed in two stages: 1) piperidine in DMF (2:3, v/v) for 3 min and 2) piperidine in DMF (1:4, v/v) for 12 min. The deprotection was followed by washing with DMF (2 × 45 s), CH_2Cl_2 (1 × 45 s), and DMF (2 × 45 s). The first coupling reaction on MeDbz-Gly-ChemMatrix resin was performed as double coupling using Fmoc-AA-OH (5.00 equiv to the resin loading), HATU (4.90 equiv) and *i*-Pr_2NEt in NMP (10.0 equiv, 2.0 M) in DMF (final concentration = 0.2 M for

Fmoc-AA-OH) for 90 min. Standard coupling reactions were performed as double couplings with Fmoc-AA-OH (5.00 equiv to the resin loading), HBTU (4.90 equiv) and *i*-Pr_2NEt in NMP (10.0 equiv, 2.0 M) in DMF (final concentration = 0.2 M for Fmoc-AA-OH) for 40 min for each coupling. The last amino acid was incorporated as Boc-AA-OH.

General procedure for *N*-acyl-benzimidazolinone (Nbz) formation

After automated peptide elongation, the peptidyl-MeDbz-Gly-ChemMatrix resin (1.00 equiv) was transferred into a polypropylene syringe equipped with a fritted disk using CH_2Cl_2 and the resin was then washed with CH_2Cl_2 (5 × 1 min). A solution of 4-nitrophenyl-chloroformate (5.00 equiv) in CH_2Cl_2 (concentration = 0.1 M) was added to the resin and the suspension was agitated for 30 min. The resin was then washed with CH_2Cl_2 (2 × 1 min) and the procedure was repeated. The resin was then washed with CH_2Cl_2 (3 × 1 min) and DMF (3 × 1 min) and a solution of *i*-Pr_2NEt (25.0 equiv) in DMF (0.5 M) was added to the resin. After 15 min, the resin was washed with DMF (3 × 1 min) and the procedure was repeated. The resin was then washed with DMF (3 × 1 min), *i*-Pr_2NEt in DMF (5%, v/v) (3 × 1 min), DMF (3 × 1 min), MeOH (3 × 1 min), and CH_2Cl_2 (3 × 1 min) and dried under high vacuum.

Method A: General procedure for on-resin cleavage-inducing cyclization

Dried peptidyl-MeNbz-Gly-ChemMatrix resin **S4** (0.02 mmol, 1.00 equiv) was placed in a polypropylene syringe equipped with a fritted disk and treated with a deprotection/cleavage cocktail (2.0 mL, TFA–*i*-Pr_3SiH–water, 94:3:3, v/v/v) for 1 h and the TFA cocktail removed from the resin. The resin **S5** was washed with CH_2Cl_2 (3 × 1 min), DMF (3 × 1 min), and CH_2Cl_2 (3 × 1 min) and dried under suction for several minutes. The cyclization buffer (5.0 mL, phosphate buffer (0.2 M, pH = 6.8)–MeCN, 1:1, v/v) was added to the resin (final concentration = 4.0 mM) and the suspension was agitated at 50 °C for 2 h. The solution was removed from the resin, collected and the resin was rinsed with fresh cyclisation buffer. The combined peptide-containing solution was purified by preparative RP-HPLC. Fractions containing pure peptide were lyophilized to afford the desired cyclized peptide.

Method B: General procedure for solution cyclization

The procedure was adapted from a previously published protocol.(22) Dried peptidyl-MeNbz-Gly-ChemMatrix resin **S4** (0.04 mmol, 1.00 equiv) was placed in a polypropylene syringe equipped with a fritted disk and treated with a solution of 3-mercaptopropionic acid ethyl ester (50.6 μL, 0.40 mmol, 10.0 equiv) and *i*-Pr_2NEt (69.8 μL, 0.40 mmol, 10.0 equiv) in DMF (3.0 mL). After overnight incubation, the peptide containing solution was removed from the resin and the resin washed twice with DMF (2.0 mL). The combined organic phase was concentrated to dryness under pressure and the remaining residue was treated with a deprotection cocktail (2.0 mL, TFA–*i*-Pr_3SiH–water, 94:3:3, v/v/v) for 2 h. The reaction mixture was concentrated under a stream of nitrogen and precipitated by addition of ice-cold diethyl ether. The crude peptide thioester **S6** was lyophilized and used in the next step without further purification. Crude **S6** (10.0 μmol) was dissolved in cyclization buffer (10.0 mL, guanidinium hydrochloride (6 M) in phosphate buffer (0.1 M, pH = 7.0)–MeCN, 4:1, v/v) with a final concentration of 1.0 mM and the reaction mixture was agitated at 37 °C. After for 2 h, the reaction mixture was purified by preparative RP-HPLC. Fractions containing pure peptide were lyophilized to afford the desired cyclized peptide.

5. Synthetic peptides

5.1 Native AIPs

The following peptides have been characterized elsewhere: *S. aureus* AIP-I–IV (**1**–**4**),(8) *S. lugdunensis* AIP-II (**8**), *S. hominis* AIP-III (**12**), *S. haemolyticus* AIP-I (**15**), *S. warneri* AIP-I (**16**), *S. saprophyticus* AIP-I (**19**), *S. hyicus* AIP-I (**26**), *S. chromogenes* AIP-I–III (**27**–**29**), *S. schleiferi* AIP-I (**30**), *S. simulans* AIP-I (**32**) and *S. vitulinus* AIP-I (**35**).(1)

***S. epidermidis* AIP-I (5)**

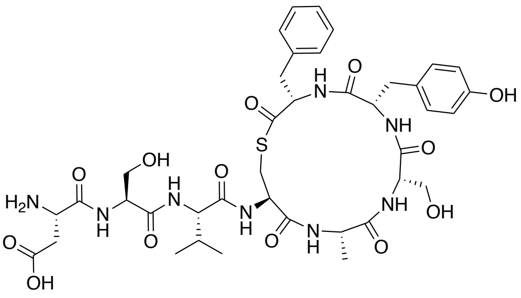

$C_{39}H_{52}N_8O_{13}S$
MW: 872.95

The peptide was synthesized according to general method B for solution cyclization (section 4.5). Purification by preparative RP-HPLC afforded *S. epidermidis* AIP-I (**5**) as a fluffy white solid after lyophilization. Purity >95% determined by UPLC (λ = 215 nm). **UPLC-MS** (ESI) *m/z* calcd for $[M+H]^+$ $C_{39}H_{53}N_8O_{13}S^+$: 873.35, found 873.32. **^{1}H NMR** (600 MHz, DMSO-d_6) δ = 0.83 (t, J = 7.1 Hz, 6H), 1.21 (d, J = 7.3 Hz, 3H), 1.95–2.04 (m, 1H), 2.58–2.67 (m, 2H), 2.67–2.72 (m, 1H), 2.73–2.82 (m, 2H), 2.90–2.95 (m, 1H), 3.16 (dd, J = 12.8, 3.8 Hz, 1H), 3.28 (dd, J = 14.0, 3.9 Hz, 1H), 3.54–3.66 (m, 4H), 4.05–4.07 (m, 2H), 4.15–4.23 (m, 3H), 4.24–4.30 (m, 2H), 4.41–4.46 (m, 1H), 5.04–5.11 (m, 1H), 5.13–5.20 (m, 1H), 6.62 (d, J = 8.5 Hz, 2H), 6.85 (d, J = 8.5 Hz, 2H), 7.01–7.04 (m, 2H), 7.16–7.20 (m, 1H), 7.21–7.24 (m, 2H), 7.83 (d, J = 8.7 Hz, 1H), 7.91 (d, J = 8.8 Hz, 1H), 8.04 (d, J = 7.5 Hz, 1H), 8.21 (d, J = 8.3 Hz, 1H), 8.45 (d, J = 7.8 Hz, 1H), 8.58 (d, J = 7.6 Hz, 1H), 8.88 (d, J = 8.2 Hz, 1H), 9.22 (s, 1H).

***S. epidermidis* AIP-II (6)**

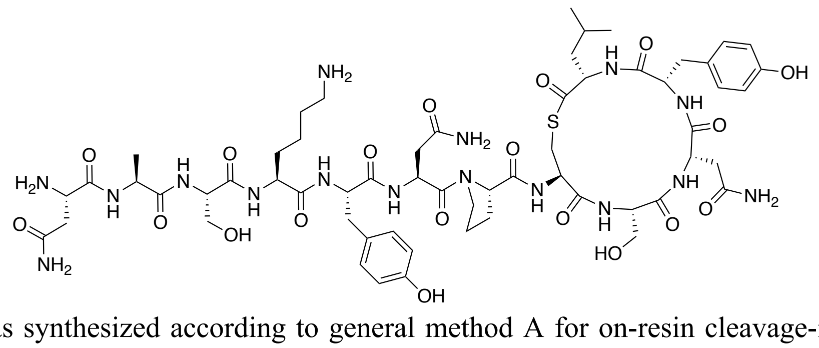

$C_{59}H_{86}N_{16}O_{19}S$
MW: 1355.49

The peptide was synthesized according to general method A for on-resin cleavage-inducing cyclization (section 4.5). Purification by preparative RP-HPLC afforded *S. epidermidis* AIP-II (**6**) as a fluffy white solid after lyophilization. Purity >95% determined by UPLC (λ = 215 nm). **UPLC-MS** (ESI) *m/z* calcd for $[M+2H]^{2+}$ $C_{59}H_{88}N_{16}O_{19}S^{2+}$: 678.31, found 678.61; $[M+H]^+$ $C_{59}H_{87}N_{16}O_{19}S^+$: 1355.61, found 1355.73. **^{1}H NMR** (600 MHz, DMSO-d_6) δ = 0.75 (d, J = 6.5 Hz, 3H), 0.83 (d, J = 6.6 Hz, 3H), 1.18–1.27 (m, 5H), 1.29–1.37 (m, 1H), 1.41–1.53 (m, 3H), 1.54–1.64 (m, 2H), 1.64–1.71 (m, 1H), 1.80–1.89 (m, 3H), 1.93–2.01 (m, 1H), 2.37 (dd, J = 15.6, 6.3 Hz, 1H), 2.51–2.65 (m, 5H), 2.66–2.76 (m, 3H), 2.78–2.90 (m, 3H), 2.94 (dd, J = 13.7, 7.6 Hz, 1H), 3.13 (dd, J = 12.9,

4.1 Hz, 1H), 3.49 (dd, J = 11.2, 4.4 Hz, 1H), 3.52–3.66 (m, 5H), 4.05–4.15 (m, 2H), 4.14–4.23 (m, 3H), 4.27–4.35 (m, 3H), 4.36–4.46 (m, 3H), 4.77 (q, J = 7.2 Hz, 1H), 4.92 (s, 1H), 5.16 (s, 1H), 6.62 (d, J = 8.3 Hz, 2H), 6.65 (d, J = 8.3 Hz, 2H), 6.92 (s, 1H), 6.93–6.97 (m, 4H), 7.01 (s, 1H), 7.26 (s, 1H), 7.47 (s, 1H), 7.51 (s, 1H), 7.62–7.75 (m, 4H), 7.84 (d, J = 8.0 Hz, 1H), 7.90–7.96 (m, 2H), 8.04 (d, J = 8.9 Hz, 1H), 8.06–8.12 (m, 4H), 8.15 (d, J = 7.7 Hz, 1H), 8.19 (d, J = 8.0 Hz, 1H), 8.27 (d, J = 7.7 Hz, 1H), 8.56 (d, J = 8.4 Hz, 1H), 8.60 (d, J = 7.4 Hz, 1H), 9.20 (s, 2H).

***S. epidermidis* AIP-III (7)**

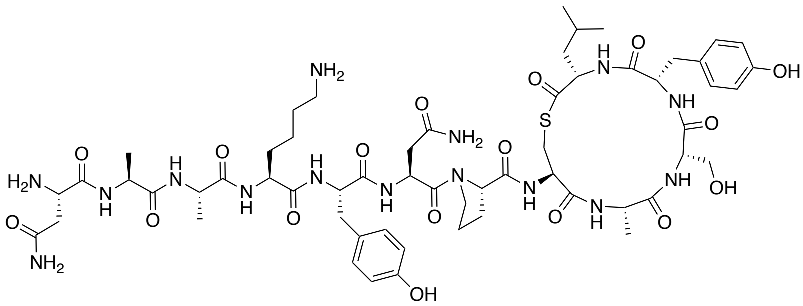

$C_{58}H_{85}N_{15}O_{17}S$
MW: 1296.47

The peptide was synthesized according to general method A for on-resin cleavage-inducing cyclization (section 4.5). Purification by preparative RP-HPLC afforded *S. epidermidis* AIP-III (**7**) as a fluffy white solid after lyophilization. Purity 95% determined by UPLC (λ = 215 nm). **UPLC-MS** (ESI) *m/z* calcd for $[M+2H]^{2+}$ $C_{58}H_{87}N_{15}O_{17}S^{2+}$: 648.81, found 649.15; $[M+H]^{+}$ $C_{58}H_{86}N_{15}O_{17}S^{+}$: 1296.60, found 1296.72. **^{1}H NMR** (600 MHz, DMSO-d_6) δ = 0.75 (d, J = 6.5 Hz, 3H), 0.82 (d, J = 6.6 Hz, 3H), 1.16–1.26 (m, 11H), 1.26–1.33 (m, 1H), 1.42–1.53 (m, 3H), 1.53–1.59 (m, 3H), 1.80–1.88 (m, 3H), 1.96–2.04 (m, 1H), 2.37 (dd, J = 15.5, 6.2 Hz, 1H), 2.54–2.71 (m, 4H), 2.71–2.78 (m, 2H), 2.80–2.89 (m, 3H), 2.90–2.95 (m, 1H), 3.11 (dd, J = 12.9, 3.8 Hz, 1H), 3.53–3.64 (m, 4H), 4.04–4.10 (m, 2H), 4.13–4.21 (m, 4H), 4.24 (t, J = 7.2 Hz, 1H), 4.25–4.30 (m, 1H), 4.30–4.36 (m, 2H), 4.44 (td, J = 7.9, 5.3 Hz, 1H), 4.77 (q, J = 7.3 Hz, 1H), 5.17 (s, 1H), 6.60 (d, J = 8.5 Hz, 2H), 6.64 (d, J = 8.5 Hz, 2H), 6.91–6.95 (m, 3H), 6.97 (d, J = 8.5 Hz, 2H), 7.26 (s, 1H), 7.49 (s, 1H), 7.66–7.75 (m, 5H), 7.77 (d, J = 8.7 Hz, 1H), 7.82 (d, J = 8.0 Hz, 1H), 7.96 (d, J = 7.6 Hz, 1H), 8.04 (d, J = 7.9 Hz, 1H), 8.08–8.11 (m, 3H), 8.13 (d, J = 7.3 Hz, 1H), 8.18 (d, J = 7.9 Hz, 1H), 8.38 (d, J = 7.7 Hz, 1H), 8.59 (d, J = 7.4 Hz, 1H), 8.68 (d, J = 8.3 Hz, 1H), 9.14–9.27 (m, 2H).

***S. lugdunensis* AIP-I (8)**

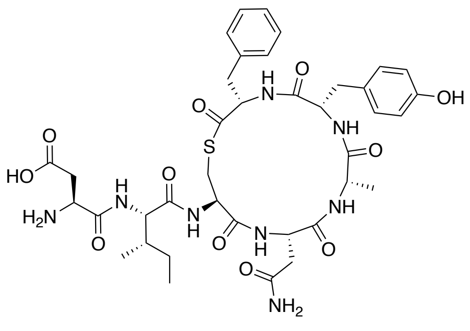

$C_{38}H_{50}N_8O_{11}S$
MW: 826.92

The peptide was synthesized as followed (Supporting Scheme S2). The fully protected linear peptide **S8** was synthesized in 40.0 μmol scale on Cl-Trt polystyrene resin **S7** preloaded with Fmoc-Phe-OH (0.69 mmol/g) using the general procedures for automated SPPS. After completed peptide elongation, the peptidyl resin **S8** was transferred into a polypropylene syringe equipped with a fritted disk using CH_2Cl_2 and washed with DMF (3 × 1 min). A solution of NMM (22.1 μL, 0.20 mmol, final concentration = 0.1 M) in a mixture of β-mercaptoethanol–DMF (2.0 mL, 1:4, v/v) was added to the peptidyl resin **S8** and the resin was agitated overnight at room temperature. The next day, the

thiol-containing solution was removed by suction and the resin was washed with DMF (3 × 1 min), MeOH (3 × 1 min) and CH_2Cl_2 (3 × 1 min) and dried under suction for 15 min. The dried resin was treated with a solution of HFIP (0.4 mL) in CH_2Cl_2 (1.6 mL) for 15 min at room temperature to cleave the partially-protected peptide **S9** from the resin. The cleavage solution was removed from the resin, collected and a fresh HFIP–CH_2Cl_2 solution (2.0 mL, 1:4, v/v) was added to the resin. After 15 min, the cleavage solution was removed from the resin, collected and the resin rinsed with CH_2Cl_2 (2.0 mL). The combined cleavage solution was concentrated to dryness under reduced pressure to yield the partially protected peptide **S9**, which was used without further purification.

The crude peptide **S9** (0.04 mmol based on the resin loading) was dissolved in anhydrous DMF (5.0 mL) under nitrogen atmosphere and added dropwise to a solution of PyOxim (42.2 mg, 0.08 mmol, 2.00 equiv) and *i*-Pr_2NEt (48.6 µL, 0.28 mmol, 7.00 equiv) in anhydrous DMF (35.0 mL). The reaction mixture was stirred overnight at room temperature and after full consumption of **S9** was confirmed by UPLC-MS, the reaction was reduced to dryness under reduced pressure. The remaining residue was treated with a deprotection cocktail (3.0 mL, TFA–*i*-Pr_3SiH–water, 94:3:3, v/v/v) for 2 h and subsequently concentrated under a stream of nitrogen followed by precipitation in ice-cold diethyl ether. Purification by preparative RP-HPLC afforded *S. lugdunensis* AIP-I (**8**) as a fluffy white solid after lyophilization. Purity >95% determined by UPLC (λ = 215 nm). **UPLC-MS** (ESI) *m/z* calcd for $[M+H]^+$ $C_{38}H_{51}N_8O_{11}S^+$: 827.34, found 827.40. **^{1}H NMR** (600 MHz, DMSO-d_6) δ = 0.79–0.83 (m, 6H), 1.03–1.11 (m, 1H), 1.17 (d, J = 6.9 Hz, 3H), 1.37–1.47 (m, 1H), 1.65–1.73 (m, 1H), 2.30 (dd, J = 15.7, 5.2 Hz, 1H), 2.50–2.59 (m, 2H), 2.64 (dd, J = 13.6, 7.0 Hz, 1H), 2.70 (dd, J = 17.3, 3.8 Hz, 1H), 2.76–2.86 (m, 2H), 2.92 (t, J = 12.0 Hz, 1H), 3.15 (dd, J = 12.7, 4.0 Hz, 1H), 3.27 (1H) signal partially overlapping with HDO peak, 3.98–4.04 (m, 1H), 4.06 (q, J = 7.6 Hz, 1H), 4.12–4.20 (m, 1H), 4.21 (t, J = 7.2 Hz, 1H), 4.31 (td, J = 9.2, 4.1 Hz, 1H), 4.43 (dt, J = 8.4, 5.8 Hz, 1H), 4.48 (ddd, J = 11.8, 8.1, 4.0 Hz, 1H), 6.64 (d, J = 8.2 Hz, 2H), 6.87 (d, J = 8.2 Hz, 2H), 6.91 (s, 1H), 7.03 (d, J = 7.4 Hz, 2H), 7.15–7.19 (m, 1H), 7.19–7.25 (m, 2H), 7.40 (s, 1H), 7.86 (d, J = 8.7 Hz, 1H), 8.13 (d, J = 8.5 Hz, 1H), 8.25 (d, J = 7.7 Hz, 1H), 8.45 (d, J = 8.5 Hz, 1H), 8.59 (d, J = 8.1 Hz, 1H), 8.80 (d, J = 8.4 Hz, 1H), 9.23 (s, 1H).

S. hominis **AIP-I (10)**

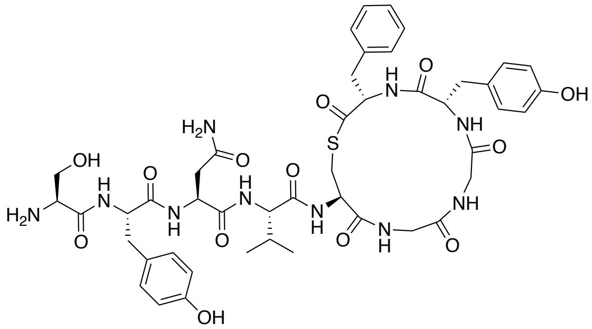

$C_{46}H_{58}N_{10}O_{13}S$
MW: 991.09

The peptide was synthesized according to general method A for on-resin cleavage-inducing cyclization (section 4.5). Purification by preparative RP-HPLC afforded *S. hominis* AIP-I (**10**) as a fluffy white solid after lyophilization. Purity >95% determined by UPLC (λ = 215 nm). **UPLC-MS** (ESI) *m/z* calcd for $[M+H]^+$ $C_{46}H_{59}N_{10}O_{13}S^+$: 991.40, found 991.57. **^{1}H NMR** (600 MHz, DMSO-d_6) δ = 0.81 (d, J = 6.8 Hz, 3H), 0.83 (d, J = 6.8 Hz, 3H), 1.95–2.07 (m, 1H), 2.32 (dd, J = 14.3, 10.6 Hz, 1H), 2.42 (dd, J = 15.5, 7.4 Hz, 1H), 2.57 (dd, J = 15.6, 6.2 Hz, 1H), 2.62–2.70 (m, 2H), 2.76 (dd, J = 13.0, 10.5 Hz, 1H), 2.88–2.98 (m, 2H), 3.22 (dd, J = 13.0, 4.8 Hz, 1H), 3.34 (2H) signal partially overlapping with HDO peak, 3.43–3.49 (m, 1H), 3.60–3.67 (m, 1H), 3.73–3.82 (m,

3H), 4.12–4.19 (m, 2H), 4.22 (dd, J = 8.8, 5.9 Hz, 1H), 4.41 (ddd, J = 10.4, 7.7, 5.0 Hz, 1H), 4.53 (td, J = 8.9, 3.9 Hz, 1H), 4.58–4.65 (m, 2H), 5.43–5.49 (m, 1H), 6.60–6.65 (m, 4H), 6.89 (d, J = 8.5 Hz, 2H), 6.96 (s, 1H), 7.05 (d, J = 8.5 Hz, 2H), 7.20–7.26 (m, 3H), 7.27–7.32 (m, 2H), 7.42 (s, 1H), 7.76 (d, J = 8.9 Hz, 1H), 7.91–8.05 (m, 3H), 8.26–8.29 (m, 2H), 8.30–8.33 (m, 1H), 8.35 (dd, J = 8.2, 4.4 Hz, 1H), 8.39–8.44 (m, 2H), 8.54 (d, J = 8.3 Hz, 1H), 9.18 (s, 1H), 9.19 (s, 1H).

***S. hominis* AIP-II (11)**

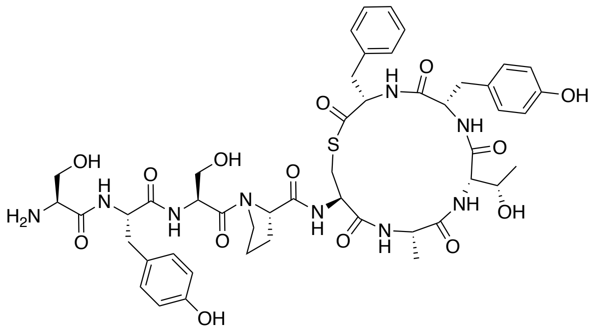

$C_{48}H_{61}N_9O_{14}S$
MW: 1020.13

The peptide was synthesized according to general method A for on-resin cleavage-inducing cyclization (section 4.5). Purification by preparative RP-HPLC afforded *S. hominis* AIP-II (**11**) as a fluffy white solid after lyophilization. Purity >95% determined by UPLC (λ = 215 nm). **HRMS** (MALDI) m/z calcd for $[M+H]^+$ $C_{48}H_{62}N_9O_{14}S^+$: 1020.4130, found 1020.4133. **^{1}H NMR** (600 MHz, DMSO-d_6) δ = 0.97 (d, J = 6.3 Hz, 3H), 1.27 (d, J = 7.3 Hz, 3H), 1.77–1.93 (m, 3H), 2.03–2.11 (m, 1H), 2.64–2.78 (m, 4H), 2.87–2.98 (m, 2H), 3.15–3.24 (m, 2H), 3.58–3.72 (m, 4H), 3.73–3.82 (m, 2H), 3.96–4.01 (m, 1H), 4.01–4.06 (m, 1H), 4.12–4.17 (m, 1H), 4.18–4.25 (m, 1H), 4.26–4.32 (m, 2H), 4.38 (dd, J = 8.4, 4.2 Hz, 1H), 4.53–4.64 (m, 2H), 5.02 (t, J = 5.8 Hz, 1H), 5.10 (d, J = 5.1 Hz, 1H), 5.46 (t, J = 4.8 Hz, 1H), 6.61–6.69 (m, 4H), 6.82–6.86 (m, 2H), 6.92 (d, J = 8.4 Hz, 2H), 7.03 (d, J = 8.5 Hz, 1H), 7.13–7.17 (m, 3H), 7.75 (d, J = 8.0 Hz, 1H), 7.86 (d, J = 9.8 Hz, 1H), 8.04 (s, 4H), 8.13 (d, J = 8.4 Hz, 1H), 8.30–8.36 (m, 1H), 8.50 (d, J = 8.1 Hz, 1H), 8.95 (d, J = 7.6 Hz, 1H), 9.21 (s, 1H), 9.24 (s, 1H).

***S. hominis* AIP-IV (13)**

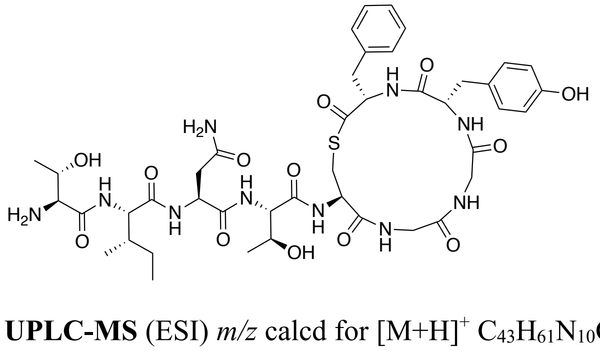

$C_{43}H_{60}N_{10}O_{13}S$
MW: 957.07

The peptide was synthesized according to general method A for on-resin cleavage-inducing cyclization (section 4.5). Purification by preparative RP-HPLC afforded *S. hominis* AIP-IV (**13**) as a fluffy white solid after lyophilization. Purity >95% determined by UPLC (λ = 215 nm). **UPLC-MS** (ESI) m/z calcd for $[M+H]^+$ $C_{43}H_{61}N_{10}O_{13}S^+$: 957.41, found 957.73. **^{1}H NMR** (600 MHz, DMSO-d_6) δ = 0.82 (t, J = 7.4 Hz, 3H), 0.86 (d, J = 6.7 Hz, 3H), 1.00 (d, J = 6.3 Hz, 3H), 1.03–1.12 (m, 0H), 1.14 (d, J = 6.3 Hz, 3H), 1.47 (ddt, J = 10.8, 7.4, 3.3 Hz, 1H), 1.68–1.77 (m, 1H), 2.33 (dd, J = 14.3, 10.4 Hz, 1H), 2.43 (dd, J = 15.6, 6.8 Hz, 1H), 2.58 (dd, J = 15.6, 7.0 Hz, 1H), 2.64 (dd, J = 14.2, 4.3 Hz, 1H), 2.81 (dd, J = 13.1, 10.2 Hz, 1H), 2.91 (dd, J = 14.1, 11.0 Hz, 1H), 3.23 (dd, J = 13.0, 4.8 Hz, 1H), 3.33–3.40 (m, 1H), 3.46 (dd, J = 14.3, 6.3 Hz, 1H), 3.65–3.69 (m, 2H), 3.77–3.84 (m, 2H), 4.05–4.19 (m, 4H), 4.30 (dd, J = 8.7, 7.0 Hz, 1H), 4.44 (ddd, J = 10.1, 7.8, 4.9 Hz, 1H), 4.56–4.66 (m, 2H), 4.87 (d, J = 5.2 Hz, 1H), 5.54 (d, J = 5.0 Hz, 1H), 6.62 (dd, J = 8.4, 2.0 Hz, 2H), 6.88 (d, J = 8.5 Hz, 2H), 6.96 (s, 1H), 7.18–7.26 (m, 3H), 7.27–7.32 (m, 2H), 7.45 (s, 1H), 7.65 (d, J = 8.5 Hz, 1H),

7.94–8.09 (m, 5H), 8.26 (t, *J* = 5.6 Hz, 1H), 8.29 (d, *J* = 9.2 Hz, 1H), 8.35 (d, *J* = 7.4 Hz, 2H), 8.40 (d, *J* = 7.9 Hz, 1H), 8.46 (d, *J* = 8.7 Hz, 1H), 9.19 (s, 1H).

***S. hominis* AIP-V (14)**

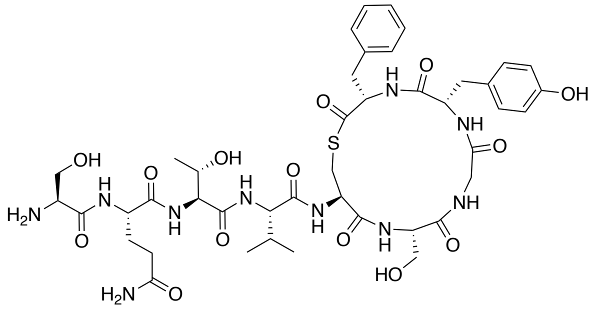

$C_{43}H_{60}N_{10}O_{14}S$
MW: 973.07

The peptide was synthesized according to general method A for on-resin cleavage-inducing cyclization (section 4.5). Purification by preparative RP-HPLC afforded *S. hominis* AIP-V (**14**) as a fluffy white solid after lyophilization. Purity >95% determined by UPLC (λ = 215 nm). **HRMS** (MALDI) *m/z* calcd for $[M+H]^+$ $C_{43}H_{61}N_{10}O_{14}S^+$: 973.4083, found 973.4096. **^{1}H NMR** (600 MHz, DMSO-d_6) δ = 0.81–0.85 (m, 6H), 1.04 (d, *J* = 6.3 Hz, 3H), 1.77–1.85 (m, 1H), 1.88–2.01 (m, 2H), 2.09–2.21 (m, 2H), 2.31 (dd, *J* = 14.4, 10.8 Hz, 1H), 2.62–2.71 (m, 2H), 2.92 (dd, *J* = 14.2, 11.1 Hz, 1H), 3.17 (dd, *J* = 12.9, 4.7 Hz, 1H), 3.33 (1H) signal overlapping with HDO peak, 3.47 (dd, *J* = 14.2, 7.4 Hz, 1H), 3.52–3.57 (m, 1H), 3.60–3.66 (m, 1H), 3.72–3.81 (m, 2H), 3.85–3.90 (m, 1H), 3.97–4.03 (m, 1H), 4.12–4.18 (m, 1H), 4.25 (dd, *J* = 8.8, 6.3 Hz, 1H), 4.30 (dd, *J* = 8.3, 4.3 Hz, 1H), 4.35–4.48 (m, 2H), 4.58–4.65 (m, 1H), 4.95 (t, *J* = 5.4 Hz, 1H), 4.97 (d, *J* = 4.8 Hz, 1H), 5.46 (t, *J* = 4.9 Hz, 1H), 6.62 (d, *J* = 8.4 Hz, 2H), 6.80 (s, 1H), 6.90 (d, *J* = 8.5 Hz, 2H), 7.18–7.28 (m, 4H), 7.27–7.33 (m, 2H), 7.76 (d, *J* = 8.8 Hz, 1H), 7.93–7.98 (m, 1H), 8.09 (s, 3H), 8.16 (d, *J* = 9.3 Hz, 1H), 8.34 (d, *J* = 7.7 Hz, 1H), 8.45–8.51 (m, 2H), 8.64 (d, *J* = 7.9 Hz, 1H), 9.20 (s, 1H).

***S. warneri* AIP-II (17)**

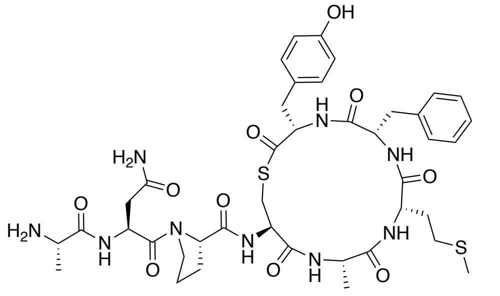

$C_{41}H_{55}N_{9}O_{10}S_2$
MW: 898.06

The peptide was synthesized according to general method A for on-resin cleavage-inducing cyclization (section 4.5). Purification by preparative RP-HPLC afforded *S. warneri* AIP-II (**17**) as a fluffy white solid after lyophilization. Purity >95% determined by UPLC (λ = 215 nm). **UPLC-MS** (ESI) *m/z* calcd for $[M+H]^+$ $C_{41}H_{56}N_{9}O_{10}S_2^+$: 898.36, found 898.14. Major conformer (conformers were observed in a ration of 1:0.1): **^{1}H NMR** (600 MHz, DMSO-d_6) δ = 1.23 (d, *J* = 7.4 Hz, 3H), 1.31 (d, *J* = 7.0 Hz, 3H), 1.75–1.84 (m, 3H), 1.86–1.93 (m, 2H), 1.99 (s, 3H), 2.00–2.06 (m, 1H), 2.13–2.27 (m, 2H), 2.42 (dd, *J* = 15.8, 7.6 Hz, 1H), 2.53–2.66 (m, 1H), 2.73 (dd, *J* = 14.2, 10.3 Hz, 1H), 2.79–2.97 (m, 3H), 3.10–3.23 (m, 2H), 3.66 (t, *J* = 6.9 Hz, 2H), 3.77–3.83 (m, 1H), 4.08–4.21 (m, 3H), 4.22–4.35 (m, 3H), 4.85 (q, *J* = 7.1 Hz, 1H), 6.64 (d, *J* = 8.0 Hz, 2H), 6.91 (d, *J* = 8.1 Hz, 2H), 6.99 (s, 1H), 7.06 (d, *J* = 7.4 Hz, 2H), 7.16–7.33 (m, 3H), 7.50 (s, 1H), 7.88 (d, *J* = 8.6 Hz, 1H), 7.97 (d, *J* = 7.6 Hz, 1H), 7.98–8.07 (m, 3H), 8.22 (d, *J* = 7.7 Hz, 1H), 8.30 (d, *J* = 8.0 Hz, 1H), 8.70 (d, *J* = 7.2 Hz, 1H), 8.83 (d, *J* = 8.4 Hz, 1H), 9.19 (s, 1H).

***S. cohnii* AIP-I (18)**

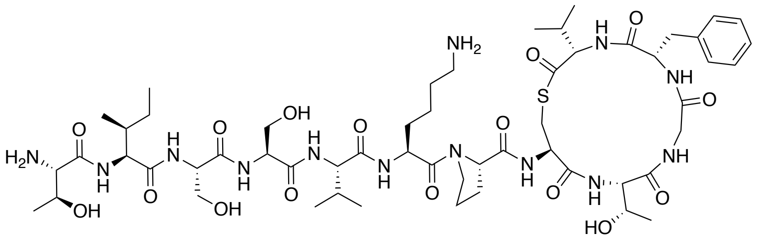

$C_{55}H_{89}N_{13}O_{16}S$
MW: 1220.45

The peptide was synthesized according to general method A for on-resin cleavage-inducing cyclization (section 4.5). Purification by preparative RP-HPLC afforded *S. cohnii* AIP-I (**18**) as a fluffy white solid after lyophilization. Purity 95% determined by UPLC (λ = 215 nm). **UPLC-MS** (ESI) *m/z* calcd for $[M+2H]^{2+}$ $C_{55}H_{91}N_{13}O_{16}S^{2+}$: 610.82, found 610.93; $[M+H]^{+}$ $C_{55}H_{90}N_{13}O_{16}S^{+}$: 1220.63, found 1220.69. **^{1}H NMR** (600 MHz, DMSO-d_6) δ = 0.80 (d, *J* = 6.7 Hz, 3H), 0.81–0.85 (m, 6H), 0.86 (d, *J* = 6.8 Hz, 3H), 0.90 (d, *J* = 6.8 Hz, 3H), 0.95 (d, *J* = 6.9 Hz, 3H), 0.99 (d, *J* = 6.2 Hz, 3H), 1.05–1.12 (m, 1H), 1.14 (d, *J* = 6.3 Hz, 3H), 1.34 –1.40 (m 2H), 1.44–1.59 (m, 4H), 1.62–1.69 (m, 1H), 1.69–1.77 (m, 2H), 1.80–1.92 (m, 2H), 1.93–2.04 (m, 2H), 2.37–2.45 (m, 1H), 2.63 (dd, *J* = 13.1, 10.8 Hz, 1H), 2.75–2.81 (m, 2H), 2.85 (dd, *J* = 14.2, 11.4 Hz, 1H), 3.21 (dd, *J* = 13.1, 4.8 Hz, 1H), 3.25 (dd, *J* = 14.2, 3.5 Hz, 1H), 3.41–3.46 (m, 1H), 3.50–3.61 (m, 4H), 3.61–3.71 (m, 4H), 3.76–3.83 (m, 1H), 3.86–3.91 (m, 1H), 4.19 (dd, *J* = 8.8, 6.2 Hz, 1H), 4.24 (dd, *J* = 9.1, 4.4 Hz, 1H), 4.32 (dd, *J* = 8.6, 7.1 Hz, 1H), 4.34–4.46 (m, 6H), 4.56 (dd, *J* = 9.9, 4.6 Hz, 1H), 4.76-4.80 (m, 1H), 4.95–4.99 (m, 1H), 5.05–5.09 (m, 1H), 5.52–5.58 (m, 1H), 7.19–7.24 (m, 1H), 7.24–7.34 (m, 5H), 7.65–7.75 (m, 4H), 7.89–7.96 (m, 2H), 8.03–8.10 (m, 3H), 8.11–8.16 (m, 2H), 8.19 (d, *J* = 7.7 Hz, 1H), 8.44 (d, *J* = 8.6 Hz, 1H), 8.53 (dd, *J* = 6.8, 4.6 Hz, 1H), 8.77 (d, *J* = 8.4 Hz, 1H).

***S. caprae* AIP-I (20)**

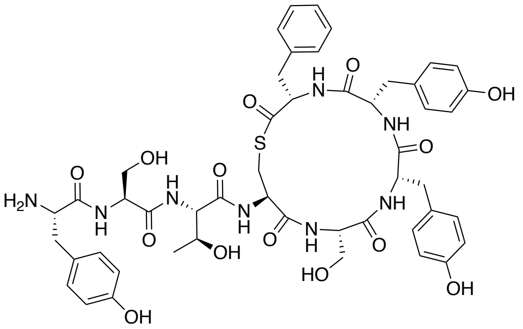

$C_{49}H_{58}N_8O_{14}S$
MW: 1015.11

The peptide was synthesized according to general method A for on-resin cleavage-inducing cyclization (section 3). Purification by preparative RP-HPLC afforded *S. caprae* AIP-I (**20**) as a fluffy white solid after lyophilization. Purity >95% determined by UPLC (λ = 215 nm). **UPLC-MS** (ESI) *m/z* calcd for $[M+H]^{+}$ $C_{49}H_{59}N_8O_{14}S^{+}$: 1015.39, found 1015.53. **^{1}H NMR** (600 MHz, DMSO-d_6) δ = 1.05 (d, *J* = 6.3 Hz, 3H), 2.62–2.72 (m, 2H), 2.73–2.86 (m, 4H), 2.90 (dd, *J* = 12.7, 11.0 Hz, 1H), 3.03 (dd, *J* = 14.3, 4.7 Hz, 1H), 3.20–3.28 (m, 2H), 3.35–3.39 (m, 1H), 3.44–3.50 (m, 1H), 3.54–3.61 (m, 1H), 3.65–3.72 (m, 1H), 4.01 (dd, *J* = 8.3, 4.8 Hz, 1H), 4.06–4.14 (m, 4H), 4.26 (dd, *J* = 8.5, 3.4 Hz, 1H), 4.36 (dt, *J* = 9.2, 7.0 Hz, 1H), 4.45 (ddd, *J* = 11.7, 8.1, 4.0 Hz, 1H), 4.55 (dt, *J* = 7.7, 6.2 Hz, 1H), 4.92 (d, *J* = 5.1 Hz, 1H), 5.03 (t, *J* = 5.3 Hz, 1H), 5.25 (t, *J* = 5.2 Hz, 1H), 6.60–6.66 (m, 4H), 6.69 (d, *J* = 8.5 Hz, 1H), 6.78 (d, *J* = 8.5 Hz, 2H), 6.89–6.96 (m, 4H), 7.07 (d, *J* = 8.5 Hz, 2H), 7.14–7.22 (m, 3H), 7.85 (d, *J* = 9.1 Hz, 1H), 7.86–7.99 (m, 3H), 7.97 (d, *J* = 8.5 Hz, 1H), 8.02 (d, *J* = 8.1 Hz, 1H), 8.18–8.23 (m, 2H), 8.72 (d, *J* = 7.7 Hz, 1H), 8.94 (d, *J* = 7.9 Hz, 1H), 9.22 (s, 1H), 9.24 (s, 1H), 9.34 (s, 1H).

***S. pasteuri* AIP-I (21)**

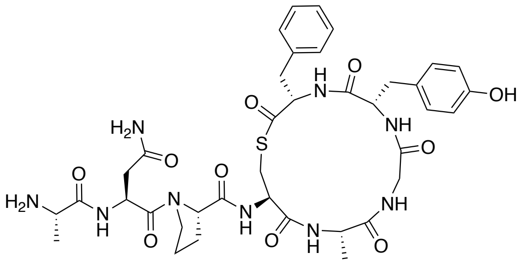

$C_{38}H_{49}N_9O_{10}S$
MW: 823.92

The peptide was synthesized according to general method A for on-resin cleavage-inducing cyclization (section 4.5). Purification by preparative RP-HPLC afforded *S. pasteuri* AIP-I (**21**) as a fluffy white solid after lyophilisation. Purity >95% determined by UPLC (λ = 215 nm). **UPLC-MS** (ESI) *m/z* calcd for $[M+H]^+$ $C_{38}H_{50}N_9O_{10}S^+$: 824.34, found 824.26. Major conformer (conformers were observed in a ration of 1:0.15): **^{1}H NMR** (600 MHz, DMSO-d_6) δ = 1.19 (d, *J* = 7.1 Hz, 3H), 1.31 (d, *J* = 7.0 Hz, 3H), 1.79–1.94 (m, 3H), 1.95–2.03 (m, 1H), 2.31 (dd, *J* = 14.3, 10.8 Hz, 1H), 2.42 (dd, *J* = 15.7, 7.9 Hz, 1H), 2.59 (dd, *J* = 15.7, 5.5 Hz, 1H), 2.63–2.72 (m, 2H), 2.97 (dd, *J* = 14.2, 11.2 Hz, 1H), 3.18 (dd, *J* = 13.0, 5.0 Hz, 1H), 3.30–3.39 (m, 2H), 3.62–3.67 (m, 2H), 3.78–3.88 (m, 2H), 4.14 (ddd, *J* = 11.5, 7.9, 4.0 Hz, 1H), 4.34–4.42 (m, 2H), 4.43–4.49 (m, 1H), 4.60 (ddd, *J* = 11.2, 9.3, 3.8 Hz, 1H), 4.81–4.88 (m, 1H), 6.62 (d, *J* = 8.4 Hz, 2H), 6.90 (d, *J* = 8.4 Hz, 2H), 6.99 (s, 1H), 7.20–7.24 (m, 1H), 7.25–7.33 (m, 4H), 7.48 (s, 1H), 8.03–8.10 (m, 4H), 8.21 (d, *J* = 9.4 Hz, 1H), 8.25 (d, *J* = 8.7 Hz, 1H), 8.48 (d, *J* = 8.0 Hz, 1H), 8.53 (t, *J* = 5.5 Hz, 1H), 8.70 (d, *J* = 7.3 Hz, 1H), 9.20 (br s, 1H).

***S. devriesei* AIP-I (22)**

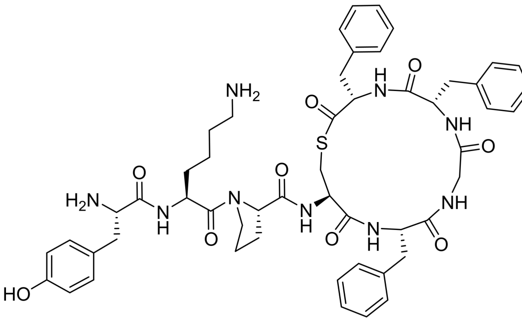

$C_{52}H_{63}N_9O_{10}S$
MW: 1006.19

The peptide was synthesized according to general method A for on-resin cleavage-inducing cyclization (section 4.5). Purification by preparative RP-HPLC afforded *S. devriesei* AIP-I (**22**) as a fluffy white solid after lyophilization. Purity >95% determined by UPLC (λ = 215 nm). **UPLC-MS** (ESI) *m/z* calcd for $[M+H]^+$ $C_{52}H_{64}N_9O_{10}S^+$: 1006.45, found 1006.71. Major conformer (conformers were observed in a ration of 1:0.14): **^{1}H NMR** (600 MHz, DMSO-d_6) δ = 1.34–1.47 (m, 2H), 1.48–1.66 (m, 4H), 1.68–1.76 (m, 1H), 1.77–1.89 (m, 2H), 1.89–2.00 (m, 1H), 2.31 (dd, *J* = 14.3, 10.9 Hz, 1H), 2.61 (dd, *J* = 12.9, 10.5 Hz, 1H), 2.68 (dd, *J* = 14.3, 3.8 Hz, 1H), 2.72–2.87 (m, 4H), 2.88–2.96 (m, 2H), 3.03 (dd, *J* = 14.4, 11.4 Hz, 1H), 3.19 (dd, *J* = 12.9, 4.9 Hz, 1H), 3.29–3.42 (m, 2H), 3.54 (t, *J* = 6.8 Hz, 2H), 3.86 (dd, *J* = 14.2, 4.7 Hz, 1H), 3.94–4.06 (m, 1H), 4.14 (ddd, *J* = 11.3, 8.0, 3.8 Hz, 1H), 4.38 (dd, *J* = 8.3, 4.3 Hz, 1H), 4.42 (ddd, *J* = 10.4, 8.2, 5.1 Hz, 1H), 4.51 (q, *J* = 7.4 Hz, 1H), 4.59–4.68 (m, 2H), 6.62 (d, *J* = 8.5 Hz, 2H), 6.69 (d, *J* = 8.4 Hz, 2H), 6.91 (d, *J* = 8.5 Hz, 2H), 6.99 (d, *J* = 8.5 Hz, 2H), 7.15–7.38 (m, 10H), 7.73–7.86 (m, 3H), 8.04–8.14 (m, 4H), 8.18 (d, *J* = 9.3 Hz, 1H), 8.39 (d, *J* = 9.1 Hz, 1H), 8.59 (d, *J* = 7.9 Hz, 1H), 8.67 (t, *J* = 5.5 Hz, 1H), 8.75 (d, *J* = 7.9 Hz, 1H), 8.96–9.62 (m, 2H).

***S. succinus* AIP-I (23)**

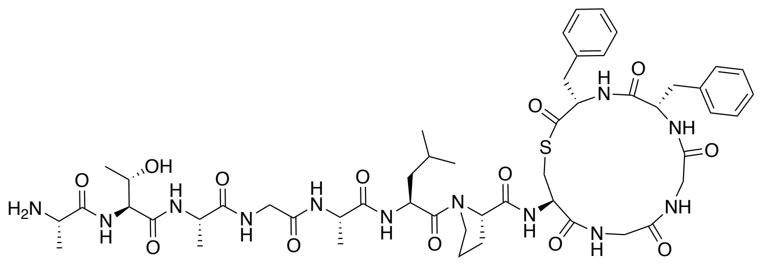

$C_{51}H_{72}N_{12}O_{13}S$
MW: 1093.27

The peptide was synthesized according to general method A for on-resin cleavage-inducing cyclization (section 4.5). Purification by preparative RP-HPLC afforded *S. succinus* AIP-I (**23**) as a fluffy white solid after lyophilization. Purity >95% determined by UPLC (λ = 215 nm). **UPLC-MS** (ESI) *m/z* calcd for $[M+2H]^{2+}$ $C_{51}H_{74}N_{12}O_{13}S^{2+}$: 547.26, found 547.39; $[M+H]^{+}$ $C_{51}H_{73}N_{12}O_{13}S^{+}$: 1093.51, found 1093.48. Major conformer (conformers were observed in a ration of 1:0.07): **^{1}H NMR** (600 MHz, DMSO-d_6) δ = 0.87–0.92 (m, 6H), 1.08 (d, *J* = 6.3 Hz, 3H), 1.18 (d, *J* = 7.0 Hz, 3H), 1.24 (d, *J* = 7.0 Hz, 3H), 1.35 (d, *J* = 6.9 Hz, 3H), 1.40–1.52 (m, 2H), 1.60–1.69 (m, 1H), 1.71–1.77 (m, 1H), 1.82–1.88 (m, 1H), 1.88–1.95 (m, 1H) 1.98–2.07 (m, 1H), 2.44 (dd, *J* = 14.3, 10.7 Hz, 1H), 2.69 (dd, *J* = 13.0, 10.2 Hz, 1H), 2.78 (dd, *J* = 14.3, 4.1 Hz, 1H), 2.93 (dd, *J* = 14.1, 11.0 Hz, 1H), 3.23 (dd, *J* = 13.0, 4.8 Hz, 1H), 3.30–3.37 (m, 2H), 3.42–3.51 (m, 2H), 3.62–3.75 (m, 3H), 3.79 (dd, *J* = 14.2, 4.4 Hz, 1H), 3.96–4.06 (m, 2H), 4.17 (dd, *J* = 15.6, 8.4 Hz, 1H), 4.22–4.33 (m, 4H), 4.34–4.40 (m, 2H), 4.50 (ddd, *J* = 9.6, 8.0, 4.8 Hz, 1H), 4.64 (ddd, *J* = 11.0, 9.2, 4.0 Hz, 1H), 7.08–7.12 (m, 2H), 7.16–7.20 (m, 1H), 7.20–7.28 (m, 5H), 7.29–7.34 (m, 2H), 7.85 (d, *J* = 7.5 Hz, 1H), 8.01 (d, *J* = 7.0 Hz, 1H), 8.03–8.07 (m, 4H), 8.17 (t, *J* = 5.8 Hz, 1H), 8.20 (d, *J* = 7.7 Hz, 1H), 8.31 (d, *J* = 9.3 Hz, 1H), 8.33–8.39 (m, 2H), 8.41 (d, *J* = 8.4 Hz, 1H), 8.51 (d, *J* = 8.0 Hz, 1H).

***S. equorum* AIP-I (24)**

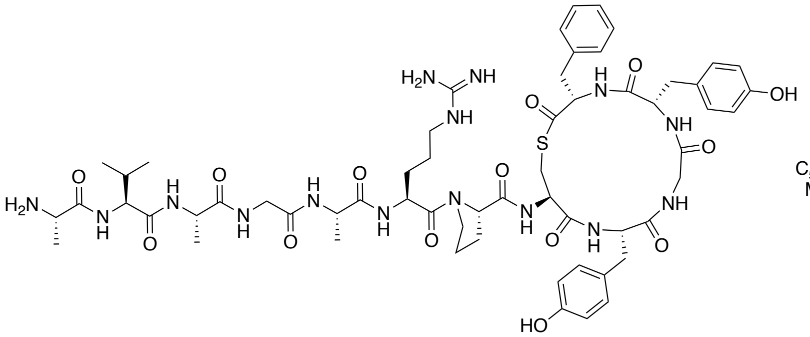

$C_{59}H_{81}N_{15}O_{14}S$
MW: 1256.45

The peptide was synthesized according to general method A for on-resin cleavage-inducing cyclization (section 4.5). Purification by preparative RP-HPLC afforded *S. equorum* AIP-I (**24**) as a fluffy white solid after lyophilization. Purity >95% determined by UPLC (λ = 215 nm). **UPLC-MS** (ESI) *m/z* calcd for $[M+H]^{+}$ $C_{59}H_{82}N_{15}O_{14}S^{+}$: 1256.59, found 1256.11. Major conformer (conformers were observed in a ration of 1:0.12): **^{1}H NMR** (600 MHz, DMSO-d_6) δ = 0.85 (d, *J* = 6.7 Hz, 3H), 0.89 (d, *J* = 6.7 Hz, 3H), 1.19 (d, *J* = 7.0 Hz, 3H), 1.19–1.25 (m, 3H), 1.32 (d, *J* = 6.9 Hz, 3H), 1.51–1.65 (m, 4H), 1.68–1.76 (m, 1H), 1.80–2.04 (m, 4H), 2.30 (dd, *J* = 14.1, 10.9 Hz, 1H), 2.57 (dd, *J* = 13.0, 10.6 Hz, 1H), 2.66–2.75 (m, 2H), 2.78 (dd, *J* = 14.0, 5.5 Hz, 1H), 2.98–3.07 (m, 1H), 3.07–3.21 (m, 3H), 3.25–3.32 (m, 1H), 3.32–3.38 (m, 1H), 3.49–3.59 (m, 1H), 3.60–3.70 (m, 2H), 3.74 (dd, *J* = 16.8, 5.8 Hz, 1H), 3.83 (dd, *J* = 14.1, 4.7 Hz, 1H), 3.89–3.99 (m, 1H), 4.10–4.18 (m, 1H), 4.18–4.23 (m, 1H), 4.22–4.29 (m, 1H), 4.27–4.33 (m, 1H), 4.32–4.37 (m, 1H), 4.37–4.44 (m, 1H), 4.44–4.52 (m, 1H), 4.51–4.58 (m, 1H), 4.57–4.64 (m, 1H), 6.58–6.63 (m, 4H), 6.70–7.41 (broad signal, 4H), 6.91 (d, *J* = 8.3 Hz, 2H), 7.01

(d, J = 8.4 Hz, 2H), 7.20–7.26 (m, 1H), 7.27–7.39 (m, 4H), 7.48–7.64 (m, 1H), 7.87 (d, J = 7.2 Hz, 1H), 8.02–8.07f (m, 3H), 8.08–8.21 (m, 5H), 8.27 (d, J = 9.1 Hz, 1H), 8.32 (d, J = 8.6 Hz, 1H), 8.51 (d, J = 7.9 Hz, 1H), 8.66 (d, J = 5.6 Hz, 1H), 9.05–9.28 (m, 2H).

***S. equorum* AIP-II (25)**

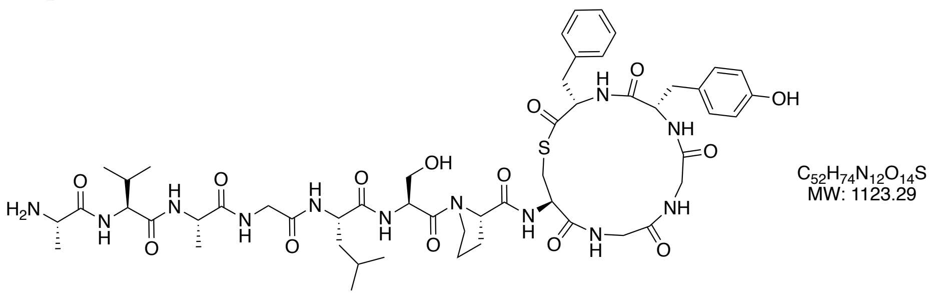

The peptide was synthesized according to general method A for on-resin cleavage-inducing cyclization (section 4.5). Purification by preparative RP-HPLC afforded *S. equorum* AIP-II (**25**) as a fluffy white solid after lyophilization. Purity >95% determined by UPLC (λ = 215 nm). **UPLC-MS** (ESI) *m/z* calcd for $[M+H]^+$ $C_{52}H_{75}N_{12}O_{14}S^+$: 1123.52, found 1123.27. Major conformer (conformers were observed in a ration of 1:0.45) **^{1}H NMR** (600 MHz, DMSO-d_6) δ = 0.79–0.83 (m, 3H), 0.83–0.88 (m, 6H), 0.86–0.91 (m, 3H), 1.21 (d, J = 7.1 Hz, 3H), 1.22–1.31 (m, 1H), 1.32 (d, J = 6.9 Hz, 3H), 1.33–1.42 (m, 1H), 1.40–1.49 (m, 1H), 1.49–1.64 (m, 1H), 1.78–2.06 (m, 4H), 2.08–2.22 (m, 1H), 2.27 (t, J = 8.8 Hz, 1H), 2.54–2.66 (m, 1H), 2.68–2.79 (m, 1H), 2.81–3.09 (m, 3H), 3.34 (t, J = 10.6 Hz, 1H), 3.50–3.59 (m, 3H), 3.60–3.73 (m, 2H), 3.74–4.02 (m, 4H), 4.04–4.17 (m, 1H), 4.17–4.53 (m, 6H), 4.53–4.72 (m, 2H), 4.99–5.13 (m, 1H), 6.66 (d, J = 8.4 Hz, 2H), 7.09–7.18 (m, 2H), 7.18–7.42 (m, 5H), 7.61 (d, J = 9.4 Hz, 1H), 7.85–8.02 (m, 2H), 8.02–8.18 (m, 5H), 8.32–8.41 (m, 2H), 8.55 (t, J = 6.3 Hz, 1H), 9.11–9.21 (m, 1H), 9.28–9.40 (m, 1H).

***S. intermedius* AIP-I (31)**

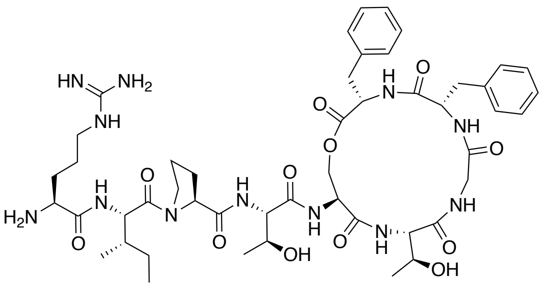

The peptide was synthesized as followed (Supporting Scheme S3). The fully protected linear peptide **S10** was synthesized in 40.0 µmol scale on Cl-Trt polystyrene resin **S7** preloaded with Fmoc-Phe-OH (0.69 mmol/g) using the general procedures for automated SPPS. After completed peptide elongation, the peptidyl-resin **S10** was transferred into a polypropylene syringe equipped with a fritted disk using CH_2Cl_2, washed with CH_2Cl_2 (3 × 1 min) and dried overnight under vacuum. The peptidyl-resin **S10** was swelled in anhydrous THF (1.5 mL) for 15 min and subsequently a solution of tetrabutylammonium fluoride (TBAF) in THF (1.0 M) (0.40 mL, 0.40 mmol, 10.0 equiv) in anhydrous THF (4.6 mL) was added to the resin and the resin was agitated at room temperature. After 1 h, the TBAF solution was removed by suction and resin treated with a fresh TBAF–THF solution. After 1 h, the TBAF solution was removed by suction and the resin washed with DMF (3 × 1 min), MeOH (3 × 1 min) and CH_2Cl_2 (3 × 1 min) and dried overnight under vacuum. On-resin esterification was performed according to a previously published protocol.(21) The resin was swelled in anhydrous CH_2Cl_2 (1.5 mL) for 15 min and subsequently a solution

of Fmoc-Phe-OH (93.0 mg, 0.24 mmol, 6.00 equiv), *N*,*N'*-diisopropylcarbodiimide (DIC) (37.6 µL, 0.24 mmol, 6.00 equiv), *N*-methylimidazole (NMI) (17.2 µL, 0.22 mmol, 5.40 equiv) in anhydrous CH_2Cl_2 (1.5 mL) was added. The resin was agitated at room temperature for 2 h and subsequently washed with anhydrous CH_2Cl_2 (3 × 1 min). A fresh Fmoc-Phe-OH–DIC–NMI solution was added to resin and after 2 h of incubation, the resin was washed with DMF (3 × 1 min), CH_2Cl_2 (3 × 1 min) and DMF (5 × 1 min). Fmoc-removal of the *O*-acylated peptidyl resin **S11** was performed by treatment of the resin with a solution of 1,8-biazabicyclo[5.4.0]undec-7-ene (DBU) in DMF (1.5 mL, 1:99, v/v) (8 × 30 s). The resin was subsequently washed with DMF (3 × 1 min), MeOH (3 × 1 min) and CH_2Cl_2 (3 × 1 min) and dried under suction for 15 min. The dried resin was treated with a solution of HFIP (0.4 mL) in CH_2Cl_2 (1.6 mL) for 15 min at room temperature to cleave the partially protected peptide **S12** from the resin. The cleavage solution was removed from the resin and collected and a fresh HFIP–CH_2Cl_2 solution was added to the resin. After 15 min the cleavage solution was removed from the resin and collected and the resin rinsed with CH_2Cl_2 (2.0 mL). The combined cleavage solutions and the rinsing solution were evaporated to dryness under reduced pressure to yield the partially protected peptide **S12,** which was used without further purification. Crude peptide **S12** (0.04 mmol based on the resin loading) was dissolved in anhydrous DMF (5.0 mL) under nitrogen atmosphere and added dropwise to a solution of HATU (15.2 mg, 0.04 mmol, 1.00 equiv) and *i*-Pr_2NEt (20.8 µL, 0.12 mmol, 3.00 equiv) in anhydrous DMF (35.0 mL). The reaction mixture was stirred overnight at room temperature and after full consumption of **S12** was confirmed by UPLC-MS, the reaction was reduced to dryness under reduced pressure. The remaining residue was treated with a deprotection cocktail (3.0 mL, TFA–*i*-Pr_3SiH–water, 94:3:3, v/v/v) for 2 h and subsequently concentrated under a stream of nitrogen followed by precipitation in ice-cold diethyl ether. Purification by preparative RP-HPLC afforded *S. intermedius* AIP-I (**31**) as a fluffy white solid after lyophilization. Purity >95% determined by UPLC (λ = 215 nm). **UPLC-MS** (ESI) *m/z* calcd for $[M+2H]^{2+}$ $C_{48}H_{72}N_{12}O_{12}^{2+}$: 504.27, found 504.53; $[M+H]^{+}$ $C_{48}H_{71}N_{12}O_{12}^{+}$: 1007.53, found 1007.68. Major conformer (conformers were observed in a ration of 1:0.12): **^{1}H NMR** (600 MHz, DMSO-*d*$_6$) δ = 0.81–0.86 (t, *J* = 7.4 Hz, 3H), 0.92 (d, *J* = 6.7 Hz, 3H), 1.03 (d, *J* = 6.3 Hz, 3H), 1.07 (d, *J* = 6.3 Hz, 3H), 1.08–1.13 (m, 1H), 1.41–1.51 (m, 2H), 1.51–1.59 (m, 1H), 1.60–1.71 (m, 1H), 1.72–1.88 (m, 3H), 1.88–1.96 (m, 1H), 1.96–2.05 (m, 1H), 2.82 (dd, *J* = 14.1, 11.5 Hz, 1H), 3.03 (dd, *J* = 13.8, 9.3 Hz, 1H), 3.06–3.16 (m, 3H), 3.26–3.32 (m, 1H), 3.60 (dt, *J* = 9.7, 6.6 Hz, 1H), 3.70–3.79 (m, 2H), 3.82–3.92 (m, 3H), 4.01–4.06 (m, 1H), 4.12–4.20 (m, 2H), 4.19–4.27 (m, 2H), 4.38 (t, *J* = 8.3 Hz, 1H), 4.43 (dd, *J* = 8.2, 4.6 Hz, 1H), 4.62 (td, *J* = 9.2, 6.2 Hz, 1H), 4.75–4.83 (m, 1H), 4.90–5.07 (m, 2H), 6.85–7.48 (4H) broad signal of guanidium group, 7.16–7.36 (m, 10H), 7.64 (d, *J* = 7.9 Hz, 1H), 7.70 (t, *J* = 6.0 Hz, 1H), 7.86 (d, *J* = 8.5 Hz, 1H), 7.93 (d, *J* = 9.1 Hz, 1H), 8.12–8.21 (m, 3H), 8.31 (d, *J* = 8.0 Hz, 1H), 8.39 (d, *J* = 7.2 Hz, 1H), 8.59 (d, *J* = 8.0 Hz, 1H), 9.02 (t, *J* = 5.6 Hz, 1H).

***S. simulans* AIP-II (33)**

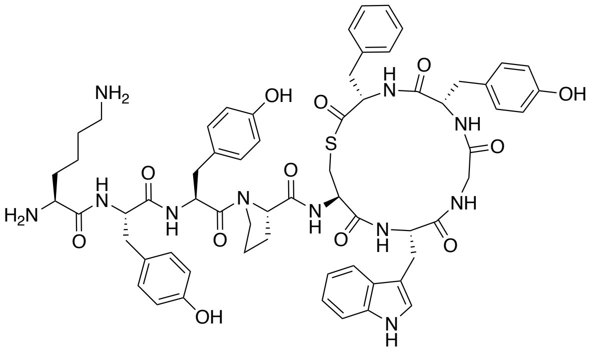

$C_{63}H_{73}N_{11}O_{12}S$
MW: 1208.40

The peptide was synthesized according to general method A for on-resin cleavage-inducing cyclization (section 4.5). Purification by preparative RP-HPLC afforded *S. simulans* AIP-II (**33**) as a fluffy white solid after lyophilization. Purity >95% determined by UPLC (λ = 215 nm). **UPLC-MS** (ESI) *m/z* calcd for $[M+2H]^{2+}$ $C_{63}H_{75}N_{11}O_{12}S^{2+}$: 604.77, found 605.07; $[M+H]^{+}$ $C_{63}H_{74}N_{11}O_{12}S^{+}$: 1208.52, found 1208.70. Major conformer (conformers were observed in a ration of 1:0.21): **^{1}H NMR** (600 MHz, DMSO-d_6) δ = 1.24–1.34 (m, 2H), 1.47–1.55 (m, 2H), 1.63–1.74 (m, 3H), 1.75–1.82 (m, 1H), 1.83–1.95 (m, 2H), 2.32 (dd, *J* = 14.2, 10.9 Hz, 1H), 2.57–2.78 (m, 6H), 2.86–2.99 (m, 3H), 3.00–3.09 (m, 2H), 3.27 (dd, *J* = 12.9, 5.0 Hz, 1H), 3.31–3.41 (3H) signals overlapping with HDO peak, 3.48–3.54 (m, 1H), 3.69–3.76 (m, 1H), 3.84 (dd, *J* = 14.0, 4.7 Hz, 1H), 4.11–4.19 (m, 1H), 4.33–4.38 (m, 1H), 4.44–4.53 (m, 2H), 4.52–4.60 (m, 1H), 4.59–4.66 (m, 1H), 4.65–4.72 (m, 1H), 6.59–6.69 (m, 6H), 6.89–6.93 (m, 2H), 6.94–6.99 (m, 1H), 7.01 (d, *J* = 8.5 Hz, 2H), 7.03–7.07 (m, 1H), 7.08–7.13 (m, 2H), 7.15 (d, *J* = 2.4 Hz, 1H), 7.20–7.26 (m, 1H), 7.26–7.37 (m, 5H), 7.59 (d, *J* = 7.9 Hz, 1H), 7.65–7.76 (m, 3H), 7.99–8.09 (m, 5H), 8.19 (d, *J* = 9.3 Hz, 1H), 8.34–8.40 (m, 2H), 8.46 (d, *J* = 8.2 Hz, 1H), 8.51 (d, *J* = 8.0 Hz, 1H), 8.73 (t, *J* = 5.6 Hz, 1H), 9.16–9.21 (m, 3H), 10.80 (d, *J* = 2.4 Hz, 1H).

***S. simulans* AIP-III (34)**

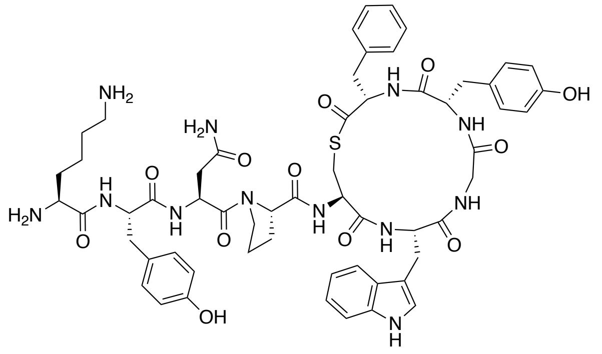

$C_{58}H_{70}N_{12}O_{12}S$
MW: 1159.33

The peptide was synthesized according to general method A for on-resin cleavage-inducing cyclization (section 4.5). Purification by preparative RP-HPLC afforded *S. simulans* AIP-III (**34**) as a fluffy white solid after lyophilization. Purity >95% determined by UPLC (λ = 215 nm). **UPLC-MS** (ESI) *m/z* calcd for $[M+2H]^{2+}$ $C_{58}H_{72}N_{12}O_{12}S^{2+}$: 580.25, found 580.46; $[M+H]^{+}$ $C_{58}H_{71}N_{12}O_{12}S^{+}$: 1159.50, found 1159.68. Major conformer (conformers were observed in a ration of 1:0.10): **^{1}H NMR** (600 MHz, DMSO-d_6) δ = 1.27–1.36 (m, 2H), 1.48–1.57 (m, 2H), 1.66–1.74 (m, 2H), 1.73–1.80 (m, 1H), 1.78–1.88 (m, 2H), 1.86–1.95 (m, 1H), 2.31 (dd, *J* = 14.2, 10.7 Hz, 1H), 2.38 (dd, *J* = 15.6, 7.1 Hz, 1H), 2.58–2.64 (m, 1H), 2.64–2.71 (m, 3H), 2.72–2.81 (m, 2H), 2.89 (dd, *J* = 14.1, 4.7 Hz, 1H), 2.94–3.08 (m, 3H), 3.16 (dd, *J* = 13.0, 4.9 Hz, 1H), 3.31–3.41 (2H) signals overlapping with HDO peak, 3.50–3.62 (m, 2H), 3.72–3.81 (m, 2H), 4.11–4.17 (m, 1H), 4.32–4.37 (m, 1H), 4.36–4.43 (m, 1H), 4.48–4.55 (m, 1H), 4.58–4.68 (m, 2H), 4.82 (q, *J* = 7.1 Hz, 1H), 6.60–6.67 (m, 4H), 6.90 (d, *J* = 8.4 Hz, 2H), 6.93–6.99 (m, 2H), 7.02 (d, *J* = 8.5 Hz, 2H), 7.03–7.07 (m, 1H), 7.16 (d, *J* = 2.3 Hz, 1H), 7.20–7.26 (m, 1H), 7.26–7.34 (m, 5H), 7.47 (s, 1H), 7.56 (d, *J* = 7.9 Hz, 1H), 7.68–7.76 (m, 3H), 8.03 (d, *J* = 8.0 Hz, 1H), 8.06–8.09 (m, 3H), 8.18–8.26 (m, 2H), 8.48–8.54 (m, 3H), 8.64 (t, *J* = 5.6 Hz, 1H), 9.13–9.35 (m, 2H), 10.81 (d, *J* = 2.5 Hz, 1H).

5.2 *S. simulans* AIP SAR study

The following peptide was characterized elsewhere: *S. aureus* AIP-III D4A (**57**) (8).

S. simulans AIP-II K1A (36)

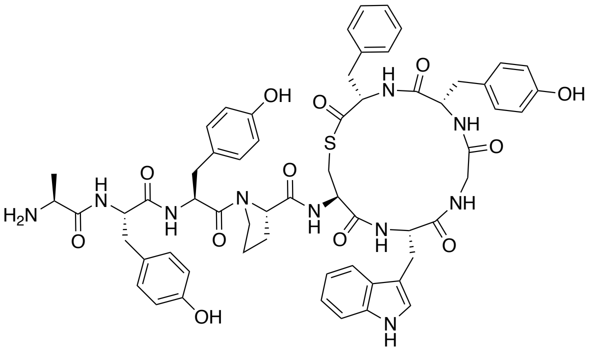

$C_{60}H_{66}N_{10}O_{12}S$
MW: 1151.31

The peptide was synthesized according to general method A for on-resin cleavage-inducing cyclization (section 4.5). Purification by preparative RP-HPLC afforded *S. simulans* AIP-II K1A (**36**) as a fluffy white solid after lyophilization. Purity >95% determined by UPLC (λ = 215 nm). **UPLC-MS** (ESI) *m/z* calcd for $[M+H]^+$ $C_{60}H_{67}N_{10}O_{12}S^+$: 1151.47, found 1151.45.

S. simulans AIP-II Y2A (37)

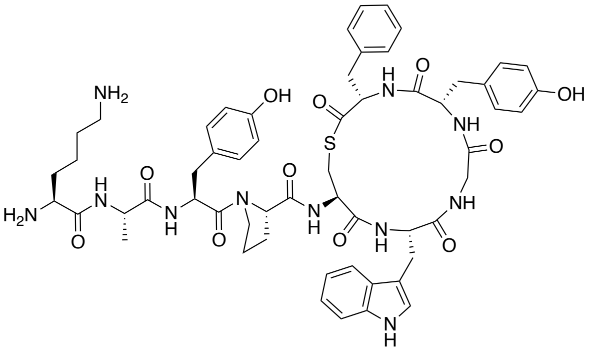

$C_{57}H_{69}N_{11}O_{11}S$
MW: 1116.31

The peptide was synthesized according to general method A for on-resin cleavage-inducing cyclization (section 4.5). Purification by preparative RP-HPLC afforded *S. simulans* AIP-II Y2A (**37**) as a fluffy white solid after lyophilization. Purity >95% determined by UPLC (λ = 215 nm). **UPLC-MS** (ESI) *m/z* calcd for $[M+H]^+$ $C_{57}H_{70}N_{11}O_{11}S^+$: 1116.50, found 1116.71.

S. simulans AIP-II/III Y3A/N3A (38)

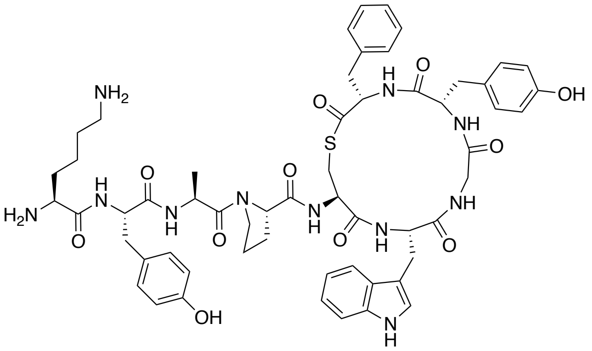

$C_{57}H_{69}N_{11}O_{11}S$
MW: 1116.31

The peptide was synthesized according to general method A for on-resin cleavage-inducing cyclization (section 4.5). Purification by preparative RP-HPLC afforded *S. simulans* AIP-II/III Y3A/N3A (**38**) as a fluffy white solid

after lyophilization. Purity >95% determined by UPLC (λ = 215 nm). **UPLC-MS** (ESI) *m/z* calcd for $[M+H]^+$ $C_{57}H_{70}N_{11}O_{11}S^+$: 1116.50, found 1116.65.

S. simulans **AIP-II P4A (39)**

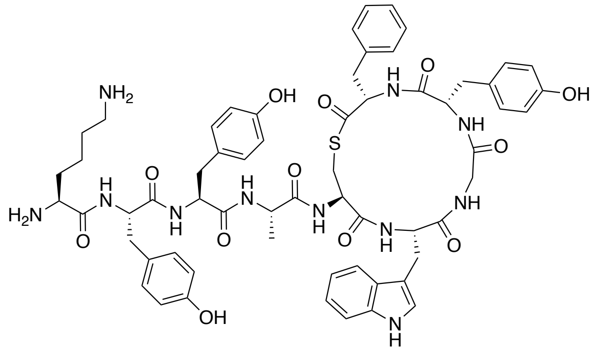

$C_{61}H_{71}N_{11}O_{12}S$
MW: 1182.36

The peptide was synthesized according to general method A for on-resin cleavage-inducing cyclization (section 4.5). Purification by preparative RP-HPLC afforded *S. simulans* AIP-II P4A (**39**) as a fluffy white solid after lyophilization. Purity >95% determined by UPLC (λ = 215 nm). **UPLC-MS** (ESI) *m/z* calcd for $[M+H]^+$ $C_{61}H_{72}N_{11}O_{12}S^+$: 1182.51, found 1182.76.

S. simulans **AIP-II W6A (40)**

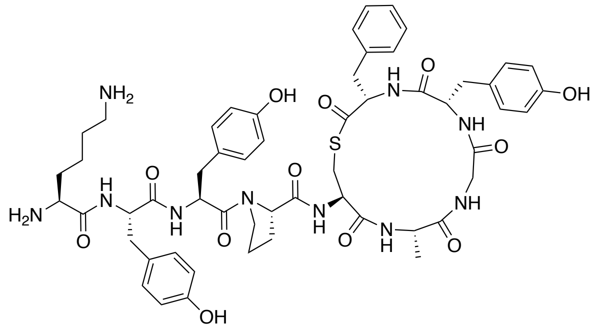

$C_{55}H_{68}N_{10}O_{12}S$
MW: 1093.27

The peptide was synthesized according to general method A for on-resin cleavage-inducing cyclization (section 4.5). Purification by preparative RP-HPLC afforded *S. simulans* AIP-II W6A (**40**) as a fluffy white solid after lyophilization. Purity >95% determined by UPLC (λ = 215 nm). **UPLC-MS** (ESI) *m/z* calcd for $[M+H]^+$ $C_{55}H_{69}N_{10}O_{12}S^+$: 1093.48, found 1093.55.

S. simulans **AIP-II G7A (41)**

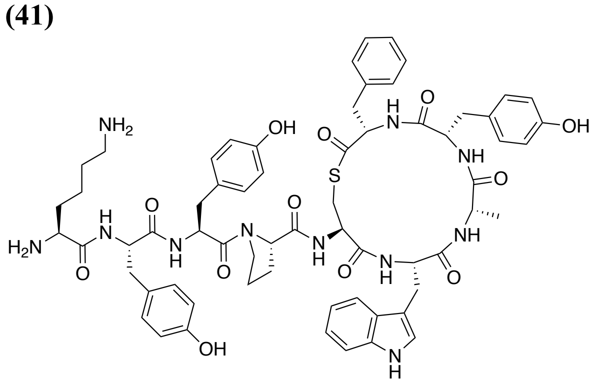

$C_{64}H_{75}N_{11}O_{12}S$
MW: 1222.43

The peptide was synthesized according to general method A for on-resin cleavage-inducing cyclization (section 4.5). Purification by preparative RP-HPLC afforded *S. simulans* AIP-II G7A (**41**) as a fluffy white solid after

lyophilization. Purity >95% determined by UPLC (λ = 215 nm). **UPLC-MS** (ESI) *m/z* calcd for $[M+H]^+$ $C_{64}H_{76}N_{11}O_{12}S^+$: 1222.54, found 1222.60.

S. simulans AIP-II Y8A (42)

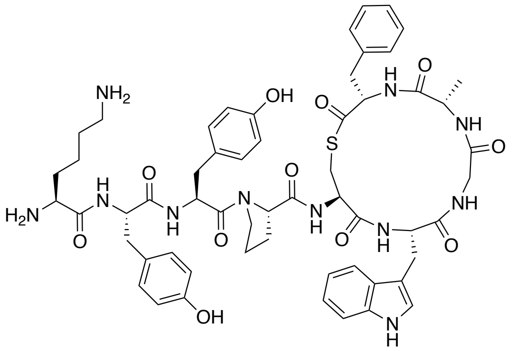

$C_{57}H_{69}N_{11}O_{11}S$
MW: 1116.31

The peptide was synthesized according to general method A for on-resin cleavage-inducing cyclization (section 4.5). Purification by preparative RP-HPLC afforded *S. simulans* AIP-II Y8A (**42**) as a fluffy white solid after lyophilization. Purity >95% determined by UPLC (λ = 215 nm). **UPLC-MS** (ESI) *m/z* calcd for $[M+H]^+$ $C_{57}H_{70}N_{11}O_{11}S^+$: 1116.50, found 1116.61.

S. simulans AIP-II F9A (43)

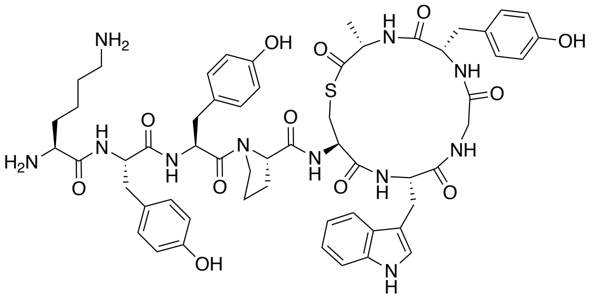

$C_{57}H_{69}N_{11}O_{12}S$
MW: 1132.30

The peptide was synthesized according to general method A for on-resin cleavage-inducing cyclization (section 4.5). Purification by preparative RP-HPLC afforded *S. simulans* AIP-II F9A (**43**) as a fluffy white solid after lyophilization. Purity >95% determined by UPLC (λ = 215 nm). **UPLC-MS** (ESI) *m/z* calcd for $[M+H]^+$ $C_{57}H_{70}N_{11}O_{12}S^+$: 1132.49, found 1132.64.

S. simulans AIP-II lactam (44)

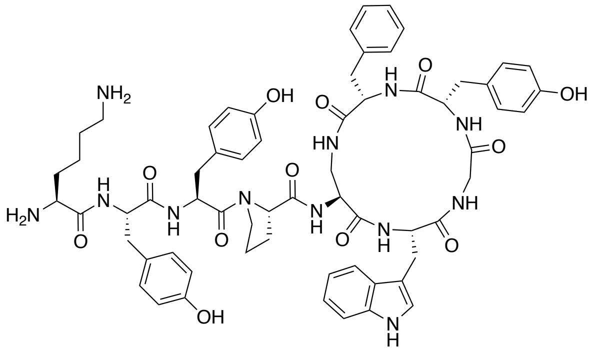

$C_{63}H_{74}N_{12}O_{12}$
MW: 1191.36

The peptide was synthesized as followed (Supporting Scheme S4). The fully protected linear peptide **S13** was synthesized in 40.0 µmol scale on Cl-Trt polystyrene resin **S7** preloaded with Fmoc-Phe-OH (0.69 mmol/g) using the general procedures for automated SPPS. After completed peptide elongation, the peptidyl-resin **S13** was transferred into a polypropylene syringe equipped with a fritted disk using CH_2Cl_2, washed with CH_2Cl_2 (3 × 1 min) and dried overnight under vacuum. The peptidyl-resin **S13** was treated twice with a solution of

$Pd(PPh_3)_4$ (4.60 mg, 4.00 µmol, 0.1 equiv) and dimethylborane (12.0 mg, 0.20 mmol, 5.00 equiv) in anhydrous CH_2Cl_2 (3.0 mL) for 15 min at room temperature. The resin was then washed with CH_2Cl_2 (3 × 1 min), DMF (3 × 1 min) and CH_2Cl_2 (3 × 1 min) and dried under suction for 15 min. The dried resin **S14** was treated with a solution of HFIP (0.4 mL) in CH_2Cl_2 (1.6 mL) for 15 min at room temperature to cleave the partially protected peptide **S15** from the resin. The cleavage solution was removed from the resin and collected and a fresh HFIP–CH_2Cl_2 solution was added to the resin. After 15 min the cleavage solution was removed from the resin and collected and the resin rinsed with CH_2Cl_2 (2.0 mL). The combined cleavage solutions and the rinsing solution were evaporated to dryness under reduced pressure to yield the partially protected peptide **S15,** which was used without further purification. Crude peptide **S15** (0.04 mmol based on the resin loading) was dissolved in anhydrous DMF (5.0 mL) under nitrogen atmosphere and added dropwise to a solution of HATU (15.2 mg, 0.04 mmol, 1.00 equiv) and *i*-Pr_2NEt (20.8 µL, 0.12 mmol, 3.00 equiv) in anhydrous DMF (35.0 mL). The reaction mixture was stirred overnight at room temperature and after full consumption of **S15** was confirmed by UPLC-MS, the reaction was reduced to dryness under reduced pressure. The remaining residue was treated with a deprotection cocktail (3.0 mL, TFA–*i*-Pr_3SiH–water, 94:3:3, v/v/v) for 2 h and subsequently concentrated under a stream of nitrogen followed by precipitation in ice-cold diethyl ether. Purification by preparative RP-HPLC afforded *S. simulans* AIP-II lactam (**44**) as a fluffy white solid after lyophilization. Purity >95% determined by UPLC ($\lambda = 215$ nm). **UPLC-MS** (ESI) *m/z* calcd for $[M+H]^+$ $C_{63}H_{75}N_{12}O_{12}^+$: 1191.56, found 1191.72.

***S. simulans* AIP-III K1A (45)**

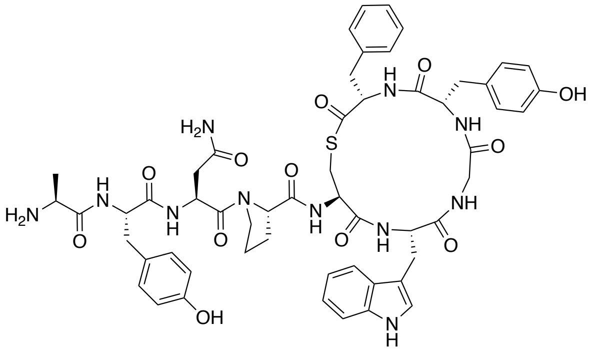

$C_{55}H_{63}N_{11}O_{12}S$
MW: 1102.23

The peptide was synthesized according to general method A for on-resin cleavage-inducing cyclization (section 4.5). Purification by preparative RP-HPLC afforded *S. simulans* AIP-III K1A (**45**) as a fluffy white solid after lyophilization. Purity >95% determined by UPLC ($\lambda = 215$ nm). **UPLC-MS** (ESI) *m/z* calcd for $[M+H]^+$ $C_{55}H_{64}N_{11}O_{12}S^+$: 1102.45, found 1102.42.

***S. simulans* AIP-III Y2A (46)**

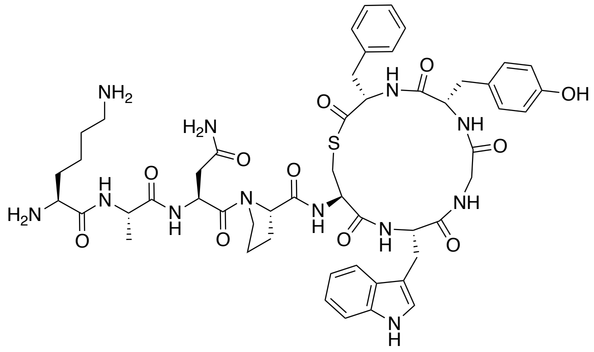

$C_{52}H_{66}N_{12}O_{11}S$
MW: 1067.23

The peptide was synthesized according to general method A for on-resin cleavage-inducing cyclization (section 4.5). Purification by preparative RP-HPLC afforded *S. simulans* AIP-III Y2A (**46**) as a fluffy white solid after lyophilization. Purity >95% determined by UPLC ($\lambda = 215$ nm). **UPLC-MS** (ESI) *m/z* calcd for $[M+H]^+$ $C_{52}H_{67}N_{12}O_{11}S^+$: 1067.48, found 1067.50.

***S. simulans* AIP-III P4A (47)**

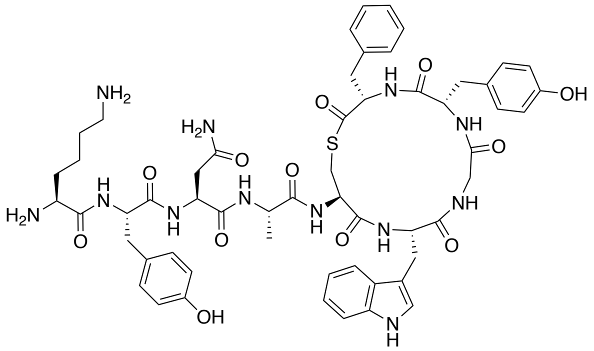

$C_{56}H_{68}N_{12}O_{12}S$
MW: 1133.29

The peptide was synthesized according to general method A for on-resin cleavage-inducing cyclization (section 4.5). Purification by preparative RP-HPLC afforded *S. simulans* AIP-III P4A (**47**) as a fluffy white solid after lyophilization. Purity >95% determined by UPLC ($\lambda = 215$ nm). **UPLC-MS** (ESI) *m/z* calcd for $[M+H]^+$ $C_{56}H_{69}N_{12}O_{12}S^+$: 1133.49, found 1133.67.

***S. simulans* AIP-III W6A (48)**

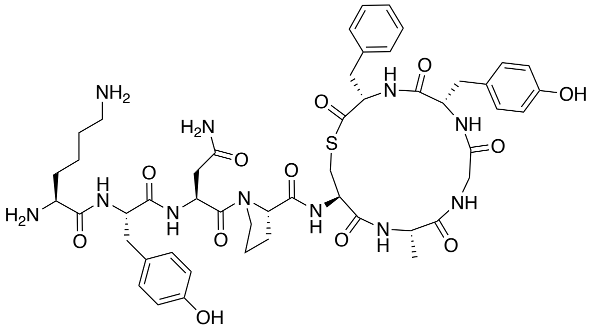

$C_{50}H_{65}N_{11}O_{12}S$
MW: 1044.20

The peptide was synthesized according to general method A for on-resin cleavage-inducing cyclization (section 4.5). Purification by preparative RP-HPLC afforded *S. simulans* AIP-III W6A (**48**) as a fluffy white solid after lyophilization. Purity >95% determined by UPLC ($\lambda = 215$ nm). **UPLC-MS** (ESI) *m/z* calcd for $[M+H]^+$ $C_{50}H_{66}N_{11}O_{12}S^+$: 1044.46, found 1044.48.

S. simulans **AIP-III G7A (49)**

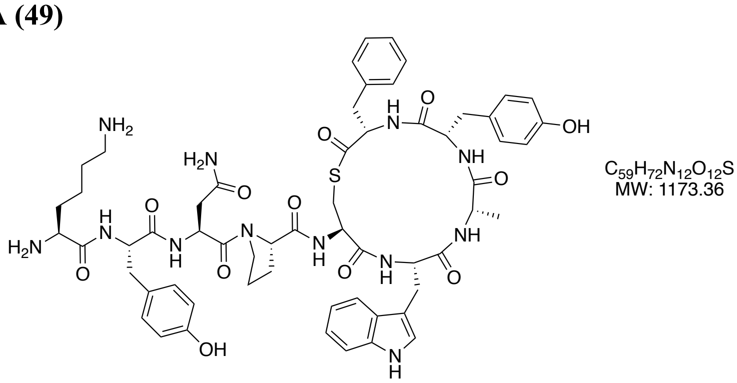

The peptide was synthesized according to general method A for on-resin cleavage-inducing cyclization (section 4.5). Purification by preparative RP-HPLC afforded *S. simulans* AIP-III G7A (**49**) as a fluffy white solid after lyophilization. Purity >95% determined by UPLC (λ = 215 nm). **UPLC-MS** (ESI) *m/z* calcd for $[M+H]^+$ $C_{59}H_{73}N_{12}O_{12}S^+$: 1173.52, found 1173.51.

S. simulans **AIP-III Y8A (50)**

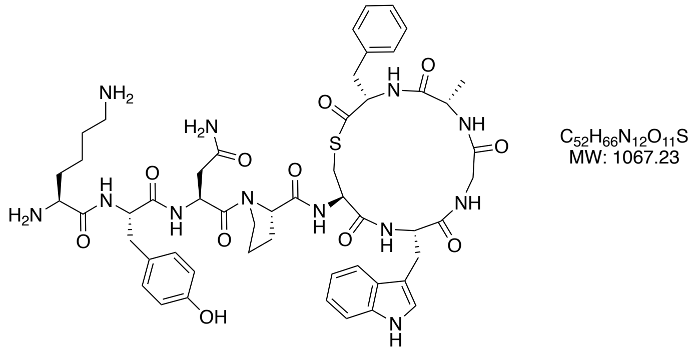

The peptide was synthesized according to general method A for on-resin cleavage-inducing cyclization (section 4.5). Purification by preparative RP-HPLC afforded *S. simulans* AIP-III Y8A (**50**) as a fluffy white solid after lyophilization. Purity >95% determined by UPLC (λ = 215 nm). **UPLC-MS** (ESI) *m/z* calcd for $[M+H]^+$ $C_{52}H_{67}N_{12}O_{11}S^+$: 1067.48, found 1067.61.

S. simulans **AIP-III F9A (51)**

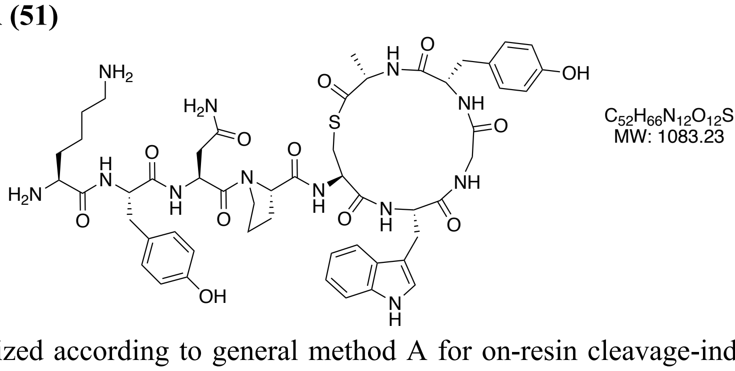

The peptide was synthesized according to general method A for on-resin cleavage-inducing cyclization (section 4.5). Purification by preparative RP-HPLC afforded *S. simulans* AIP-III F9A (**51**) as a fluffy white solid after lyophilization. Purity 93% determined by UPLC (λ = 215 nm). **UPLC-MS** (ESI) *m/z* calcd for $[M+H]^+$ $C_{52}H_{67}N_{12}O_{12}S^+$: 1083.47, found 1083.52.

***S. simulans* AIP-III 8-mer (52)**

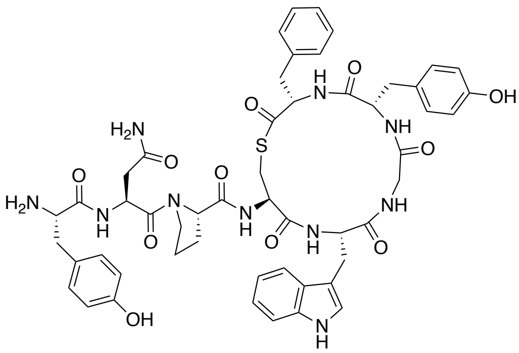

$C_{52}H_{58}N_{10}O_{11}S$
MW: 1031.16

The peptide was synthesized according to general method A for on-resin cleavage-inducing cyclization (section 4.5). Purification by preparative RP-HPLC afforded *S. simulans* AIP-III 8-mer (**52**) as a fluffy white solid after lyophilization. Purity >95% determined by UPLC ($\lambda = 215$ nm). **UPLC-MS** (ESI) *m/z* calcd for $[M+H]^+$ $C_{52}H_{59}N_{10}O_{11}S^+$: 1031.41, found 1031.67.

***S. simulans* AIP-III 7-mer (53)**

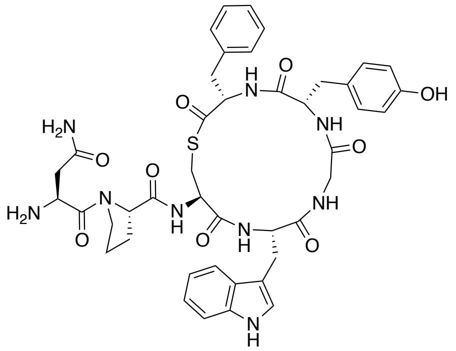

$C_{43}H_{49}N_{9}O_{9}S$
MW: 867.98

The peptide was synthesized according to general method A for on-resin cleavage-inducing cyclization (section 4.5). Purification by preparative RP-HPLC afforded *S. simulans* AIP-III 7-mer (**53**) as a fluffy white solid after lyophilization. Purity >95% determined by UPLC ($\lambda = 215$ nm). **UPLC-MS** (ESI) *m/z* calcd for $[M+H]^+$ $C_{43}H_{50}N_{9}O_{9}S^+$: 868.34, found 868.53.

***S. simulans* AIP-II/III 6-mer (54)**

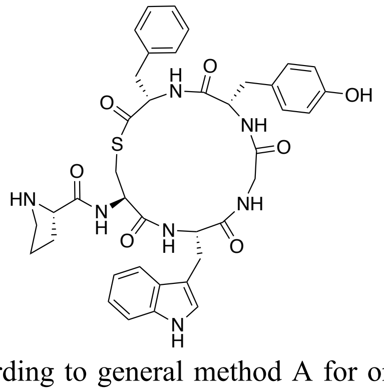

$C_{39}H_{43}N_{7}O_{7}S$
MW: 753.88

The peptide was synthesized according to general method A for on-resin cleavage-inducing cyclization (section 4.5). Purification by preparative RP-HPLC afforded *S. simulans* AIP-III 6-mer (**54**) as a fluffy white solid after lyophilization. Purity >95% determined by UPLC ($\lambda = 215$ nm). **UPLC-MS** (ESI) *m/z* calcd for $[M+H]^+$ $C_{39}H_{44}N_{7}O_{7}S^+$: 754.30, found 754.48.

***S. simulans* AIP-II/III 5-mer *N*-Ac (55)**

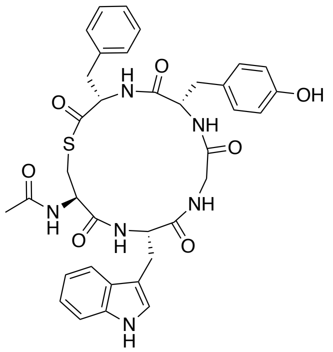

$C_{36}H_{38}N_6O_7S$
MW: 698.80

The peptide was synthesized according to general method A for on-resin cleavage-inducing cyclization (section 4.5). Purification by preparative RP-HPLC afforded *S. simulans* AIP-II/III 5-mer *N*-Ac (**55**) as a fluffy white solid after lyophilization. Purity >95% determined by UPLC (λ = 215 nm). **UPLC-MS** (ESI) *m/z* calcd for $[M+H]^+$ $C_{36}H_{39}N_6O_7S^+$: 699.26, found 699.41.

***S. simulans* AIP-II/III 5-mer *N*-Me₂ (56)**

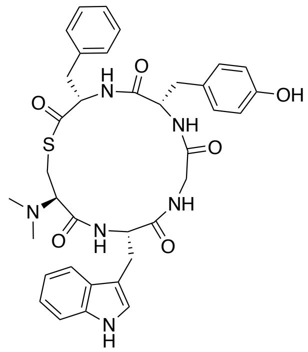

$C_{36}H_{40}N_6O_6S$
MW: 684.81

The peptide was synthesized according to general method A for on-resin cleavage-inducing cyclization (section 4.5). The amino acid Me_2N-Cys(Trt)-OH(22) was used for the synthesis. Purification by preparative RP-HPLC afforded *S. simulans* AIP-II/III 5-mer *N*-Me_2 (**56**) as a fluffy white solid after lyophilization. Purity >95% determined by UPLC (λ = 215 nm). **UPLC-MS** (ESI) *m/z* calcd for $[M+H]^+$ $C_{36}H_{41}N_6O_6S^+$: 685.28, found 685.42.

6. Supplementary references

1. **Gless BH, Bojer MS, Peng P, Baldry M, Ingmer H, Olsen CA**. 2019. Identification of autoinducing thiodepsipeptides from staphylococci enabled by native chemical ligation. *Nat Chem* 11:463-469.
2. **Mayville P, Ji G, Beavis R, Yang H, Goger M, Novick RP, Muir TW**. 1999. Structure-activity analysis of synthetic autoinducing thiolactone peptides from *Staphylococcus aureus* responsible for virulence. *Proc Natl Acad Sci USA* 96:1218-1223.
3. **Lyon GJ, Mayville P, Muir TW, Novick RP**. 2000. Rational design of a global inhibitor of the virulence response in *Staphylococcus aureus*, based in part on localization of the site of inhibition to the receptor-histidine kinase, AgrC. *Proc Natl Acad Sci USA* 97:13330-13335.
4. **Lyon GJ, Wright JS, Muir TW, Novick RP**. 2002. Key determinants of receptor activation in the *agr* autoinducing peptides of *Staphylococcus aureus*. *Biochemistry* 41:10095-10104.
5. **Tal-Gan Y, Stacy DM, Foegen MK, Koenig DW, Blackwell HE**. 2013. Highly potent inhibitors of quorum sensing in *Staphylococcus aureus* revealed through a systematic synthetic study of the group-III autoinducing peptide. *J Am Chem Soc* 135:7869-7882.
6. **Johnson JG, Wang B, Debelouchina GT, Novick RP, Muir TW**. 2015. Increasing AIP macrocycle size reveals key features of *agr* activation in *Staphylococcus aureus*. *ChemBioChem* 16:1093-1100.
7. **Tal-Gan Y, Stacy DM, Blackwell HE**. 2014. N-Methyl and peptoid scans of an autoinducing peptide reveal new structural features required for inhibition and activation of AgrC quorum sensing receptors in *Staphylococcus aureus*. *Chem Commun* 50:3000-3003.
8. **Gless BH, Peng P, Pedersen KD, Gotfredsen CH, Ingmer H, Olsen CA**. 2017. Structure-activity relationship study based on autoinducing peptide (AIP) from dog pathogen *S. schleiferi*. *Org Lett* 19:5276–5279.
9. **Otto M, Echner H, Voelter W, Götz F**. 2001. Pheromone Cross-Inhibition between *Staphylococcus aureus* and *Staphylococcus epidermidis*. *Infect Immun* 69:1957-1960.
10. **Tal-Gan Y, Ivancic M, Cornilescu G, Blackwell HE**. 2016. Characterization of structural elements in native autoinducing peptides and non-native analogues that permit the differential modulation of AgrC-type quorum sensing receptors in Staphylococcus aureus. *Org Biomol Chem* 14:113-21.
11. **Yang T, Tal-Gan Y, Paharik AE, Horswill AR, Blackwell HE**. 2016. Structure-function analyses of a *Staphylococcus epidermidis* autoinducing peptide reveals motifs critical for AgrC-type receptor modulation. *ACS Chem Biol* 11:1982-91.
12. **Williams MR, Costa SK, Zaramela LS, Khalil S, Todd DA, Winter HL, Sanford JA, O'Neill AM, Liggins MC, Nakatsuji T, Cech NB, Cheung AL, Zengler K, Horswill AR, Gallo RL**. 2019. Quorum sensing between bacterial species on the skin protects against epidermal injury in atopic dermatitis. *Sci Transl Med* 11:eaat8329.
13. **Severn MM, Williams MR, Shahbandi A, Bunch ZL, Lyon LM, Nguyen A, Zaramela LS, Todd DA, Zengler K, Cech NB, Gallo RL, Horswill AR**. 2022. The Ubiquitous Human Skin Commensal Staphylococcus hominis Protects against Opportunistic Pathogens. *mBio* 13:e00930-22.
14. **Severn MM, Cho Y-SK, Manzer HS, Bunch ZL, Shahbandi A, Todd DA, Cech NB, Horswill AR**. 2022. The Commensal Staphylococcus warneri Makes Peptide Inhibitors of MRSA Quorum Sensing that Protect Skin from Atopic or Necrotic Damage. *J Invest Dermatol* 142:3349-3352.e5.
15. **Paharik AE, Parlet CP, Chung N, Todd DA, Rodriguez EI, Van Dyke MJ, Cech NB, Horswill AR**. 2017. Coagulase-negative staphylococcal strain prevents *Staphylococcus aureus* colonization and skin infection by blocking quorum sensing. *Cell Host Microbe* 22:1-11.
16. **Ji G, Pei W, Zhang L, Qiu R, Lin J, Benito Y, Lina G, Novick RP**. 2005. Staphylococcus intermedius Produces a Functional agr Autoinducing Peptide Containing a Cyclic Lactone. *J Bacteriol* 187:3139-3150.
17. **Brown MM, Kwiecinski JM, Cruz LM, Shahbandi A, Todd DA, Cech NB, Horswill AR**. 2020. Novel Peptide from Commensal *Staphylococcus simulans* Blocks Methicillin-Resistant *Staphylococcus aureus* Quorum Sensing and Protects Host Skin from Damage. *Antimicrob Agents Chemother* 64:e00172-20.

18. **Olson ME, Todd DA, Schaeffer CR, Paharik AE, Van Dyke MJ, Büttner H, Dunman PM, Rohde H, Cech NB, Fey PD, Horswill AR**. 2014. *Staphylococcus epidermidis agr* quorum-sensing system: signal identification, cross talk, and importance in colonization. *J Bacteriol* 196:3482-93.
19. **Gordon CP, Olson SD, Lister JL, Kavanaugh JS, Horswill AR**. 2016. Truncated Autoinducing Peptides as Antagonists of *Staphylococcus lugdunensis* Quorum Sensing. *J Med Chem* 59:8879-8888.
20. **Malone CL, Boles BR, Lauderdale KJ, Thoendel M, Kavanaugh JS, Horswill AR**. 2009. Fluorescent reporters for Staphylococcus aureus. *J Microbiol Methods* 77:251-260.
21. **Bejder BS, Monda F, Gless BH, Bojer MS, Ingmer H, Olsen CA**. 2024. A short-lived peptide signal regulates cell-to-cell communication in Listeria monocytogenes. *Commun Biol* 7:942.
22. **Khoo KK, Galleano I, Gasparri F, Wieneke R, Harms H, Poulsen MH, Chua HC, Wulf M, Tampé R, Pless SA**. 2020. Chemical modification of proteins by insertion of synthetic peptides using tandem protein trans-splicing. *Nat Commun* 11:2284.
23. **Coin I, Beyermann M, Bienert M**. 2007. Solid-phase peptide synthesis: from standard procedures to the synthesis of difficult sequences. *Nat Protoc* 2:3247-3256.
